# Phosphoproteomics to Characterize Host Response During H3N2 Canine Influenza Virus Infection of Dog Lung

**DOI:** 10.3389/fvets.2020.585071

**Published:** 2020-12-03

**Authors:** Yongbo Liu, Cheng Fu, Shaotang Ye, Yingxin Liang, Zhonghe Qi, Congwen Yao, Zhen Wang, Ji Wang, Siqi Cai, Shiyu Tang, Ying Chen, Shoujun Li

**Affiliations:** ^1^College of Veterinary Medicine, South China Agricultural University, Guangzhou, China; ^2^Guangdong Provincial Key Laboratory of Prevention and Control for Severe Clinical Animal Diseases, Guangzhou, China; ^3^Guangdong Technological Engineering Research Center for Pet, Guangzhou, China; ^4^College of Animal Science & Technology, Zhongkai University of Agriculture and Engineering, Guangzhou, China

**Keywords:** go, KEGG, dog, phosphoproteomics, H3N2, canine influenza

## Abstract

Avian-origin H3N2 canine influenza viruses (CIVs) cause severe contagious respiratory disease in dogs, and quickly adapt to new environments. To further understand the mechanism of virus infection and host-virus interactions, we characterized the complete phosphoproteome of dogs infected with H3N2 CIV. Nine-week-old Beagle dogs were inoculated intranasally with 10^6^ EID_50_ of A/canine/Guangdong/04/2014 (H3N2) virus. Lung sections were harvested at 5 days post-inoculation (dpi) and processed for global and quantitative analysis of differentially expressed phosphoproteins. A total of 1,235 differentially expressed phosphorylated proteins were identified in the dog lung after H3N2 CIV infection, and 3,016 modification sites were identified among all differentially expressed proteins. We then performed an enrichment analysis of functional annotations using Kyoto Encyclopedia of Genes and Genomes (KEGG) and gene ontology (GO) database analyses to predict the functions of the identified differential phosphoproteins. Our data indicate that H3N2 CIV infection causes dramatic changes in the host protein phosphorylation of dog lungs. To our knowledge, this is the first study to assess the effect of H3N2 CIV infection on the phosphoproteome of beagles. These data provide novel insights into H3N2-CIV-triggered regulatory phosphorylation circuits and signaling networks and may improve our understanding of the mechanisms underlying CIV pathogenesis in dogs.

## Introduction

Influenza A is a highly contagious disease caused by influenza A virus (IAV) that has led to severe local and pandemic disease outbreaks, seriously threatening human and animal health (1). Waterfowl are natural reservoir hosts for IAV, while the virus can be isolated from a wide variety of species, including humans, pigs, horses, and other mammals and birds. Avian influenza viruses are considered to be the precursors of human influenza A viruses (2), which may be transmitted directly from their avian reservoirs, or infect other mammalian species such as swine before subsequent transmission to human hosts (3). Although generally poorly transmissible, IAVs frequently spill over from their reservoirs in aquatic birds and infect humans (4, 5). The frequent genetic mutations and wide host spectrum are the main characteristics of IAVs, and the constant cross-species transmission is a constant threat to humans and other mammals (6, 7).

Canine influenza virus (CIV) is a new branch of IAVs that has become fully adapted to mammals and shows mammalian cross-species risk (8, 9). In dogs, CIV causes respiratory system diseases, mainly sneezing, fever, cough, and depression, or even death (10, 11). The equine-origin H3N8 CIV caused respiratory disease outbreaks among racing greyhounds between 2004 and 2006 in the United States (12, 13). In 2007, an outbreak of avian-origin H3N2 CIV was first reported in South Korea (14), which occurred via interspecies transmission from avian hosts to dogs (15). Subsequently, Li et al. reported that H3N2 CIV has been circulating in China and Asia since 2006 (16). In 2015, an outbreak of H3N2 CIV originating from China occurred in the United States (17, 18), and more than 1,000 dogs were infected. H3N2 CIV can infect domestic cats, although its spread in the cat population is weak (19, 20). Although there is no evidence that dogs could be mixed containers of IAVs, they are susceptible to multiple subtypes of IAVs (21). Interspecies transmission of IAVs from humans to dogs, horses to dogs, and avian hosts to dogs has been frequently observed. CIVs are the result of successful cross-species virus transfers, and various IAVs (including two enzootic CIV subtypes) have been shown to infect dogs. Thus, the unique relationship between dogs and humans has raised concerns that CIVs may pose a potential risk to public health (22).

How has the avian H3N2 influenza virus gained the ability to infect dogs and become highly adaptable in dog hosts? Would this new avian-origin virus infect new mammals other than domestic cats, and would the virulence increase to cause a large number of dog deaths? These aspects remain unclear. A thorough understanding of the interactions between the canine dog host and virus and the determinants of pathogenesis is essential to study the mechanisms of disease in dogs and the host adaption procedure of IAVs.

CIV infection triggers massive changes in the host body, including the cells, metabolism, and molecules. Proteomics and genomics can shed light on these disease mechanisms. Tao et al. assessed changes in the complete transcriptome of MDCK cells after infection with H3N2 CIV to search for differentially expressed RNAs (23) and identified the significantly expressed RNAs involved in the whole process, including those associated with signal transduction, energy conversion, metabolic regulation, innate immunity, ionic channel, etc. Su et al. performed global and quantitative proteomics to identify host proteins that were differentially expressed upon inoculation of H3N2 CIV in dogs and identified 278 host proteins that were differentially expressed at different time points after inoculation (21). Nevertheless, many proteins rely on reversible modification by post-translational modifications (PTMs) such as phosphorylation, ubiquitination, and SUMOylation to perform their functions (24, 25).

Phosphorylation is one of the main PTMs, and it mediates many aspects of cell function, such as the formation of multiprotein assemblies, as well as protein stabilities and enzymatic activities (26). The phosphorylation patterns triggered by IAVs have attracted considerable attention in studies on the dynamic changes in proteins in IAV-infected cells (25). Mass spectrometry (MS)-based phosphoproteome analyses show advantages in studying the interaction mechanisms of host and virus, such as DNA viruses (27, 28) and RNA viruses (24, 29, 30). However, at present, there are no comprehensive phosphorylation proteomics analyses of the lungs of dogs infected with H3N2 CIV.

In this study, we characterized the phosphoproteomic changes in CIV-infected dog lungs in order to further understand the interactions between virus infection and host reaction. We identified more than 1,200 host phosphoproteins that were induced or repressed in infected lungs, indicating that numerous definable phosphopeptides are regulated during CIV infection. Simultaneously, 3,016 modification sites were identified among all the differentially expressed proteins. This study may improve our understanding of the CIV infection process and the host phosphorylation dynamics for blocking and limiting viral replication mechanisms.

## Materials and Methods

### Ethics Statement

All procedures in the animal experiments were approved by the South China Agricultural University Experimental Animal Welfare Ethics Committee (permit number, SYXK (YUE) 2018-0007).

### Virus and Viral Inoculation

**Figure 1:**
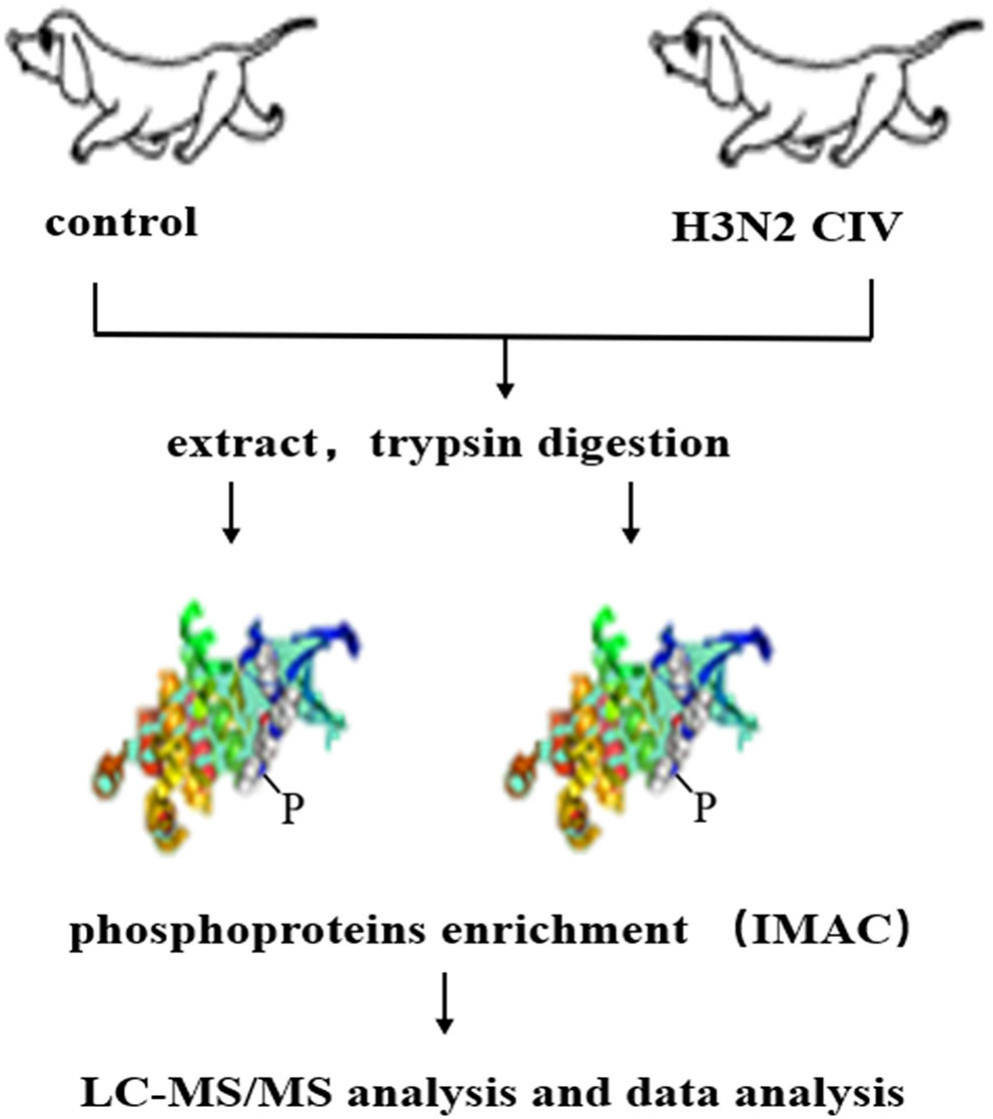
Schematic display of the workflow employed for the phosphoproteomic screen. Extracted proteins were digested with trypsin and were used to enrich peptides phosphorylated on Ser/Thr or Tyr using immobilized metal affinity chromatography (IMAC). Enriched phosphopeptides were analyzed in LC-MS/MS runs, and the original MS/MS file data were submitted to ProteinPilot Software v4.5 for data analysis.

CIV, A/canine/Guangdong/04/2014 (H3N2), was isolated from a dog with respiratory symptoms at Guangdong in 2014. Six 9-week-old beagles were purchased from Guangzhou General Pharmaceutical Research Institute Co., Ltd, Guangzhou, China. Hemagglutination-inhibition (HI) assay was conducted prior to the experiment to ensure that the dogs were sero-naïve for H3N2 CIV. After anesthesia with Quanmianbao (main active ingredients, N-(2,6-dimethylphenyl)-4, 5-dihydro-1, 3-thiazol-2-amine, haloperidol, and dihydroetophine hydrochloride; Northeast Agricultural University, CHN), three dogs were infected intranasally with 10 ^6^ EID_50_ /mL of the H3N2 CIV (WT group, WT), while the other three were infected intranasally with 1 mL of sterile phosphate-buffered saline (PBS) as control group (Ctrol). At 5 days post-infection (dpi), all dogs were euthanized through a pentobarbital overdose. Lung tissues with lesions were sampled in the lobus of the right lung in the infected group and the corresponding site in the control group were chosen for the next process (21). The subsequent experiment flow is shown in Figure 1.

### Protein Extraction

Lung tissues were collected and used for the following measures. The samples were obtained at −80°C, the appropriate amount of tissue sample was weighed into a liquid nitrogen pre-cooled mortar, and liquid nitrogen was added to grind it to a powder form. Next, 4 volumes of protein lysate (8 M Urea/100 mM Tris-HCl/1 mM PMSF/1×PhosSTOP; pH 8.0) were added to each group of samples and mixed before incubating the samples on ice for 5 min. Next a final concentration of 2 mM EDTA was added along with 10 mM dithiothreitol (DTT), and the samples were subjected to ultrasonication on an ice bath for 15 min. They were then centrifuged at 13,000 × *g*, at 4°C for 20 min, the supernatant was collected and transferred to a new centrifuge tube; four volumes of cold acetone were added to the centrifuge tube, and the solution was allowed to stand at −20°C overnight. The collected protein was precipitated by centrifugation and air dried, 8 M urea/100 mM Triethylammonium bicarbonate (TEAB) (pH 8.0) solution was added to re-dissolve the protein, DTT was added to a final concentration of 10 mM, and a reduction reaction was performed in a 56°C water bath for 30 min. Then, iodoacetamide (IAM) was added to a final concentration of 55 mM, and the solution was placed in a dark place at room temperature for 30 min to facilitate the alkylation reaction. Protein concentration was measured using the Bradford method (31).

### Trypsin Digestion

Equal amounts of protein from each sample were used for trypsin digestion. We obtained 1 mg of protein for trypsin digestion. After diluting the protein solution five times with 100 mM TEAB, trypsin was added at a mass ratio of 1:50 (trypsin:protein) and digested overnight at 37°C (12–16 h). The peptide fragments after enzymatic hydrolysis were desalted with a C18 column, and the desalted peptides dried with vacuum concentration meter.

### iTRAQ Labeling and Fractionation

The peptide was dissolved with 0.5M TEAB, labeled according to the instructions of iTRAQ-8 standard kit (SCIEX, MA, USA), mixed with the sample after labeling, and then used in the Ultimate 3000 HPLC system (Thermo DINOEX, USA) to classify the peptide samples separately. Samples were iTRAQ labeled as follows: WT-1, 115; WT-2, 116; WT-3, 117; Ctrol-1, 118; Ctrol-2, 119; and Ctrol-3, 121. The column used was a Durashell C18 column (5 μm, 100 Å, 4.6 × 250 mm). Separation of peptides was achieved using a gradually increasing acetonitrile (ACN) concentration under alkaline conditions, with a flow rate of 1 mL/min, and one tube was collected every minute. A total of 42 secondary fractions were collected and combined into 6 components. The combined components were desalted on a Strata-X column and dried in vacuum.

### Phosphopeptide Enrichment

The peptide was dissolved in the enrichment buffer solution (50% acetonitrile/6% trifluoroacetic acid); the supernatant was transferred to the IMAC material washed in advance, and the solution was incubated on a rotary shaker with gentle shaking. After the incubation, the resin was washed three times with buffer solution containing 50% acetonitrile/6% trifluoroacetic acid and 30% acetonitrile/0.1% trifluoroacetic acid. To elute the enriched phosphopeptides from the IMAC microspheres, an elution buffer containing 10% NH_4_OH was added, and the enriched phosphopeptides were eluted with vibration (32). The supernatant containing phosphopeptides was collected and lyophilized for liquid-mass spectrometry (LC-MS/MS) analysis.

### LC-MS/MS Analysis

The mass spectrometry data was collected using the TripleTOF 5600 + LC/MS system (SCIEX, MA, USA). The peptide samples were dissolved in 2% acetonitrile/0.1% formic acid and analyzed using a TripleTOF 5600+ mass spectrometer coupled with the Eksigent nanoLC system (SCIEX, MA, USA). The peptide solution was added to a C18 capture column (5 μm, 100 μm × 20 mm), and gradient elution was performed on a C18 analytical column (3 μm, 75 μm × 150 mm) with a 90-min gradient and a flow rate of 300 nL/min. The two mobile phases were buffer A (2% acetonitrile/0.1% formic acid/98% H_2_O) and buffer B (98% acetonitrile/0.1% formic acid/2% H_2_O). For IDA (Information Dependent Acquisition), the MS spectrum is scanned with an ion accumulation time of 250 ms, and the MS spectrum of 30 precursor ions was acquired with an ion accumulation time of 50 ms. MS1 spectra were collected in the range of 350–1,500 m/z, and MS2 spectra were collected in the range of 100–1,500 m/z. Precursor ions were excluded from reselection for 15 s.

### Data Analysis

The original MS/MS file data has been submitted to ProteinPilot Software v4.5 for data analysis. For protein identification, the Paragon algorithm (33) that was integrated into ProteinPilot was employed against uniprot-canis-lupus-familiaris (https://www.uniprot.org/uniprot/?query=canis%20lupus%20familiaris&fil=organism%3A%22Canis+lupus+familiaris+%28Dog%29+%28Canis+familiaris%29+%5B9615%5D%22&sort=score) for database searching. The parameters were set as follows: iTRAQ quantification; instrument, TripleTOF 5600; ID focus, biological modifications; cysteine modified with iodoacetamide; trypsin digestion; protein quantification and normalization, Quantitate, Bias Correction and Background Correction. An automatic decoy database search strategy was employed to estimate FDR (false discovery rate) using the PSPEP (Proteomics System Performance Evaluation Pipeline Software, integrated in the ProteinPilot software) (34). Only unique peptides were contained for iTRAQ labeling quantification, and peptides with a global FDR value of <1% were considered suitable for further analysis. During each iTRAQ run, the differentially expressed protein was determined according to the ratio of proteins labeled and the *p*-value provided by Protein Pilot. *P*-values were generated by Protein Pilot using a peptide used to quantitate the corresponding protein. For DEP measurements, fold change was calculated as an average comparison pairs among biological replicates, and proteins with a fold change >1.5 and *p* < 0.05 were considered significantly differentially expressed.

### Bioinformatics and Annotations

To determine the biological and functional properties of all the identified proteins, the identified protein sequences were mapped with GO terms (http://geneontology.org/). For this, all the identified sequences were first homologous searched using the localized NCBI Protein Blast (https://blast.ncbi.nlm.nih.gov/Blast.cgi?PAGE=Proteins) against the NCBI-nr animal database (https://www.ncbi.nlm.nih.gov/taxonomy). The e-value was set to < 1 e^−5^, and the GO term matching was considered the best match for each query sequence. The GO term matching was performed with the blast2go v4.5 pipeline (35). The database of Clusters of Orthologous Groups of proteins (COG, http://www.ncbi.nlm.nih.gov/COG/) was used in the new genome gene functional annotation and genome evolution. To identify candidate biomarkers, Fisher's exact test was used to determine the relationship between GO and pathway enrichment. All other images in this paper were drawn using the R language (http://www.r-project.org/).

### Motif Analysis

For Motif-X analyses (36), the sequences of all phosphopeptides (limited to 13 amino acids) were pre-aligned with identified phosphatesite centers. In addition, uniport Canis lupus familiaris (https://www.uniprot.org/uniprot/?query=canis%20lupus%20familiaris&fil=organism%3A%22Canis+lupus+familiaris+%28Dog%29+%28Canis+familiaris%29+%5B9615%5D%22&sort=score) protein sequences were used as the background database parameter, and other parameters were set to their default.

## Results

### Overview of the Identified Phosphopeptides

**Figure 2:**
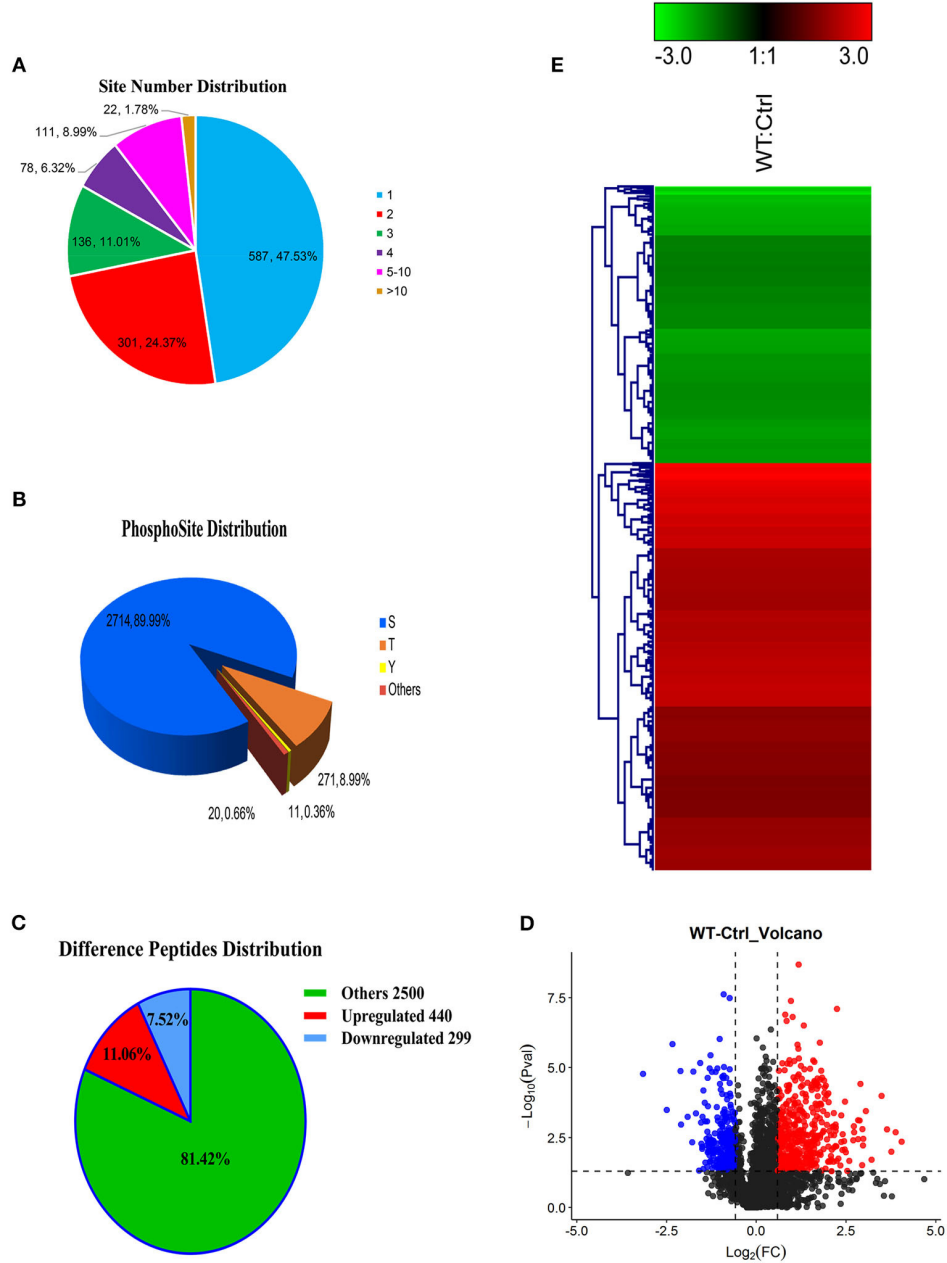
Overview of the identified proteins and phosphopeptides. **(A)** The distribution of the number of modified sites on the modified protein. One means the number of proteins with only one modification site, two means the number of proteins with only two modification sites, and so on. **(B)** Proportional distribution of modification sites S, T, Y. **(C)** Distribution of different peptides. If ratio ≥ 1.5 and *p* ≤ 0.05, its expression is considered to be significantly up-regulated; if ratio ≤ 0.67 and *p* ≤ 0.05, its expression is considered to be significantly down-regulated. **(D)** Comparison group WT: Ctrl volcano map. The red/blue dots on both sides represent the proteins with significant differences in up/down regulation. **(E)** Hierarchical clustering heat map of differentially phosphorylated peptides. The rows represent the clustering of phosphorylated peptides, and the columns represent the clustering of sample pairs. As the ratio of phosphorylated peptides changes from small to large, the color of the heat map shows a corresponding green-black-red change.

Most phosphorylated proteins contained only one phosphorylation modification site in the sequence, while some proteins were modified at multiple sites. The total phosphoproteins in the six samples of the two groups (WT and Ctrl) were explored by isobaric tags for relative and absolute quantification (iTRAQ labeling). A total of 1235 iTRAQ phosphoproteins were detected (Figure 2A). Among these, over 52% of the proteins included at least two phosphorylation sites, indicating an increase in the phosphorylation modification sites of proteins expressed in the lungs of dogs infected with CIV. In addition, 3,016 phosphorylation sites were detected, in which the numbers of phosphoserine (pS), phosphothreonine (pT), and phosphotyrosine (pY) residues were 2714 (89.99%), 271 (8.99%), and 11(0.36%), respectively (Figure 2B). To further study the different relationships of phosphoproteins in response to H3N2 CIV infection in “WT vs. Ctrl groups,” they were compared in diagrams. After CIV infection, a total of 739 phosphorylated peptides were found to be significantly different, of which 440 were significantly up-regulated (ratio ≥ 1.5, *p* ≤ 0.05), and 299 were significantly down-regulated (ratio ≤ 0.67, *p* ≤ 0.05) (Figure 2C). PTM peptide expression ratio (i.e., fold change, FC) and the *T*-test (*P*-value) were used to mark the significantly changed peptides with different colors to distinguish them, and log_2_ (FC) was used as the abscissa and negative –log_10_ (*P*-value) as the ordinate in the volcano map (Figure 2D). Clustering analysis indicated that the phosphopeptides of the WT group were clearly distinguishable from those of the Ctrl group. Among these phosphorylated peptides with significant differences, the up-regulated phosphopeptides were obviously more common than down-regulated phosphopeptides, consistent with the ratio seen for the Volcano Plot (Figure 2E). These specific CIV-responsive phosphopeptides with significant expression differences in the lungs might be crucial and valuable factors in the mechanism of interaction in CIV infections.

### Motif Analysis

**Figure 3:**
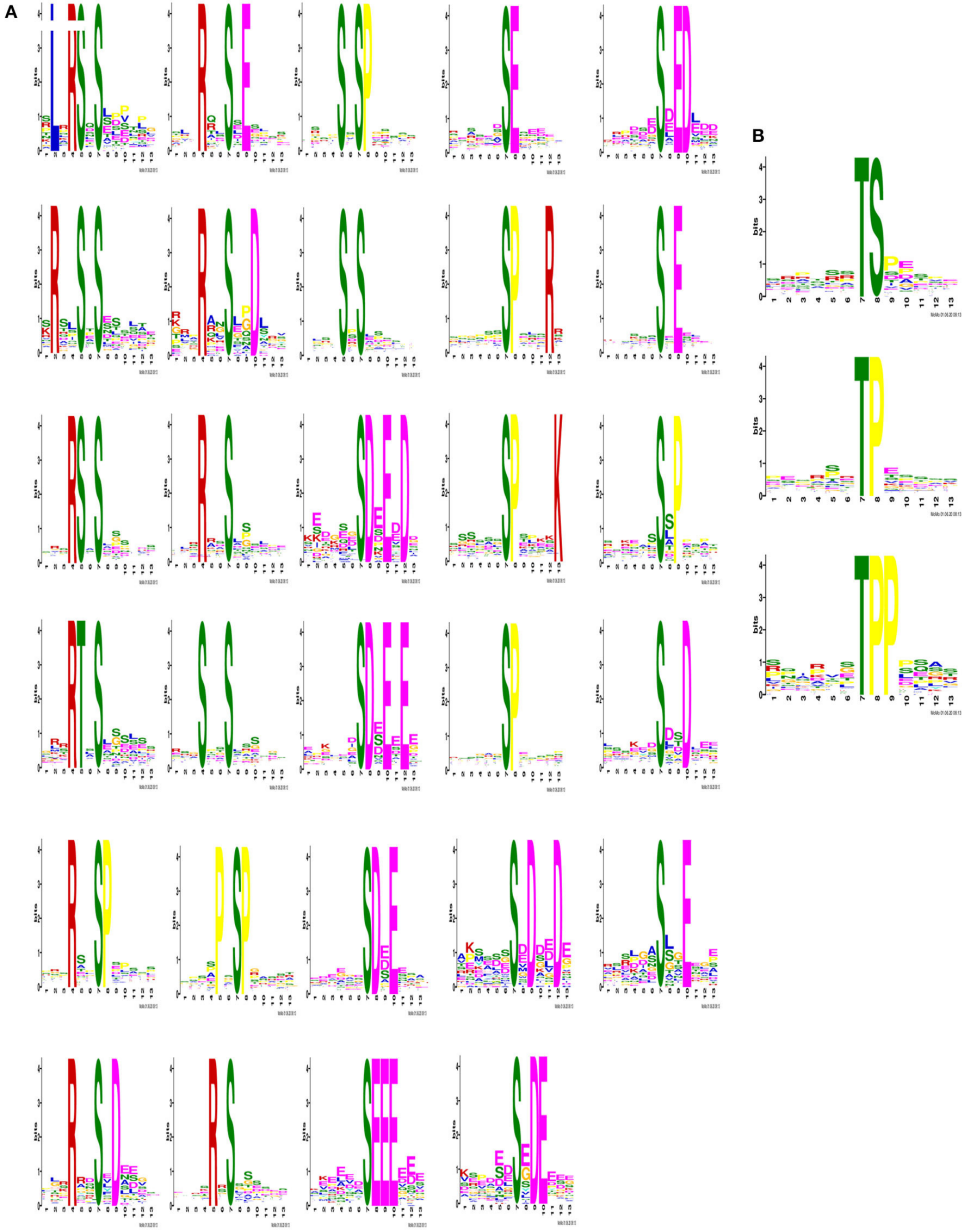
Identification of phosphosite motifs. The phosphorylation site was designated as the center to obtain a peptide sequence of 13 amino acids in total with 6 amino acids on the left and right sides. After removing the repetitions, the modified sequences centered on Ser, Thr and Tyr were analyzed for motif characteristics. When serine and threonine were used as the central modification site, the number of statistically significant motifs were calculated as 29 **(A)** and 3 **(B)**.

IMAC-enriched phosphopeptides regulated after 5 days of CIV infection were collected. The phosphorylation site was considered as the center to obtain a peptide sequence of 13 amino acids with 6 amino acids on each side using the 3,427 phosphorylated peptides identified in this study. A total of 2,995 modified sequences centered on Ser, Thr, and Tyr were analyzed by the Motif-X tool after removing duplicates. Sequence frequency graphs of significantly enriched phosphosite *de novo* motifs for centered Ser and Thr are shown (Figures 3A,B). Motif analysis showed that phosphorylation on the putative substrate peptides predominantly occurred on Ser and threonine Thr residues. However, no motifs with Tyr residues as the central modification site were identified.

During the significantly enriched phosphosite *de novo* motifs for centered Ser, The basic motif [sPxR] is related to cyclin-dependent kinase (CDK), growth-associated histone kinase (GHK), or cell division cycle 2 (CDC2) kinase (37). The motif [sP] was a proline-directed motif, which is related to CDK and mitogen-activated protein kinases (MAPKs) (38, 39). The motif [Rxxs] is a basic potential substrate for calmodulin kinase-II (CaMK-II), protein kinases A (PKA), and protein kinases C (PKC) (40). The motif [sD] is an acidic motif that can be recognized by casein kinase-II (CK-II) (40). These significantly differently expressed basic motifs in the lung characterize the mechanisms and dynamic nature of host-virus interaction after CIV infection.

### GO Analysis

We then performed an enrichment analysis of functional annotations using KEGG and GO database analysis. GO is an internationally standardized gene function classification system that provides a dynamically updated standard vocabulary (controlled vocabulary) to comprehensively describe the properties of genes and gene products in organisms. KEGG is an encyclopedia of genes and genomes and the main public database for pathway analysis (41). Pathway analysis can determine the main biochemical metabolic pathways and signal transduction pathways involved in proteins. Phosphoproteins were categorized according to their cellular components, molecular functions, and biological processes.

**Figure 4:**
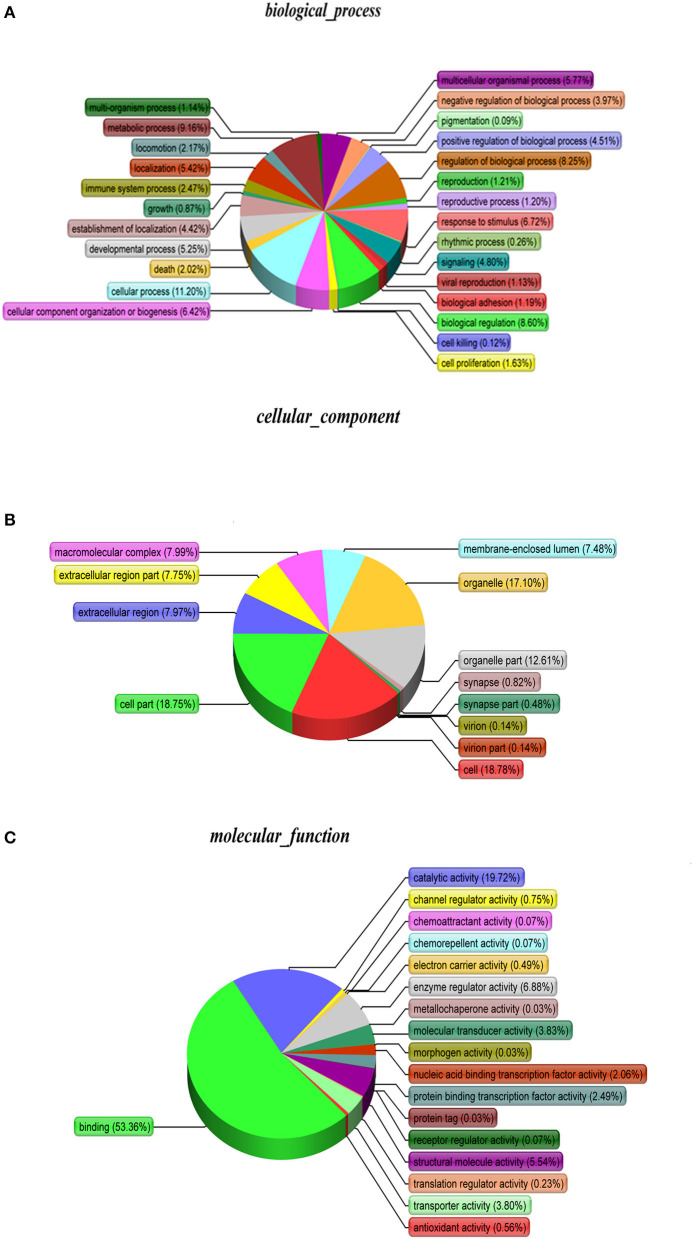
Gene Ontology (GO) classification. The GO classification diagram shows the distribution of the items involved in the three ontologies, and the different colors mark the items involved in the three ontologies. **(A)** Biological Processes. **(B)** Cellular Components. **(C)** Molecular Function.

**Figure 5:**
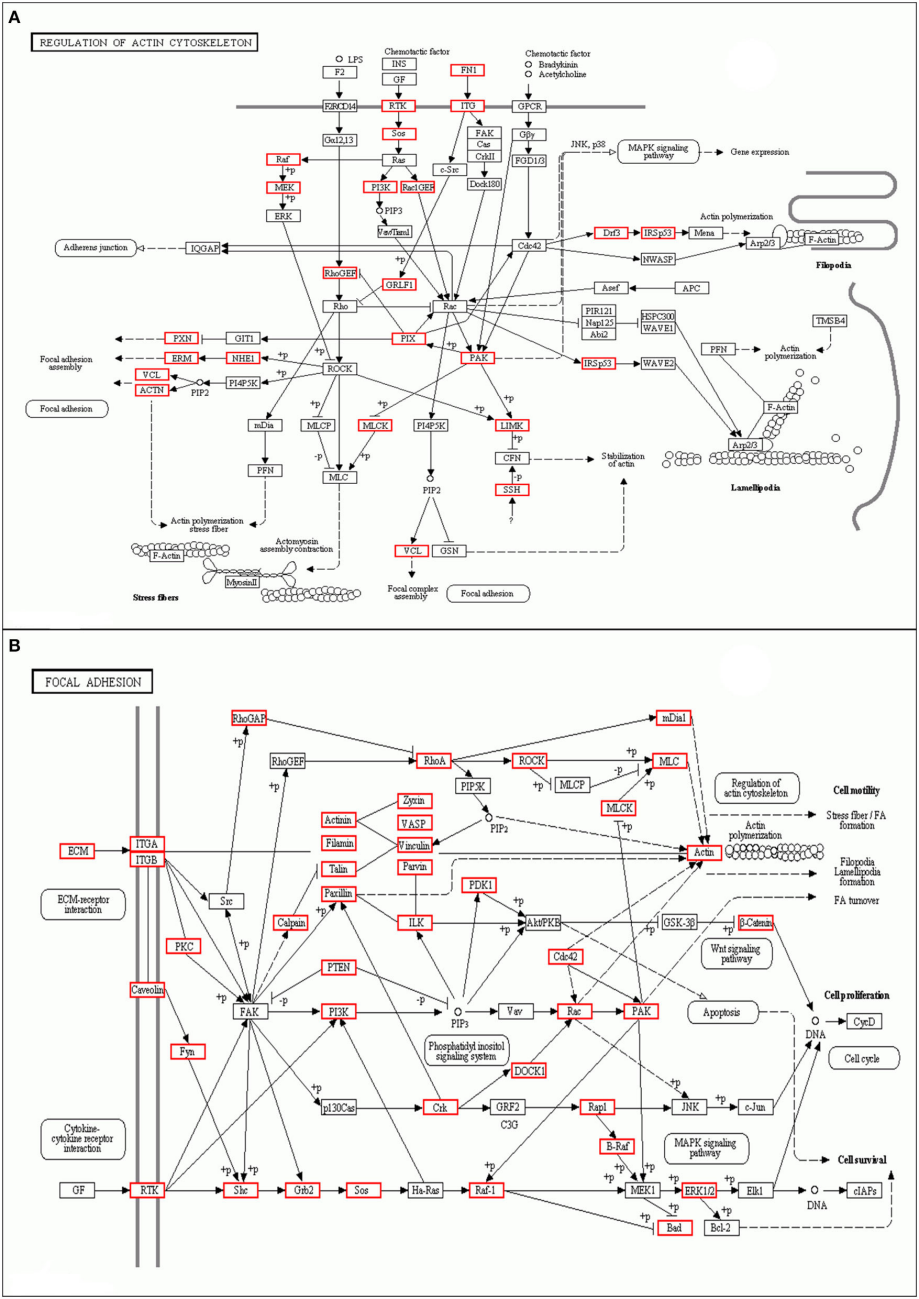
Differentially phosphorylated protein Pathway enrichment analysis. KEGG is the main public database about Pathway. Through Pathway analysis, the most important biochemical metabolic pathways and signal transduction pathways involved in protein can be determined. **(A)** Actin cytoskeleton pathway. **(B)** Focal adhesion pathway.

As shown, phosphoproteins in 56 GO categories were affected by CIV infection. The GO analysis for biological processes highlighted several processes, including metabolic process, signaling, as well as the localization (Figure 4A). The GO analysis for cellular components showed enrichment for annotations related to many different subcellular structures, including organelles, membrane-enclosed lumen, and the extracellular region (Figure 4B), which is closely related to the viral life cycle of reproduction in cells. The GO analysis for molecular function showed the involvement of diverse functions, including signaling events, structural molecular activity, and enzyme regulators, as well as chemoattractant (Figure 4C). Apparently, the molecular functions of phosphoproteins were affected greatly by CIV infection. In organisms, different proteins coordinate with each other to exercise their biological behaviors, and analysis based on pathway can improve our understanding of their biological function. The overrepresentation of phosphorylated proteins in the actin cytoskeleton pathway (Figure 5A) and focal adhesion pathway (Figure 5B) is shown by direct visualization of the phosphorylated proteins in the KEGG pathways.

### COG Analysis

**Figure 6:**
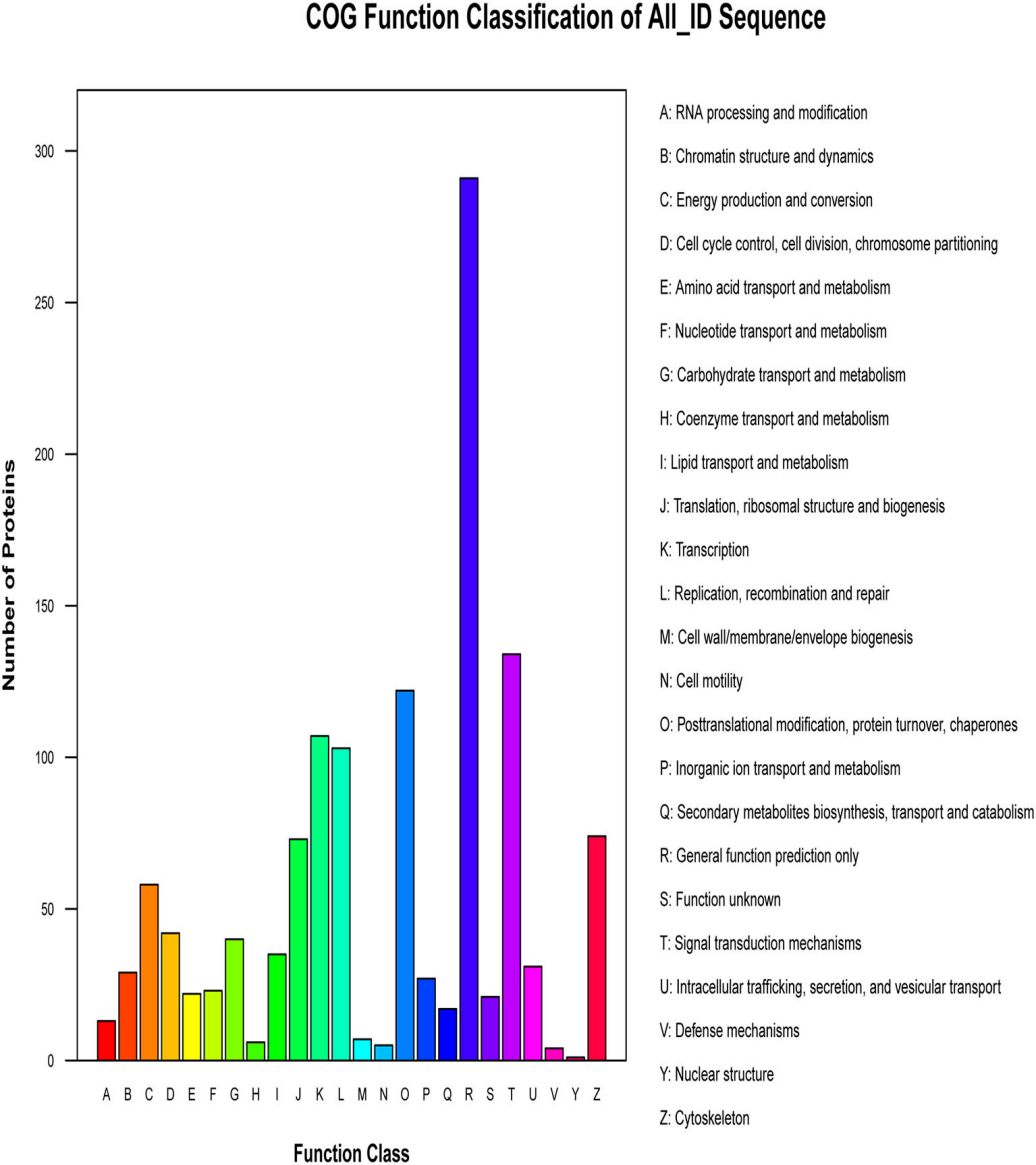
Cluster of Orthologous Groups of proteins (COG) classification. The abscissa of the figure is the COG entry, and the ordinate is the number of proteins. The figure shows the statistical number of proteins with different functions in the sample.

The proteins that make up each COG (Cluster of Orthologous Groups of proteins) are assumed to be derived from an ancestral protein, making the COG a database for orthologous classification of proteins. In different functional classes, the amount of protein reflects the content of metabolism or the physiological bias in the corresponding period and environment. We compared the identified phosphorylated proteins with the COG database, predicted the possible functions of these proteins, and used functional statistics to process classification statistics. The COG analysis showed that most of the enrichment proteins were related to general function, followed by those related to signal transduction mechanisms and PTMs (Figure 6). The results of COG analysis showed that the phosphorylated proteins expressed in the lungs after CIV infection mainly affected the general function, signal transduction mechanisms, and other functions.

### Phosphorylated Peptide Precursor Proteins GO Enrichment Analysis

**Figure 7:**
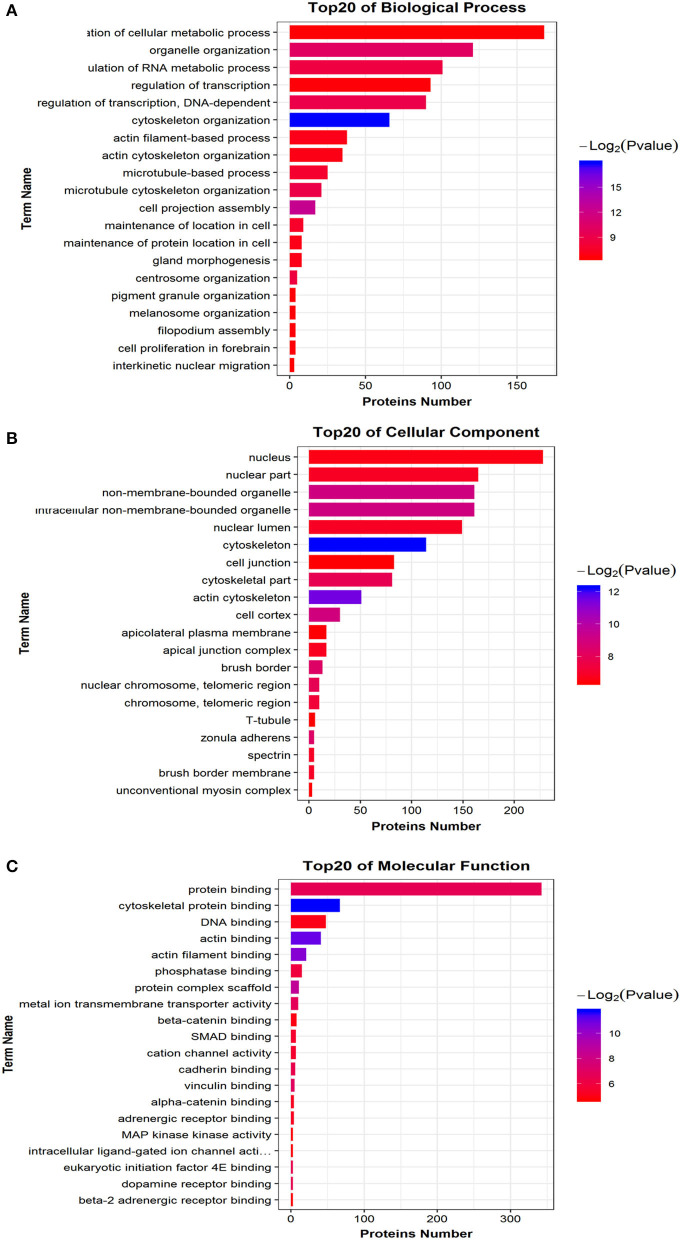
Differential phosphorylated peptide precursor protein GO enrichment analysis Histogram. **(A)** Biological Process. **(B)** Cellular Component. **(C)** Molecular Function. The abscissa EnrichFactor is the number of differential proteins annotated to this entry and the total number of proteins annotated to this entry.

We mapped all differentially phosphorylated peptide precursor proteins to each term of the Gene Ontology database (http://www.geneontology.org/), counted the number of proteins in each term, then identified the GO entries that are significantly enriched in the precursor proteins of differentially phosphorylated peptides compared to all protein backgrounds by the Fisher's standard test. When the difference in the abundance value of the differential phosphorylated peptides reached 1.5 times or more, and the *p* ≤ 0.05 after statistical testing, the peptide is regarded as the differentially phosphorylated between different samples. Comparison group WT: Ctrl differential phosphorylated peptide precursor protein GO functional enrichment analysis three ontologies (Biological Process, Cellular Component, Molecular Function). Bar chart of the top 20 function entries with significant enrichment is shown in Figure 7. The GO analysis for biological processes of differentially phosphorylated peptide precursor proteins highlighted several processes, including regulation of cellular metabolic process, organelle organization, and regulation of RNA metabolic process (Figure 7A). The GO analysis for cellular components of differentially phosphorylated peptide precursor proteins showed enrichment for annotations mostly related to the nucleus, nuclear part, and non-membrane-bounded organelle, as well as intracellular non-membrane-bounded organelle (Figure 7B). Moreover, the GO analysis of differentially phosphorylated peptide precursor proteins for molecular function showed the involvement of diverse functions, including protein binding, cytoskeletal protein binding, and DNA binding, as well as actin binding (Figure 7C).

### Phosphorylated Peptide Precursor Protein Pathway Enrichment Analysis

**Figure 8:**
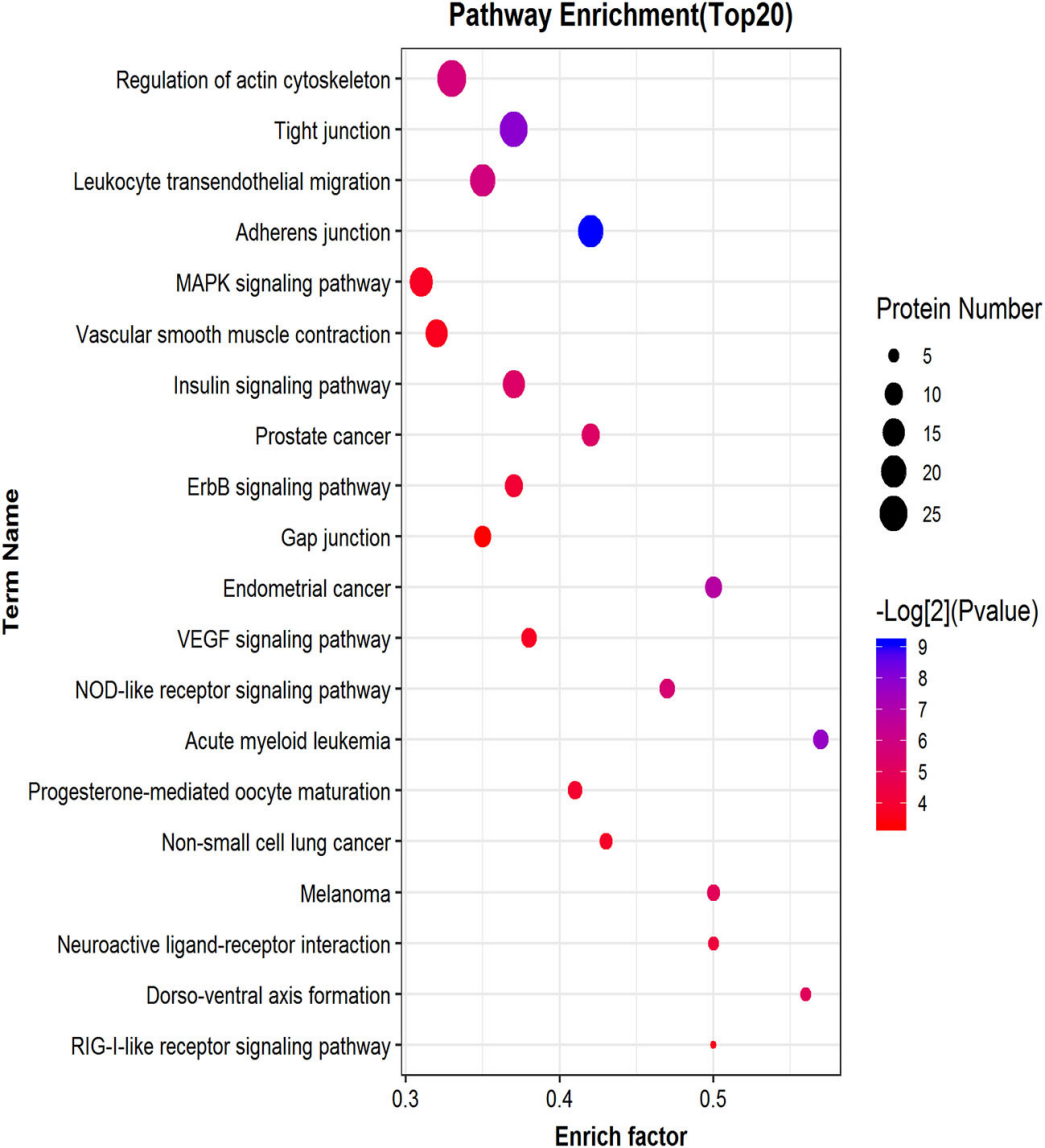
Differential phosphorylated peptide precursor protein pathway enrichment analysis bubble chart. The ordinate on the left is the name of each functional pathway, and the abscissa is the enrichment factor (the number of differential protein annotations for this functional pathway/the number of total identified protein annotations for this functional pathway).

Similar to phosphorylated proteins, we performed an enrichment pathway analysis of functional annotations using KEGG. The main biochemical metabolism pathways and signal transduction pathways involved in differential phosphorylated peptide precursor proteins could be determined by pathway enrichment. Direct visualization of the differentially phosphorylated peptide precursor protein pathway enrichment analysis is shown by the bubble chart (Figure 8). Pathway enrichment results show that the overrepresentation of differentially phosphorylated peptide precursor proteins was mainly related to regulation of the actin cytoskeleton, tight junction, and leukocyte transendothelial migration, as well as the adherens junction.

## Discussion

Virus infection and the host reaction are complex, dynamic processes. The dynamic changes in the host proteins are the result of both the viral triggers and the host's antiviral response (21). Viral infections can change the PTMs of hosts, such as phosphorylation, which viruses use to control the activities of signaling proteins (42). Phosphorylation often acts as a molecular switch dominating the signal transmission activity and downstream target proteins in signal transduction pathways (43). Our study of previous infection experiments showed that most dogs infected with H3N2 CIV started to cough at 3 dpi post-infection, with the most severely infected starting at 5 dpi, and then the symptoms gradually reduced and eventually disappeared (data not shown). Thus, in this study we collected lung samples for our phosphorylation proteomics experiments at 5 dpi. In this study, we used MS-based phosphoproteome analysis of control and infected lung samples along with functional and bioinformatics assays to identify thousands of CIV-regulated phosphorylation events in dog lungs. Phosphoproteome analysis shows major changes in host protein phosphorylation after CIV infection of dog lungs. These phosphoproteins represent several critical biological functions, including RNA processing and modification, chromatin structure and dynamics, and energy production and conversion. These dynamic adjustments of the phosphoproteome may be the direct consequence of virus infection and a series of host response events after infection.

Short sequence motifs are ubiquitous across the three major types of biomolecules, and the increased complexity of transcription, post-transcription, and post-translational regulation coincides with significant expansion of motif use (44). They are involved in multiple functions, such as recruitment of substrates to modify enzymes, subcellular localization of proteins, and PTMs (45, 46). The biochemical preference of an enzyme for a specific substrate can be directly influenced by a motif. For example, the regulatory subunit B56 of protein phosphatase 2 recognizes the LxxIxE motif (47), while the PxxP motif of the yeast protein Pbs2 can recognized by only the Sho1 SH3 domain (48). We used Motif-X (http://meme-suite.org/) to predict amino acid sequence characteristics near the identified phosphorylation sites in canine lungs triggered by CIV infection, which proved valuable for predicting the biochemical preferences of enzyme substrates.

Random encounters between protein kinases and substrate recognition motifs may result in non-functional phosphorylations, so not all phosphorylations would work (49, 50). We performed GO functional annotation analysis on all identified phosphonoproteins and classified the three involved ontologies as biological process, cellular component, and molecular function. Our data indicates that the identified phosphorylated proteins are involved in many processes of biological function, cellular component, and molecular function. COG is an orthologous protein classification database and a tool for evolutionary classification of protein families (51). By comparing the identified phosphorylated proteins of infected canine lungs in response to avian-origin CIV (H3N2) with the COG database, we predicted that these phosphoproteins are involved in 24-h biological functions, including RNA processing and modification. Interestingly, most of the differentially expressed phosphoproteins classified by COG were not directly involved in respiratory diseases or conditions, consistent with previously reported findings (21). This information supports the idea that the clinical symptoms of the infected animals may not be caused by the influenza virus but may be attributed to the host's own immune system response, such as the “cytokine storm” (52).

Some viruses promote viral entry and enhance viral protein synthesis to increase replication ability by activating the p38 MAPK and PI3K pathways or other pathways (53). In this study, we have characterized the phosphoproteome of CIV-infected dog lungs and performed bioinformatics to examine the signaling pathways activated during infection. We show that CIV infection induces major protein phosphorylation changes and activates many signaling pathways, including PI3K (Figures 5A,B) and Ras/Raf (Figures 5A,B). The PI3K pathway is an important intracellular signaling pathway used to regulate various biological processes in mammalian cells, including cell proliferation, metabolism, transformation, and motility (54). In order to improve cell survival, activation of the PI3K pathway can activate or inhibit a series of downstream target proteins, such as caspase-9, p21, Bax, and MMP2 (55, 56). Monomeric raf is a multidomain protein that is autoinhibited in the inactive state (57). The phosphorylated Ser259 of Raf-1 is recognized by 14-3-3 proteins (58, 59), which promote autoinhibition, while Ser446 phosphorylation of B-Raf attenuates autoinhibition. In our study, PI3K and Ras/Raf signaling pathways were activated under the control of the phosphorylated life processes in the lungs of dogs infected with CIV (H3N2). Although the specific signal transmission mechanism is not clear, this information is still helpful for us to understand the process of CIV infection in dogs.

In conclusion, we show that H3N2 CIV infection causes dramatic changes in the host protein phosphorylation of dog lungs. Although we have characterized the complete phosphoproteome of H3N2 CIV-infected dog lungs, full annotation was not completed. The processes underlying H3N2 CIV infection and the interaction between the virus and the host are quite complicated. It is necessary to study the downstream signaling pathways of phosphorylated proteins identified in order to gain a deeper understanding of the pathogenic mechanism of the virus and identify new therapeutic targets.

## Data Availability Statement

The datasets presented in this study can be found in online repositories. The names of the repository/repositories and accession number(s) can be found in the article/Supplementary Material.

## Ethics Statement

The animal study was reviewed and approved by South China Agricultural University Experimental Animal Welfare Ethics Committee.

## Author Contributions

YLiu contributed to the data analysis and the writing of the manuscript. CF and YLia contributed to the drafting of the manuscript. SY, ZQ, ZW, JW, CY, ST, and YC conducted the animal experiments. YLiu, SC, ZQ, and YLia contributed to the data collection and the laboratory work. SL designed the experiments and revised the paper. All authors read and approved the manuscript.

## Conflict of Interest

The authors declare that the research was conducted in the absence of any commercial or financial relationships that could be construed as a potential conflict of interest.

## Abbreviations

CIV: Canine influenza virus
IAV: Influenza A virus
EID_50_: Egg infectious dose
HI: Hemagglutination-inhibition
dpi: Days post-infection
GO: Gene ontology
KEGG: Kyoto Encyclopedia of Genes and Genomes
COG: Cluster of Orthologous Groups of proteins
PTMs: Post-translational modifications
MS: Mass spectrometry
IMAC: Immobilized metal affinity chromatography
iTRAQ: Isobaric tags for relative and absolute quantification
TEAB: Triethylammonium bicarbonate
IDA: Information Dependent Acquisition
FDR: False discovery rate
PSPEP: Proteomics System Performance Evaluation Pipeline Software
CDK: Cyclin-dependent kinase
GHK: Growth-associated histone kinase
CDC2: Cell division cycle 2
MAPKs: Mitogen-activated protein kinases
CaMK-II: Calmodulin kinase-II
PKA: Protein kinases A
PKC: Protein kinases C
CK-II: Casein kinase-II.
