# Supplementary material for: Phosphoproteomics to Characterize Host Response During H3N2 Canine Influenza Virus Infection of Dog Lung
Source: Front Vet Sci. 2020 Dec 3;7:585071. doi: 10.3389/fvets.2020.585071 (PMC7744373; doi:10.3389/fvets.2020.585071)
Supplement: Supplementary file 1 [file Data_Sheet_1.ZIP › Project_data/Enrichment/Pathway_enrichment/WT-Ctrl_Pathway_enrichment/WT-Ctrl.html]

WT-Ctrl

1. WT-Ctrl

| # | Pathway | Sample1 (349) | Sample2 (1563) | Pvalue | Pathway ID |
| 1 | Adherens junction | 20 | 48 | 0.001809739 | ko04520 |
| 2 | Tight junction | 25 | 68 | 0.004056655 | ko04530 |
| 3 | Acute myeloid leukemia | 8 | 14 | 0.004790102 | ko05221 |
| 4 | Endometrial cancer | 9 | 18 | 0.008760858 | ko05213 |
| 5 | Leukocyte transendothelial migration | 20 | 57 | 0.0173109 | ko04670 |
| 6 | Regulation of actin cytoskeleton | 27 | 83 | 0.01821724 | ko04810 |
| 7 | NOD-like receptor signaling pathway | 8 | 17 | 0.02053014 | ko04621 |
| 8 | Insulin signaling pathway | 15 | 41 | 0.02539105 | ko04910 |
| 9 | Prostate cancer | 10 | 24 | 0.02571149 | ko05215 |
| 10 | Dorso-ventral axis formation | 5 | 9 | 0.03057313 | ko04320 |
| 11 | Melanoma | 6 | 12 | 0.03205765 | ko05218 |
| 12 | Neuroactive ligand-receptor interaction | 5 | 10 | 0.05018192 | ko04080 |
| 13 | ErbB signaling pathway | 10 | 27 | 0.05831477 | ko04012 |
| 14 | Progesterone-mediated oocyte maturation | 7 | 17 | 0.06309226 | ko04914 |
| 15 | Non-small cell lung cancer | 6 | 14 | 0.06958325 | ko05223 |
| 16 | MAPK signaling pathway | 17 | 54 | 0.07347189 | ko04010 |
| 17 | VEGF signaling pathway | 8 | 21 | 0.07451536 | ko04370 |
| 18 | RIG-I-like receptor signaling pathway | 4 | 8 | 0.07964248 | ko04622 |
| 19 | Vascular smooth muscle contraction | 15 | 47 | 0.08081585 | ko04270 |
| 20 | Gap junction | 9 | 26 | 0.1037277 | ko04540 |
| 21 | Thyroid cancer | 4 | 9 | 0.1188979 | ko05216 |
| 22 | Bladder cancer | 4 | 9 | 0.1188979 | ko05219 |
| 23 | mTOR signaling pathway | 6 | 16 | 0.1246106 | ko04150 |
| 24 | Focal adhesion | 27 | 98 | 0.1247725 | ko04510 |
| 25 | Glioma | 7 | 20 | 0.136853 | ko05214 |
| 26 | Hepatitis C | 9 | 28 | 0.1515588 | ko05160 |
| 27 | Renal cell carcinoma | 6 | 17 | 0.1581661 | ko05211 |
| 28 | Pancreatic cancer | 6 | 17 | 0.1581661 | ko05212 |
| 29 | Pancreatic secretion | 8 | 25 | 0.1745823 | ko04972 |
| 30 | SNARE interactions in vesicular transport | 3 | 7 | 0.1899067 | ko04130 |
| 31 | RNA transport | 17 | 62 | 0.2018157 | ko03013 |
| 32 | Colorectal cancer | 4 | 11 | 0.2156141 | ko05210 |
| 33 | Epithelial cell signaling in Helicobacter pylori infection | 4 | 11 | 0.2156141 | ko05120 |
| 34 | Endocytosis | 19 | 71 | 0.2171568 | ko04144 |
| 35 | Glycosaminoglycan biosynthesis - keratan sulfate | 1 | 1 | 0.2232885 | ko00533 |
| 36 | Glycosphingolipid biosynthesis - lacto and neolacto series | 1 | 1 | 0.2232885 | ko00601 |
| 37 | Non-homologous end-joining | 1 | 1 | 0.2232885 | ko03450 |
| 38 | Basal cell carcinoma | 1 | 1 | 0.2232885 | ko05217 |
| 39 | Toll-like receptor signaling pathway | 5 | 15 | 0.2283558 | ko04620 |
| 40 | Pathways in cancer | 20 | 76 | 0.2339584 | ko05200 |
| 41 | Phosphatidylinositol signaling system | 8 | 27 | 0.2396055 | ko04070 |
| 42 | GnRH signaling pathway | 8 | 27 | 0.2396055 | ko04912 |
| 43 | Bacterial invasion of epithelial cells | 12 | 44 | 0.2628186 | ko05100 |
| 44 | Hypertrophic cardiomyopathy (HCM) | 10 | 36 | 0.2694461 | ko05410 |
| 45 | p53 signaling pathway | 4 | 12 | 0.2700496 | ko04115 |
| 46 | Pentose phosphate pathway | 4 | 12 | 0.2700496 | ko00030 |
| 47 | Arrhythmogenic right ventricular cardiomyopathy (ARVC) | 9 | 32 | 0.2723671 | ko05412 |
| 48 | Gastric acid secretion | 8 | 28 | 0.2748242 | ko04971 |
| 49 | Long-term depression | 5 | 16 | 0.2755127 | ko04730 |
| 50 | Long-term potentiation | 6 | 20 | 0.2770673 | ko04720 |
| 51 | Chronic myeloid leukemia | 6 | 20 | 0.2770673 | ko05220 |
| 52 | Viral myocarditis | 8 | 29 | 0.3113507 | ko05416 |
| 53 | Chemokine signaling pathway | 13 | 50 | 0.3141917 | ko04062 |
| 54 | Systemic lupus erythematosus | 7 | 25 | 0.3161995 | ko05322 |
| 55 | Wnt signaling pathway | 7 | 25 | 0.3161995 | ko04310 |
| 56 | Salivary secretion | 6 | 21 | 0.3206855 | ko04970 |
| 57 | Pyrimidine metabolism | 3 | 9 | 0.3246488 | ko00240 |
| 58 | Axon guidance | 10 | 38 | 0.3340139 | ko04360 |
| 59 | Natural killer cell mediated cytotoxicity | 6 | 22 | 0.3652 | ko04650 |
| 60 | Apoptosis | 5 | 18 | 0.374119 | ko04210 |
| 61 | Chagas disease | 4 | 14 | 0.3835225 | ko05142 |
| 62 | Glycerophospholipid metabolism | 3 | 10 | 0.3931946 | ko00564 |
| 63 | Steroid hormone biosynthesis | 1 | 2 | 0.3968304 | ko00140 |
| 64 | DNA replication | 1 | 2 | 0.3968304 | ko03030 |
| 65 | RNA polymerase | 1 | 2 | 0.3968304 | ko03020 |
| 66 | Renin-angiotensin system | 1 | 2 | 0.3968304 | ko04614 |
| 67 | Dilated cardiomyopathy | 10 | 40 | 0.401078 | ko05414 |
| 68 | N-Glycan biosynthesis | 2 | 6 | 0.40181 | ko00510 |
| 69 | Cytosolic DNA-sensing pathway | 2 | 6 | 0.40181 | ko04623 |
| 70 | MAPK signaling pathway - fly | 2 | 6 | 0.40181 | ko04013 |
| 71 | Cytokine-cytokine receptor interaction | 2 | 6 | 0.40181 | ko04060 |
| 72 | Melanogenesis | 5 | 19 | 0.4238032 | ko04916 |
| 73 | T cell receptor signaling pathway | 6 | 24 | 0.4544641 | ko04660 |
| 74 | Taste transduction | 2 | 7 | 0.4865714 | ko04742 |
| 75 | Valine, leucine and isoleucine degradation | 4 | 16 | 0.4947581 | ko00280 |
| 76 | Small cell lung cancer | 6 | 25 | 0.4981266 | ko05222 |
| 77 | Amoebiasis | 9 | 39 | 0.5184771 | ko05146 |
| 78 | Malaria | 3 | 12 | 0.5230332 | ko05144 |
| 79 | Fatty acid biosynthesis | 1 | 3 | 0.5316838 | ko00061 |
| 80 | Calcium signaling pathway | 9 | 40 | 0.5525607 | ko04020 |
| 81 | Olfactory transduction | 2 | 8 | 0.5634068 | ko04740 |
| 82 | Cardiac muscle contraction | 5 | 22 | 0.5658199 | ko04260 |
| 83 | Vibrio cholerae infection | 3 | 13 | 0.5819975 | ko05110 |
| 84 | Inositol phosphate metabolism | 4 | 18 | 0.596618 | ko00562 |
| 85 | Oocyte meiosis | 6 | 28 | 0.6201973 | ko04114 |
| 86 | Aldosterone-regulated sodium reabsorption | 2 | 9 | 0.6316234 | ko04960 |
| 87 | Type II diabetes mellitus | 2 | 9 | 0.6316234 | ko04930 |
| 88 | Drug metabolism - other enzymes | 1 | 4 | 0.6364545 | ko00983 |
| 89 | Graft-versus-host disease | 1 | 4 | 0.6364545 | ko05332 |
| 90 | B cell receptor signaling pathway | 4 | 19 | 0.642702 | ko04662 |
| 91 | Glutathione metabolism | 3 | 15 | 0.6851721 | ko00480 |
| 92 | Fc epsilon RI signaling pathway | 4 | 20 | 0.6851987 | ko04664 |
| 93 | Basal transcription factors | 1 | 5 | 0.7178383 | ko03022 |
| 94 | Nucleotide excision repair | 1 | 5 | 0.7178383 | ko03420 |
| 95 | Collecting duct acid secretion | 1 | 5 | 0.7178383 | ko04966 |
| 96 | ABC transporters | 1 | 5 | 0.7178383 | ko02010 |
| 97 | Toxoplasmosis | 5 | 26 | 0.7227672 | ko05145 |
| 98 | Antigen processing and presentation | 4 | 21 | 0.7240161 | ko04612 |
| 99 | Aminoacyl-tRNA biosynthesis | 2 | 11 | 0.7426624 | ko00970 |
| 100 | Phototransduction | 1 | 6 | 0.781044 | ko04744 |
| 101 | Type I diabetes mellitus | 1 | 6 | 0.781044 | ko04940 |
| 102 | Fructose and mannose metabolism | 1 | 6 | 0.781044 | ko00051 |
| 103 | Ubiquitin mediated proteolysis | 5 | 28 | 0.7842008 | ko04120 |
| 104 | Adipocytokine signaling pathway | 2 | 12 | 0.7865882 | ko04920 |
| 105 | Jak-STAT signaling pathway | 2 | 12 | 0.7865882 | ko04630 |
| 106 | Fc gamma R-mediated phagocytosis | 7 | 39 | 0.8025309 | ko04666 |
| 107 | Spliceosome | 13 | 69 | 0.8031525 | ko03040 |
| 108 | Autoimmune thyroid disease | 1 | 7 | 0.8301228 | ko05320 |
| 109 | Allograft rejection | 1 | 7 | 0.8301228 | ko05330 |
| 110 | Carbohydrate digestion and absorption | 1 | 7 | 0.8301228 | ko04973 |
| 111 | PPAR signaling pathway | 2 | 14 | 0.8550631 | ko03320 |
| 112 | Purine metabolism | 5 | 31 | 0.8559162 | ko00230 |
| 113 | Cell adhesion molecules (CAMs) | 5 | 31 | 0.8559162 | ko04514 |
| 114 | Alzheimer's disease | 7 | 42 | 0.8619291 | ko05010 |
| 115 | Proximal tubule bicarbonate reclamation | 1 | 8 | 0.8682251 | ko04964 |
| 116 | Pyruvate metabolism | 2 | 15 | 0.8812002 | ko00620 |
| 117 | Parkinson's disease | 5 | 33 | 0.8917667 | ko05012 |
| 118 | Protein processing in endoplasmic reticulum | 7 | 44 | 0.8927926 | ko04141 |
| 119 | Neurotrophin signaling pathway | 7 | 44 | 0.8927926 | ko04722 |
| 120 | Galactose metabolism | 1 | 9 | 0.8978003 | ko00052 |
| 121 | Notch signaling pathway | 1 | 9 | 0.8978003 | ko04330 |
| 122 | ECM-receptor interaction | 6 | 39 | 0.8988212 | ko04512 |
| 123 | Phagosome | 7 | 45 | 0.9059144 | ko04145 |
| 124 | Lysosome | 3 | 23 | 0.9162422 | ko04142 |
| 125 | Vasopressin-regulated water reabsorption | 2 | 17 | 0.9209036 | ko04962 |
| 126 | Staphylococcus aureus infection | 2 | 17 | 0.9209036 | ko05150 |
| 127 | RNA degradation | 2 | 17 | 0.9209036 | ko03018 |
| 128 | Pathogenic Escherichia coli infection | 7 | 47 | 0.9280917 | ko05130 |
| 129 | Glycolysis / Gluconeogenesis | 3 | 24 | 0.9301403 | ko00010 |
| 130 | Hematopoietic cell lineage | 2 | 18 | 0.9357168 | ko04640 |
| 131 | Complement and coagulation cascades | 2 | 18 | 0.9357168 | ko04610 |
| 132 | Propanoate metabolism | 1 | 11 | 0.9385615 | ko00640 |
| 133 | Amyotrophic lateral sclerosis (ALS) | 1 | 12 | 0.9523772 | ko05014 |
| 134 | Huntington's disease | 6 | 45 | 0.9578248 | ko05016 |
| 135 | Phototransduction - fly | 1 | 13 | 0.9630931 | ko04745 |
| 136 | TGF-beta signaling pathway | 1 | 13 | 0.9630931 | ko04350 |
| 137 | Prion diseases | 1 | 14 | 0.9714031 | ko05020 |
| 138 | Peroxisome | 1 | 15 | 0.9778462 | ko04146 |
| 139 | Cell cycle | 2 | 23 | 0.9779227 | ko04110 |
| 140 | Leishmaniasis | 1 | 16 | 0.9828408 | ko05140 |
| 141 | Oxidative phosphorylation | 1 | 18 | 0.9897116 | ko00190 |
| 142 | Shigellosis | 2 | 28 | 0.9927224 | ko05131 |
| 143 | Protein digestion and absorption | 1 | 22 | 0.9963097 | ko04974 |
| 144 | Microbial metabolism in diverse environments | 6 | 64 | 0.9983566 | ko01120 |
| 145 | Metabolic pathways | 20 | 166 | 0.9998809 | ko01100 |

| # | Pathway | Proteins |
| --- | --- | --- |
| 1 | Adherens junction | tr|E2R9S7|E2R9S7\_CANLF, tr|F1PND0|F1PND0\_CANLF, tr|E2R434|E2R434\_CANLF, tr|F1P7S2|F1P7S2\_CANLF, tr|A0A5F4D662|A0A5F4D662\_CANLF, tr|A0A5F4D0Z3|A0A5F4D0Z3\_CANLF, sp|O97758|ZO1\_CANLF, tr|F1PHJ6|F1PHJ6\_CANLF, tr|E2REU8|E2REU8\_CANLF, tr|F1PL36|F1PL36\_CANLF, tr|A0A5F4D0W8|A0A5F4D0W8\_CANLF, tr|F1PKR3|F1PKR3\_CANLF, tr|F1PF03|F1PF03\_CANLF, tr|A0A5F4DEY9|A0A5F4DEY9\_CANLF, tr|A0A5F4C280|A0A5F4C280\_CANLF, tr|E2RRM5|E2RRM5\_CANLF, tr|F6Y429|F6Y429\_CANLF, tr|F1PUA5|F1PUA5\_CANLF, tr|E2RNM8|E2RNM8\_CANLF, tr|E2R3R9|E2R3R9\_CANLF |
| 2 | Tight junction | tr|A0A5F4D6R1|A0A5F4D6R1\_CANLF, tr|F1PAP5|F1PAP5\_CANLF, tr|E2R9S7|E2R9S7\_CANLF, tr|J9P749|J9P749\_CANLF, tr|F1PVV7|F1PVV7\_CANLF, tr|F1Q2K7|F1Q2K7\_CANLF, tr|F1P7S2|F1P7S2\_CANLF, tr|A0A5F4D662|A0A5F4D662\_CANLF, tr|A0A5F4D0Z3|A0A5F4D0Z3\_CANLF, sp|O97758|ZO1\_CANLF, tr|A0A5F4CD60|A0A5F4CD60\_CANLF, tr|F1PXU1|F1PXU1\_CANLF, tr|J9P296|J9P296\_CANLF, tr|F1Q253|F1Q253\_CANLF, tr|F1PJ01|F1PJ01\_CANLF, tr|A0A5F4C280|A0A5F4C280\_CANLF, tr|E2R1P6|E2R1P6\_CANLF, tr|Q9N0W3|Q9N0W3\_CANLF, tr|A0A5F4C8U3|A0A5F4C8U3\_CANLF, tr|A0A5F4CRZ5|A0A5F4CRZ5\_CANLF, tr|A0A5F4DFZ5|A0A5F4DFZ5\_CANLF, tr|E2RRM5|E2RRM5\_CANLF, tr|E2QYC9|E2QYC9\_CANLF, tr|F6Y429|F6Y429\_CANLF, tr|F1PYS6|F1PYS6\_CANLF |
| 3 | Acute myeloid leukemia | tr|A0A5F4C5D6|A0A5F4C5D6\_CANLF, tr|Q45KI9|Q45KI9\_CANLF, tr|E2RPA3|E2RPA3\_CANLF, tr|A0A140T8E6|A0A140T8E6\_CANLF, sp|Q1HG70|MP2K2\_CANLF, tr|A0A5F4C4S3|A0A5F4C4S3\_CANLF, tr|F1P874|F1P874\_CANLF, tr|E2RRA9|E2RRA9\_CANLF |
| 4 | Endometrial cancer | tr|A0A5F4D6R1|A0A5F4D6R1\_CANLF, tr|E2R9S7|E2R9S7\_CANLF, tr|A0A5F4D0Z3|A0A5F4D0Z3\_CANLF, tr|Q45KI9|Q45KI9\_CANLF, tr|F1PF03|F1PF03\_CANLF, sp|Q1HG70|MP2K2\_CANLF, tr|A0A5F4C4S3|A0A5F4C4S3\_CANLF, tr|F1P874|F1P874\_CANLF, tr|E2RRA9|E2RRA9\_CANLF |
| 5 | Leukocyte transendothelial migration | tr|J9P5X9|J9P5X9\_CANLF, tr|E2R9S7|E2R9S7\_CANLF, tr|E2RSI6|E2RSI6\_CANLF, tr|E2R434|E2R434\_CANLF, tr|F1P7S2|F1P7S2\_CANLF, tr|A0A5F4D0Z3|A0A5F4D0Z3\_CANLF, tr|F1PUT6|F1PUT6\_CANLF, tr|E2QUL4|E2QUL4\_CANLF, tr|F1PKR3|F1PKR3\_CANLF, tr|F6UT09|F6UT09\_CANLF, tr|J9P296|J9P296\_CANLF, tr|Q9N0W3|Q9N0W3\_CANLF, tr|E2RRM5|E2RRM5\_CANLF, tr|F6V544|F6V544\_CANLF, tr|F6Y429|F6Y429\_CANLF, tr|E2RRA9|E2RRA9\_CANLF, tr|F1PCR1|F1PCR1\_CANLF, tr|E2R049|E2R049\_CANLF, tr|E2RTE0|E2RTE0\_CANLF, tr|J9P8W1|J9P8W1\_CANLF |
| 6 | Regulation of actin cytoskeleton | tr|J9P820|J9P820\_CANLF, tr|A0A5F4DF90|A0A5F4DF90\_CANLF, tr|J9P5X9|J9P5X9\_CANLF, tr|E2RSI6|E2RSI6\_CANLF, tr|F1PID1|F1PID1\_CANLF, tr|F1PVV7|F1PVV7\_CANLF, tr|F1Q2K7|F1Q2K7\_CANLF, tr|F1P7S2|F1P7S2\_CANLF, tr|E2REH3|E2REH3\_CANLF, tr|F1PUT6|F1PUT6\_CANLF, tr|E2QUL4|E2QUL4\_CANLF, tr|F1PL36|F1PL36\_CANLF, tr|E2R0Z1|E2R0Z1\_CANLF, tr|J9NVU0|J9NVU0\_CANLF, tr|F1PKR3|F1PKR3\_CANLF, tr|F1PF03|F1PF03\_CANLF, tr|E2R1Q1|E2R1Q1\_CANLF, tr|F1PJ01|F1PJ01\_CANLF, sp|Q1HG70|MP2K2\_CANLF, tr|F1PIF6|F1PIF6\_CANLF, tr|A0A5F4C4S3|A0A5F4C4S3\_CANLF, tr|F1P874|F1P874\_CANLF, tr|F6V544|F6V544\_CANLF, tr|E2RRA9|E2RRA9\_CANLF, tr|E2RTE0|E2RTE0\_CANLF, tr|A0A5F4D5S9|A0A5F4D5S9\_CANLF, tr|F1PYS6|F1PYS6\_CANLF |
| 7 | NOD-like receptor signaling pathway | tr|E2RLS3|E2RLS3\_CANLF, tr|F1PGY1|F1PGY1\_CANLF, tr|E2RQV5|E2RQV5\_CANLF, tr|A0A5F4CMN1|A0A5F4CMN1\_CANLF, tr|A0A5F4CWJ6|A0A5F4CWJ6\_CANLF, tr|F1Q433|F1Q433\_CANLF, tr|E2RI72|E2RI72\_CANLF, tr|E2R3R9|E2R3R9\_CANLF |
| 8 | Insulin signaling pathway | tr|A0A5F4D6R1|A0A5F4D6R1\_CANLF, tr|F1PND0|F1PND0\_CANLF, tr|E2QZV5|E2QZV5\_CANLF, tr|Q45KI9|Q45KI9\_CANLF, tr|A0A5F4C246|A0A5F4C246\_CANLF, tr|A0A5F4DEY9|A0A5F4DEY9\_CANLF, tr|E2RPA3|E2RPA3\_CANLF, tr|A0A5F4CNS5|A0A5F4CNS5\_CANLF, tr|J9PAP3|J9PAP3\_CANLF, sp|Q1HG70|MP2K2\_CANLF, tr|A0A5F4C4S3|A0A5F4C4S3\_CANLF, tr|F1P874|F1P874\_CANLF, tr|E2RRA9|E2RRA9\_CANLF, tr|J9P1N8|J9P1N8\_CANLF, tr|J9NS46|J9NS46\_CANLF |
| 9 | Prostate cancer | tr|A0A5F4D6R1|A0A5F4D6R1\_CANLF, tr|E2RLS3|E2RLS3\_CANLF, tr|A0A5F4D0Z3|A0A5F4D0Z3\_CANLF, tr|F1PGY1|F1PGY1\_CANLF, tr|Q45KI9|Q45KI9\_CANLF, tr|F1PF03|F1PF03\_CANLF, sp|Q1HG70|MP2K2\_CANLF, tr|A0A5F4C4S3|A0A5F4C4S3\_CANLF, tr|F1P874|F1P874\_CANLF, tr|E2RRA9|E2RRA9\_CANLF |
| 10 | Dorso-ventral axis formation | tr|J9P3Q9|J9P3Q9\_CANLF, tr|E2RSQ3|E2RSQ3\_CANLF, tr|F1PF03|F1PF03\_CANLF, tr|A0A5F4C4S3|A0A5F4C4S3\_CANLF, tr|A0A5F4CNN3|A0A5F4CNN3\_CANLF |
| 11 | Melanoma | tr|A0A5F4D6R1|A0A5F4D6R1\_CANLF, tr|Q45KI9|Q45KI9\_CANLF, tr|F1PF03|F1PF03\_CANLF, sp|Q1HG70|MP2K2\_CANLF, tr|F1P874|F1P874\_CANLF, tr|E2RRA9|E2RRA9\_CANLF |
| 12 | Neuroactive ligand-receptor interaction | tr|A0A5F4D662|A0A5F4D662\_CANLF, tr|F1P7Q6|F1P7Q6\_CANLF, tr|A0A5F4C280|A0A5F4C280\_CANLF, tr|F1PI78|F1PI78\_CANLF, tr|F1Q298|F1Q298\_CANLF |
| 13 | ErbB signaling pathway | tr|A0A5F4DF90|A0A5F4DF90\_CANLF, tr|E2REH3|E2REH3\_CANLF, tr|Q45KI9|Q45KI9\_CANLF, tr|F1PF03|F1PF03\_CANLF, tr|E2RPA3|E2RPA3\_CANLF, sp|Q1HG70|MP2K2\_CANLF, tr|A0A5F4CJ79|A0A5F4CJ79\_CANLF, tr|A0A5F4C4S3|A0A5F4C4S3\_CANLF, tr|F1P874|F1P874\_CANLF, tr|E2RRA9|E2RRA9\_CANLF |
| 14 | Progesterone-mediated oocyte maturation | tr|E2QY29|E2QY29\_CANLF, tr|E2RLS3|E2RLS3\_CANLF, tr|F1PGY1|F1PGY1\_CANLF, tr|A0A5F4CXJ9|A0A5F4CXJ9\_CANLF, tr|F1P874|F1P874\_CANLF, tr|E2RRA9|E2RRA9\_CANLF, sp|P30803|ADCY5\_CANLF |
| 15 | Non-small cell lung cancer | tr|Q45KI9|Q45KI9\_CANLF, tr|F1PF03|F1PF03\_CANLF, sp|Q1HG70|MP2K2\_CANLF, tr|A0A5F4C4S3|A0A5F4C4S3\_CANLF, tr|F1P874|F1P874\_CANLF, tr|E2RRA9|E2RRA9\_CANLF |
| 16 | MAPK signaling pathway | tr|A0A5F4DF90|A0A5F4DF90\_CANLF, tr|F1PYE3|F1PYE3\_CANLF, tr|F1PWW0|F1PWW0\_CANLF, tr|A0A5F4C0Y2|A0A5F4C0Y2\_CANLF, tr|E2REH3|E2REH3\_CANLF, tr|E2RE02|E2RE02\_CANLF, tr|J9NY42|J9NY42\_CANLF, tr|A0A5F4CXJ9|A0A5F4CXJ9\_CANLF, tr|F1PF03|F1PF03\_CANLF, sp|Q1HG70|MP2K2\_CANLF, tr|J9P5S3|J9P5S3\_CANLF, tr|A0A5F4CUX4|A0A5F4CUX4\_CANLF, tr|A0A5F4CCM0|A0A5F4CCM0\_CANLF, tr|J9NVP2|J9NVP2\_CANLF, tr|A0A5F4C4S3|A0A5F4C4S3\_CANLF, tr|F1P874|F1P874\_CANLF, tr|E2R3R9|E2R3R9\_CANLF |
| 17 | VEGF signaling pathway | tr|F1PYE3|F1PYE3\_CANLF, tr|F1PUT6|F1PUT6\_CANLF, tr|E2QUL4|E2QUL4\_CANLF, tr|Q45KI9|Q45KI9\_CANLF, sp|Q1HG70|MP2K2\_CANLF, tr|F6V544|F6V544\_CANLF, tr|E2RRA9|E2RRA9\_CANLF, tr|E2RTE0|E2RTE0\_CANLF |
| 18 | RIG-I-like receptor signaling pathway | tr|A0A5F4CDU8|A0A5F4CDU8\_CANLF, tr|E2R3V7|E2R3V7\_CANLF, tr|F1PIK5|F1PIK5\_CANLF, tr|E2R3R9|E2R3R9\_CANLF |
| 19 | Vascular smooth muscle contraction | tr|F1PVV7|F1PVV7\_CANLF, tr|F1Q2K7|F1Q2K7\_CANLF, tr|F1PBZ1|F1PBZ1\_CANLF, tr|A0A5F4BSP1|A0A5F4BSP1\_CANLF, tr|F1PW86|F1PW86\_CANLF, tr|F1PJ01|F1PJ01\_CANLF, sp|Q1HG70|MP2K2\_CANLF, tr|F1PIF6|F1PIF6\_CANLF, sp|P63091|GNAS\_CANLF, tr|A0A5F4CNW6|A0A5F4CNW6\_CANLF, tr|F1P874|F1P874\_CANLF, tr|F1PI78|F1PI78\_CANLF, tr|A0A5F4CXZ9|A0A5F4CXZ9\_CANLF, sp|P30803|ADCY5\_CANLF, tr|F1PYS6|F1PYS6\_CANLF |
| 20 | Gap junction | tr|E2QY29|E2QY29\_CANLF, sp|O97758|ZO1\_CANLF, tr|F1PF03|F1PF03\_CANLF, tr|F1PW86|F1PW86\_CANLF, sp|Q1HG70|MP2K2\_CANLF, tr|A0A5F4C4S3|A0A5F4C4S3\_CANLF, sp|P63091|GNAS\_CANLF, tr|A0A5F4CNW6|A0A5F4CNW6\_CANLF, sp|P30803|ADCY5\_CANLF |
| 21 | Thyroid cancer | tr|A0A5F4D0Z3|A0A5F4D0Z3\_CANLF, sp|Q1HG70|MP2K2\_CANLF, tr|E2RQX1|E2RQX1\_CANLF, tr|F1P874|F1P874\_CANLF |
| 22 | Bladder cancer | tr|F6X9Y6|F6X9Y6\_CANLF, tr|F1PF03|F1PF03\_CANLF, sp|Q1HG70|MP2K2\_CANLF, tr|F1P874|F1P874\_CANLF |
| 23 | mTOR signaling pathway | tr|A0A5F4CXJ9|A0A5F4CXJ9\_CANLF, tr|E2RPA3|E2RPA3\_CANLF, tr|A0A5F4CNS5|A0A5F4CNS5\_CANLF, tr|F1P874|F1P874\_CANLF, tr|A0A5F4CH02|A0A5F4CH02\_CANLF, tr|E2RRA9|E2RRA9\_CANLF |
| 24 | Focal adhesion | tr|A0A5F4DF90|A0A5F4DF90\_CANLF, tr|A0A5F4D6R1|A0A5F4D6R1\_CANLF, tr|J9P5X9|J9P5X9\_CANLF, tr|J9P614|J9P614\_CANLF, sp|P33724|CAV1\_CANLF, tr|F1P7S2|F1P7S2\_CANLF, tr|A0A5F4D0Z3|A0A5F4D0Z3\_CANLF, tr|F1PWW0|F1PWW0\_CANLF, tr|E2REH3|E2REH3\_CANLF, tr|F1PUT6|F1PUT6\_CANLF, tr|F1PSC2|F1PSC2\_CANLF, tr|F6X9Y6|F6X9Y6\_CANLF, tr|E2QUL4|E2QUL4\_CANLF, tr|Q45KI9|Q45KI9\_CANLF, tr|J9NVU0|J9NVU0\_CANLF, tr|F1PKR3|F1PKR3\_CANLF, tr|F1PF03|F1PF03\_CANLF, tr|J9P5S3|J9P5S3\_CANLF, tr|F1PIF6|F1PIF6\_CANLF, tr|A0A5F4C4S3|A0A5F4C4S3\_CANLF, sp|O46550|CAV2\_CANLF, tr|F1Q133|F1Q133\_CANLF, tr|F1P874|F1P874\_CANLF, tr|F6V544|F6V544\_CANLF, tr|E2RRA9|E2RRA9\_CANLF, tr|E2RTE0|E2RTE0\_CANLF, tr|A0A5F4D5S9|A0A5F4D5S9\_CANLF |
| 25 | Glioma | tr|A0A5F4D6R1|A0A5F4D6R1\_CANLF, tr|F1PF03|F1PF03\_CANLF, sp|Q1HG70|MP2K2\_CANLF, tr|A0A5F4CJ79|A0A5F4CJ79\_CANLF, tr|A0A5F4C4S3|A0A5F4C4S3\_CANLF, tr|F1P874|F1P874\_CANLF, tr|E2RRA9|E2RRA9\_CANLF |
| 26 | Hepatitis C | tr|A0A5F4CDU8|A0A5F4CDU8\_CANLF, tr|E2R3V7|E2R3V7\_CANLF, tr|Q45KI9|Q45KI9\_CANLF, tr|J9P296|J9P296\_CANLF, tr|F1PF03|F1PF03\_CANLF, tr|Q9N0W3|Q9N0W3\_CANLF, tr|A0A5F4C4S3|A0A5F4C4S3\_CANLF, tr|F1P874|F1P874\_CANLF, tr|E2RRA9|E2RRA9\_CANLF |
| 27 | Renal cell carcinoma | tr|A0A5F4DF90|A0A5F4DF90\_CANLF, tr|E2REH3|E2REH3\_CANLF, sp|Q1HG70|MP2K2\_CANLF, tr|A0A5F4C4S3|A0A5F4C4S3\_CANLF, tr|F1P874|F1P874\_CANLF, tr|E2RRA9|E2RRA9\_CANLF |
| 28 | Pancreatic cancer | tr|J9P5X9|J9P5X9\_CANLF, tr|Q45KI9|Q45KI9\_CANLF, tr|E2R0Z1|E2R0Z1\_CANLF, tr|F1PF03|F1PF03\_CANLF, tr|F1P874|F1P874\_CANLF, tr|E2RRA9|E2RRA9\_CANLF |
| 29 | Pancreatic secretion | tr|A0A5F4C9C0|A0A5F4C9C0\_CANLF, tr|F1PID1|F1PID1\_CANLF, tr|F1PIB8|F1PIB8\_CANLF, tr|F1PW86|F1PW86\_CANLF, tr|A0A5F4CSQ5|A0A5F4CSQ5\_CANLF, sp|P63091|GNAS\_CANLF, tr|A0A5F4CNW6|A0A5F4CNW6\_CANLF, sp|P30803|ADCY5\_CANLF |
| 30 | SNARE interactions in vesicular transport | tr|A0A5F4C0Q0|A0A5F4C0Q0\_CANLF, tr|A0A5F4D5G0|A0A5F4D5G0\_CANLF, tr|A0A5F4DBI9|A0A5F4DBI9\_CANLF |
| 31 | RNA transport | tr|A0A5F4C4T7|A0A5F4C4T7\_CANLF, tr|J9NW32|J9NW32\_CANLF, tr|E2RCA5|E2RCA5\_CANLF, tr|J9P357|J9P357\_CANLF, tr|E2RFM7|E2RFM7\_CANLF, tr|J9P504|J9P504\_CANLF, tr|E2RPA3|E2RPA3\_CANLF, tr|E2R334|E2R334\_CANLF, tr|A0A5F4CJL3|A0A5F4CJL3\_CANLF, tr|A0A5F4D555|A0A5F4D555\_CANLF, tr|A0A5F4CH02|A0A5F4CH02\_CANLF, tr|F1PVT1|F1PVT1\_CANLF, tr|A0A5F4DIQ5|A0A5F4DIQ5\_CANLF, tr|F6V4W0|F6V4W0\_CANLF, tr|F6XRK3|F6XRK3\_CANLF, sp|P79149|PININ\_CANLF, tr|E2R4V6|E2R4V6\_CANLF |
| 32 | Colorectal cancer | tr|A0A5F4D0Z3|A0A5F4D0Z3\_CANLF, tr|Q45KI9|Q45KI9\_CANLF, tr|F1P874|F1P874\_CANLF, tr|E2RRA9|E2RRA9\_CANLF |
| 33 | Epithelial cell signaling in Helicobacter pylori infection | tr|E2REH3|E2REH3\_CANLF, sp|O97758|ZO1\_CANLF, tr|F1PF03|F1PF03\_CANLF, tr|E2RI72|E2RI72\_CANLF |
| 34 | Endocytosis | tr|A0A5F4C5D6|A0A5F4C5D6\_CANLF, sp|P33724|CAV1\_CANLF, tr|J9NW32|J9NW32\_CANLF, tr|E2RH35|E2RH35\_CANLF, tr|A0A5F4D662|A0A5F4D662\_CANLF, tr|F1PNP7|F1PNP7\_CANLF, tr|O46880|O46880\_CANLF, tr|J9NY42|J9NY42\_CANLF, tr|F1PF03|F1PF03\_CANLF, tr|A0A5F4C280|A0A5F4C280\_CANLF, sp|O46550|CAV2\_CANLF, tr|A0A5F4CK26|A0A5F4CK26\_CANLF, tr|F1PVE5|F1PVE5\_CANLF, tr|E2R8Q3|E2R8Q3\_CANLF, tr|F1PK11|F1PK11\_CANLF, tr|F6X9W7|F6X9W7\_CANLF, tr|A0A5F4BSN1|A0A5F4BSN1\_CANLF, tr|J9NZR2|J9NZR2\_CANLF, tr|E2R417|E2R417\_CANLF |
| 35 | Glycosaminoglycan biosynthesis - keratan sulfate (no map in kegg database) | tr|F1PGZ1|F1PGZ1\_CANLF |
| 36 | Glycosphingolipid biosynthesis - lacto and neolacto series | tr|F1PGZ1|F1PGZ1\_CANLF |
| 37 | Non-homologous end-joining | tr|J9P1R2|J9P1R2\_CANLF |
| 38 | Basal cell carcinoma | tr|A0A5F4D0Z3|A0A5F4D0Z3\_CANLF |
| 39 | Toll-like receptor signaling pathway | tr|A0A5F4CDU8|A0A5F4CDU8\_CANLF, tr|F1PIK5|F1PIK5\_CANLF, sp|Q1HG70|MP2K2\_CANLF, tr|E2RRA9|E2RRA9\_CANLF, tr|E2R3R9|E2R3R9\_CANLF |
| 40 | Pathways in cancer | tr|A0A5F4D6R1|A0A5F4D6R1\_CANLF, tr|J9P5X9|J9P5X9\_CANLF, tr|A0A5F4C5D6|A0A5F4C5D6\_CANLF, tr|J9P614|J9P614\_CANLF, tr|E2R9S7|E2R9S7\_CANLF, tr|E2RLS3|E2RLS3\_CANLF, tr|A0A5F4D0Z3|A0A5F4D0Z3\_CANLF, tr|F1PGY1|F1PGY1\_CANLF, tr|F1PIK5|F1PIK5\_CANLF, tr|Q45KI9|Q45KI9\_CANLF, tr|J9NVU0|J9NVU0\_CANLF, tr|F1PF03|F1PF03\_CANLF, tr|A0A140T8E6|A0A140T8E6\_CANLF, sp|Q1HG70|MP2K2\_CANLF, tr|A0A5F4C4S3|A0A5F4C4S3\_CANLF, tr|E2RQX1|E2RQX1\_CANLF, tr|F1Q133|F1Q133\_CANLF, tr|F1P874|F1P874\_CANLF, tr|E2RRA9|E2RRA9\_CANLF, tr|A0A5F4D5S9|A0A5F4D5S9\_CANLF |
| 41 | Phosphatidylinositol signaling system | tr|F1PWW8|F1PWW8\_CANLF, tr|A0A5F4D6R1|A0A5F4D6R1\_CANLF, tr|A0A5F4D8L2|A0A5F4D8L2\_CANLF, tr|F1PW86|F1PW86\_CANLF, tr|E2R185|E2R185\_CANLF, tr|A0A5F4CNW6|A0A5F4CNW6\_CANLF, tr|E2RRA9|E2RRA9\_CANLF, tr|A0A5F4BQ52|A0A5F4BQ52\_CANLF |
| 42 | GnRH signaling pathway | tr|F1PF03|F1PF03\_CANLF, tr|F1PW86|F1PW86\_CANLF, sp|Q1HG70|MP2K2\_CANLF, tr|A0A5F4CJ79|A0A5F4CJ79\_CANLF, tr|A0A5F4C4S3|A0A5F4C4S3\_CANLF, sp|P63091|GNAS\_CANLF, tr|A0A5F4CNW6|A0A5F4CNW6\_CANLF, sp|P30803|ADCY5\_CANLF |
| 43 | Bacterial invasion of epithelial cells | tr|E2R9S7|E2R9S7\_CANLF, sp|P33724|CAV1\_CANLF, tr|A0A5F4D0Z3|A0A5F4D0Z3\_CANLF, tr|F1PUU5|F1PUU5\_CANLF, tr|F1PUT6|F1PUT6\_CANLF, tr|E2QUL4|E2QUL4\_CANLF, tr|F1PKR3|F1PKR3\_CANLF, sp|O46550|CAV2\_CANLF, tr|F6V544|F6V544\_CANLF, tr|E2RRA9|E2RRA9\_CANLF, tr|E2RTE0|E2RTE0\_CANLF, tr|A0A5F4D5S9|A0A5F4D5S9\_CANLF |
| 44 | Hypertrophic cardiomyopathy (HCM) | tr|A0A5F4C9C0|A0A5F4C9C0\_CANLF, tr|J9NSW5|J9NSW5\_CANLF, tr|F1PVV7|F1PVV7\_CANLF, tr|F1PCV3|F1PCV3\_CANLF, tr|J9NVU0|J9NVU0\_CANLF, tr|E2RDU7|E2RDU7\_CANLF, tr|F1PJ01|F1PJ01\_CANLF, tr|F1P8F3|F1P8F3\_CANLF, tr|J9P1N8|J9P1N8\_CANLF, tr|F1PYS6|F1PYS6\_CANLF |
| 45 | p53 signaling pathway | tr|A0A5F4D6R1|A0A5F4D6R1\_CANLF, tr|E2QY29|E2QY29\_CANLF, tr|F6X9Y6|F6X9Y6\_CANLF, tr|A0A5F4CNS5|A0A5F4CNS5\_CANLF |
| 46 | Pentose phosphate pathway | tr|E2QVM3|E2QVM3\_CANLF, tr|E2REJ7|E2REJ7\_CANLF, tr|F1PE09|F1PE09\_CANLF, tr|J9P7A6|J9P7A6\_CANLF |
| 47 | Arrhythmogenic right ventricular cardiomyopathy (ARVC) | tr|A0A5F4C9C0|A0A5F4C9C0\_CANLF, tr|E2R9S7|E2R9S7\_CANLF, tr|J9NSW5|J9NSW5\_CANLF, tr|F1P7S2|F1P7S2\_CANLF, tr|A0A5F4D0Z3|A0A5F4D0Z3\_CANLF, tr|J9NVU0|J9NVU0\_CANLF, tr|A0A140T8E6|A0A140T8E6\_CANLF, tr|F1P8F3|F1P8F3\_CANLF, tr|E2RHY0|E2RHY0\_CANLF |
| 48 | Gastric acid secretion | tr|E2RSI6|E2RSI6\_CANLF, tr|F1PID1|F1PID1\_CANLF, tr|F1PW86|F1PW86\_CANLF, tr|F1PIF6|F1PIF6\_CANLF, tr|A0A5F4CJ79|A0A5F4CJ79\_CANLF, sp|P63091|GNAS\_CANLF, tr|A0A5F4CNW6|A0A5F4CNW6\_CANLF, sp|P30803|ADCY5\_CANLF |
| 49 | Long-term depression | tr|F1PW86|F1PW86\_CANLF, sp|Q1HG70|MP2K2\_CANLF, sp|P63091|GNAS\_CANLF, tr|A0A5F4CNW6|A0A5F4CNW6\_CANLF, tr|F1P874|F1P874\_CANLF |
| 50 | Long-term potentiation | tr|A0A5F4CXJ9|A0A5F4CXJ9\_CANLF, tr|F1PW86|F1PW86\_CANLF, sp|Q1HG70|MP2K2\_CANLF, tr|A0A5F4CJ79|A0A5F4CJ79\_CANLF, tr|A0A5F4CNW6|A0A5F4CNW6\_CANLF, tr|F1P874|F1P874\_CANLF |
| 51 | Chronic myeloid leukemia | tr|Q45KI9|Q45KI9\_CANLF, tr|J9NYG3|J9NYG3\_CANLF, sp|Q1HG70|MP2K2\_CANLF, tr|A0A5F4C4S3|A0A5F4C4S3\_CANLF, tr|F1P874|F1P874\_CANLF, tr|E2RRA9|E2RRA9\_CANLF |
| 52 | Viral myocarditis | sp|P33724|CAV1\_CANLF, tr|F1PVV7|F1PVV7\_CANLF, tr|J9P357|J9P357\_CANLF, tr|O46880|O46880\_CANLF, tr|F1PJ01|F1PJ01\_CANLF, tr|E2R334|E2R334\_CANLF, tr|F1P8F3|F1P8F3\_CANLF, tr|F1PYS6|F1PYS6\_CANLF |
| 53 | Chemokine signaling pathway | tr|A0A5F4D662|A0A5F4D662\_CANLF, tr|E2REH3|E2REH3\_CANLF, tr|F1PUT6|F1PUT6\_CANLF, tr|J9NY42|J9NY42\_CANLF, tr|E2QUL4|E2QUL4\_CANLF, tr|A0A5F4C280|A0A5F4C280\_CANLF, tr|A0A5F4C4S3|A0A5F4C4S3\_CANLF, tr|F1P874|F1P874\_CANLF, tr|F6V544|F6V544\_CANLF, tr|F6X9W7|F6X9W7\_CANLF, tr|E2RRA9|E2RRA9\_CANLF, tr|E2RTE0|E2RTE0\_CANLF, sp|P30803|ADCY5\_CANLF |
| 54 | Systemic lupus erythematosus | tr|E2QW27|E2QW27\_CANLF, tr|J9PB22|J9PB22\_CANLF, tr|F1P7S2|F1P7S2\_CANLF, tr|F1PHT0|F1PHT0\_CANLF, tr|A0A5F4BU46|A0A5F4BU46\_CANLF, tr|E2RCZ7|E2RCZ7\_CANLF, tr|E2RDF9|E2RDF9\_CANLF |
| 55 | Wnt signaling pathway | tr|A0A5F4CFW2|A0A5F4CFW2\_CANLF, tr|A0A5F4D0Z3|A0A5F4D0Z3\_CANLF, tr|E2RDQ5|E2RDQ5\_CANLF, tr|F1PKX6|F1PKX6\_CANLF, tr|E2QVG1|E2QVG1\_CANLF, tr|A0A5F4CJ79|A0A5F4CJ79\_CANLF, tr|E2R3R9|E2R3R9\_CANLF |
| 56 | Salivary secretion | tr|F1PID1|F1PID1\_CANLF, tr|F1PW86|F1PW86\_CANLF, tr|A0A5F4CSQ5|A0A5F4CSQ5\_CANLF, sp|P63091|GNAS\_CANLF, tr|A0A5F4CNW6|A0A5F4CNW6\_CANLF, sp|P30803|ADCY5\_CANLF |
| 57 | Pyrimidine metabolism | tr|E2RD29|E2RD29\_CANLF, tr|A0A5F4D0K7|A0A5F4D0K7\_CANLF, tr|A0A5F4DIU7|A0A5F4DIU7\_CANLF |
| 58 | Axon guidance | tr|A0A5F4DF90|A0A5F4DF90\_CANLF, tr|F1PWQ6|F1PWQ6\_CANLF, tr|E2QY27|E2QY27\_CANLF, tr|F1Q2K7|F1Q2K7\_CANLF, tr|F1P9U4|F1P9U4\_CANLF, tr|E2REH3|E2REH3\_CANLF, tr|F1PU29|F1PU29\_CANLF, tr|F6X9Y6|F6X9Y6\_CANLF, tr|F6V544|F6V544\_CANLF, tr|F1PUA5|F1PUA5\_CANLF |
| 59 | Natural killer cell mediated cytotoxicity | tr|E2REH3|E2REH3\_CANLF, tr|O46880|O46880\_CANLF, sp|Q1HG70|MP2K2\_CANLF, tr|A0A5F4C4S3|A0A5F4C4S3\_CANLF, tr|F1P874|F1P874\_CANLF, tr|E2RRA9|E2RRA9\_CANLF |
| 60 | Apoptosis | tr|E2QZV5|E2QZV5\_CANLF, tr|A0A5F4CDU8|A0A5F4CDU8\_CANLF, tr|F1PIK5|F1PIK5\_CANLF, tr|Q45KI9|Q45KI9\_CANLF, tr|E2RRA9|E2RRA9\_CANLF |
| 61 | Chagas disease | tr|F1PCV3|F1PCV3\_CANLF, tr|F1PIK5|F1PIK5\_CANLF, sp|P63091|GNAS\_CANLF, tr|E2RRA9|E2RRA9\_CANLF |
| 62 | Glycerophospholipid metabolism | tr|A0A5F4DFU1|A0A5F4DFU1\_CANLF, tr|A0A5F4D0K7|A0A5F4D0K7\_CANLF, tr|E2R185|E2R185\_CANLF |
| 63 | Steroid hormone biosynthesis | tr|A0A5F4DKQ5|A0A5F4DKQ5\_CANLF |
| 64 | DNA replication | tr|J9P937|J9P937\_CANLF |
| 65 | RNA polymerase | tr|A0A5F4D0K7|A0A5F4D0K7\_CANLF |
| 66 | Renin-angiotensin system | tr|F1PCV3|F1PCV3\_CANLF |
| 67 | Dilated cardiomyopathy | tr|A0A5F4C9C0|A0A5F4C9C0\_CANLF, tr|J9NSW5|J9NSW5\_CANLF, tr|F1PVV7|F1PVV7\_CANLF, tr|J9NVU0|J9NVU0\_CANLF, tr|E2RDU7|E2RDU7\_CANLF, tr|F1PJ01|F1PJ01\_CANLF, tr|F1P8F3|F1P8F3\_CANLF, sp|P63091|GNAS\_CANLF, sp|P30803|ADCY5\_CANLF, tr|F1PYS6|F1PYS6\_CANLF |
| 68 | N-Glycan biosynthesis | sp|E2RG47|STT3B\_CANLF, tr|F1PGZ1|F1PGZ1\_CANLF |
| 69 | Cytosolic DNA-sensing pathway | tr|A0A5F4CDU8|A0A5F4CDU8\_CANLF, tr|E2R3V7|E2R3V7\_CANLF |
| 70 | MAPK signaling pathway - fly | tr|F1PF03|F1PF03\_CANLF, tr|A0A5F4C4S3|A0A5F4C4S3\_CANLF |
| 71 | Cytokine-cytokine receptor interaction | tr|F1PF03|F1PF03\_CANLF, tr|A0A5F4CUX4|A0A5F4CUX4\_CANLF |
| 72 | Melanogenesis | tr|A0A5F4D0Z3|A0A5F4D0Z3\_CANLF, sp|Q1HG70|MP2K2\_CANLF, tr|A0A5F4CJ79|A0A5F4CJ79\_CANLF, sp|P63091|GNAS\_CANLF, sp|P30803|ADCY5\_CANLF |
| 73 | T cell receptor signaling pathway | tr|A0A5F4DF90|A0A5F4DF90\_CANLF, tr|E2REH3|E2REH3\_CANLF, sp|Q1HG70|MP2K2\_CANLF, tr|A0A5F4C4S3|A0A5F4C4S3\_CANLF, tr|E2RRA9|E2RRA9\_CANLF, tr|E2R3R9|E2R3R9\_CANLF |
| 74 | Taste transduction | sp|P63091|GNAS\_CANLF, tr|A0A5F4CNW6|A0A5F4CNW6\_CANLF |
| 75 | Valine, leucine and isoleucine degradation | tr|E2QVM3|E2QVM3\_CANLF, tr|E2REJ7|E2REJ7\_CANLF, tr|F1PE09|F1PE09\_CANLF, tr|A0A5F4CYX7|A0A5F4CYX7\_CANLF |
| 76 | Small cell lung cancer | tr|A0A5F4D6R1|A0A5F4D6R1\_CANLF, tr|J9P614|J9P614\_CANLF, tr|J9NVU0|J9NVU0\_CANLF, tr|F1Q133|F1Q133\_CANLF, tr|E2RRA9|E2RRA9\_CANLF, tr|A0A5F4D5S9|A0A5F4D5S9\_CANLF |
| 77 | Amoebiasis | tr|J9P614|J9P614\_CANLF, tr|F1P7S2|F1P7S2\_CANLF, tr|F1PYE3|F1PYE3\_CANLF, tr|F1PKR3|F1PKR3\_CANLF, tr|A0A5F4CUX4|A0A5F4CUX4\_CANLF, sp|P63091|GNAS\_CANLF, tr|F1Q133|F1Q133\_CANLF, tr|E2RRA9|E2RRA9\_CANLF, tr|A0A5F4D5S9|A0A5F4D5S9\_CANLF |
| 78 | Malaria | tr|F6X9Y6|F6X9Y6\_CANLF, tr|E2R629|E2R629\_CANLF, tr|J9NXL3|J9NXL3\_CANLF |
| 79 | Fatty acid biosynthesis | tr|A0A5F4D6R1|A0A5F4D6R1\_CANLF |
| 80 | Calcium signaling pathway | tr|A0A5F4C9C0|A0A5F4C9C0\_CANLF, tr|F1PWW8|F1PWW8\_CANLF, tr|F1PF03|F1PF03\_CANLF, tr|F1PW86|F1PW86\_CANLF, tr|F1PIF6|F1PIF6\_CANLF, tr|A0A5F4CSQ5|A0A5F4CSQ5\_CANLF, tr|A0A5F4CJ79|A0A5F4CJ79\_CANLF, sp|P63091|GNAS\_CANLF, tr|A0A5F4CNW6|A0A5F4CNW6\_CANLF |
| 81 | Olfactory transduction | tr|J9NY42|J9NY42\_CANLF, tr|A0A5F4CJ79|A0A5F4CJ79\_CANLF |
| 82 | Cardiac muscle contraction | tr|A0A5F4C9C0|A0A5F4C9C0\_CANLF, tr|F1PID1|F1PID1\_CANLF, tr|F1PVV7|F1PVV7\_CANLF, tr|F1PJ01|F1PJ01\_CANLF, tr|F1PYS6|F1PYS6\_CANLF |
| 83 | Vibrio cholerae infection | tr|J9P749|J9P749\_CANLF, sp|O97758|ZO1\_CANLF, sp|P63091|GNAS\_CANLF |
| 84 | Inositol phosphate metabolism | tr|F1PWW8|F1PWW8\_CANLF, tr|A0A5F4D6R1|A0A5F4D6R1\_CANLF, tr|A0A5F4D8L2|A0A5F4D8L2\_CANLF, tr|A0A5F4BQ52|A0A5F4BQ52\_CANLF |
| 85 | Oocyte meiosis | tr|E2QY29|E2QY29\_CANLF, tr|A0A5F4CXJ9|A0A5F4CXJ9\_CANLF, tr|F1PW86|F1PW86\_CANLF, tr|A0A5F4CJ79|A0A5F4CJ79\_CANLF, tr|A0A5F4CNW6|A0A5F4CNW6\_CANLF, sp|P30803|ADCY5\_CANLF |
| 86 | Aldosterone-regulated sodium reabsorption | tr|E2RRA9|E2RRA9\_CANLF, tr|F1Q039|F1Q039\_CANLF |
| 87 | Type II diabetes mellitus | tr|A0A5F4DKH0|A0A5F4DKH0\_CANLF, tr|E2RRA9|E2RRA9\_CANLF |
| 88 | Drug metabolism - other enzymes | tr|A0A5F4DIU7|A0A5F4DIU7\_CANLF |
| 89 | Graft-versus-host disease | tr|O46880|O46880\_CANLF |
| 90 | B cell receptor signaling pathway | tr|J9NYG3|J9NYG3\_CANLF, sp|Q1HG70|MP2K2\_CANLF, tr|A0A5F4C4S3|A0A5F4C4S3\_CANLF, tr|E2RRA9|E2RRA9\_CANLF |
| 91 | Glutathione metabolism | tr|E2QVM3|E2QVM3\_CANLF, tr|E2REJ7|E2REJ7\_CANLF, tr|F1PE09|F1PE09\_CANLF |
| 92 | Fc epsilon RI signaling pathway | tr|J9NYG3|J9NYG3\_CANLF, sp|Q1HG70|MP2K2\_CANLF, tr|A0A5F4C4S3|A0A5F4C4S3\_CANLF, tr|E2RRA9|E2RRA9\_CANLF |
| 93 | Basal transcription factors | tr|F6Y258|F6Y258\_CANLF |
| 94 | Nucleotide excision repair | tr|A0A5F4CP79|A0A5F4CP79\_CANLF |
| 95 | Collecting duct acid secretion | tr|F1PA63|F1PA63\_CANLF |
| 96 | ABC transporters | tr|E2R872|E2R872\_CANLF |
| 97 | Toxoplasmosis | tr|J9P614|J9P614\_CANLF, tr|Q45KI9|Q45KI9\_CANLF, tr|J9NVU0|J9NVU0\_CANLF, tr|E2RRA9|E2RRA9\_CANLF, tr|E2R3R9|E2R3R9\_CANLF |
| 98 | Antigen processing and presentation | tr|E2RLS3|E2RLS3\_CANLF, tr|O46880|O46880\_CANLF, tr|F1PGY1|F1PGY1\_CANLF, tr|F1P8B4|F1P8B4\_CANLF |
| 99 | Aminoacyl-tRNA biosynthesis | sp|P49822|ALBU\_CANLF, tr|F1PYM5|F1PYM5\_CANLF |
| 100 | Phototransduction | tr|J9NY42|J9NY42\_CANLF |
| 101 | Type I diabetes mellitus | tr|O46880|O46880\_CANLF |
| 102 | Fructose and mannose metabolism | tr|J9P7A6|J9P7A6\_CANLF |
| 103 | Ubiquitin mediated proteolysis | tr|A0A5F4C5D6|A0A5F4C5D6\_CANLF, tr|F1PG13|F1PG13\_CANLF, tr|E2RGH5|E2RGH5\_CANLF, tr|J9NZR2|J9NZR2\_CANLF, tr|A0A5F4C0N2|A0A5F4C0N2\_CANLF |
| 104 | Adipocytokine signaling pathway | tr|J9P1N8|J9P1N8\_CANLF, tr|E2R3S0|E2R3S0\_CANLF |
| 105 | Jak-STAT signaling pathway | tr|A0A5F4C4S3|A0A5F4C4S3\_CANLF, tr|E2RRA9|E2RRA9\_CANLF |
| 106 | Fc gamma R-mediated phagocytosis | tr|F1PZH8|F1PZH8\_CANLF, tr|E2REH3|E2REH3\_CANLF, tr|A0A5F4DDG2|A0A5F4DDG2\_CANLF, tr|J9NYG3|J9NYG3\_CANLF, tr|A0A5F4D9F1|A0A5F4D9F1\_CANLF, tr|F6V544|F6V544\_CANLF, tr|E2RRA9|E2RRA9\_CANLF |
| 107 | Spliceosome | tr|A0A5F4C4T7|A0A5F4C4T7\_CANLF, tr|F1P8K1|F1P8K1\_CANLF, tr|E2R068|E2R068\_CANLF, tr|E2RK20|E2RK20\_CANLF, tr|A0A5F4CK91|A0A5F4CK91\_CANLF, tr|E2RCH8|E2RCH8\_CANLF, tr|E2R4I0|E2R4I0\_CANLF, tr|E2RFV7|E2RFV7\_CANLF, tr|A0A5F4D764|A0A5F4D764\_CANLF, tr|A0A5F4CJL3|A0A5F4CJL3\_CANLF, tr|E2RQV7|E2RQV7\_CANLF, tr|E2RL65|E2RL65\_CANLF, tr|F1Q3G7|F1Q3G7\_CANLF |
| 108 | Autoimmune thyroid disease | tr|O46880|O46880\_CANLF |
| 109 | Allograft rejection | tr|O46880|O46880\_CANLF |
| 110 | Carbohydrate digestion and absorption | tr|E2RRA9|E2RRA9\_CANLF |
| 111 | PPAR signaling pathway | tr|F1PND0|F1PND0\_CANLF, tr|A0A5F4DEY9|A0A5F4DEY9\_CANLF |
| 112 | Purine metabolism | tr|A0A5F4CYQ8|A0A5F4CYQ8\_CANLF, tr|A0A5F4C9Q4|A0A5F4C9Q4\_CANLF, tr|A0A5F4DKH0|A0A5F4DKH0\_CANLF, tr|A0A5F4D0K7|A0A5F4D0K7\_CANLF, sp|P30803|ADCY5\_CANLF |
| 113 | Cell adhesion molecules (CAMs) | tr|O46880|O46880\_CANLF, tr|J9NVU0|J9NVU0\_CANLF, tr|J9P296|J9P296\_CANLF, tr|Q9N0W3|Q9N0W3\_CANLF, tr|E2R049|E2R049\_CANLF |
| 114 | Alzheimer's disease | tr|A0A5F4C9C0|A0A5F4C9C0\_CANLF, tr|A0A5F4C0Y2|A0A5F4C0Y2\_CANLF, tr|F1PIK5|F1PIK5\_CANLF, tr|Q45KI9|Q45KI9\_CANLF, tr|F1PW86|F1PW86\_CANLF, tr|A0A5F4DJV1|A0A5F4DJV1\_CANLF, tr|A0A5F4CNW6|A0A5F4CNW6\_CANLF |
| 115 | Proximal tubule bicarbonate reclamation | sp|Q9N2J4|AQP1\_CANLF |
| 116 | Pyruvate metabolism | tr|A0A5F4DKH0|A0A5F4DKH0\_CANLF, tr|J9P7N4|J9P7N4\_CANLF |
| 117 | Parkinson's disease | tr|A0A5F4CT03|A0A5F4CT03\_CANLF, tr|F1PEK5|F1PEK5\_CANLF, tr|A0A5F4DJV1|A0A5F4DJV1\_CANLF, tr|A0A5F4C290|A0A5F4C290\_CANLF, tr|E2RGH5|E2RGH5\_CANLF |
| 118 | Protein processing in endoplasmic reticulum | tr|E2RLS3|E2RLS3\_CANLF, tr|A0A5F4CB52|A0A5F4CB52\_CANLF, tr|F1PGY1|F1PGY1\_CANLF, tr|F1P8B4|F1P8B4\_CANLF, tr|J9NRG5|J9NRG5\_CANLF, sp|E2RG47|STT3B\_CANLF, tr|A0A5F4CP79|A0A5F4CP79\_CANLF |
| 119 | Neurotrophin signaling pathway | tr|A0A5F4CXJ9|A0A5F4CXJ9\_CANLF, tr|Q45KI9|Q45KI9\_CANLF, sp|Q1HG70|MP2K2\_CANLF, tr|A0A5F4CJ79|A0A5F4CJ79\_CANLF, tr|A0A5F4C4S3|A0A5F4C4S3\_CANLF, tr|F1P874|F1P874\_CANLF, tr|E2RRA9|E2RRA9\_CANLF |
| 120 | Galactose metabolism | tr|F1PGZ1|F1PGZ1\_CANLF |
| 121 | Notch signaling pathway | tr|F1PKX6|F1PKX6\_CANLF |
| 122 | ECM-receptor interaction | tr|J9P614|J9P614\_CANLF, tr|J9NRJ0|J9NRJ0\_CANLF, tr|F6X9Y6|F6X9Y6\_CANLF, tr|J9NVU0|J9NVU0\_CANLF, tr|F1Q133|F1Q133\_CANLF, tr|A0A5F4D5S9|A0A5F4D5S9\_CANLF |
| 123 | Phagosome | tr|E2RH71|E2RH71\_CANLF, tr|O46880|O46880\_CANLF, tr|F6X9Y6|F6X9Y6\_CANLF, tr|E2R4C1|E2R4C1\_CANLF, tr|F1P8B4|F1P8B4\_CANLF, tr|A0A5F4C0Q0|A0A5F4C0Q0\_CANLF, tr|A0A5F4DBI9|A0A5F4DBI9\_CANLF |
| 124 | Lysosome | tr|A0A5F4DIW1|A0A5F4DIW1\_CANLF, tr|E2R4C1|E2R4C1\_CANLF, tr|J9P1I0|J9P1I0\_CANLF |
| 125 | Vasopressin-regulated water reabsorption | tr|E2RH71|E2RH71\_CANLF, sp|P63091|GNAS\_CANLF |
| 126 | Staphylococcus aureus infection (no map in kegg database) | tr|A0A5F4DJY1|A0A5F4DJY1\_CANLF, tr|F1Q0N9|F1Q0N9\_CANLF |
| 127 | RNA degradation | tr|F1PB11|F1PB11\_CANLF, tr|F1PDS0|F1PDS0\_CANLF |
| 128 | Pathogenic Escherichia coli infection | tr|J9NYB2|J9NYB2\_CANLF, tr|E2RSI6|E2RSI6\_CANLF, tr|A0A5F4D0Z3|A0A5F4D0Z3\_CANLF, tr|J9P296|J9P296\_CANLF, tr|Q9N0W3|Q9N0W3\_CANLF, tr|A0A5F4DJY1|A0A5F4DJY1\_CANLF, tr|F1Q0N9|F1Q0N9\_CANLF |
| 129 | Glycolysis / Gluconeogenesis | tr|J9P7A6|J9P7A6\_CANLF, tr|A0A5F4DKH0|A0A5F4DKH0\_CANLF, tr|J9P7N4|J9P7N4\_CANLF |
| 130 | Hematopoietic cell lineage | tr|J9NVU0|J9NVU0\_CANLF, tr|A0A5F4CUX4|A0A5F4CUX4\_CANLF |
| 131 | Complement and coagulation cascades | tr|J9NRV7|J9NRV7\_CANLF, tr|E2R886|E2R886\_CANLF |
| 132 | Propanoate metabolism | tr|J9P7N4|J9P7N4\_CANLF |
| 133 | Amyotrophic lateral sclerosis (ALS) | tr|Q45KI9|Q45KI9\_CANLF |
| 134 | Huntington's disease | tr|A0A5F4CWJ6|A0A5F4CWJ6\_CANLF, tr|F1PW86|F1PW86\_CANLF, tr|A0A5F4DJV1|A0A5F4DJV1\_CANLF, tr|A0A5F4D0K7|A0A5F4D0K7\_CANLF, tr|F1Q433|F1Q433\_CANLF, tr|A0A5F4BSN1|A0A5F4BSN1\_CANLF |
| 135 | Phototransduction - fly | tr|A0A5F4CJ79|A0A5F4CJ79\_CANLF |
| 136 | TGF-beta signaling pathway | tr|F6X9Y6|F6X9Y6\_CANLF |
| 137 | Prion diseases | sp|Q1HG70|MP2K2\_CANLF |
| 138 | Peroxisome | tr|E2RCH8|E2RCH8\_CANLF |
| 139 | Cell cycle | tr|E2QY29|E2QY29\_CANLF, tr|J9P937|J9P937\_CANLF |
| 140 | Leishmaniasis | tr|E2R3R9|E2R3R9\_CANLF |
| 141 | Oxidative phosphorylation | tr|A0A5F4DJV1|A0A5F4DJV1\_CANLF |
| 142 | Shigellosis | tr|F1PKR3|F1PKR3\_CANLF, tr|E2RI72|E2RI72\_CANLF |
| 143 | Protein digestion and absorption | tr|F1Q133|F1Q133\_CANLF |
| 144 | Microbial metabolism in diverse environments (no map in kegg database) | tr|E2QVM3|E2QVM3\_CANLF, tr|E2REJ7|E2REJ7\_CANLF, tr|F1PE09|F1PE09\_CANLF, tr|J9P7A6|J9P7A6\_CANLF, tr|A0A5F4DKH0|A0A5F4DKH0\_CANLF, tr|J9P7N4|J9P7N4\_CANLF |
| 145 | Metabolic pathways (no map in kegg database) | tr|F1PWW8|F1PWW8\_CANLF, tr|A0A5F4D6R1|A0A5F4D6R1\_CANLF, tr|E2QVM3|E2QVM3\_CANLF, tr|A0A5F4D8L2|A0A5F4D8L2\_CANLF, tr|A0A5F4DFU1|A0A5F4DFU1\_CANLF, tr|E2REJ7|E2REJ7\_CANLF, tr|F1PE09|F1PE09\_CANLF, tr|E2RD29|E2RD29\_CANLF, tr|A0A5F4DJV1|A0A5F4DJV1\_CANLF, tr|A0A5F4CYX7|A0A5F4CYX7\_CANLF, tr|A0A5F4DKQ5|A0A5F4DKQ5\_CANLF, tr|J9P7A6|J9P7A6\_CANLF, tr|A0A5F4DKH0|A0A5F4DKH0\_CANLF, tr|A0A5F4D0K7|A0A5F4D0K7\_CANLF, tr|E2R185|E2R185\_CANLF, sp|E2RG47|STT3B\_CANLF, tr|F1PGZ1|F1PGZ1\_CANLF, tr|A0A5F4BQ52|A0A5F4BQ52\_CANLF, tr|A0A5F4DIU7|A0A5F4DIU7\_CANLF, tr|J9P7N4|J9P7N4\_CANLF |
