# Supplementary material for: Phosphoproteomics to Characterize Host Response During H3N2 Canine Influenza Virus Infection of Dog Lung
Source: Front Vet Sci. 2020 Dec 3;7:585071. doi: 10.3389/fvets.2020.585071 (PMC7744373; doi:10.3389/fvets.2020.585071)
Supplement: Supplementary file 1 [file Data_Sheet_1.ZIP › Project_data/Function/All_ID_Pathway/All_ID.html]

All\_ID

1. All\_ID

| # | Pathway | Count (1563) | Pathway ID |
| --- | --- | --- | --- |
| 1 | Metabolic pathways | 166 | ko01100 |
| 2 | Focal adhesion | 98 | ko04510 |
| 3 | Regulation of actin cytoskeleton | 83 | ko04810 |
| 4 | Pathways in cancer | 76 | ko05200 |
| 5 | Endocytosis | 71 | ko04144 |
| 6 | Spliceosome | 69 | ko03040 |
| 7 | Tight junction | 68 | ko04530 |
| 8 | Microbial metabolism in diverse environments | 64 | ko01120 |
| 9 | RNA transport | 62 | ko03013 |
| 10 | Leukocyte transendothelial migration | 57 | ko04670 |
| 11 | MAPK signaling pathway | 54 | ko04010 |
| 12 | Chemokine signaling pathway | 50 | ko04062 |
| 13 | Adherens junction | 48 | ko04520 |
| 14 | Vascular smooth muscle contraction | 47 | ko04270 |
| 15 | Pathogenic Escherichia coli infection | 47 | ko05130 |
| 16 | Phagosome | 45 | ko04145 |
| 17 | Huntington's disease | 45 | ko05016 |
| 18 | Bacterial invasion of epithelial cells | 44 | ko05100 |
| 19 | Protein processing in endoplasmic reticulum | 44 | ko04141 |
| 20 | Neurotrophin signaling pathway | 44 | ko04722 |
| 21 | Alzheimer's disease | 42 | ko05010 |
| 22 | Insulin signaling pathway | 41 | ko04910 |
| 23 | Calcium signaling pathway | 40 | ko04020 |
| 24 | Dilated cardiomyopathy | 40 | ko05414 |
| 25 | Amoebiasis | 39 | ko05146 |
| 26 | Fc gamma R-mediated phagocytosis | 39 | ko04666 |
| 27 | ECM-receptor interaction | 39 | ko04512 |
| 28 | Axon guidance | 38 | ko04360 |
| 29 | Hypertrophic cardiomyopathy (HCM) | 36 | ko05410 |
| 30 | Parkinson's disease | 33 | ko05012 |
| 31 | Arrhythmogenic right ventricular cardiomyopathy (ARVC) | 32 | ko05412 |
| 32 | Purine metabolism | 31 | ko00230 |
| 33 | Ribosome | 31 | ko03010 |
| 34 | Cell adhesion molecules (CAMs) | 31 | ko04514 |
| 35 | Viral myocarditis | 29 | ko05416 |
| 36 | Hepatitis C | 28 | ko05160 |
| 37 | Gastric acid secretion | 28 | ko04971 |
| 38 | Shigellosis | 28 | ko05131 |
| 39 | Oocyte meiosis | 28 | ko04114 |
| 40 | Ubiquitin mediated proteolysis | 28 | ko04120 |
| 41 | ErbB signaling pathway | 27 | ko04012 |
| 42 | Phosphatidylinositol signaling system | 27 | ko04070 |
| 43 | GnRH signaling pathway | 27 | ko04912 |
| 44 | Gap junction | 26 | ko04540 |
| 45 | Toxoplasmosis | 26 | ko05145 |
| 46 | Systemic lupus erythematosus | 25 | ko05322 |
| 47 | Wnt signaling pathway | 25 | ko04310 |
| 48 | Small cell lung cancer | 25 | ko05222 |
| 49 | Pancreatic secretion | 25 | ko04972 |
| 50 | Prostate cancer | 24 | ko05215 |
| 51 | Glycolysis / Gluconeogenesis | 24 | ko00010 |
| 52 | T cell receptor signaling pathway | 24 | ko04660 |
| 53 | Cell cycle | 23 | ko04110 |
| 54 | Lysosome | 23 | ko04142 |
| 55 | Natural killer cell mediated cytotoxicity | 22 | ko04650 |
| 56 | Protein digestion and absorption | 22 | ko04974 |
| 57 | Cardiac muscle contraction | 22 | ko04260 |
| 58 | Salivary secretion | 21 | ko04970 |
| 59 | VEGF signaling pathway | 21 | ko04370 |
| 60 | Antigen processing and presentation | 21 | ko04612 |
| 61 | Fc epsilon RI signaling pathway | 20 | ko04664 |
| 62 | Long-term potentiation | 20 | ko04720 |
| 63 | Glioma | 20 | ko05214 |
| 64 | Chronic myeloid leukemia | 20 | ko05220 |
| 65 | Melanogenesis | 19 | ko04916 |
| 66 | B cell receptor signaling pathway | 19 | ko04662 |
| 67 | Endometrial cancer | 18 | ko05213 |
| 68 | Oxidative phosphorylation | 18 | ko00190 |
| 69 | Inositol phosphate metabolism | 18 | ko00562 |
| 70 | Hematopoietic cell lineage | 18 | ko04640 |
| 71 | Apoptosis | 18 | ko04210 |
| 72 | Complement and coagulation cascades | 18 | ko04610 |
| 73 | Renal cell carcinoma | 17 | ko05211 |
| 74 | NOD-like receptor signaling pathway | 17 | ko04621 |
| 75 | Progesterone-mediated oocyte maturation | 17 | ko04914 |
| 76 | Vasopressin-regulated water reabsorption | 17 | ko04962 |
| 77 | Pancreatic cancer | 17 | ko05212 |
| 78 | Staphylococcus aureus infection | 17 | ko05150 |
| 79 | RNA degradation | 17 | ko03018 |
| 80 | Leishmaniasis | 16 | ko05140 |
| 81 | Valine, leucine and isoleucine degradation | 16 | ko00280 |
| 82 | mTOR signaling pathway | 16 | ko04150 |
| 83 | Long-term depression | 16 | ko04730 |
| 84 | Peroxisome | 15 | ko04146 |
| 85 | Toll-like receptor signaling pathway | 15 | ko04620 |
| 86 | Glutathione metabolism | 15 | ko00480 |
| 87 | Pyruvate metabolism | 15 | ko00620 |
| 88 | PPAR signaling pathway | 14 | ko03320 |
| 89 | Non-small cell lung cancer | 14 | ko05223 |
| 90 | Chagas disease | 14 | ko05142 |
| 91 | Acute myeloid leukemia | 14 | ko05221 |
| 92 | Prion diseases | 14 | ko05020 |
| 93 | Vibrio cholerae infection | 13 | ko05110 |
| 94 | Phototransduction - fly | 13 | ko04745 |
| 95 | TGF-beta signaling pathway | 13 | ko04350 |
| 96 | Melanoma | 12 | ko05218 |
| 97 | Malaria | 12 | ko05144 |
| 98 | p53 signaling pathway | 12 | ko04115 |
| 99 | Pentose phosphate pathway | 12 | ko00030 |
| 100 | Lysine degradation | 12 | ko00310 |
| 101 | Adipocytokine signaling pathway | 12 | ko04920 |
| 102 | Jak-STAT signaling pathway | 12 | ko04630 |
| 103 | Citrate cycle (TCA cycle) | 12 | ko00020 |
| 104 | Amyotrophic lateral sclerosis (ALS) | 12 | ko05014 |
| 105 | Amino sugar and nucleotide sugar metabolism | 11 | ko00520 |
| 106 | Propanoate metabolism | 11 | ko00640 |
| 107 | Colorectal cancer | 11 | ko05210 |
| 108 | Epithelial cell signaling in Helicobacter pylori infection | 11 | ko05120 |
| 109 | Aminoacyl-tRNA biosynthesis | 11 | ko00970 |
| 110 | Proteasome | 11 | ko03050 |
| 111 | Neuroactive ligand-receptor interaction | 10 | ko04080 |
| 112 | Tryptophan metabolism | 10 | ko00380 |
| 113 | Glycerophospholipid metabolism | 10 | ko00564 |
| 114 | Fatty acid metabolism | 10 | ko00071 |
| 115 | Arginine and proline metabolism | 10 | ko00330 |
| 116 | Primary immunodeficiency | 9 | ko05340 |
| 117 | Galactose metabolism | 9 | ko00052 |
| 118 | Dorso-ventral axis formation | 9 | ko04320 |
| 119 | Thyroid cancer | 9 | ko05216 |
| 120 | Aldosterone-regulated sodium reabsorption | 9 | ko04960 |
| 121 | Bladder cancer | 9 | ko05219 |
| 122 | Notch signaling pathway | 9 | ko04330 |
| 123 | Type II diabetes mellitus | 9 | ko04930 |
| 124 | Alanine, aspartate and glutamate metabolism | 9 | ko00250 |
| 125 | Pyrimidine metabolism | 9 | ko00240 |
| 126 | RIG-I-like receptor signaling pathway | 8 | ko04622 |
| 127 | beta-Alanine metabolism | 8 | ko00410 |
| 128 | Tyrosine metabolism | 8 | ko00350 |
| 129 | Butanoate metabolism | 8 | ko00650 |
| 130 | Glycerolipid metabolism | 8 | ko00561 |
| 131 | Proximal tubule bicarbonate reclamation | 8 | ko04964 |
| 132 | Olfactory transduction | 8 | ko04740 |
| 133 | Cysteine and methionine metabolism | 7 | ko00270 |
| 134 | Autoimmune thyroid disease | 7 | ko05320 |
| 135 | Allograft rejection | 7 | ko05330 |
| 136 | Drug metabolism - cytochrome P450 | 7 | ko00982 |
| 137 | Starch and sucrose metabolism | 7 | ko00500 |
| 138 | Carbohydrate digestion and absorption | 7 | ko04973 |
| 139 | Intestinal immune network for IgA production | 7 | ko04672 |
| 140 | SNARE interactions in vesicular transport | 7 | ko04130 |
| 141 | Taste transduction | 7 | ko04742 |
| 142 | N-Glycan biosynthesis | 6 | ko00510 |
| 143 | Cytosolic DNA-sensing pathway | 6 | ko04623 |
| 144 | Metabolism of xenobiotics by cytochrome P450 | 6 | ko00980 |
| 145 | Phototransduction | 6 | ko04744 |
| 146 | Riboflavin metabolism | 6 | ko00740 |
| 147 | Type I diabetes mellitus | 6 | ko04940 |
| 148 | Nicotinate and nicotinamide metabolism | 6 | ko00760 |
| 149 | Fructose and mannose metabolism | 6 | ko00051 |
| 150 | MAPK signaling pathway - fly | 6 | ko04013 |
| 151 | Cytokine-cytokine receptor interaction | 6 | ko04060 |
| 152 | Basal transcription factors | 5 | ko03022 |
| 153 | Nucleotide excision repair | 5 | ko03420 |
| 154 | Collecting duct acid secretion | 5 | ko04966 |
| 155 | ABC transporters | 5 | ko02010 |
| 156 | Glyoxylate and dicarboxylate metabolism | 5 | ko00630 |
| 157 | Asthma | 5 | ko05310 |
| 158 | Arachidonic acid metabolism | 5 | ko00590 |
| 159 | Ether lipid metabolism | 5 | ko00565 |
| 160 | Fatty acid elongation in mitochondria | 5 | ko00062 |
| 161 | Phenylalanine metabolism | 5 | ko00360 |
| 162 | Drug metabolism - other enzymes | 4 | ko00983 |
| 163 | Protein export | 4 | ko03060 |
| 164 | Glycosaminoglycan degradation | 4 | ko00531 |
| 165 | Histidine metabolism | 4 | ko00340 |
| 166 | Biosynthesis of unsaturated fatty acids | 4 | ko01040 |
| 167 | Sphingolipid metabolism | 4 | ko00600 |
| 168 | Nitrogen metabolism | 4 | ko00910 |
| 169 | Graft-versus-host disease | 4 | ko05332 |
| 170 | Valine, leucine and isoleucine biosynthesis | 4 | ko00290 |
| 171 | Fatty acid biosynthesis | 3 | ko00061 |
| 172 | Pentose and glucuronate interconversions | 3 | ko00040 |
| 173 | Base excision repair | 3 | ko03410 |
| 174 | Glycine, serine and threonine metabolism | 3 | ko00260 |
| 175 | alpha-Linolenic acid metabolism | 3 | ko00592 |
| 176 | Ascorbate and aldarate metabolism | 3 | ko00053 |
| 177 | Glycosphingolipid biosynthesis - ganglio series | 2 | ko00604 |
| 178 | Porphyrin and chlorophyll metabolism | 2 | ko00860 |
| 179 | Selenoamino acid metabolism | 2 | ko00450 |
| 180 | Hedgehog signaling pathway | 2 | ko04340 |
| 181 | D-Glutamine and D-glutamate metabolism | 2 | ko00471 |
| 182 | Retinol metabolism | 2 | ko00830 |
| 183 | Sulfur metabolism | 2 | ko00920 |
| 184 | Regulation of autophagy | 2 | ko04140 |
| 185 | Terpenoid backbone biosynthesis | 2 | ko00900 |
| 186 | Steroid hormone biosynthesis | 2 | ko00140 |
| 187 | Other glycan degradation | 2 | ko00511 |
| 188 | Phenylalanine, tyrosine and tryptophan biosynthesis | 2 | ko00400 |
| 189 | DNA replication | 2 | ko03030 |
| 190 | Synthesis and degradation of ketone bodies | 2 | ko00072 |
| 191 | RNA polymerase | 2 | ko03020 |
| 192 | One carbon pool by folate | 2 | ko00670 |
| 193 | Renin-angiotensin system | 2 | ko04614 |
| 194 | Glycosaminoglycan biosynthesis - keratan sulfate | 1 | ko00533 |
| 195 | Linoleic acid metabolism | 1 | ko00591 |
| 196 | Circadian rhythm - mammal | 1 | ko04710 |
| 197 | Taurine and hypotaurine metabolism | 1 | ko00430 |
| 198 | Primary bile acid biosynthesis | 1 | ko00120 |
| 199 | Lysine biosynthesis | 1 | ko00300 |
| 200 | Steroid biosynthesis | 1 | ko00100 |
| 201 | Sulfur relay system | 1 | ko04122 |
| 202 | Glycosphingolipid biosynthesis - globo series | 1 | ko00603 |
| 203 | Caffeine metabolism | 1 | ko00232 |
| 204 | Glycosphingolipid biosynthesis - lacto and neolacto series | 1 | ko00601 |
| 205 | Mismatch repair | 1 | ko03430 |
| 206 | Pantothenate and CoA biosynthesis | 1 | ko00770 |
| 207 | Folate biosynthesis | 1 | ko00790 |
| 208 | Thiamine metabolism | 1 | ko00730 |
| 209 | Non-homologous end-joining | 1 | ko03450 |
| 210 | Basal cell carcinoma | 1 | ko05217 |
| 211 | Butirosin and neomycin biosynthesis | 1 | ko00524 |

| # | Pathway | Proteins |
| --- | --- | --- |
| 1 | Metabolic pathways (no map in kegg database) | sp\|E2RG47\|STT3B\_CANLF, sp\|E2RQ08\|RPN1\_CANLF, sp\|O97492\|CATA\_CANLF, sp\|P05124\|KCRB\_CANLF, tr\|A0A346M2B2\|A0A346M2B2\_CANLF, tr\|A0A5F4BQ52\|A0A5F4BQ52\_CANLF, tr\|A0A5F4BTY2\|A0A5F4BTY2\_CANLF, tr\|A0A5F4BU16\|A0A5F4BU16\_CANLF, tr\|A0A5F4BUE3\|A0A5F4BUE3\_CANLF, tr\|A0A5F4BUZ8\|A0A5F4BUZ8\_CANLF, tr\|A0A5F4BVY4\|A0A5F4BVY4\_CANLF, tr\|A0A5F4BYS0\|A0A5F4BYS0\_CANLF, tr\|A0A5F4BZU6\|A0A5F4BZU6\_CANLF, tr\|A0A5F4C1A4\|A0A5F4C1A4\_CANLF, tr\|A0A5F4C483\|A0A5F4C483\_CANLF, tr\|A0A5F4C484\|A0A5F4C484\_CANLF, tr\|A0A5F4C4W3\|A0A5F4C4W3\_CANLF, tr\|A0A5F4C6G2\|A0A5F4C6G2\_CANLF, tr\|A0A5F4C8H3\|A0A5F4C8H3\_CANLF, tr\|A0A5F4CF81\|A0A5F4CF81\_CANLF, tr\|A0A5F4CHX9\|A0A5F4CHX9\_CANLF, tr\|A0A5F4CIA0\|A0A5F4CIA0\_CANLF, tr\|A0A5F4CNR7\|A0A5F4CNR7\_CANLF, tr\|A0A5F4CNU9\|A0A5F4CNU9\_CANLF, tr\|A0A5F4CSH3\|A0A5F4CSH3\_CANLF, tr\|A0A5F4CT67\|A0A5F4CT67\_CANLF, tr\|A0A5F4CVB8\|A0A5F4CVB8\_CANLF, tr\|A0A5F4CVL0\|A0A5F4CVL0\_CANLF, tr\|A0A5F4CYX7\|A0A5F4CYX7\_CANLF, tr\|A0A5F4CZB6\|A0A5F4CZB6\_CANLF, tr\|A0A5F4D0C4\|A0A5F4D0C4\_CANLF, tr\|A0A5F4D0J5\|A0A5F4D0J5\_CANLF, tr\|A0A5F4D0K7\|A0A5F4D0K7\_CANLF, tr\|A0A5F4D296\|A0A5F4D296\_CANLF, tr\|A0A5F4D491\|A0A5F4D491\_CANLF, tr\|A0A5F4D5L1\|A0A5F4D5L1\_CANLF, tr\|A0A5F4D6E6\|A0A5F4D6E6\_CANLF, tr\|A0A5F4D6M3\|A0A5F4D6M3\_CANLF, tr\|A0A5F4D6R1\|A0A5F4D6R1\_CANLF, tr\|A0A5F4D8F3\|A0A5F4D8F3\_CANLF, tr\|A0A5F4D8L2\|A0A5F4D8L2\_CANLF, tr\|A0A5F4D8P8\|A0A5F4D8P8\_CANLF, tr\|A0A5F4DCR1\|A0A5F4DCR1\_CANLF, tr\|A0A5F4DCR7\|A0A5F4DCR7\_CANLF, tr\|A0A5F4DF70\|A0A5F4DF70\_CANLF, tr\|A0A5F4DFU1\|A0A5F4DFU1\_CANLF, tr\|A0A5F4DG67\|A0A5F4DG67\_CANLF, tr\|A0A5F4DI81\|A0A5F4DI81\_CANLF, tr\|A0A5F4DIU7\|A0A5F4DIU7\_CANLF, tr\|A0A5F4DIX0\|A0A5F4DIX0\_CANLF, tr\|A0A5F4DJV1\|A0A5F4DJV1\_CANLF, tr\|A0A5F4DKH0\|A0A5F4DKH0\_CANLF, tr\|A0A5F4DKQ5\|A0A5F4DKQ5\_CANLF, tr\|B4YY02\|B4YY02\_CANLF, tr\|E2QUA8\|E2QUA8\_CANLF, tr\|E2QV08\|E2QV08\_CANLF, tr\|E2QV12\|E2QV12\_CANLF, tr\|E2QV27\|E2QV27\_CANLF, tr\|E2QV28\|E2QV28\_CANLF, tr\|E2QVM3\|E2QVM3\_CANLF, tr\|E2QXA3\|E2QXA3\_CANLF, tr\|E2QXJ5\|E2QXJ5\_CANLF, tr\|E2QY46\|E2QY46\_CANLF, tr\|E2R185\|E2R185\_CANLF, tr\|E2R1E3\|E2R1E3\_CANLF, tr\|E2R1E8\|E2R1E8\_CANLF, tr\|E2R2C3\|E2R2C3\_CANLF, tr\|E2R5U8\|E2R5U8\_CANLF, tr\|E2R8C9\|E2R8C9\_CANLF, tr\|E2R8N8\|E2R8N8\_CANLF, tr\|E2R9U0\|E2R9U0\_CANLF, tr\|E2RC20\|E2RC20\_CANLF, tr\|E2RD29\|E2RD29\_CANLF, tr\|E2RD75\|E2RD75\_CANLF, tr\|E2REJ7\|E2REJ7\_CANLF, tr\|E2RGR9\|E2RGR9\_CANLF, tr\|E2RHL0\|E2RHL0\_CANLF, tr\|E2RLA8\|E2RLA8\_CANLF, tr\|E2RNG2\|E2RNG2\_CANLF, tr\|E2RQ99\|E2RQ99\_CANLF, tr\|E2RRC9\|E2RRC9\_CANLF, tr\|E2RRI3\|E2RRI3\_CANLF, tr\|E2RRP1\|E2RRP1\_CANLF, tr\|E2RSF7\|E2RSF7\_CANLF, tr\|E2RSJ5\|E2RSJ5\_CANLF, tr\|E2RSX5\|E2RSX5\_CANLF, tr\|E2RT65\|E2RT65\_CANLF, tr\|F1P6L7\|F1P6L7\_CANLF, tr\|F1P6W6\|F1P6W6\_CANLF, tr\|F1P797\|F1P797\_CANLF, tr\|F1PAB7\|F1PAB7\_CANLF, tr\|F1PAZ6\|F1PAZ6\_CANLF, tr\|F1PBZ5\|F1PBZ5\_CANLF, tr\|F1PE09\|F1PE09\_CANLF, tr\|F1PF82\|F1PF82\_CANLF, tr\|F1PGZ1\|F1PGZ1\_CANLF, tr\|F1PIP0\|F1PIP0\_CANLF, tr\|F1PK43\|F1PK43\_CANLF, tr\|F1PKR8\|F1PKR8\_CANLF, tr\|F1PKY3\|F1PKY3\_CANLF, tr\|F1PLG2\|F1PLG2\_CANLF, tr\|F1PLL0\|F1PLL0\_CANLF, tr\|F1PLP2\|F1PLP2\_CANLF, tr\|F1PLR0\|F1PLR0\_CANLF, tr\|F1PMC5\|F1PMC5\_CANLF, tr\|F1PMQ1\|F1PMQ1\_CANLF, tr\|F1PP35\|F1PP35\_CANLF, tr\|F1PPB7\|F1PPB7\_CANLF, tr\|F1PPD7\|F1PPD7\_CANLF, tr\|F1PPF7\|F1PPF7\_CANLF, tr\|F1PPT7\|F1PPT7\_CANLF, tr\|F1PQM1\|F1PQM1\_CANLF, tr\|F1PRF8\|F1PRF8\_CANLF, tr\|F1PRH5\|F1PRH5\_CANLF, tr\|F1PTS5\|F1PTS5\_CANLF, tr\|F1PV92\|F1PV92\_CANLF, tr\|F1PVW0\|F1PVW0\_CANLF, tr\|F1PWA9\|F1PWA9\_CANLF, tr\|F1PWH9\|F1PWH9\_CANLF, tr\|F1PWW8\|F1PWW8\_CANLF, tr\|F1PXN6\|F1PXN6\_CANLF, tr\|F1PY24\|F1PY24\_CANLF, tr\|F1PY94\|F1PY94\_CANLF, tr\|F1PYB6\|F1PYB6\_CANLF, tr\|F1PYG8\|F1PYG8\_CANLF, tr\|F1PZA0\|F1PZA0\_CANLF, tr\|F1PZA1\|F1PZA1\_CANLF, tr\|F1Q0J0\|F1Q0J0\_CANLF, tr\|F1Q2H0\|F1Q2H0\_CANLF, tr\|F1Q3R2\|F1Q3R2\_CANLF, tr\|F1Q3S9\|F1Q3S9\_CANLF, tr\|F1Q3V2\|F1Q3V2\_CANLF, tr\|F1Q3W8\|F1Q3W8\_CANLF, tr\|F1Q3X6\|F1Q3X6\_CANLF, tr\|F6UQ33\|F6UQ33\_CANLF, tr\|F6XHT8\|F6XHT8\_CANLF, tr\|F6XNH4\|F6XNH4\_CANLF, tr\|F6Y290\|F6Y290\_CANLF, tr\|H9GW87\|H9GW87\_CANLF, tr\|J9JHC8\|J9JHC8\_CANLF, tr\|J9JHD4\|J9JHD4\_CANLF, tr\|J9JHK8\|J9JHK8\_CANLF, tr\|J9NSJ8\|J9NSJ8\_CANLF, tr\|J9NVT2\|J9NVT2\_CANLF, tr\|J9NZC6\|J9NZC6\_CANLF, tr\|J9P111\|J9P111\_CANLF, tr\|J9P208\|J9P208\_CANLF, tr\|J9P2C8\|J9P2C8\_CANLF, tr\|J9P2I9\|J9P2I9\_CANLF, tr\|J9P3N9\|J9P3N9\_CANLF, tr\|J9P432\|J9P432\_CANLF, tr\|J9P6E1\|J9P6E1\_CANLF, tr\|J9P6F5\|J9P6F5\_CANLF, tr\|J9P7A6\|J9P7A6\_CANLF, tr\|J9P7N1\|J9P7N1\_CANLF, tr\|J9P7N4\|J9P7N4\_CANLF, tr\|J9P7R2\|J9P7R2\_CANLF, tr\|J9P988\|J9P988\_CANLF, tr\|J9P9G0\|J9P9G0\_CANLF, tr\|J9P9J4\|J9P9J4\_CANLF, tr\|K0J6C5\|K0J6C5\_CANLF, tr\|L7N0H7\|L7N0H7\_CANLF, tr\|Q28226\|Q28226\_CANLF, tr\|Q60F99\|Q60F99\_CANLF, tr\|Q95N05\|Q95N05\_CANLF, tr\|V5LKV5\|V5LKV5\_CANLF |
| 2 | Focal adhesion | sp\|O18840\|ACTB\_CANLF, sp\|O46550\|CAV2\_CANLF, sp\|P33724\|CAV1\_CANLF, tr\|A0A5F4BVB0\|A0A5F4BVB0\_CANLF, tr\|A0A5F4C3X9\|A0A5F4C3X9\_CANLF, tr\|A0A5F4C4G4\|A0A5F4C4G4\_CANLF, tr\|A0A5F4C4S3\|A0A5F4C4S3\_CANLF, tr\|A0A5F4C566\|A0A5F4C566\_CANLF, tr\|A0A5F4C5E5\|A0A5F4C5E5\_CANLF, tr\|A0A5F4C7K2\|A0A5F4C7K2\_CANLF, tr\|A0A5F4C9R4\|A0A5F4C9R4\_CANLF, tr\|A0A5F4CA24\|A0A5F4CA24\_CANLF, tr\|A0A5F4CB93\|A0A5F4CB93\_CANLF, tr\|A0A5F4CJ74\|A0A5F4CJ74\_CANLF, tr\|A0A5F4CT67\|A0A5F4CT67\_CANLF, tr\|A0A5F4CWM0\|A0A5F4CWM0\_CANLF, tr\|A0A5F4CWX2\|A0A5F4CWX2\_CANLF, tr\|A0A5F4CZS8\|A0A5F4CZS8\_CANLF, tr\|A0A5F4D0Z3\|A0A5F4D0Z3\_CANLF, tr\|A0A5F4D5S9\|A0A5F4D5S9\_CANLF, tr\|A0A5F4D5V0\|A0A5F4D5V0\_CANLF, tr\|A0A5F4D6R1\|A0A5F4D6R1\_CANLF, tr\|A0A5F4DC86\|A0A5F4DC86\_CANLF, tr\|A0A5F4DCI2\|A0A5F4DCI2\_CANLF, tr\|A0A5F4DF90\|A0A5F4DF90\_CANLF, tr\|A0A5F4DFP9\|A0A5F4DFP9\_CANLF, tr\|A0A5F4DIK8\|A0A5F4DIK8\_CANLF, tr\|E2QUK3\|E2QUK3\_CANLF, tr\|E2QUL4\|E2QUL4\_CANLF, tr\|E2QWB3\|E2QWB3\_CANLF, tr\|E2QWD3\|E2QWD3\_CANLF, tr\|E2QXY7\|E2QXY7\_CANLF, tr\|E2QY08\|E2QY08\_CANLF, tr\|E2QY85\|E2QY85\_CANLF, tr\|E2R0D7\|E2R0D7\_CANLF, tr\|E2R2K4\|E2R2K4\_CANLF, tr\|E2R4J1\|E2R4J1\_CANLF, tr\|E2R5H4\|E2R5H4\_CANLF, tr\|E2RC09\|E2RC09\_CANLF, tr\|E2RED4\|E2RED4\_CANLF, tr\|E2REH3\|E2REH3\_CANLF, tr\|E2RF98\|E2RF98\_CANLF, tr\|E2RRA9\|E2RRA9\_CANLF, tr\|E2RT60\|E2RT60\_CANLF, tr\|E2RTE0\|E2RTE0\_CANLF, tr\|F1P634\|F1P634\_CANLF, tr\|F1P7F8\|F1P7F8\_CANLF, tr\|F1P7S2\|F1P7S2\_CANLF, tr\|F1P803\|F1P803\_CANLF, tr\|F1P874\|F1P874\_CANLF, tr\|F1P975\|F1P975\_CANLF, tr\|F1PEM6\|F1PEM6\_CANLF, tr\|F1PF03\|F1PF03\_CANLF, tr\|F1PF31\|F1PF31\_CANLF, tr\|F1PFM6\|F1PFM6\_CANLF, tr\|F1PG69\|F1PG69\_CANLF, tr\|F1PHX8\|F1PHX8\_CANLF, tr\|F1PHY1\|F1PHY1\_CANLF, tr\|F1PIF6\|F1PIF6\_CANLF, tr\|F1PKR3\|F1PKR3\_CANLF, tr\|F1PKR8\|F1PKR8\_CANLF, tr\|F1PKU7\|F1PKU7\_CANLF, tr\|F1PP33\|F1PP33\_CANLF, tr\|F1PPG5\|F1PPG5\_CANLF, tr\|F1PQ29\|F1PQ29\_CANLF, tr\|F1PQC4\|F1PQC4\_CANLF, tr\|F1PR31\|F1PR31\_CANLF, tr\|F1PR66\|F1PR66\_CANLF, tr\|F1PSC2\|F1PSC2\_CANLF, tr\|F1PUT6\|F1PUT6\_CANLF, tr\|F1PWW0\|F1PWW0\_CANLF, tr\|F1PYQ1\|F1PYQ1\_CANLF, tr\|F1Q133\|F1Q133\_CANLF, tr\|F1Q1D5\|F1Q1D5\_CANLF, tr\|F1Q2F7\|F1Q2F7\_CANLF, tr\|F1Q2Y3\|F1Q2Y3\_CANLF, tr\|F1Q3I5\|F1Q3I5\_CANLF, tr\|F1Q4I3\|F1Q4I3\_CANLF, tr\|F6V544\|F6V544\_CANLF, tr\|F6X9Y6\|F6X9Y6\_CANLF, tr\|F6XY66\|F6XY66\_CANLF, tr\|J9NUY1\|J9NUY1\_CANLF, tr\|J9NVU0\|J9NVU0\_CANLF, tr\|J9NXT9\|J9NXT9\_CANLF, tr\|J9P1X5\|J9P1X5\_CANLF, tr\|J9P4L9\|J9P4L9\_CANLF, tr\|J9P5S3\|J9P5S3\_CANLF, tr\|J9P5X9\|J9P5X9\_CANLF, tr\|J9P614\|J9P614\_CANLF, tr\|J9P889\|J9P889\_CANLF, tr\|J9P9H3\|J9P9H3\_CANLF, tr\|J9PAK3\|J9PAK3\_CANLF, tr\|Q28272\|Q28272\_CANLF, tr\|Q45KI9\|Q45KI9\_CANLF, tr\|Q597P9\|Q597P9\_CANLF, tr\|Q867A1\|Q867A1\_CANLF, tr\|Q867A2\|Q867A2\_CANLF, tr\|Z4YHI2\|Z4YHI2\_CANLF |
| 3 | Regulation of actin cytoskeleton | sp\|O18840\|ACTB\_CANLF, sp\|Q1HG70\|MP2K2\_CANLF, tr\|A0A5F4BVB0\|A0A5F4BVB0\_CANLF, tr\|A0A5F4C3X9\|A0A5F4C3X9\_CANLF, tr\|A0A5F4C4S3\|A0A5F4C4S3\_CANLF, tr\|A0A5F4C5E5\|A0A5F4C5E5\_CANLF, tr\|A0A5F4C9R4\|A0A5F4C9R4\_CANLF, tr\|A0A5F4CB93\|A0A5F4CB93\_CANLF, tr\|A0A5F4CMX3\|A0A5F4CMX3\_CANLF, tr\|A0A5F4CWM0\|A0A5F4CWM0\_CANLF, tr\|A0A5F4CWM9\|A0A5F4CWM9\_CANLF, tr\|A0A5F4CWP8\|A0A5F4CWP8\_CANLF, tr\|A0A5F4D5S9\|A0A5F4D5S9\_CANLF, tr\|A0A5F4D5V0\|A0A5F4D5V0\_CANLF, tr\|A0A5F4DC86\|A0A5F4DC86\_CANLF, tr\|A0A5F4DF75\|A0A5F4DF75\_CANLF, tr\|A0A5F4DF90\|A0A5F4DF90\_CANLF, tr\|A0A5F4DHY6\|A0A5F4DHY6\_CANLF, tr\|A0A5F4DIK8\|A0A5F4DIK8\_CANLF, tr\|A0A5F4DMB6\|A0A5F4DMB6\_CANLF, tr\|E2QUL4\|E2QUL4\_CANLF, tr\|E2QWD3\|E2QWD3\_CANLF, tr\|E2QWU0\|E2QWU0\_CANLF, tr\|E2QXY7\|E2QXY7\_CANLF, tr\|E2QY08\|E2QY08\_CANLF, tr\|E2QY85\|E2QY85\_CANLF, tr\|E2R0N1\|E2R0N1\_CANLF, tr\|E2R0Z1\|E2R0Z1\_CANLF, tr\|E2R1Q1\|E2R1Q1\_CANLF, tr\|E2REH3\|E2REH3\_CANLF, tr\|E2RF98\|E2RF98\_CANLF, tr\|E2RMT4\|E2RMT4\_CANLF, tr\|E2RRA9\|E2RRA9\_CANLF, tr\|E2RSI6\|E2RSI6\_CANLF, tr\|E2RT60\|E2RT60\_CANLF, tr\|E2RTE0\|E2RTE0\_CANLF, tr\|F1P634\|F1P634\_CANLF, tr\|F1P7S2\|F1P7S2\_CANLF, tr\|F1P803\|F1P803\_CANLF, tr\|F1P874\|F1P874\_CANLF, tr\|F1PAG6\|F1PAG6\_CANLF, tr\|F1PC86\|F1PC86\_CANLF, tr\|F1PET2\|F1PET2\_CANLF, tr\|F1PF03\|F1PF03\_CANLF, tr\|F1PH57\|F1PH57\_CANLF, tr\|F1PID1\|F1PID1\_CANLF, tr\|F1PIF6\|F1PIF6\_CANLF, tr\|F1PIV9\|F1PIV9\_CANLF, tr\|F1PJ01\|F1PJ01\_CANLF, tr\|F1PJ65\|F1PJ65\_CANLF, tr\|F1PJP7\|F1PJP7\_CANLF, tr\|F1PKR3\|F1PKR3\_CANLF, tr\|F1PL36\|F1PL36\_CANLF, tr\|F1PP33\|F1PP33\_CANLF, tr\|F1PPG5\|F1PPG5\_CANLF, tr\|F1PQ29\|F1PQ29\_CANLF, tr\|F1PUT6\|F1PUT6\_CANLF, tr\|F1PVV7\|F1PVV7\_CANLF, tr\|F1PYQ1\|F1PYQ1\_CANLF, tr\|F1PYS6\|F1PYS6\_CANLF, tr\|F1Q2K7\|F1Q2K7\_CANLF, tr\|F1Q3Y0\|F1Q3Y0\_CANLF, tr\|F1Q443\|F1Q443\_CANLF, tr\|F1Q4I3\|F1Q4I3\_CANLF, tr\|F6V544\|F6V544\_CANLF, tr\|F6XBX5\|F6XBX5\_CANLF, tr\|F6XY66\|F6XY66\_CANLF, tr\|F6Y3P9\|F6Y3P9\_CANLF, tr\|J9NUY1\|J9NUY1\_CANLF, tr\|J9NV86\|J9NV86\_CANLF, tr\|J9NVN4\|J9NVN4\_CANLF, tr\|J9NVU0\|J9NVU0\_CANLF, tr\|J9NXT9\|J9NXT9\_CANLF, tr\|J9P1X5\|J9P1X5\_CANLF, tr\|J9P2G7\|J9P2G7\_CANLF, tr\|J9P4L9\|J9P4L9\_CANLF, tr\|J9P5X9\|J9P5X9\_CANLF, tr\|J9P702\|J9P702\_CANLF, tr\|J9P820\|J9P820\_CANLF, tr\|J9P8E3\|J9P8E3\_CANLF, tr\|J9P9H3\|J9P9H3\_CANLF, tr\|J9PAK3\|J9PAK3\_CANLF, tr\|Z4YHI2\|Z4YHI2\_CANLF |
| 4 | Pathways in cancer | sp\|F1PAA9\|CADH1\_CANLF, sp\|P41148\|ENPL\_CANLF, sp\|Q1HG70\|MP2K2\_CANLF, tr\|A0A140T8E6\|A0A140T8E6\_CANLF, tr\|A0A5F4BSL9\|A0A5F4BSL9\_CANLF, tr\|A0A5F4BVB0\|A0A5F4BVB0\_CANLF, tr\|A0A5F4C0L0\|A0A5F4C0L0\_CANLF, tr\|A0A5F4C4G4\|A0A5F4C4G4\_CANLF, tr\|A0A5F4C4S3\|A0A5F4C4S3\_CANLF, tr\|A0A5F4C566\|A0A5F4C566\_CANLF, tr\|A0A5F4C5D6\|A0A5F4C5D6\_CANLF, tr\|A0A5F4C5E5\|A0A5F4C5E5\_CANLF, tr\|A0A5F4C9R4\|A0A5F4C9R4\_CANLF, tr\|A0A5F4CB93\|A0A5F4CB93\_CANLF, tr\|A0A5F4CC58\|A0A5F4CC58\_CANLF, tr\|A0A5F4CE07\|A0A5F4CE07\_CANLF, tr\|A0A5F4CJ74\|A0A5F4CJ74\_CANLF, tr\|A0A5F4CNR7\|A0A5F4CNR7\_CANLF, tr\|A0A5F4CT67\|A0A5F4CT67\_CANLF, tr\|A0A5F4CYP2\|A0A5F4CYP2\_CANLF, tr\|A0A5F4D0Z3\|A0A5F4D0Z3\_CANLF, tr\|A0A5F4D5S9\|A0A5F4D5S9\_CANLF, tr\|A0A5F4D5V0\|A0A5F4D5V0\_CANLF, tr\|A0A5F4D6M3\|A0A5F4D6M3\_CANLF, tr\|A0A5F4D6R1\|A0A5F4D6R1\_CANLF, tr\|A0A5F4D721\|A0A5F4D721\_CANLF, tr\|A0A5F4DAI3\|A0A5F4DAI3\_CANLF, tr\|A0A5F4DCE0\|A0A5F4DCE0\_CANLF, tr\|A0A5F4DCI2\|A0A5F4DCI2\_CANLF, tr\|E2QWD3\|E2QWD3\_CANLF, tr\|E2R4J1\|E2R4J1\_CANLF, tr\|E2R651\|E2R651\_CANLF, tr\|E2R6J0\|E2R6J0\_CANLF, tr\|E2R9S7\|E2R9S7\_CANLF, tr\|E2RA06\|E2RA06\_CANLF, tr\|E2REP2\|E2REP2\_CANLF, tr\|E2RF98\|E2RF98\_CANLF, tr\|E2RGR9\|E2RGR9\_CANLF, tr\|E2RI99\|E2RI99\_CANLF, tr\|E2RLS3\|E2RLS3\_CANLF, tr\|E2RQX1\|E2RQX1\_CANLF, tr\|E2RRA9\|E2RRA9\_CANLF, tr\|E2RT60\|E2RT60\_CANLF, tr\|F1P874\|F1P874\_CANLF, tr\|F1PEX7\|F1PEX7\_CANLF, tr\|F1PF03\|F1PF03\_CANLF, tr\|F1PGB1\|F1PGB1\_CANLF, tr\|F1PGY1\|F1PGY1\_CANLF, tr\|F1PIK5\|F1PIK5\_CANLF, tr\|F1PKR8\|F1PKR8\_CANLF, tr\|F1PKU7\|F1PKU7\_CANLF, tr\|F1PNW8\|F1PNW8\_CANLF, tr\|F1PPM7\|F1PPM7\_CANLF, tr\|F1PQ60\|F1PQ60\_CANLF, tr\|F1PR63\|F1PR63\_CANLF, tr\|F1PR66\|F1PR66\_CANLF, tr\|F1PS09\|F1PS09\_CANLF, tr\|F1PYQ1\|F1PYQ1\_CANLF, tr\|F1Q0J0\|F1Q0J0\_CANLF, tr\|F1Q133\|F1Q133\_CANLF, tr\|F1Q1D5\|F1Q1D5\_CANLF, tr\|F1Q2Y7\|F1Q2Y7\_CANLF, tr\|F6XY66\|F6XY66\_CANLF, tr\|J9NVU0\|J9NVU0\_CANLF, tr\|J9NX28\|J9NX28\_CANLF, tr\|J9P1X5\|J9P1X5\_CANLF, tr\|J9P2Z2\|J9P2Z2\_CANLF, tr\|J9P4L9\|J9P4L9\_CANLF, tr\|J9P5X9\|J9P5X9\_CANLF, tr\|J9P614\|J9P614\_CANLF, tr\|Q28272\|Q28272\_CANLF, tr\|Q45KI9\|Q45KI9\_CANLF, tr\|Q597P9\|Q597P9\_CANLF, tr\|Q68V02\|Q68V02\_CANLF, tr\|Q867A1\|Q867A1\_CANLF, tr\|Q867A2\|Q867A2\_CANLF |
| 5 | Endocytosis | sp\|O46550\|CAV2\_CANLF, sp\|P33724\|CAV1\_CANLF, sp\|P51147\|RAB5C\_CANLF, sp\|Q5ECR9\|CCR5\_CANLF, tr\|A0A5F4BPT0\|A0A5F4BPT0\_CANLF, tr\|A0A5F4BQZ0\|A0A5F4BQZ0\_CANLF, tr\|A0A5F4BSN1\|A0A5F4BSN1\_CANLF, tr\|A0A5F4C1H0\|A0A5F4C1H0\_CANLF, tr\|A0A5F4C280\|A0A5F4C280\_CANLF, tr\|A0A5F4C5D6\|A0A5F4C5D6\_CANLF, tr\|A0A5F4CJC3\|A0A5F4CJC3\_CANLF, tr\|A0A5F4CK26\|A0A5F4CK26\_CANLF, tr\|A0A5F4CR65\|A0A5F4CR65\_CANLF, tr\|A0A5F4CUL0\|A0A5F4CUL0\_CANLF, tr\|A0A5F4CV58\|A0A5F4CV58\_CANLF, tr\|A0A5F4D062\|A0A5F4D062\_CANLF, tr\|A0A5F4D5V0\|A0A5F4D5V0\_CANLF, tr\|A0A5F4D662\|A0A5F4D662\_CANLF, tr\|A0A5F4D672\|A0A5F4D672\_CANLF, tr\|A0A5F4DAX4\|A0A5F4DAX4\_CANLF, tr\|A0A5F4DEW4\|A0A5F4DEW4\_CANLF, tr\|E2QVB0\|E2QVB0\_CANLF, tr\|E2QW49\|E2QW49\_CANLF, tr\|E2QWR9\|E2QWR9\_CANLF, tr\|E2QXM7\|E2QXM7\_CANLF, tr\|E2R273\|E2R273\_CANLF, tr\|E2R3R3\|E2R3R3\_CANLF, tr\|E2R417\|E2R417\_CANLF, tr\|E2R802\|E2R802\_CANLF, tr\|E2R8Q3\|E2R8Q3\_CANLF, tr\|E2RH35\|E2RH35\_CANLF, tr\|E2RPI9\|E2RPI9\_CANLF, tr\|F1P719\|F1P719\_CANLF, tr\|F1P946\|F1P946\_CANLF, tr\|F1P9H0\|F1P9H0\_CANLF, tr\|F1PAC1\|F1PAC1\_CANLF, tr\|F1PAM9\|F1PAM9\_CANLF, tr\|F1PF03\|F1PF03\_CANLF, tr\|F1PFB8\|F1PFB8\_CANLF, tr\|F1PFG6\|F1PFG6\_CANLF, tr\|F1PHQ0\|F1PHQ0\_CANLF, tr\|F1PJP7\|F1PJP7\_CANLF, tr\|F1PK11\|F1PK11\_CANLF, tr\|F1PM32\|F1PM32\_CANLF, tr\|F1PNP7\|F1PNP7\_CANLF, tr\|F1PNV8\|F1PNV8\_CANLF, tr\|F1PSK6\|F1PSK6\_CANLF, tr\|F1PVC5\|F1PVC5\_CANLF, tr\|F1PVE5\|F1PVE5\_CANLF, tr\|F1PYA8\|F1PYA8\_CANLF, tr\|F1PYW3\|F1PYW3\_CANLF, tr\|F1Q0X8\|F1Q0X8\_CANLF, tr\|F6X5M3\|F6X5M3\_CANLF, tr\|F6X9W7\|F6X9W7\_CANLF, tr\|F6XBX5\|F6XBX5\_CANLF, tr\|F6XY66\|F6XY66\_CANLF, tr\|G1K268\|G1K268\_CANLF, tr\|H9GW49\|H9GW49\_CANLF, tr\|J9JHN1\|J9JHN1\_CANLF, tr\|J9NTI1\|J9NTI1\_CANLF, tr\|J9NW32\|J9NW32\_CANLF, tr\|J9NWS6\|J9NWS6\_CANLF, tr\|J9NWU4\|J9NWU4\_CANLF, tr\|J9NWU6\|J9NWU6\_CANLF, tr\|J9NY42\|J9NY42\_CANLF, tr\|J9NZR2\|J9NZR2\_CANLF, tr\|J9P2S1\|J9P2S1\_CANLF, tr\|J9P987\|J9P987\_CANLF, tr\|L7MTN8\|L7MTN8\_CANLF, tr\|O46880\|O46880\_CANLF, tr\|O46882\|O46882\_CANLF |
| 6 | Spliceosome | tr\|A0A5F4C4T7\|A0A5F4C4T7\_CANLF, tr\|A0A5F4CHQ6\|A0A5F4CHQ6\_CANLF, tr\|A0A5F4CJL3\|A0A5F4CJL3\_CANLF, tr\|A0A5F4CK91\|A0A5F4CK91\_CANLF, tr\|A0A5F4D1E4\|A0A5F4D1E4\_CANLF, tr\|A0A5F4D2S8\|A0A5F4D2S8\_CANLF, tr\|A0A5F4D3P9\|A0A5F4D3P9\_CANLF, tr\|A0A5F4D764\|A0A5F4D764\_CANLF, tr\|A0A5F4DAY0\|A0A5F4DAY0\_CANLF, tr\|A0A5F4DFD3\|A0A5F4DFD3\_CANLF, tr\|A0A5F4DFZ6\|A0A5F4DFZ6\_CANLF, tr\|A0A5F4DG64\|A0A5F4DG64\_CANLF, tr\|A0A5F4DG78\|A0A5F4DG78\_CANLF, tr\|E2QU53\|E2QU53\_CANLF, tr\|E2QWU2\|E2QWU2\_CANLF, tr\|E2QXU5\|E2QXU5\_CANLF, tr\|E2QZF6\|E2QZF6\_CANLF, tr\|E2R068\|E2R068\_CANLF, tr\|E2R0G3\|E2R0G3\_CANLF, tr\|E2R1V3\|E2R1V3\_CANLF, tr\|E2R4I0\|E2R4I0\_CANLF, tr\|E2R4X9\|E2R4X9\_CANLF, tr\|E2R680\|E2R680\_CANLF, tr\|E2R6I0\|E2R6I0\_CANLF, tr\|E2R9R9\|E2R9R9\_CANLF, tr\|E2RCH8\|E2RCH8\_CANLF, tr\|E2RDB2\|E2RDB2\_CANLF, tr\|E2RDL8\|E2RDL8\_CANLF, tr\|E2RFV7\|E2RFV7\_CANLF, tr\|E2RJL3\|E2RJL3\_CANLF, tr\|E2RK20\|E2RK20\_CANLF, tr\|E2RL65\|E2RL65\_CANLF, tr\|E2RLP4\|E2RLP4\_CANLF, tr\|E2RLQ4\|E2RLQ4\_CANLF, tr\|E2RMQ0\|E2RMQ0\_CANLF, tr\|E2RPE2\|E2RPE2\_CANLF, tr\|E2RPX4\|E2RPX4\_CANLF, tr\|E2RQR0\|E2RQR0\_CANLF, tr\|E2RQV7\|E2RQV7\_CANLF, tr\|E2RR50\|E2RR50\_CANLF, tr\|E2RRM7\|E2RRM7\_CANLF, tr\|E2RSA6\|E2RSA6\_CANLF, tr\|E2RTK0\|E2RTK0\_CANLF, tr\|F1P818\|F1P818\_CANLF, tr\|F1P8K1\|F1P8K1\_CANLF, tr\|F1P9W3\|F1P9W3\_CANLF, tr\|F1PA16\|F1PA16\_CANLF, tr\|F1PEZ0\|F1PEZ0\_CANLF, tr\|F1PGL2\|F1PGL2\_CANLF, tr\|F1PIC2\|F1PIC2\_CANLF, tr\|F1PKF6\|F1PKF6\_CANLF, tr\|F1PL92\|F1PL92\_CANLF, tr\|F1PT07\|F1PT07\_CANLF, tr\|F1PUP7\|F1PUP7\_CANLF, tr\|F1Q0L5\|F1Q0L5\_CANLF, tr\|F1Q3G7\|F1Q3G7\_CANLF, tr\|F2Z4N6\|F2Z4N6\_CANLF, tr\|F6V5G7\|F6V5G7\_CANLF, tr\|F6XAE9\|F6XAE9\_CANLF, tr\|G1K268\|G1K268\_CANLF, tr\|H9GW49\|H9GW49\_CANLF, tr\|J9JHA9\|J9JHA9\_CANLF, tr\|J9NZD6\|J9NZD6\_CANLF, tr\|J9P0D9\|J9P0D9\_CANLF, tr\|J9P334\|J9P334\_CANLF, tr\|J9P4I7\|J9P4I7\_CANLF, tr\|J9P4S4\|J9P4S4\_CANLF, tr\|J9P8J3\|J9P8J3\_CANLF, tr\|J9PA38\|J9PA38\_CANLF |
| 7 | Tight junction | sp\|A7YH32\|CING\_CANLF, sp\|O18840\|ACTB\_CANLF, sp\|O97758\|ZO1\_CANLF, sp\|P38400\|GNAI2\_CANLF, sp\|Q95KM5\|CLD3\_CANLF, tr\|A0A5F4BQZ0\|A0A5F4BQZ0\_CANLF, tr\|A0A5F4BYB4\|A0A5F4BYB4\_CANLF, tr\|A0A5F4C280\|A0A5F4C280\_CANLF, tr\|A0A5F4C3L2\|A0A5F4C3L2\_CANLF, tr\|A0A5F4C3X9\|A0A5F4C3X9\_CANLF, tr\|A0A5F4C8U3\|A0A5F4C8U3\_CANLF, tr\|A0A5F4CD60\|A0A5F4CD60\_CANLF, tr\|A0A5F4CIB4\|A0A5F4CIB4\_CANLF, tr\|A0A5F4CRZ5\|A0A5F4CRZ5\_CANLF, tr\|A0A5F4CT67\|A0A5F4CT67\_CANLF, tr\|A0A5F4CWM9\|A0A5F4CWM9\_CANLF, tr\|A0A5F4D0Z3\|A0A5F4D0Z3\_CANLF, tr\|A0A5F4D5V0\|A0A5F4D5V0\_CANLF, tr\|A0A5F4D662\|A0A5F4D662\_CANLF, tr\|A0A5F4D6R1\|A0A5F4D6R1\_CANLF, tr\|A0A5F4DC86\|A0A5F4DC86\_CANLF, tr\|A0A5F4DC95\|A0A5F4DC95\_CANLF, tr\|A0A5F4DFX5\|A0A5F4DFX5\_CANLF, tr\|A0A5F4DFZ5\|A0A5F4DFZ5\_CANLF, tr\|E2QV06\|E2QV06\_CANLF, tr\|E2QXY7\|E2QXY7\_CANLF, tr\|E2QY08\|E2QY08\_CANLF, tr\|E2QY85\|E2QY85\_CANLF, tr\|E2QYC9\|E2QYC9\_CANLF, tr\|E2R1P6\|E2R1P6\_CANLF, tr\|E2R215\|E2R215\_CANLF, tr\|E2R9S7\|E2R9S7\_CANLF, tr\|E2RGU7\|E2RGU7\_CANLF, tr\|E2RJV1\|E2RJV1\_CANLF, tr\|E2RNP2\|E2RNP2\_CANLF, tr\|E2RRM5\|E2RRM5\_CANLF, tr\|F1P634\|F1P634\_CANLF, tr\|F1P759\|F1P759\_CANLF, tr\|F1P7S2\|F1P7S2\_CANLF, tr\|F1PAP5\|F1PAP5\_CANLF, tr\|F1PDX2\|F1PDX2\_CANLF, tr\|F1PJ01\|F1PJ01\_CANLF, tr\|F1PKR8\|F1PKR8\_CANLF, tr\|F1PKU7\|F1PKU7\_CANLF, tr\|F1PP71\|F1PP71\_CANLF, tr\|F1PVV7\|F1PVV7\_CANLF, tr\|F1PX75\|F1PX75\_CANLF, tr\|F1PXT8\|F1PXT8\_CANLF, tr\|F1PXU1\|F1PXU1\_CANLF, tr\|F1PYS6\|F1PYS6\_CANLF, tr\|F1Q080\|F1Q080\_CANLF, tr\|F1Q1D5\|F1Q1D5\_CANLF, tr\|F1Q253\|F1Q253\_CANLF, tr\|F1Q2K7\|F1Q2K7\_CANLF, tr\|F6V234\|F6V234\_CANLF, tr\|F6XY66\|F6XY66\_CANLF, tr\|F6Y429\|F6Y429\_CANLF, tr\|H9GW54\|H9GW54\_CANLF, tr\|J9NV99\|J9NV99\_CANLF, tr\|J9NWS2\|J9NWS2\_CANLF, tr\|J9NZ27\|J9NZ27\_CANLF, tr\|J9P0L7\|J9P0L7\_CANLF, tr\|J9P296\|J9P296\_CANLF, tr\|J9P6F5\|J9P6F5\_CANLF, tr\|J9P749\|J9P749\_CANLF, tr\|J9PAK3\|J9PAK3\_CANLF, tr\|Q9N0W3\|Q9N0W3\_CANLF, tr\|Z4YHI2\|Z4YHI2\_CANLF |
| 8 | Microbial metabolism in diverse environments (no map in kegg database) | sp\|O97492\|CATA\_CANLF, sp\|Q29476\|ST1A1\_CANLF, sp\|Q95LA2\|FMO1\_CANLF, tr\|A0A346M2B2\|A0A346M2B2\_CANLF, tr\|A0A5F4BUE3\|A0A5F4BUE3\_CANLF, tr\|A0A5F4BUZ8\|A0A5F4BUZ8\_CANLF, tr\|A0A5F4C4W3\|A0A5F4C4W3\_CANLF, tr\|A0A5F4C6G2\|A0A5F4C6G2\_CANLF, tr\|A0A5F4CF81\|A0A5F4CF81\_CANLF, tr\|A0A5F4CNR7\|A0A5F4CNR7\_CANLF, tr\|A0A5F4CNU9\|A0A5F4CNU9\_CANLF, tr\|A0A5F4CVL0\|A0A5F4CVL0\_CANLF, tr\|A0A5F4CZB6\|A0A5F4CZB6\_CANLF, tr\|A0A5F4D491\|A0A5F4D491\_CANLF, tr\|A0A5F4D6M3\|A0A5F4D6M3\_CANLF, tr\|A0A5F4D8P8\|A0A5F4D8P8\_CANLF, tr\|A0A5F4DAB6\|A0A5F4DAB6\_CANLF, tr\|A0A5F4DCR1\|A0A5F4DCR1\_CANLF, tr\|A0A5F4DIX0\|A0A5F4DIX0\_CANLF, tr\|A0A5F4DKH0\|A0A5F4DKH0\_CANLF, tr\|E2QV08\|E2QV08\_CANLF, tr\|E2QV12\|E2QV12\_CANLF, tr\|E2QVM3\|E2QVM3\_CANLF, tr\|E2R1E3\|E2R1E3\_CANLF, tr\|E2R2C3\|E2R2C3\_CANLF, tr\|E2R5U8\|E2R5U8\_CANLF, tr\|E2R9U0\|E2R9U0\_CANLF, tr\|E2REJ7\|E2REJ7\_CANLF, tr\|E2RGR9\|E2RGR9\_CANLF, tr\|E2RHC8\|E2RHC8\_CANLF, tr\|E2RLF5\|E2RLF5\_CANLF, tr\|E2RQ99\|E2RQ99\_CANLF, tr\|E2RRC9\|E2RRC9\_CANLF, tr\|E2RT65\|E2RT65\_CANLF, tr\|F1PAZ6\|F1PAZ6\_CANLF, tr\|F1PBZ5\|F1PBZ5\_CANLF, tr\|F1PE09\|F1PE09\_CANLF, tr\|F1PIP0\|F1PIP0\_CANLF, tr\|F1PJD1\|F1PJD1\_CANLF, tr\|F1PK43\|F1PK43\_CANLF, tr\|F1PKG8\|F1PKG8\_CANLF, tr\|F1PP35\|F1PP35\_CANLF, tr\|F1PPF7\|F1PPF7\_CANLF, tr\|F1PTS5\|F1PTS5\_CANLF, tr\|F1PU81\|F1PU81\_CANLF, tr\|F1PV92\|F1PV92\_CANLF, tr\|F1PVW0\|F1PVW0\_CANLF, tr\|F1PXN6\|F1PXN6\_CANLF, tr\|F1PYB6\|F1PYB6\_CANLF, tr\|F1PYG8\|F1PYG8\_CANLF, tr\|F1PZA1\|F1PZA1\_CANLF, tr\|F1Q0J0\|F1Q0J0\_CANLF, tr\|F1Q3S9\|F1Q3S9\_CANLF, tr\|F1Q3V2\|F1Q3V2\_CANLF, tr\|F6XHT8\|F6XHT8\_CANLF, tr\|H9GW87\|H9GW87\_CANLF, tr\|J9JHD4\|J9JHD4\_CANLF, tr\|J9NZC6\|J9NZC6\_CANLF, tr\|J9P208\|J9P208\_CANLF, tr\|J9P3N9\|J9P3N9\_CANLF, tr\|J9P7A6\|J9P7A6\_CANLF, tr\|J9P7N4\|J9P7N4\_CANLF, tr\|L7N0H7\|L7N0H7\_CANLF, tr\|Q28226\|Q28226\_CANLF |
| 9 | RNA transport | sp\|P62825\|RAN\_CANLF, sp\|P79149\|PININ\_CANLF, tr\|A0A5F4BNY6\|A0A5F4BNY6\_CANLF, tr\|A0A5F4BXH1\|A0A5F4BXH1\_CANLF, tr\|A0A5F4C3Q7\|A0A5F4C3Q7\_CANLF, tr\|A0A5F4C3W6\|A0A5F4C3W6\_CANLF, tr\|A0A5F4C4T7\|A0A5F4C4T7\_CANLF, tr\|A0A5F4CA63\|A0A5F4CA63\_CANLF, tr\|A0A5F4CCZ9\|A0A5F4CCZ9\_CANLF, tr\|A0A5F4CEZ8\|A0A5F4CEZ8\_CANLF, tr\|A0A5F4CH02\|A0A5F4CH02\_CANLF, tr\|A0A5F4CJL3\|A0A5F4CJL3\_CANLF, tr\|A0A5F4D555\|A0A5F4D555\_CANLF, tr\|A0A5F4D672\|A0A5F4D672\_CANLF, tr\|A0A5F4D713\|A0A5F4D713\_CANLF, tr\|A0A5F4DEP2\|A0A5F4DEP2\_CANLF, tr\|A0A5F4DFQ0\|A0A5F4DFQ0\_CANLF, tr\|A0A5F4DGH8\|A0A5F4DGH8\_CANLF, tr\|A0A5F4DHH9\|A0A5F4DHH9\_CANLF, tr\|A0A5F4DIQ5\|A0A5F4DIQ5\_CANLF, tr\|E2QWR9\|E2QWR9\_CANLF, tr\|E2R1J6\|E2R1J6\_CANLF, tr\|E2R334\|E2R334\_CANLF, tr\|E2R4V6\|E2R4V6\_CANLF, tr\|E2R5K5\|E2R5K5\_CANLF, tr\|E2R9K4\|E2R9K4\_CANLF, tr\|E2RCA5\|E2RCA5\_CANLF, tr\|E2REK3\|E2REK3\_CANLF, tr\|E2RFM7\|E2RFM7\_CANLF, tr\|E2RGG6\|E2RGG6\_CANLF, tr\|E2RGN5\|E2RGN5\_CANLF, tr\|E2RGZ5\|E2RGZ5\_CANLF, tr\|E2RL17\|E2RL17\_CANLF, tr\|E2RL29\|E2RL29\_CANLF, tr\|E2RLN2\|E2RLN2\_CANLF, tr\|E2RMK6\|E2RMK6\_CANLF, tr\|E2RPA3\|E2RPA3\_CANLF, tr\|E2RQP6\|E2RQP6\_CANLF, tr\|E2RR50\|E2RR50\_CANLF, tr\|F1P6G3\|F1P6G3\_CANLF, tr\|F1P969\|F1P969\_CANLF, tr\|F1PDJ2\|F1PDJ2\_CANLF, tr\|F1PDM3\|F1PDM3\_CANLF, tr\|F1PL92\|F1PL92\_CANLF, tr\|F1PT07\|F1PT07\_CANLF, tr\|F1PUP7\|F1PUP7\_CANLF, tr\|F1PVT1\|F1PVT1\_CANLF, tr\|F1PZL7\|F1PZL7\_CANLF, tr\|F6V4W0\|F6V4W0\_CANLF, tr\|F6X637\|F6X637\_CANLF, tr\|F6XAE9\|F6XAE9\_CANLF, tr\|F6XD36\|F6XD36\_CANLF, tr\|F6XEW2\|F6XEW2\_CANLF, tr\|F6XRK3\|F6XRK3\_CANLF, tr\|J9NU36\|J9NU36\_CANLF, tr\|J9NW32\|J9NW32\_CANLF, tr\|J9P0U9\|J9P0U9\_CANLF, tr\|J9P2W5\|J9P2W5\_CANLF, tr\|J9P357\|J9P357\_CANLF, tr\|J9P504\|J9P504\_CANLF, tr\|L7N0M6\|L7N0M6\_CANLF, tr\|S5U7Q9\|S5U7Q9\_CANLF |
| 10 | Leukocyte transendothelial migration | sp\|O18840\|ACTB\_CANLF, sp\|P38400\|GNAI2\_CANLF, sp\|Q95KM5\|CLD3\_CANLF, tr\|A0A5F4BYB4\|A0A5F4BYB4\_CANLF, tr\|A0A5F4C173\|A0A5F4C173\_CANLF, tr\|A0A5F4C3X9\|A0A5F4C3X9\_CANLF, tr\|A0A5F4CRM4\|A0A5F4CRM4\_CANLF, tr\|A0A5F4CWP8\|A0A5F4CWP8\_CANLF, tr\|A0A5F4D0Z3\|A0A5F4D0Z3\_CANLF, tr\|A0A5F4D5V0\|A0A5F4D5V0\_CANLF, tr\|A0A5F4DC86\|A0A5F4DC86\_CANLF, tr\|A0A5F4DCP4\|A0A5F4DCP4\_CANLF, tr\|A0A5F4DFX5\|A0A5F4DFX5\_CANLF, tr\|A0A5F4DIK8\|A0A5F4DIK8\_CANLF, tr\|E2QUL4\|E2QUL4\_CANLF, tr\|E2QXY7\|E2QXY7\_CANLF, tr\|E2QY08\|E2QY08\_CANLF, tr\|E2QY85\|E2QY85\_CANLF, tr\|E2R049\|E2R049\_CANLF, tr\|E2R2K4\|E2R2K4\_CANLF, tr\|E2R434\|E2R434\_CANLF, tr\|E2R8P3\|E2R8P3\_CANLF, tr\|E2R9S7\|E2R9S7\_CANLF, tr\|E2RE03\|E2RE03\_CANLF, tr\|E2RED4\|E2RED4\_CANLF, tr\|E2RRA9\|E2RRA9\_CANLF, tr\|E2RRM5\|E2RRM5\_CANLF, tr\|E2RSI6\|E2RSI6\_CANLF, tr\|E2RT60\|E2RT60\_CANLF, tr\|E2RTE0\|E2RTE0\_CANLF, tr\|F1P634\|F1P634\_CANLF, tr\|F1P7S2\|F1P7S2\_CANLF, tr\|F1PB95\|F1PB95\_CANLF, tr\|F1PCR1\|F1PCR1\_CANLF, tr\|F1PEM6\|F1PEM6\_CANLF, tr\|F1PKR3\|F1PKR3\_CANLF, tr\|F1PKU7\|F1PKU7\_CANLF, tr\|F1PP71\|F1PP71\_CANLF, tr\|F1PQ29\|F1PQ29\_CANLF, tr\|F1PUT6\|F1PUT6\_CANLF, tr\|F1PYL8\|F1PYL8\_CANLF, tr\|F1PYQ1\|F1PYQ1\_CANLF, tr\|F1Q1D5\|F1Q1D5\_CANLF, tr\|F1Q4I3\|F1Q4I3\_CANLF, tr\|F6UT09\|F6UT09\_CANLF, tr\|F6V544\|F6V544\_CANLF, tr\|F6XY66\|F6XY66\_CANLF, tr\|F6Y429\|F6Y429\_CANLF, tr\|J9NUY1\|J9NUY1\_CANLF, tr\|J9NXT9\|J9NXT9\_CANLF, tr\|J9P1X5\|J9P1X5\_CANLF, tr\|J9P296\|J9P296\_CANLF, tr\|J9P5X9\|J9P5X9\_CANLF, tr\|J9P8W1\|J9P8W1\_CANLF, tr\|J9PAK3\|J9PAK3\_CANLF, tr\|Q9N0W3\|Q9N0W3\_CANLF, tr\|Z4YHI2\|Z4YHI2\_CANLF |
| 11 | MAPK signaling pathway | sp\|Q1HG70\|MP2K2\_CANLF, tr\|A0A5F4C0Y2\|A0A5F4C0Y2\_CANLF, tr\|A0A5F4C4S3\|A0A5F4C4S3\_CANLF, tr\|A0A5F4C566\|A0A5F4C566\_CANLF, tr\|A0A5F4C5E5\|A0A5F4C5E5\_CANLF, tr\|A0A5F4CA24\|A0A5F4CA24\_CANLF, tr\|A0A5F4CCM0\|A0A5F4CCM0\_CANLF, tr\|A0A5F4CE39\|A0A5F4CE39\_CANLF, tr\|A0A5F4CUX4\|A0A5F4CUX4\_CANLF, tr\|A0A5F4CVA1\|A0A5F4CVA1\_CANLF, tr\|A0A5F4CWX2\|A0A5F4CWX2\_CANLF, tr\|A0A5F4CXA1\|A0A5F4CXA1\_CANLF, tr\|A0A5F4CXJ9\|A0A5F4CXJ9\_CANLF, tr\|A0A5F4D5V0\|A0A5F4D5V0\_CANLF, tr\|A0A5F4DF90\|A0A5F4DF90\_CANLF, tr\|E2QUK3\|E2QUK3\_CANLF, tr\|E2QWD3\|E2QWD3\_CANLF, tr\|E2QWG3\|E2QWG3\_CANLF, tr\|E2R2K4\|E2R2K4\_CANLF, tr\|E2R3R9\|E2R3R9\_CANLF, tr\|E2R597\|E2R597\_CANLF, tr\|E2RCV5\|E2RCV5\_CANLF, tr\|E2RE02\|E2RE02\_CANLF, tr\|E2REH3\|E2REH3\_CANLF, tr\|E2RF98\|E2RF98\_CANLF, tr\|E2RM17\|E2RM17\_CANLF, tr\|F1P750\|F1P750\_CANLF, tr\|F1P874\|F1P874\_CANLF, tr\|F1PAC5\|F1PAC5\_CANLF, tr\|F1PBJ8\|F1PBJ8\_CANLF, tr\|F1PF03\|F1PF03\_CANLF, tr\|F1PGB1\|F1PGB1\_CANLF, tr\|F1PH12\|F1PH12\_CANLF, tr\|F1PKI6\|F1PKI6\_CANLF, tr\|F1PKU7\|F1PKU7\_CANLF, tr\|F1PRZ2\|F1PRZ2\_CANLF, tr\|F1PWW0\|F1PWW0\_CANLF, tr\|F1PYE3\|F1PYE3\_CANLF, tr\|F1PYQ1\|F1PYQ1\_CANLF, tr\|F1Q1D5\|F1Q1D5\_CANLF, tr\|F2Z4P5\|F2Z4P5\_CANLF, tr\|F6UQ33\|F6UQ33\_CANLF, tr\|F6URD2\|F6URD2\_CANLF, tr\|F6XBX5\|F6XBX5\_CANLF, tr\|G1K268\|G1K268\_CANLF, tr\|H9GW49\|H9GW49\_CANLF, tr\|H9GWG4\|H9GWG4\_CANLF, tr\|J9NVP2\|J9NVP2\_CANLF, tr\|J9NXT4\|J9NXT4\_CANLF, tr\|J9NY42\|J9NY42\_CANLF, tr\|J9P381\|J9P381\_CANLF, tr\|J9P4L9\|J9P4L9\_CANLF, tr\|J9P5S3\|J9P5S3\_CANLF, tr\|J9P702\|J9P702\_CANLF |
| 12 | Chemokine signaling pathway | sp\|P30803\|ADCY5\_CANLF, sp\|P38400\|GNAI2\_CANLF, sp\|P62872\|GBB1\_CANLF, sp\|Q5ECR9\|CCR5\_CANLF, tr\|A0A5F4BRD0\|A0A5F4BRD0\_CANLF, tr\|A0A5F4BVY4\|A0A5F4BVY4\_CANLF, tr\|A0A5F4C280\|A0A5F4C280\_CANLF, tr\|A0A5F4C4S3\|A0A5F4C4S3\_CANLF, tr\|A0A5F4C566\|A0A5F4C566\_CANLF, tr\|A0A5F4C5E5\|A0A5F4C5E5\_CANLF, tr\|A0A5F4CBU4\|A0A5F4CBU4\_CANLF, tr\|A0A5F4CC58\|A0A5F4CC58\_CANLF, tr\|A0A5F4D4H5\|A0A5F4D4H5\_CANLF, tr\|A0A5F4D5V0\|A0A5F4D5V0\_CANLF, tr\|A0A5F4D662\|A0A5F4D662\_CANLF, tr\|A0A5F4DCP4\|A0A5F4DCP4\_CANLF, tr\|A0A5F4DIK8\|A0A5F4DIK8\_CANLF, tr\|E2QUL4\|E2QUL4\_CANLF, tr\|E2QWD3\|E2QWD3\_CANLF, tr\|E2R0N1\|E2R0N1\_CANLF, tr\|E2R2K4\|E2R2K4\_CANLF, tr\|E2R2S8\|E2R2S8\_CANLF, tr\|E2REH3\|E2REH3\_CANLF, tr\|E2RF98\|E2RF98\_CANLF, tr\|E2RGU7\|E2RGU7\_CANLF, tr\|E2RI99\|E2RI99\_CANLF, tr\|E2RNP2\|E2RNP2\_CANLF, tr\|E2RRA9\|E2RRA9\_CANLF, tr\|E2RTE0\|E2RTE0\_CANLF, tr\|F1P874\|F1P874\_CANLF, tr\|F1PAE3\|F1PAE3\_CANLF, tr\|F1PC86\|F1PC86\_CANLF, tr\|F1PDX2\|F1PDX2\_CANLF, tr\|F1PKU7\|F1PKU7\_CANLF, tr\|F1PNW8\|F1PNW8\_CANLF, tr\|F1PQC4\|F1PQC4\_CANLF, tr\|F1PUT6\|F1PUT6\_CANLF, tr\|F1PYQ1\|F1PYQ1\_CANLF, tr\|F1Q1D5\|F1Q1D5\_CANLF, tr\|F1Q4I3\|F1Q4I3\_CANLF, tr\|F6V544\|F6V544\_CANLF, tr\|F6X9W7\|F6X9W7\_CANLF, tr\|F6XY66\|F6XY66\_CANLF, tr\|J9NXT4\|J9NXT4\_CANLF, tr\|J9NXT9\|J9NXT9\_CANLF, tr\|J9NY42\|J9NY42\_CANLF, tr\|J9P1X5\|J9P1X5\_CANLF, tr\|J9P2M3\|J9P2M3\_CANLF, tr\|J9P4L9\|J9P4L9\_CANLF, tr\|J9P702\|J9P702\_CANLF |
| 13 | Adherens junction | sp\|F1PAA9\|CADH1\_CANLF, sp\|O18840\|ACTB\_CANLF, sp\|O97758\|ZO1\_CANLF, tr\|A0A5F4C173\|A0A5F4C173\_CANLF, tr\|A0A5F4C280\|A0A5F4C280\_CANLF, tr\|A0A5F4C3X9\|A0A5F4C3X9\_CANLF, tr\|A0A5F4C5E5\|A0A5F4C5E5\_CANLF, tr\|A0A5F4CRM4\|A0A5F4CRM4\_CANLF, tr\|A0A5F4D0W8\|A0A5F4D0W8\_CANLF, tr\|A0A5F4D0Z3\|A0A5F4D0Z3\_CANLF, tr\|A0A5F4D5V0\|A0A5F4D5V0\_CANLF, tr\|A0A5F4D662\|A0A5F4D662\_CANLF, tr\|A0A5F4D6Z6\|A0A5F4D6Z6\_CANLF, tr\|A0A5F4DAB6\|A0A5F4DAB6\_CANLF, tr\|A0A5F4DC86\|A0A5F4DC86\_CANLF, tr\|A0A5F4DEY9\|A0A5F4DEY9\_CANLF, tr\|A0A5F4DFX5\|A0A5F4DFX5\_CANLF, tr\|E2QXY7\|E2QXY7\_CANLF, tr\|E2QY08\|E2QY08\_CANLF, tr\|E2R3R9\|E2R3R9\_CANLF, tr\|E2R434\|E2R434\_CANLF, tr\|E2R5Q1\|E2R5Q1\_CANLF, tr\|E2R9S7\|E2R9S7\_CANLF, tr\|E2R9U0\|E2R9U0\_CANLF, tr\|E2RC09\|E2RC09\_CANLF, tr\|E2REU8\|E2REU8\_CANLF, tr\|E2RLF5\|E2RLF5\_CANLF, tr\|E2RNM8\|E2RNM8\_CANLF, tr\|E2RRM5\|E2RRM5\_CANLF, tr\|F1P7S2\|F1P7S2\_CANLF, tr\|F1PF03\|F1PF03\_CANLF, tr\|F1PHJ6\|F1PHJ6\_CANLF, tr\|F1PJ65\|F1PJ65\_CANLF, tr\|F1PJD1\|F1PJD1\_CANLF, tr\|F1PKG8\|F1PKG8\_CANLF, tr\|F1PKR3\|F1PKR3\_CANLF, tr\|F1PL36\|F1PL36\_CANLF, tr\|F1PND0\|F1PND0\_CANLF, tr\|F1PU81\|F1PU81\_CANLF, tr\|F1PUA5\|F1PUA5\_CANLF, tr\|F1PYQ1\|F1PYQ1\_CANLF, tr\|F1Q0X8\|F1Q0X8\_CANLF, tr\|F6XY66\|F6XY66\_CANLF, tr\|F6Y429\|F6Y429\_CANLF, tr\|J9NVN4\|J9NVN4\_CANLF, tr\|J9P766\|J9P766\_CANLF, tr\|J9PAK3\|J9PAK3\_CANLF, tr\|Z4YHI2\|Z4YHI2\_CANLF |
| 14 | Vascular smooth muscle contraction | sp\|P30803\|ADCY5\_CANLF, sp\|P63091\|GNAS\_CANLF, sp\|Q1HG70\|MP2K2\_CANLF, tr\|A0A5F4BSP1\|A0A5F4BSP1\_CANLF, tr\|A0A5F4BVY4\|A0A5F4BVY4\_CANLF, tr\|A0A5F4C5E5\|A0A5F4C5E5\_CANLF, tr\|A0A5F4C962\|A0A5F4C962\_CANLF, tr\|A0A5F4CNW6\|A0A5F4CNW6\_CANLF, tr\|A0A5F4CWM9\|A0A5F4CWM9\_CANLF, tr\|A0A5F4CXZ9\|A0A5F4CXZ9\_CANLF, tr\|A0A5F4CYN5\|A0A5F4CYN5\_CANLF, tr\|A0A5F4DDV9\|A0A5F4DDV9\_CANLF, tr\|E2QUK2\|E2QUK2\_CANLF, tr\|E2RGU7\|E2RGU7\_CANLF, tr\|E2RNP2\|E2RNP2\_CANLF, tr\|F1P634\|F1P634\_CANLF, tr\|F1P874\|F1P874\_CANLF, tr\|F1PBZ1\|F1PBZ1\_CANLF, tr\|F1PDX2\|F1PDX2\_CANLF, tr\|F1PET2\|F1PET2\_CANLF, tr\|F1PI77\|F1PI77\_CANLF, tr\|F1PI78\|F1PI78\_CANLF, tr\|F1PIF6\|F1PIF6\_CANLF, tr\|F1PJ01\|F1PJ01\_CANLF, tr\|F1PKU7\|F1PKU7\_CANLF, tr\|F1PVV7\|F1PVV7\_CANLF, tr\|F1PW86\|F1PW86\_CANLF, tr\|F1PXM5\|F1PXM5\_CANLF, tr\|F1PYS6\|F1PYS6\_CANLF, tr\|F1Q1D5\|F1Q1D5\_CANLF, tr\|F1Q2K7\|F1Q2K7\_CANLF, tr\|F1Q443\|F1Q443\_CANLF, tr\|F1Q4I3\|F1Q4I3\_CANLF, tr\|F2Z4N0\|F2Z4N0\_CANLF, tr\|F2Z4N7\|F2Z4N7\_CANLF, tr\|F2Z4N8\|F2Z4N8\_CANLF, tr\|F6UQ33\|F6UQ33\_CANLF, tr\|F6XY66\|F6XY66\_CANLF, tr\|G1K2D5\|G1K2D5\_CANLF, tr\|J9NWJ7\|J9NWJ7\_CANLF, tr\|J9NXT4\|J9NXT4\_CANLF, tr\|J9P2M3\|J9P2M3\_CANLF, tr\|J9P3B5\|J9P3B5\_CANLF, tr\|J9P4L9\|J9P4L9\_CANLF, tr\|J9P8E3\|J9P8E3\_CANLF, tr\|J9P8S3\|J9P8S3\_CANLF, tr\|J9P9H3\|J9P9H3\_CANLF |
| 15 | Pathogenic Escherichia coli infection | sp\|F1PAA9\|CADH1\_CANLF, sp\|O18840\|ACTB\_CANLF, sp\|Q865S3\|ARHG2\_CANLF, sp\|Q95KM5\|CLD3\_CANLF, tr\|A0A5F4BQZ0\|A0A5F4BQZ0\_CANLF, tr\|A0A5F4BVY6\|A0A5F4BVY6\_CANLF, tr\|A0A5F4BYB4\|A0A5F4BYB4\_CANLF, tr\|A0A5F4CIB4\|A0A5F4CIB4\_CANLF, tr\|A0A5F4CT62\|A0A5F4CT62\_CANLF, tr\|A0A5F4CTR8\|A0A5F4CTR8\_CANLF, tr\|A0A5F4D0Z3\|A0A5F4D0Z3\_CANLF, tr\|A0A5F4D5V0\|A0A5F4D5V0\_CANLF, tr\|A0A5F4DJY1\|A0A5F4DJY1\_CANLF, tr\|E2QSF4\|E2QSF4\_CANLF, tr\|E2QWU0\|E2QWU0\_CANLF, tr\|E2QXY7\|E2QXY7\_CANLF, tr\|E2R150\|E2R150\_CANLF, tr\|E2RBC3\|E2RBC3\_CANLF, tr\|E2RC09\|E2RC09\_CANLF, tr\|E2REU6\|E2REU6\_CANLF, tr\|E2RHV6\|E2RHV6\_CANLF, tr\|E2RMT4\|E2RMT4\_CANLF, tr\|E2RNQ2\|E2RNQ2\_CANLF, tr\|E2RPY6\|E2RPY6\_CANLF, tr\|E2RSI6\|E2RSI6\_CANLF, tr\|E2RT60\|E2RT60\_CANLF, tr\|F1PAG6\|F1PAG6\_CANLF, tr\|F1PBL1\|F1PBL1\_CANLF, tr\|F1PEX7\|F1PEX7\_CANLF, tr\|F1PKU7\|F1PKU7\_CANLF, tr\|F1PKW7\|F1PKW7\_CANLF, tr\|F1Q0B0\|F1Q0B0\_CANLF, tr\|F1Q0N9\|F1Q0N9\_CANLF, tr\|F1Q1D5\|F1Q1D5\_CANLF, tr\|F1Q4I3\|F1Q4I3\_CANLF, tr\|F6XY66\|F6XY66\_CANLF, tr\|H9GW54\|H9GW54\_CANLF, tr\|J9NRH5\|J9NRH5\_CANLF, tr\|J9NYB2\|J9NYB2\_CANLF, tr\|J9P296\|J9P296\_CANLF, tr\|J9P6N4\|J9P6N4\_CANLF, tr\|J9PAA1\|J9PAA1\_CANLF, tr\|J9PAK3\|J9PAK3\_CANLF, tr\|L7N0B2\|L7N0B2\_CANLF, tr\|L7N0I7\|L7N0I7\_CANLF, tr\|Q9N0W3\|Q9N0W3\_CANLF, tr\|Z4YHI2\|Z4YHI2\_CANLF |
| 16 | Phagosome | sp\|O18840\|ACTB\_CANLF, sp\|P51147\|RAB5C\_CANLF, sp\|Q9GL24\|CATL1\_CANLF, tr\|A0A5F4BPT0\|A0A5F4BPT0\_CANLF, tr\|A0A5F4BUG1\|A0A5F4BUG1\_CANLF, tr\|A0A5F4C0Q0\|A0A5F4C0Q0\_CANLF, tr\|A0A5F4C9R4\|A0A5F4C9R4\_CANLF, tr\|A0A5F4CBH6\|A0A5F4CBH6\_CANLF, tr\|A0A5F4CZ62\|A0A5F4CZ62\_CANLF, tr\|A0A5F4CZW1\|A0A5F4CZW1\_CANLF, tr\|A0A5F4DBI9\|A0A5F4DBI9\_CANLF, tr\|A0A5F4DCP4\|A0A5F4DCP4\_CANLF, tr\|E2QSF4\|E2QSF4\_CANLF, tr\|E2QXJ5\|E2QXJ5\_CANLF, tr\|E2QXY7\|E2QXY7\_CANLF, tr\|E2R4C1\|E2R4C1\_CANLF, tr\|E2R4Q2\|E2R4Q2\_CANLF, tr\|E2RBC3\|E2RBC3\_CANLF, tr\|E2RCC8\|E2RCC8\_CANLF, tr\|E2RDX1\|E2RDX1\_CANLF, tr\|E2RE31\|E2RE31\_CANLF, tr\|E2RH71\|E2RH71\_CANLF, tr\|E2RNQ2\|E2RNQ2\_CANLF, tr\|E2RT60\|E2RT60\_CANLF, tr\|F1P6V8\|F1P6V8\_CANLF, tr\|F1P8B4\|F1P8B4\_CANLF, tr\|F1PGM1\|F1PGM1\_CANLF, tr\|F1PPG5\|F1PPG5\_CANLF, tr\|F1PQ52\|F1PQ52\_CANLF, tr\|F1PYQ1\|F1PYQ1\_CANLF, tr\|F1Q260\|F1Q260\_CANLF, tr\|F6UYJ9\|F6UYJ9\_CANLF, tr\|F6X9Y6\|F6X9Y6\_CANLF, tr\|G1K2D9\|G1K2D9\_CANLF, tr\|J9NUJ2\|J9NUJ2\_CANLF, tr\|J9NY60\|J9NY60\_CANLF, tr\|J9PAK3\|J9PAK3\_CANLF, tr\|J9PB51\|J9PB51\_CANLF, tr\|L7N0B2\|L7N0B2\_CANLF, tr\|L7N0I7\|L7N0I7\_CANLF, tr\|O46880\|O46880\_CANLF, tr\|O46882\|O46882\_CANLF, tr\|Q30348\|Q30348\_CANLF, tr\|Q5W417\|Q5W417\_CANLF, tr\|Z4YHI2\|Z4YHI2\_CANLF |
| 17 | Huntington's disease | sp\|Q8WNN6\|SODC\_CANLF, tr\|A0A5F4BSN1\|A0A5F4BSN1\_CANLF, tr\|A0A5F4BVY4\|A0A5F4BVY4\_CANLF, tr\|A0A5F4BYS0\|A0A5F4BYS0\_CANLF, tr\|A0A5F4CHV2\|A0A5F4CHV2\_CANLF, tr\|A0A5F4CWJ6\|A0A5F4CWJ6\_CANLF, tr\|A0A5F4D0C4\|A0A5F4D0C4\_CANLF, tr\|A0A5F4D0K7\|A0A5F4D0K7\_CANLF, tr\|A0A5F4D296\|A0A5F4D296\_CANLF, tr\|A0A5F4D2J2\|A0A5F4D2J2\_CANLF, tr\|A0A5F4D6E6\|A0A5F4D6E6\_CANLF, tr\|A0A5F4D804\|A0A5F4D804\_CANLF, tr\|A0A5F4DJV1\|A0A5F4DJV1\_CANLF, tr\|E2QUA8\|E2QUA8\_CANLF, tr\|E2QV28\|E2QV28\_CANLF, tr\|E2QYN0\|E2QYN0\_CANLF, tr\|E2QZ05\|E2QZ05\_CANLF, tr\|E2RD75\|E2RD75\_CANLF, tr\|E2RHL0\|E2RHL0\_CANLF, tr\|E2RJG8\|E2RJG8\_CANLF, tr\|E2RNG2\|E2RNG2\_CANLF, tr\|E2RPI9\|E2RPI9\_CANLF, tr\|F1PG47\|F1PG47\_CANLF, tr\|F1PHQ0\|F1PHQ0\_CANLF, tr\|F1PLL0\|F1PLL0\_CANLF, tr\|F1PLP2\|F1PLP2\_CANLF, tr\|F1PMN8\|F1PMN8\_CANLF, tr\|F1PR63\|F1PR63\_CANLF, tr\|F1PRZ1\|F1PRZ1\_CANLF, tr\|F1PW86\|F1PW86\_CANLF, tr\|F1PXM5\|F1PXM5\_CANLF, tr\|F1PY24\|F1PY24\_CANLF, tr\|F1PYJ0\|F1PYJ0\_CANLF, tr\|F1Q1J6\|F1Q1J6\_CANLF, tr\|F1Q3W8\|F1Q3W8\_CANLF, tr\|F1Q433\|F1Q433\_CANLF, tr\|F1Q435\|F1Q435\_CANLF, tr\|F6UMF1\|F6UMF1\_CANLF, tr\|F6X5M3\|F6X5M3\_CANLF, tr\|F6X895\|F6X895\_CANLF, tr\|F6XH46\|F6XH46\_CANLF, tr\|J9NWS6\|J9NWS6\_CANLF, tr\|J9P028\|J9P028\_CANLF, tr\|J9P8L1\|J9P8L1\_CANLF, tr\|V5LKV5\|V5LKV5\_CANLF |
| 18 | Bacterial invasion of epithelial cells | sp\|F1PAA9\|CADH1\_CANLF, sp\|O18840\|ACTB\_CANLF, sp\|O46550\|CAV2\_CANLF, sp\|P33724\|CAV1\_CANLF, tr\|A0A5F4BQZ0\|A0A5F4BQZ0\_CANLF, tr\|A0A5F4CB93\|A0A5F4CB93\_CANLF, tr\|A0A5F4CBU4\|A0A5F4CBU4\_CANLF, tr\|A0A5F4CIB4\|A0A5F4CIB4\_CANLF, tr\|A0A5F4CV58\|A0A5F4CV58\_CANLF, tr\|A0A5F4D0Z3\|A0A5F4D0Z3\_CANLF, tr\|A0A5F4D5S9\|A0A5F4D5S9\_CANLF, tr\|A0A5F4D5V0\|A0A5F4D5V0\_CANLF, tr\|A0A5F4DIK8\|A0A5F4DIK8\_CANLF, tr\|E2QUL4\|E2QUL4\_CANLF, tr\|E2QWD3\|E2QWD3\_CANLF, tr\|E2QWU0\|E2QWU0\_CANLF, tr\|E2QXY7\|E2QXY7\_CANLF, tr\|E2R5H4\|E2R5H4\_CANLF, tr\|E2R9S7\|E2R9S7\_CANLF, tr\|E2RF98\|E2RF98\_CANLF, tr\|E2RMT4\|E2RMT4\_CANLF, tr\|E2RPI9\|E2RPI9\_CANLF, tr\|E2RRA9\|E2RRA9\_CANLF, tr\|E2RT60\|E2RT60\_CANLF, tr\|E2RTE0\|E2RTE0\_CANLF, tr\|F1P803\|F1P803\_CANLF, tr\|F1PAG6\|F1PAG6\_CANLF, tr\|F1PFB8\|F1PFB8\_CANLF, tr\|F1PGF9\|F1PGF9\_CANLF, tr\|F1PHQ0\|F1PHQ0\_CANLF, tr\|F1PKR3\|F1PKR3\_CANLF, tr\|F1PQC4\|F1PQC4\_CANLF, tr\|F1PUT6\|F1PUT6\_CANLF, tr\|F1PUU5\|F1PUU5\_CANLF, tr\|F1PYQ1\|F1PYQ1\_CANLF, tr\|F6V544\|F6V544\_CANLF, tr\|F6X5M3\|F6X5M3\_CANLF, tr\|F6XY66\|F6XY66\_CANLF, tr\|H9GW54\|H9GW54\_CANLF, tr\|J9NXT9\|J9NXT9\_CANLF, tr\|J9P1X5\|J9P1X5\_CANLF, tr\|J9PAK3\|J9PAK3\_CANLF, tr\|L7MTN8\|L7MTN8\_CANLF, tr\|Z4YHI2\|Z4YHI2\_CANLF |
| 19 | Protein processing in endoplasmic reticulum | sp\|E2RG47\|STT3B\_CANLF, sp\|E2RQ08\|RPN1\_CANLF, sp\|P41148\|ENPL\_CANLF, sp\|P49256\|LMAN2\_CANLF, tr\|A0A5F4BSL9\|A0A5F4BSL9\_CANLF, tr\|A0A5F4CB52\|A0A5F4CB52\_CANLF, tr\|A0A5F4CP79\|A0A5F4CP79\_CANLF, tr\|A0A5F4CVB8\|A0A5F4CVB8\_CANLF, tr\|A0A5F4D0J5\|A0A5F4D0J5\_CANLF, tr\|E2R0K5\|E2R0K5\_CANLF, tr\|E2R4A5\|E2R4A5\_CANLF, tr\|E2R4Q6\|E2R4Q6\_CANLF, tr\|E2R5S2\|E2R5S2\_CANLF, tr\|E2R7L1\|E2R7L1\_CANLF, tr\|E2R8C9\|E2R8C9\_CANLF, tr\|E2RB37\|E2RB37\_CANLF, tr\|E2RD86\|E2RD86\_CANLF, tr\|E2RG27\|E2RG27\_CANLF, tr\|E2RHE9\|E2RHE9\_CANLF, tr\|E2RK67\|E2RK67\_CANLF, tr\|E2RKK6\|E2RKK6\_CANLF, tr\|E2RL02\|E2RL02\_CANLF, tr\|E2RL17\|E2RL17\_CANLF, tr\|E2RLQ9\|E2RLQ9\_CANLF, tr\|E2RLS3\|E2RLS3\_CANLF, tr\|E2RM09\|E2RM09\_CANLF, tr\|E2RRW0\|E2RRW0\_CANLF, tr\|F1P8B4\|F1P8B4\_CANLF, tr\|F1P975\|F1P975\_CANLF, tr\|F1P9W3\|F1P9W3\_CANLF, tr\|F1PDM3\|F1PDM3\_CANLF, tr\|F1PEI2\|F1PEI2\_CANLF, tr\|F1PGY1\|F1PGY1\_CANLF, tr\|F1PIC7\|F1PIC7\_CANLF, tr\|F1PL97\|F1PL97\_CANLF, tr\|F1PWN6\|F1PWN6\_CANLF, tr\|F6UYJ9\|F6UYJ9\_CANLF, tr\|F6UZV8\|F6UZV8\_CANLF, tr\|G1K268\|G1K268\_CANLF, tr\|H9GW49\|H9GW49\_CANLF, tr\|J9JHK6\|J9JHK6\_CANLF, tr\|J9NRG5\|J9NRG5\_CANLF, tr\|J9NUW8\|J9NUW8\_CANLF, tr\|J9P4L2\|J9P4L2\_CANLF |
| 20 | Neurotrophin signaling pathway | sp\|Q1HG70\|MP2K2\_CANLF, tr\|A0A5F4BVY6\|A0A5F4BVY6\_CANLF, tr\|A0A5F4C4S3\|A0A5F4C4S3\_CANLF, tr\|A0A5F4C566\|A0A5F4C566\_CANLF, tr\|A0A5F4C5E5\|A0A5F4C5E5\_CANLF, tr\|A0A5F4CJ79\|A0A5F4CJ79\_CANLF, tr\|A0A5F4CT62\|A0A5F4CT62\_CANLF, tr\|A0A5F4CTR8\|A0A5F4CTR8\_CANLF, tr\|A0A5F4CXJ9\|A0A5F4CXJ9\_CANLF, tr\|A0A5F4D429\|A0A5F4D429\_CANLF, tr\|A0A5F4D5V0\|A0A5F4D5V0\_CANLF, tr\|A0A5F4DJ85\|A0A5F4DJ85\_CANLF, tr\|A0A5K1UA11\|A0A5K1UA11\_CANLF, tr\|E2QWD3\|E2QWD3\_CANLF, tr\|E2QXE3\|E2QXE3\_CANLF, tr\|E2R2K4\|E2R2K4\_CANLF, tr\|E2R3U9\|E2R3U9\_CANLF, tr\|E2RAL0\|E2RAL0\_CANLF, tr\|E2RF98\|E2RF98\_CANLF, tr\|E2RG56\|E2RG56\_CANLF, tr\|E2RGU7\|E2RGU7\_CANLF, tr\|E2RMN7\|E2RMN7\_CANLF, tr\|E2RNP2\|E2RNP2\_CANLF, tr\|E2RRA9\|E2RRA9\_CANLF, tr\|F1P6N4\|F1P6N4\_CANLF, tr\|F1P750\|F1P750\_CANLF, tr\|F1P874\|F1P874\_CANLF, tr\|F1PBL1\|F1PBL1\_CANLF, tr\|F1PDX2\|F1PDX2\_CANLF, tr\|F1PEX7\|F1PEX7\_CANLF, tr\|F1PIV1\|F1PIV1\_CANLF, tr\|F1PKW7\|F1PKW7\_CANLF, tr\|F1PQC4\|F1PQC4\_CANLF, tr\|F1PYQ1\|F1PYQ1\_CANLF, tr\|F6XY66\|F6XY66\_CANLF, tr\|G1K2D5\|G1K2D5\_CANLF, tr\|J9NRH5\|J9NRH5\_CANLF, tr\|J9NWJ7\|J9NWJ7\_CANLF, tr\|J9P1X5\|J9P1X5\_CANLF, tr\|J9P4L9\|J9P4L9\_CANLF, tr\|J9P6N4\|J9P6N4\_CANLF, tr\|J9P8S3\|J9P8S3\_CANLF, tr\|J9PAA1\|J9PAA1\_CANLF, tr\|Q45KI9\|Q45KI9\_CANLF |
| 21 | Alzheimer's disease | sp\|P18649\|APOE\_CANLF, sp\|P56595\|APOC1\_CANLF, tr\|A0A5F4BVY4\|A0A5F4BVY4\_CANLF, tr\|A0A5F4BYS0\|A0A5F4BYS0\_CANLF, tr\|A0A5F4C0Y2\|A0A5F4C0Y2\_CANLF, tr\|A0A5F4C5E5\|A0A5F4C5E5\_CANLF, tr\|A0A5F4C9C0\|A0A5F4C9C0\_CANLF, tr\|A0A5F4CE07\|A0A5F4CE07\_CANLF, tr\|A0A5F4CNW6\|A0A5F4CNW6\_CANLF, tr\|A0A5F4D0C4\|A0A5F4D0C4\_CANLF, tr\|A0A5F4D0M2\|A0A5F4D0M2\_CANLF, tr\|A0A5F4D296\|A0A5F4D296\_CANLF, tr\|A0A5F4D6E6\|A0A5F4D6E6\_CANLF, tr\|A0A5F4DBL7\|A0A5F4DBL7\_CANLF, tr\|A0A5F4DJV1\|A0A5F4DJV1\_CANLF, tr\|A0A5K1UA11\|A0A5K1UA11\_CANLF, tr\|E2QUA8\|E2QUA8\_CANLF, tr\|E2QV28\|E2QV28\_CANLF, tr\|E2RD75\|E2RD75\_CANLF, tr\|E2RHL0\|E2RHL0\_CANLF, tr\|E2RJG8\|E2RJG8\_CANLF, tr\|E2RNG2\|E2RNG2\_CANLF, tr\|E2RS74\|E2RS74\_CANLF, tr\|F1P975\|F1P975\_CANLF, tr\|F1PG47\|F1PG47\_CANLF, tr\|F1PIK5\|F1PIK5\_CANLF, tr\|F1PLL0\|F1PLL0\_CANLF, tr\|F1PLP2\|F1PLP2\_CANLF, tr\|F1PW86\|F1PW86\_CANLF, tr\|F1PXM5\|F1PXM5\_CANLF, tr\|F1PY24\|F1PY24\_CANLF, tr\|F1PZR4\|F1PZR4\_CANLF, tr\|F1Q3W8\|F1Q3W8\_CANLF, tr\|F2Z4P5\|F2Z4P5\_CANLF, tr\|F6UZV8\|F6UZV8\_CANLF, tr\|G1K2D5\|G1K2D5\_CANLF, tr\|J9NWJ7\|J9NWJ7\_CANLF, tr\|J9P2B9\|J9P2B9\_CANLF, tr\|J9P8S3\|J9P8S3\_CANLF, tr\|L7N0H7\|L7N0H7\_CANLF, tr\|Q45KI9\|Q45KI9\_CANLF, tr\|V5LKV5\|V5LKV5\_CANLF |
| 22 | Insulin signaling pathway | sp\|Q1HG70\|MP2K2\_CANLF, tr\|A0A5F4C246\|A0A5F4C246\_CANLF, tr\|A0A5F4C4S3\|A0A5F4C4S3\_CANLF, tr\|A0A5F4C566\|A0A5F4C566\_CANLF, tr\|A0A5F4C5E5\|A0A5F4C5E5\_CANLF, tr\|A0A5F4CD52\|A0A5F4CD52\_CANLF, tr\|A0A5F4CKK8\|A0A5F4CKK8\_CANLF, tr\|A0A5F4CNS5\|A0A5F4CNS5\_CANLF, tr\|A0A5F4CT67\|A0A5F4CT67\_CANLF, tr\|A0A5F4D6R1\|A0A5F4D6R1\_CANLF, tr\|A0A5F4D6Z6\|A0A5F4D6Z6\_CANLF, tr\|A0A5F4DEY9\|A0A5F4DEY9\_CANLF, tr\|E2QSP3\|E2QSP3\_CANLF, tr\|E2QWD3\|E2QWD3\_CANLF, tr\|E2QZV5\|E2QZV5\_CANLF, tr\|E2RF98\|E2RF98\_CANLF, tr\|E2RP05\|E2RP05\_CANLF, tr\|E2RPA3\|E2RPA3\_CANLF, tr\|E2RRA1\|E2RRA1\_CANLF, tr\|E2RRA9\|E2RRA9\_CANLF, tr\|E2RRD1\|E2RRD1\_CANLF, tr\|F1P7Q1\|F1P7Q1\_CANLF, tr\|F1P874\|F1P874\_CANLF, tr\|F1PKR8\|F1PKR8\_CANLF, tr\|F1PND0\|F1PND0\_CANLF, tr\|F1PPF7\|F1PPF7\_CANLF, tr\|F1PQC4\|F1PQC4\_CANLF, tr\|F1PSM2\|F1PSM2\_CANLF, tr\|F1Q0X8\|F1Q0X8\_CANLF, tr\|F1Q2Y3\|F1Q2Y3\_CANLF, tr\|G1K2D5\|G1K2D5\_CANLF, tr\|J9NS46\|J9NS46\_CANLF, tr\|J9NWJ7\|J9NWJ7\_CANLF, tr\|J9NXT4\|J9NXT4\_CANLF, tr\|J9P1N8\|J9P1N8\_CANLF, tr\|J9P1X5\|J9P1X5\_CANLF, tr\|J9P4L9\|J9P4L9\_CANLF, tr\|J9P6E1\|J9P6E1\_CANLF, tr\|J9P8S3\|J9P8S3\_CANLF, tr\|J9PAP3\|J9PAP3\_CANLF, tr\|Q45KI9\|Q45KI9\_CANLF |
| 23 | Calcium signaling pathway | sp\|P63091\|GNAS\_CANLF, tr\|A0A5F4BVY4\|A0A5F4BVY4\_CANLF, tr\|A0A5F4C4P9\|A0A5F4C4P9\_CANLF, tr\|A0A5F4C9C0\|A0A5F4C9C0\_CANLF, tr\|A0A5F4CBH6\|A0A5F4CBH6\_CANLF, tr\|A0A5F4CJ79\|A0A5F4CJ79\_CANLF, tr\|A0A5F4CKN4\|A0A5F4CKN4\_CANLF, tr\|A0A5F4CNW6\|A0A5F4CNW6\_CANLF, tr\|A0A5F4CSQ5\|A0A5F4CSQ5\_CANLF, tr\|A0A5F4D429\|A0A5F4D429\_CANLF, tr\|A0A5F4D804\|A0A5F4D804\_CANLF, tr\|E2QVG6\|E2QVG6\_CANLF, tr\|E2QW49\|E2QW49\_CANLF, tr\|E2RCC8\|E2RCC8\_CANLF, tr\|E2RE31\|E2RE31\_CANLF, tr\|E2RG56\|E2RG56\_CANLF, tr\|E2RS74\|E2RS74\_CANLF, tr\|E2RSF7\|E2RSF7\_CANLF, tr\|F1P9Y2\|F1P9Y2\_CANLF, tr\|F1PF03\|F1PF03\_CANLF, tr\|F1PIF6\|F1PIF6\_CANLF, tr\|F1PKU7\|F1PKU7\_CANLF, tr\|F1PRZ1\|F1PRZ1\_CANLF, tr\|F1PVC5\|F1PVC5\_CANLF, tr\|F1PW86\|F1PW86\_CANLF, tr\|F1PWW8\|F1PWW8\_CANLF, tr\|F1PXM5\|F1PXM5\_CANLF, tr\|F1Q1D5\|F1Q1D5\_CANLF, tr\|F1Q1J6\|F1Q1J6\_CANLF, tr\|F2Z4P5\|F2Z4P5\_CANLF, tr\|F6X895\|F6X895\_CANLF, tr\|G1K2D5\|G1K2D5\_CANLF, tr\|J9NSZ5\|J9NSZ5\_CANLF, tr\|J9NWJ7\|J9NWJ7\_CANLF, tr\|J9NXT4\|J9NXT4\_CANLF, tr\|J9P2B9\|J9P2B9\_CANLF, tr\|J9P8P9\|J9P8P9\_CANLF, tr\|J9P8S3\|J9P8S3\_CANLF, tr\|J9P988\|J9P988\_CANLF, tr\|J9P9H3\|J9P9H3\_CANLF |
| 24 | Dilated cardiomyopathy | sp\|O18840\|ACTB\_CANLF, sp\|P30803\|ADCY5\_CANLF, sp\|P63091\|GNAS\_CANLF, sp\|Q5XFN2\|DESM\_CANLF, tr\|A0A5F4BVB0\|A0A5F4BVB0\_CANLF, tr\|A0A5F4BXT7\|A0A5F4BXT7\_CANLF, tr\|A0A5F4C4P9\|A0A5F4C4P9\_CANLF, tr\|A0A5F4C9C0\|A0A5F4C9C0\_CANLF, tr\|A0A5F4C9R4\|A0A5F4C9R4\_CANLF, tr\|A0A5F4CBF2\|A0A5F4CBF2\_CANLF, tr\|A0A5F4CBH6\|A0A5F4CBH6\_CANLF, tr\|A0A5F4CFM8\|A0A5F4CFM8\_CANLF, tr\|A0A5F4CUQ1\|A0A5F4CUQ1\_CANLF, tr\|A0A5F4CWM9\|A0A5F4CWM9\_CANLF, tr\|A0A5F4D721\|A0A5F4D721\_CANLF, tr\|A0A5F4DCE0\|A0A5F4DCE0\_CANLF, tr\|E2QXY7\|E2QXY7\_CANLF, tr\|E2RCC8\|E2RCC8\_CANLF, tr\|E2RDU7\|E2RDU7\_CANLF, tr\|E2RE31\|E2RE31\_CANLF, tr\|E2RRY2\|E2RRY2\_CANLF, tr\|E2RT60\|E2RT60\_CANLF, tr\|F1P6C3\|F1P6C3\_CANLF, tr\|F1P7G4\|F1P7G4\_CANLF, tr\|F1P8F3\|F1P8F3\_CANLF, tr\|F1PJ01\|F1PJ01\_CANLF, tr\|F1PP33\|F1PP33\_CANLF, tr\|F1PPG5\|F1PPG5\_CANLF, tr\|F1PQ29\|F1PQ29\_CANLF, tr\|F1PVV7\|F1PVV7\_CANLF, tr\|F1PYS6\|F1PYS6\_CANLF, tr\|G1K2D5\|G1K2D5\_CANLF, tr\|J9NSW5\|J9NSW5\_CANLF, tr\|J9NVU0\|J9NVU0\_CANLF, tr\|J9NXT4\|J9NXT4\_CANLF, tr\|J9P2B9\|J9P2B9\_CANLF, tr\|J9P2M3\|J9P2M3\_CANLF, tr\|J9P3Z7\|J9P3Z7\_CANLF, tr\|J9PAK3\|J9PAK3\_CANLF, tr\|Z4YHI2\|Z4YHI2\_CANLF |
| 25 | Amoebiasis | sp\|P51147\|RAB5C\_CANLF, sp\|P63091\|GNAS\_CANLF, tr\|A0A5F4BPT0\|A0A5F4BPT0\_CANLF, tr\|A0A5F4BVY4\|A0A5F4BVY4\_CANLF, tr\|A0A5F4C3X9\|A0A5F4C3X9\_CANLF, tr\|A0A5F4C4G4\|A0A5F4C4G4\_CANLF, tr\|A0A5F4CB93\|A0A5F4CB93\_CANLF, tr\|A0A5F4CBH6\|A0A5F4CBH6\_CANLF, tr\|A0A5F4CJ74\|A0A5F4CJ74\_CANLF, tr\|A0A5F4CUX4\|A0A5F4CUX4\_CANLF, tr\|A0A5F4D5S9\|A0A5F4D5S9\_CANLF, tr\|A0A5F4DC86\|A0A5F4DC86\_CANLF, tr\|A0A5F4DCI2\|A0A5F4DCI2\_CANLF, tr\|E2QY08\|E2QY08\_CANLF, tr\|E2R4J1\|E2R4J1\_CANLF, tr\|E2RCC8\|E2RCC8\_CANLF, tr\|E2RE31\|E2RE31\_CANLF, tr\|E2RKJ6\|E2RKJ6\_CANLF, tr\|E2RRA9\|E2RRA9\_CANLF, tr\|F1P7S2\|F1P7S2\_CANLF, tr\|F1PG69\|F1PG69\_CANLF, tr\|F1PHX8\|F1PHX8\_CANLF, tr\|F1PHY1\|F1PHY1\_CANLF, tr\|F1PKR3\|F1PKR3\_CANLF, tr\|F1PKU7\|F1PKU7\_CANLF, tr\|F1PR66\|F1PR66\_CANLF, tr\|F1PXM5\|F1PXM5\_CANLF, tr\|F1PYE3\|F1PYE3\_CANLF, tr\|F1Q133\|F1Q133\_CANLF, tr\|F1Q1D5\|F1Q1D5\_CANLF, tr\|F1Q3I5\|F1Q3I5\_CANLF, tr\|J9NSZ5\|J9NSZ5\_CANLF, tr\|J9NXT4\|J9NXT4\_CANLF, tr\|J9P1X5\|J9P1X5\_CANLF, tr\|J9P614\|J9P614\_CANLF, tr\|Q28272\|Q28272\_CANLF, tr\|Q597P9\|Q597P9\_CANLF, tr\|Q867A1\|Q867A1\_CANLF, tr\|Q867A2\|Q867A2\_CANLF |
| 26 | Fc gamma R-mediated phagocytosis | tr\|A0A5F4BU16\|A0A5F4BU16\_CANLF, tr\|A0A5F4C5E5\|A0A5F4C5E5\_CANLF, tr\|A0A5F4CBH6\|A0A5F4CBH6\_CANLF, tr\|A0A5F4CV58\|A0A5F4CV58\_CANLF, tr\|A0A5F4D4H5\|A0A5F4D4H5\_CANLF, tr\|A0A5F4D5V0\|A0A5F4D5V0\_CANLF, tr\|A0A5F4D9F1\|A0A5F4D9F1\_CANLF, tr\|A0A5F4DCP4\|A0A5F4DCP4\_CANLF, tr\|A0A5F4DDG2\|A0A5F4DDG2\_CANLF, tr\|A0A5F4DHY6\|A0A5F4DHY6\_CANLF, tr\|E2QWD3\|E2QWD3\_CANLF, tr\|E2QWU0\|E2QWU0\_CANLF, tr\|E2RCC8\|E2RCC8\_CANLF, tr\|E2RE31\|E2RE31\_CANLF, tr\|E2REH3\|E2REH3\_CANLF, tr\|E2RF98\|E2RF98\_CANLF, tr\|E2RGU7\|E2RGU7\_CANLF, tr\|E2RMT4\|E2RMT4\_CANLF, tr\|E2RNP2\|E2RNP2\_CANLF, tr\|E2RRA9\|E2RRA9\_CANLF, tr\|F1P946\|F1P946\_CANLF, tr\|F1PAG6\|F1PAG6\_CANLF, tr\|F1PDX2\|F1PDX2\_CANLF, tr\|F1PEM6\|F1PEM6\_CANLF, tr\|F1PFB8\|F1PFB8\_CANLF, tr\|F1PKU7\|F1PKU7\_CANLF, tr\|F1PYQ1\|F1PYQ1\_CANLF, tr\|F1PZH8\|F1PZH8\_CANLF, tr\|F1Q1D5\|F1Q1D5\_CANLF, tr\|F1Q1S4\|F1Q1S4\_CANLF, tr\|F6UQ33\|F6UQ33\_CANLF, tr\|F6V544\|F6V544\_CANLF, tr\|F6Y3P9\|F6Y3P9\_CANLF, tr\|F6Y4A3\|F6Y4A3\_CANLF, tr\|J9NV86\|J9NV86\_CANLF, tr\|J9NYG3\|J9NYG3\_CANLF, tr\|J9P1X5\|J9P1X5\_CANLF, tr\|J9P2G7\|J9P2G7\_CANLF, tr\|J9P4L9\|J9P4L9\_CANLF |
| 27 | ECM-receptor interaction | tr\|A0A5F4BVB0\|A0A5F4BVB0\_CANLF, tr\|A0A5F4C2A2\|A0A5F4C2A2\_CANLF, tr\|A0A5F4C4G4\|A0A5F4C4G4\_CANLF, tr\|A0A5F4C7K2\|A0A5F4C7K2\_CANLF, tr\|A0A5F4C9R4\|A0A5F4C9R4\_CANLF, tr\|A0A5F4CB93\|A0A5F4CB93\_CANLF, tr\|A0A5F4CJ74\|A0A5F4CJ74\_CANLF, tr\|A0A5F4CZG6\|A0A5F4CZG6\_CANLF, tr\|A0A5F4D5S9\|A0A5F4D5S9\_CANLF, tr\|A0A5F4DCI2\|A0A5F4DCI2\_CANLF, tr\|A0A5F4DFP9\|A0A5F4DFP9\_CANLF, tr\|E2R0D7\|E2R0D7\_CANLF, tr\|E2R480\|E2R480\_CANLF, tr\|E2R4J1\|E2R4J1\_CANLF, tr\|E2R6A0\|E2R6A0\_CANLF, tr\|E2RT60\|E2RT60\_CANLF, tr\|F1PF31\|F1PF31\_CANLF, tr\|F1PFM6\|F1PFM6\_CANLF, tr\|F1PG69\|F1PG69\_CANLF, tr\|F1PHX8\|F1PHX8\_CANLF, tr\|F1PHY1\|F1PHY1\_CANLF, tr\|F1PP33\|F1PP33\_CANLF, tr\|F1PPG5\|F1PPG5\_CANLF, tr\|F1PQ29\|F1PQ29\_CANLF, tr\|F1PR31\|F1PR31\_CANLF, tr\|F1PR66\|F1PR66\_CANLF, tr\|F1Q133\|F1Q133\_CANLF, tr\|F1Q2F7\|F1Q2F7\_CANLF, tr\|F1Q3I5\|F1Q3I5\_CANLF, tr\|F6X9Y6\|F6X9Y6\_CANLF, tr\|J9NRJ0\|J9NRJ0\_CANLF, tr\|J9NVU0\|J9NVU0\_CANLF, tr\|J9P423\|J9P423\_CANLF, tr\|J9P614\|J9P614\_CANLF, tr\|J9P889\|J9P889\_CANLF, tr\|Q28272\|Q28272\_CANLF, tr\|Q597P9\|Q597P9\_CANLF, tr\|Q867A1\|Q867A1\_CANLF, tr\|Q867A2\|Q867A2\_CANLF |
| 28 | Axon guidance | sp\|P38400\|GNAI2\_CANLF, tr\|A0A5F4C5E5\|A0A5F4C5E5\_CANLF, tr\|A0A5F4CVA1\|A0A5F4CVA1\_CANLF, tr\|A0A5F4D5V0\|A0A5F4D5V0\_CANLF, tr\|A0A5F4D9M8\|A0A5F4D9M8\_CANLF, tr\|A0A5F4DF90\|A0A5F4DF90\_CANLF, tr\|A0A5F4DHR2\|A0A5F4DHR2\_CANLF, tr\|A0A5F4DHY6\|A0A5F4DHY6\_CANLF, tr\|E2QY27\|E2QY27\_CANLF, tr\|E2R6A6\|E2R6A6\_CANLF, tr\|E2RC09\|E2RC09\_CANLF, tr\|E2REH3\|E2REH3\_CANLF, tr\|E2RFM4\|E2RFM4\_CANLF, tr\|E2RHV6\|E2RHV6\_CANLF, tr\|E2RKY7\|E2RKY7\_CANLF, tr\|E2RPY6\|E2RPY6\_CANLF, tr\|E2RT60\|E2RT60\_CANLF, tr\|F1P9U4\|F1P9U4\_CANLF, tr\|F1PDQ2\|F1PDQ2\_CANLF, tr\|F1PET2\|F1PET2\_CANLF, tr\|F1PEX7\|F1PEX7\_CANLF, tr\|F1PFV3\|F1PFV3\_CANLF, tr\|F1PU29\|F1PU29\_CANLF, tr\|F1PUA5\|F1PUA5\_CANLF, tr\|F1PWQ6\|F1PWQ6\_CANLF, tr\|F1PYQ1\|F1PYQ1\_CANLF, tr\|F1PYR0\|F1PYR0\_CANLF, tr\|F1Q2K7\|F1Q2K7\_CANLF, tr\|F1Q2N4\|F1Q2N4\_CANLF, tr\|F1Q3Y2\|F1Q3Y2\_CANLF, tr\|F1Q443\|F1Q443\_CANLF, tr\|F1Q4I3\|F1Q4I3\_CANLF, tr\|F2Z4P5\|F2Z4P5\_CANLF, tr\|F6V544\|F6V544\_CANLF, tr\|F6X9Y6\|F6X9Y6\_CANLF, tr\|F6XY66\|F6XY66\_CANLF, tr\|J9NV86\|J9NV86\_CANLF, tr\|J9P2G7\|J9P2G7\_CANLF |
| 29 | Hypertrophic cardiomyopathy (HCM) | sp\|O18840\|ACTB\_CANLF, sp\|Q5XFN2\|DESM\_CANLF, tr\|A0A5F4BVB0\|A0A5F4BVB0\_CANLF, tr\|A0A5F4BXT7\|A0A5F4BXT7\_CANLF, tr\|A0A5F4C4P9\|A0A5F4C4P9\_CANLF, tr\|A0A5F4C9C0\|A0A5F4C9C0\_CANLF, tr\|A0A5F4C9R4\|A0A5F4C9R4\_CANLF, tr\|A0A5F4CBF2\|A0A5F4CBF2\_CANLF, tr\|A0A5F4CD52\|A0A5F4CD52\_CANLF, tr\|A0A5F4CFM8\|A0A5F4CFM8\_CANLF, tr\|A0A5F4CUQ1\|A0A5F4CUQ1\_CANLF, tr\|A0A5F4CWM9\|A0A5F4CWM9\_CANLF, tr\|A0A5F4D721\|A0A5F4D721\_CANLF, tr\|A0A5F4DCE0\|A0A5F4DCE0\_CANLF, tr\|E2QXY7\|E2QXY7\_CANLF, tr\|E2RDU7\|E2RDU7\_CANLF, tr\|E2RRY2\|E2RRY2\_CANLF, tr\|E2RT60\|E2RT60\_CANLF, tr\|F1P6C3\|F1P6C3\_CANLF, tr\|F1P7G4\|F1P7G4\_CANLF, tr\|F1P8F3\|F1P8F3\_CANLF, tr\|F1PCV3\|F1PCV3\_CANLF, tr\|F1PJ01\|F1PJ01\_CANLF, tr\|F1PP33\|F1PP33\_CANLF, tr\|F1PPG5\|F1PPG5\_CANLF, tr\|F1PQ29\|F1PQ29\_CANLF, tr\|F1PVV7\|F1PVV7\_CANLF, tr\|F1PYS6\|F1PYS6\_CANLF, tr\|G1K2D5\|G1K2D5\_CANLF, tr\|J9NSW5\|J9NSW5\_CANLF, tr\|J9NVU0\|J9NVU0\_CANLF, tr\|J9P1N8\|J9P1N8\_CANLF, tr\|J9P2B9\|J9P2B9\_CANLF, tr\|J9P3Z7\|J9P3Z7\_CANLF, tr\|J9PAK3\|J9PAK3\_CANLF, tr\|Z4YHI2\|Z4YHI2\_CANLF |
| 30 | Parkinson's disease | tr\|A0A5F4BYS0\|A0A5F4BYS0\_CANLF, tr\|A0A5F4C290\|A0A5F4C290\_CANLF, tr\|A0A5F4C7T7\|A0A5F4C7T7\_CANLF, tr\|A0A5F4CJ48\|A0A5F4CJ48\_CANLF, tr\|A0A5F4CT03\|A0A5F4CT03\_CANLF, tr\|A0A5F4D0C4\|A0A5F4D0C4\_CANLF, tr\|A0A5F4D296\|A0A5F4D296\_CANLF, tr\|A0A5F4D6E6\|A0A5F4D6E6\_CANLF, tr\|A0A5F4D804\|A0A5F4D804\_CANLF, tr\|A0A5F4DJV1\|A0A5F4DJV1\_CANLF, tr\|E2QS13\|E2QS13\_CANLF, tr\|E2QUA8\|E2QUA8\_CANLF, tr\|E2QUM4\|E2QUM4\_CANLF, tr\|E2QV28\|E2QV28\_CANLF, tr\|E2R4Q6\|E2R4Q6\_CANLF, tr\|E2RD75\|E2RD75\_CANLF, tr\|E2RGH5\|E2RGH5\_CANLF, tr\|E2RHL0\|E2RHL0\_CANLF, tr\|E2RJG8\|E2RJG8\_CANLF, tr\|E2RNG2\|E2RNG2\_CANLF, tr\|F1PEK5\|F1PEK5\_CANLF, tr\|F1PG47\|F1PG47\_CANLF, tr\|F1PLL0\|F1PLL0\_CANLF, tr\|F1PLP2\|F1PLP2\_CANLF, tr\|F1PML1\|F1PML1\_CANLF, tr\|F1PRZ1\|F1PRZ1\_CANLF, tr\|F1PY24\|F1PY24\_CANLF, tr\|F1Q067\|F1Q067\_CANLF, tr\|F1Q1J6\|F1Q1J6\_CANLF, tr\|F1Q3W8\|F1Q3W8\_CANLF, tr\|F6X895\|F6X895\_CANLF, tr\|J9NZQ0\|J9NZQ0\_CANLF, tr\|V5LKV5\|V5LKV5\_CANLF |
| 31 | Arrhythmogenic right ventricular cardiomyopathy (ARVC) | sp\|O18840\|ACTB\_CANLF, sp\|Q5XFN2\|DESM\_CANLF, tr\|A0A140T8E6\|A0A140T8E6\_CANLF, tr\|A0A5F4BVB0\|A0A5F4BVB0\_CANLF, tr\|A0A5F4C3X9\|A0A5F4C3X9\_CANLF, tr\|A0A5F4C4P9\|A0A5F4C4P9\_CANLF, tr\|A0A5F4C9C0\|A0A5F4C9C0\_CANLF, tr\|A0A5F4C9R4\|A0A5F4C9R4\_CANLF, tr\|A0A5F4D0Z3\|A0A5F4D0Z3\_CANLF, tr\|A0A5F4DC86\|A0A5F4DC86\_CANLF, tr\|E2QXY7\|E2QXY7\_CANLF, tr\|E2QY08\|E2QY08\_CANLF, tr\|E2R9S7\|E2R9S7\_CANLF, tr\|E2RHY0\|E2RHY0\_CANLF, tr\|E2RNG4\|E2RNG4\_CANLF, tr\|E2RT60\|E2RT60\_CANLF, tr\|F1P6C3\|F1P6C3\_CANLF, tr\|F1P7G4\|F1P7G4\_CANLF, tr\|F1P7S2\|F1P7S2\_CANLF, tr\|F1P8F3\|F1P8F3\_CANLF, tr\|F1PGX2\|F1PGX2\_CANLF, tr\|F1PP33\|F1PP33\_CANLF, tr\|F1PPG5\|F1PPG5\_CANLF, tr\|F1PQ29\|F1PQ29\_CANLF, tr\|J9NSW5\|J9NSW5\_CANLF, tr\|J9NVU0\|J9NVU0\_CANLF, tr\|J9P2B9\|J9P2B9\_CANLF, tr\|J9P3Z7\|J9P3Z7\_CANLF, tr\|J9P7X9\|J9P7X9\_CANLF, tr\|J9PAK3\|J9PAK3\_CANLF, tr\|O77704\|O77704\_CANLF, tr\|Z4YHI2\|Z4YHI2\_CANLF |
| 32 | Purine metabolism | sp\|P30803\|ADCY5\_CANLF, tr\|A0A5F4C9Q4\|A0A5F4C9Q4\_CANLF, tr\|A0A5F4CKN4\|A0A5F4CKN4\_CANLF, tr\|A0A5F4CSH3\|A0A5F4CSH3\_CANLF, tr\|A0A5F4CVL0\|A0A5F4CVL0\_CANLF, tr\|A0A5F4CWL0\|A0A5F4CWL0\_CANLF, tr\|A0A5F4CYQ8\|A0A5F4CYQ8\_CANLF, tr\|A0A5F4D0K7\|A0A5F4D0K7\_CANLF, tr\|A0A5F4D5L1\|A0A5F4D5L1\_CANLF, tr\|A0A5F4DCC4\|A0A5F4DCC4\_CANLF, tr\|A0A5F4DKH0\|A0A5F4DKH0\_CANLF, tr\|B4YY02\|B4YY02\_CANLF, tr\|E2QV12\|E2QV12\_CANLF, tr\|E2R1E3\|E2R1E3\_CANLF, tr\|E2R3I2\|E2R3I2\_CANLF, tr\|E2R5U8\|E2R5U8\_CANLF, tr\|E2R8N8\|E2R8N8\_CANLF, tr\|E2RC20\|E2RC20\_CANLF, tr\|E2RPH0\|E2RPH0\_CANLF, tr\|F1P797\|F1P797\_CANLF, tr\|F1PBZ5\|F1PBZ5\_CANLF, tr\|F1PI77\|F1PI77\_CANLF, tr\|F1PKY3\|F1PKY3\_CANLF, tr\|F1PPT7\|F1PPT7\_CANLF, tr\|F1PQM1\|F1PQM1\_CANLF, tr\|F1PTS5\|F1PTS5\_CANLF, tr\|F1PY94\|F1PY94\_CANLF, tr\|J9JHC8\|J9JHC8\_CANLF, tr\|J9NSJ8\|J9NSJ8\_CANLF, tr\|J9NVT2\|J9NVT2\_CANLF, tr\|J9P2M3\|J9P2M3\_CANLF |
| 33 | Ribosome | sp\|E2RH47\|RS3\_CANLF, sp\|Q5TJE9\|RS18\_CANLF, tr\|A0A5F4BW72\|A0A5F4BW72\_CANLF, tr\|A0A5F4C461\|A0A5F4C461\_CANLF, tr\|A0A5F4C6J8\|A0A5F4C6J8\_CANLF, tr\|A0A5F4CKM6\|A0A5F4CKM6\_CANLF, tr\|A0A5F4CLV3\|A0A5F4CLV3\_CANLF, tr\|A0A5F4CTI9\|A0A5F4CTI9\_CANLF, tr\|A0A5F4D1V8\|A0A5F4D1V8\_CANLF, tr\|A0A5F4D9G3\|A0A5F4D9G3\_CANLF, tr\|E2QUE7\|E2QUE7\_CANLF, tr\|E2R0A6\|E2R0A6\_CANLF, tr\|E2R4F5\|E2R4F5\_CANLF, tr\|E2R9Y9\|E2R9Y9\_CANLF, tr\|E2RCL4\|E2RCL4\_CANLF, tr\|E2RIA1\|E2RIA1\_CANLF, tr\|E2RIQ8\|E2RIQ8\_CANLF, tr\|E2RP05\|E2RP05\_CANLF, tr\|F1PEZ4\|F1PEZ4\_CANLF, tr\|F1PLN2\|F1PLN2\_CANLF, tr\|F1Q424\|F1Q424\_CANLF, tr\|F2Z4N1\|F2Z4N1\_CANLF, tr\|F2Z4Q5\|F2Z4Q5\_CANLF, tr\|J9JHJ2\|J9JHJ2\_CANLF, tr\|J9NRY1\|J9NRY1\_CANLF, tr\|J9NS00\|J9NS00\_CANLF, tr\|J9P425\|J9P425\_CANLF, tr\|J9P5Q4\|J9P5Q4\_CANLF, tr\|J9P5V0\|J9P5V0\_CANLF, tr\|J9P9Q2\|J9P9Q2\_CANLF, tr\|J9PAG2\|J9PAG2\_CANLF |
| 34 | Cell adhesion molecules (CAMs) | sp\|F1PAA9\|CADH1\_CANLF, sp\|Q95KM5\|CLD3\_CANLF, tr\|A0A5F4BUG1\|A0A5F4BUG1\_CANLF, tr\|A0A5F4BYB4\|A0A5F4BYB4\_CANLF, tr\|A0A5F4C9R4\|A0A5F4C9R4\_CANLF, tr\|A0A5F4CZG6\|A0A5F4CZG6\_CANLF, tr\|A0A5F4DCC6\|A0A5F4DCC6\_CANLF, tr\|A0A5F4DJH6\|A0A5F4DJH6\_CANLF, tr\|C0IP13\|C0IP13\_CANLF, tr\|E2QZQ6\|E2QZQ6\_CANLF, tr\|E2R049\|E2R049\_CANLF, tr\|E2R9N5\|E2R9N5\_CANLF, tr\|E2RE03\|E2RE03\_CANLF, tr\|E2RT60\|E2RT60\_CANLF, tr\|F1PB95\|F1PB95\_CANLF, tr\|F1PDR0\|F1PDR0\_CANLF, tr\|F1PP71\|F1PP71\_CANLF, tr\|F1PQ29\|F1PQ29\_CANLF, tr\|F1PYL8\|F1PYL8\_CANLF, tr\|F1Q1S4\|F1Q1S4\_CANLF, tr\|F6V9R9\|F6V9R9\_CANLF, tr\|J9NSZ7\|J9NSZ7\_CANLF, tr\|J9NVU0\|J9NVU0\_CANLF, tr\|J9P296\|J9P296\_CANLF, tr\|J9P2T1\|J9P2T1\_CANLF, tr\|J9P884\|J9P884\_CANLF, tr\|O46880\|O46880\_CANLF, tr\|O46882\|O46882\_CANLF, tr\|Q30348\|Q30348\_CANLF, tr\|Q3HR31\|Q3HR31\_CANLF, tr\|Q9N0W3\|Q9N0W3\_CANLF |
| 35 | Viral myocarditis | sp\|O18840\|ACTB\_CANLF, sp\|P33724\|CAV1\_CANLF, tr\|A0A5F4BUG1\|A0A5F4BUG1\_CANLF, tr\|A0A5F4C3W6\|A0A5F4C3W6\_CANLF, tr\|A0A5F4CBH6\|A0A5F4CBH6\_CANLF, tr\|A0A5F4CE07\|A0A5F4CE07\_CANLF, tr\|A0A5F4CWM9\|A0A5F4CWM9\_CANLF, tr\|A0A5F4D211\|A0A5F4D211\_CANLF, tr\|A0A5F4D713\|A0A5F4D713\_CANLF, tr\|E2QXY7\|E2QXY7\_CANLF, tr\|E2R334\|E2R334\_CANLF, tr\|E2RC09\|E2RC09\_CANLF, tr\|E2RCC8\|E2RCC8\_CANLF, tr\|E2RE31\|E2RE31\_CANLF, tr\|F1P6C3\|F1P6C3\_CANLF, tr\|F1P7G4\|F1P7G4\_CANLF, tr\|F1P8F3\|F1P8F3\_CANLF, tr\|F1PB95\|F1PB95\_CANLF, tr\|F1PEX7\|F1PEX7\_CANLF, tr\|F1PJ01\|F1PJ01\_CANLF, tr\|F1PVV7\|F1PVV7\_CANLF, tr\|F1PYQ1\|F1PYQ1\_CANLF, tr\|F1PYS6\|F1PYS6\_CANLF, tr\|J9P357\|J9P357\_CANLF, tr\|J9PAK3\|J9PAK3\_CANLF, tr\|O46880\|O46880\_CANLF, tr\|O46882\|O46882\_CANLF, tr\|Q30348\|Q30348\_CANLF, tr\|Z4YHI2\|Z4YHI2\_CANLF |
| 36 | Hepatitis C | sp\|Q95KM5\|CLD3\_CANLF, tr\|A0A5F4BYB4\|A0A5F4BYB4\_CANLF, tr\|A0A5F4C4S3\|A0A5F4C4S3\_CANLF, tr\|A0A5F4C566\|A0A5F4C566\_CANLF, tr\|A0A5F4C5E5\|A0A5F4C5E5\_CANLF, tr\|A0A5F4CC58\|A0A5F4CC58\_CANLF, tr\|A0A5F4CDU8\|A0A5F4CDU8\_CANLF, tr\|A5H028\|A5H028\_CANLF, tr\|E2R1J6\|E2R1J6\_CANLF, tr\|E2R3V7\|E2R3V7\_CANLF, tr\|E2RI99\|E2RI99\_CANLF, tr\|E2RL17\|E2RL17\_CANLF, tr\|E2RM84\|E2RM84\_CANLF, tr\|E2RMV6\|E2RMV6\_CANLF, tr\|E2RRA9\|E2RRA9\_CANLF, tr\|F1P874\|F1P874\_CANLF, tr\|F1PB66\|F1PB66\_CANLF, tr\|F1PF03\|F1PF03\_CANLF, tr\|F1PQ60\|F1PQ60\_CANLF, tr\|F1PX75\|F1PX75\_CANLF, tr\|J9P0L7\|J9P0L7\_CANLF, tr\|J9P0U9\|J9P0U9\_CANLF, tr\|J9P1X5\|J9P1X5\_CANLF, tr\|J9P296\|J9P296\_CANLF, tr\|J9P4L9\|J9P4L9\_CANLF, tr\|Q2TJA4\|Q2TJA4\_CANLF, tr\|Q45KI9\|Q45KI9\_CANLF, tr\|Q9N0W3\|Q9N0W3\_CANLF |
| 37 | Gastric acid secretion | sp\|O18840\|ACTB\_CANLF, sp\|P30803\|ADCY5\_CANLF, sp\|P38400\|GNAI2\_CANLF, sp\|P63091\|GNAS\_CANLF, tr\|A0A5F4BVY4\|A0A5F4BVY4\_CANLF, tr\|A0A5F4CJ79\|A0A5F4CJ79\_CANLF, tr\|A0A5F4CNW6\|A0A5F4CNW6\_CANLF, tr\|A0A5F4D507\|A0A5F4D507\_CANLF, tr\|E2QXY7\|E2QXY7\_CANLF, tr\|E2RG56\|E2RG56\_CANLF, tr\|E2RHJ8\|E2RHJ8\_CANLF, tr\|E2RSI6\|E2RSI6\_CANLF, tr\|F1PBA1\|F1PBA1\_CANLF, tr\|F1PDY8\|F1PDY8\_CANLF, tr\|F1PID1\|F1PID1\_CANLF, tr\|F1PIF6\|F1PIF6\_CANLF, tr\|F1PKU7\|F1PKU7\_CANLF, tr\|F1PW86\|F1PW86\_CANLF, tr\|F1PXM5\|F1PXM5\_CANLF, tr\|F1Q1D5\|F1Q1D5\_CANLF, tr\|G1K2D5\|G1K2D5\_CANLF, tr\|J9NWJ7\|J9NWJ7\_CANLF, tr\|J9NXT4\|J9NXT4\_CANLF, tr\|J9P2M3\|J9P2M3\_CANLF, tr\|J9P8S3\|J9P8S3\_CANLF, tr\|J9P9H3\|J9P9H3\_CANLF, tr\|J9PAK3\|J9PAK3\_CANLF, tr\|Z4YHI2\|Z4YHI2\_CANLF |
| 38 | Shigellosis | sp\|O18840\|ACTB\_CANLF, tr\|A0A5F4BQZ0\|A0A5F4BQZ0\_CANLF, tr\|A0A5F4C5E5\|A0A5F4C5E5\_CANLF, tr\|A0A5F4CBU4\|A0A5F4CBU4\_CANLF, tr\|A0A5F4CIB4\|A0A5F4CIB4\_CANLF, tr\|A0A5F4CWM0\|A0A5F4CWM0\_CANLF, tr\|A0A5F4D5V0\|A0A5F4D5V0\_CANLF, tr\|E2QWD3\|E2QWD3\_CANLF, tr\|E2QWU0\|E2QWU0\_CANLF, tr\|E2QXY7\|E2QXY7\_CANLF, tr\|E2R6A0\|E2R6A0\_CANLF, tr\|E2RF98\|E2RF98\_CANLF, tr\|E2RI72\|E2RI72\_CANLF, tr\|E2RMT4\|E2RMT4\_CANLF, tr\|E2RT60\|E2RT60\_CANLF, tr\|F1P803\|F1P803\_CANLF, tr\|F1PAG6\|F1PAG6\_CANLF, tr\|F1PEX7\|F1PEX7\_CANLF, tr\|F1PIC2\|F1PIC2\_CANLF, tr\|F1PKR3\|F1PKR3\_CANLF, tr\|F1PUS0\|F1PUS0\_CANLF, tr\|F1PYQ1\|F1PYQ1\_CANLF, tr\|F1Q3Y0\|F1Q3Y0\_CANLF, tr\|F1Q4I3\|F1Q4I3\_CANLF, tr\|H9GW54\|H9GW54\_CANLF, tr\|J9P423\|J9P423\_CANLF, tr\|J9PAK3\|J9PAK3\_CANLF, tr\|Z4YHI2\|Z4YHI2\_CANLF |
| 39 | Oocyte meiosis | sp\|P30803\|ADCY5\_CANLF, tr\|A0A5F4BVY6\|A0A5F4BVY6\_CANLF, tr\|A0A5F4C5E5\|A0A5F4C5E5\_CANLF, tr\|A0A5F4CJ79\|A0A5F4CJ79\_CANLF, tr\|A0A5F4CNW6\|A0A5F4CNW6\_CANLF, tr\|A0A5F4CT62\|A0A5F4CT62\_CANLF, tr\|A0A5F4CTR8\|A0A5F4CTR8\_CANLF, tr\|A0A5F4CXJ9\|A0A5F4CXJ9\_CANLF, tr\|E2QV07\|E2QV07\_CANLF, tr\|E2QY29\|E2QY29\_CANLF, tr\|E2R7T4\|E2R7T4\_CANLF, tr\|E2RG56\|E2RG56\_CANLF, tr\|E2RRW0\|E2RRW0\_CANLF, tr\|F1P9J9\|F1P9J9\_CANLF, tr\|F1PBL1\|F1PBL1\_CANLF, tr\|F1PKW7\|F1PKW7\_CANLF, tr\|F1PTH2\|F1PTH2\_CANLF, tr\|F1PW86\|F1PW86\_CANLF, tr\|F1PX75\|F1PX75\_CANLF, tr\|F2Z4P5\|F2Z4P5\_CANLF, tr\|G1K2D5\|G1K2D5\_CANLF, tr\|J9NRH5\|J9NRH5\_CANLF, tr\|J9NWJ7\|J9NWJ7\_CANLF, tr\|J9NXT4\|J9NXT4\_CANLF, tr\|J9P2M3\|J9P2M3\_CANLF, tr\|J9P6N4\|J9P6N4\_CANLF, tr\|J9P8S3\|J9P8S3\_CANLF, tr\|J9PAA1\|J9PAA1\_CANLF |
| 40 | Ubiquitin mediated proteolysis | tr\|A0A5F4BXH1\|A0A5F4BXH1\_CANLF, tr\|A0A5F4C0N2\|A0A5F4C0N2\_CANLF, tr\|A0A5F4C5D6\|A0A5F4C5D6\_CANLF, tr\|A0A5F4C7T7\|A0A5F4C7T7\_CANLF, tr\|A0A5F4CGL9\|A0A5F4CGL9\_CANLF, tr\|A0A5F4CUL0\|A0A5F4CUL0\_CANLF, tr\|A0A5F4D062\|A0A5F4D062\_CANLF, tr\|A0A5F4D2S8\|A0A5F4D2S8\_CANLF, tr\|A0A5F4D937\|A0A5F4D937\_CANLF, tr\|A0A5F4DGX8\|A0A5F4DGX8\_CANLF, tr\|D9ILX1\|D9ILX1\_CANLF, tr\|E2QXM7\|E2QXM7\_CANLF, tr\|E2QYQ5\|E2QYQ5\_CANLF, tr\|E2R3R3\|E2R3R3\_CANLF, tr\|E2R4Q6\|E2R4Q6\_CANLF, tr\|E2R680\|E2R680\_CANLF, tr\|E2R6W9\|E2R6W9\_CANLF, tr\|E2RGH5\|E2RGH5\_CANLF, tr\|E2RKS8\|E2RKS8\_CANLF, tr\|E2RRW0\|E2RRW0\_CANLF, tr\|F1P9J9\|F1P9J9\_CANLF, tr\|F1PG13\|F1PG13\_CANLF, tr\|F1PQ60\|F1PQ60\_CANLF, tr\|F1PTH2\|F1PTH2\_CANLF, tr\|F1PYW3\|F1PYW3\_CANLF, tr\|F1Q2Y7\|F1Q2Y7\_CANLF, tr\|J9NRQ4\|J9NRQ4\_CANLF, tr\|J9NZR2\|J9NZR2\_CANLF |
| 41 | ErbB signaling pathway | sp\|Q1HG70\|MP2K2\_CANLF, tr\|A0A5F4C4S3\|A0A5F4C4S3\_CANLF, tr\|A0A5F4C566\|A0A5F4C566\_CANLF, tr\|A0A5F4C5E5\|A0A5F4C5E5\_CANLF, tr\|A0A5F4CJ79\|A0A5F4CJ79\_CANLF, tr\|A0A5F4DF90\|A0A5F4DF90\_CANLF, tr\|E2QW49\|E2QW49\_CANLF, tr\|E2QWD3\|E2QWD3\_CANLF, tr\|E2REH3\|E2REH3\_CANLF, tr\|E2RF98\|E2RF98\_CANLF, tr\|E2RG56\|E2RG56\_CANLF, tr\|E2RHV6\|E2RHV6\_CANLF, tr\|E2RPA3\|E2RPA3\_CANLF, tr\|E2RPY6\|E2RPY6\_CANLF, tr\|E2RRA9\|E2RRA9\_CANLF, tr\|F1P874\|F1P874\_CANLF, tr\|F1PEX7\|F1PEX7\_CANLF, tr\|F1PF03\|F1PF03\_CANLF, tr\|F1PKU7\|F1PKU7\_CANLF, tr\|F1PNW8\|F1PNW8\_CANLF, tr\|F1PQC4\|F1PQC4\_CANLF, tr\|F1PVC5\|F1PVC5\_CANLF, tr\|F1Q1D5\|F1Q1D5\_CANLF, tr\|J9P1X5\|J9P1X5\_CANLF, tr\|J9P4L9\|J9P4L9\_CANLF, tr\|Q45KI9\|Q45KI9\_CANLF, tr\|Q68V02\|Q68V02\_CANLF |
| 42 | Phosphatidylinositol signaling system | tr\|A0A5F4BQ52\|A0A5F4BQ52\_CANLF, tr\|A0A5F4BVY4\|A0A5F4BVY4\_CANLF, tr\|A0A5F4CNW6\|A0A5F4CNW6\_CANLF, tr\|A0A5F4CT67\|A0A5F4CT67\_CANLF, tr\|A0A5F4D6R1\|A0A5F4D6R1\_CANLF, tr\|A0A5F4D8L2\|A0A5F4D8L2\_CANLF, tr\|E2R185\|E2R185\_CANLF, tr\|E2R9U0\|E2R9U0\_CANLF, tr\|E2RRA9\|E2RRA9\_CANLF, tr\|E2RSF7\|E2RSF7\_CANLF, tr\|E2RSJ5\|E2RSJ5\_CANLF, tr\|F1PKR8\|F1PKR8\_CANLF, tr\|F1PKU7\|F1PKU7\_CANLF, tr\|F1PMC5\|F1PMC5\_CANLF, tr\|F1PPB7\|F1PPB7\_CANLF, tr\|F1PW86\|F1PW86\_CANLF, tr\|F1PWW8\|F1PWW8\_CANLF, tr\|F1Q1D5\|F1Q1D5\_CANLF, tr\|F1Q2H0\|F1Q2H0\_CANLF, tr\|G1K2D5\|G1K2D5\_CANLF, tr\|J9JHK8\|J9JHK8\_CANLF, tr\|J9NWJ7\|J9NWJ7\_CANLF, tr\|J9P1X5\|J9P1X5\_CANLF, tr\|J9P2C8\|J9P2C8\_CANLF, tr\|J9P6E1\|J9P6E1\_CANLF, tr\|J9P6F5\|J9P6F5\_CANLF, tr\|J9P8S3\|J9P8S3\_CANLF |
| 43 | GnRH signaling pathway | sp\|P30803\|ADCY5\_CANLF, sp\|P63091\|GNAS\_CANLF, sp\|Q1HG70\|MP2K2\_CANLF, tr\|A0A5F4BVY4\|A0A5F4BVY4\_CANLF, tr\|A0A5F4C4S3\|A0A5F4C4S3\_CANLF, tr\|A0A5F4C566\|A0A5F4C566\_CANLF, tr\|A0A5F4C5E5\|A0A5F4C5E5\_CANLF, tr\|A0A5F4CJ79\|A0A5F4CJ79\_CANLF, tr\|A0A5F4CNW6\|A0A5F4CNW6\_CANLF, tr\|A0A5F4D5V0\|A0A5F4D5V0\_CANLF, tr\|E2RG56\|E2RG56\_CANLF, tr\|E2RGU7\|E2RGU7\_CANLF, tr\|E2RNP2\|E2RNP2\_CANLF, tr\|F1P750\|F1P750\_CANLF, tr\|F1PDX2\|F1PDX2\_CANLF, tr\|F1PF03\|F1PF03\_CANLF, tr\|F1PKU7\|F1PKU7\_CANLF, tr\|F1PW86\|F1PW86\_CANLF, tr\|F1PXM5\|F1PXM5\_CANLF, tr\|F1Q1D5\|F1Q1D5\_CANLF, tr\|F6UQ33\|F6UQ33\_CANLF, tr\|G1K2D5\|G1K2D5\_CANLF, tr\|J9NWJ7\|J9NWJ7\_CANLF, tr\|J9NXT4\|J9NXT4\_CANLF, tr\|J9P2M3\|J9P2M3\_CANLF, tr\|J9P4L9\|J9P4L9\_CANLF, tr\|J9P8S3\|J9P8S3\_CANLF |
| 44 | Gap junction | sp\|O97758\|ZO1\_CANLF, sp\|P30803\|ADCY5\_CANLF, sp\|P38400\|GNAI2\_CANLF, sp\|P63091\|GNAS\_CANLF, sp\|Q1HG70\|MP2K2\_CANLF, tr\|A0A5F4BVY4\|A0A5F4BVY4\_CANLF, tr\|A0A5F4C4S3\|A0A5F4C4S3\_CANLF, tr\|A0A5F4C566\|A0A5F4C566\_CANLF, tr\|A0A5F4C5E5\|A0A5F4C5E5\_CANLF, tr\|A0A5F4CNW6\|A0A5F4CNW6\_CANLF, tr\|E2QSF4\|E2QSF4\_CANLF, tr\|E2QY29\|E2QY29\_CANLF, tr\|E2RBC3\|E2RBC3\_CANLF, tr\|E2RNQ2\|E2RNQ2\_CANLF, tr\|F1PF03\|F1PF03\_CANLF, tr\|F1PI77\|F1PI77\_CANLF, tr\|F1PKU7\|F1PKU7\_CANLF, tr\|F1PW86\|F1PW86\_CANLF, tr\|F1PXM5\|F1PXM5\_CANLF, tr\|F1Q1D5\|F1Q1D5\_CANLF, tr\|J9NXT4\|J9NXT4\_CANLF, tr\|J9P2M3\|J9P2M3\_CANLF, tr\|J9P4L9\|J9P4L9\_CANLF, tr\|J9P7X9\|J9P7X9\_CANLF, tr\|L7N0B2\|L7N0B2\_CANLF, tr\|L7N0I7\|L7N0I7\_CANLF |
| 45 | Toxoplasmosis | sp\|P38400\|GNAI2\_CANLF, sp\|Q5ECR9\|CCR5\_CANLF, tr\|A0A5F4BRD0\|A0A5F4BRD0\_CANLF, tr\|A0A5F4BUG1\|A0A5F4BUG1\_CANLF, tr\|A0A5F4C4G4\|A0A5F4C4G4\_CANLF, tr\|A0A5F4C5E5\|A0A5F4C5E5\_CANLF, tr\|A0A5F4CC58\|A0A5F4CC58\_CANLF, tr\|A0A5F4CJ74\|A0A5F4CJ74\_CANLF, tr\|A0A5F4DCI2\|A0A5F4DCI2\_CANLF, tr\|E2R3R9\|E2R3R9\_CANLF, tr\|E2R4J1\|E2R4J1\_CANLF, tr\|E2RI99\|E2RI99\_CANLF, tr\|E2RM84\|E2RM84\_CANLF, tr\|E2RRA9\|E2RRA9\_CANLF, tr\|E2RT60\|E2RT60\_CANLF, tr\|F1PR66\|F1PR66\_CANLF, tr\|F6UQ33\|F6UQ33\_CANLF, tr\|G1K268\|G1K268\_CANLF, tr\|H9GW49\|H9GW49\_CANLF, tr\|J9NVU0\|J9NVU0\_CANLF, tr\|J9P1X5\|J9P1X5\_CANLF, tr\|J9P614\|J9P614\_CANLF, tr\|Q30348\|Q30348\_CANLF, tr\|Q45KI9\|Q45KI9\_CANLF, tr\|Q867A1\|Q867A1\_CANLF, tr\|Q867A2\|Q867A2\_CANLF |
| 46 | Systemic lupus erythematosus | tr\|A0A5F4BU46\|A0A5F4BU46\_CANLF, tr\|A0A5F4BUG1\|A0A5F4BUG1\_CANLF, tr\|A0A5F4C3X9\|A0A5F4C3X9\_CANLF, tr\|A0A5F4CBH6\|A0A5F4CBH6\_CANLF, tr\|A0A5F4DC86\|A0A5F4DC86\_CANLF, tr\|E2QW27\|E2QW27\_CANLF, tr\|E2QY08\|E2QY08\_CANLF, tr\|E2RCC8\|E2RCC8\_CANLF, tr\|E2RCZ7\|E2RCZ7\_CANLF, tr\|E2RDF9\|E2RDF9\_CANLF, tr\|E2RE31\|E2RE31\_CANLF, tr\|E2RH09\|E2RH09\_CANLF, tr\|E2RQX0\|E2RQX0\_CANLF, tr\|F1P7S2\|F1P7S2\_CANLF, tr\|F1PGM1\|F1PGM1\_CANLF, tr\|F1PHT0\|F1PHT0\_CANLF, tr\|G1K2D9\|G1K2D9\_CANLF, tr\|J9NRV0\|J9NRV0\_CANLF, tr\|J9P0L5\|J9P0L5\_CANLF, tr\|J9P3D2\|J9P3D2\_CANLF, tr\|J9P6M9\|J9P6M9\_CANLF, tr\|J9P6S8\|J9P6S8\_CANLF, tr\|J9PB22\|J9PB22\_CANLF, tr\|Q30348\|Q30348\_CANLF, tr\|Q8MJD1\|Q8MJD1\_CANLF |
| 47 | Wnt signaling pathway | sp\|A0M8U6\|TES\_CANLF, tr\|A0A5F4BVY4\|A0A5F4BVY4\_CANLF, tr\|A0A5F4CFW2\|A0A5F4CFW2\_CANLF, tr\|A0A5F4CJ79\|A0A5F4CJ79\_CANLF, tr\|A0A5F4D0Z3\|A0A5F4D0Z3\_CANLF, tr\|A0A5K1UA11\|A0A5K1UA11\_CANLF, tr\|E2QVG1\|E2QVG1\_CANLF, tr\|E2R3R9\|E2R3R9\_CANLF, tr\|E2RDQ5\|E2RDQ5\_CANLF, tr\|E2RG56\|E2RG56\_CANLF, tr\|E2RRW0\|E2RRW0\_CANLF, tr\|F1PDW7\|F1PDW7\_CANLF, tr\|F1PKU7\|F1PKU7\_CANLF, tr\|F1PKX6\|F1PKX6\_CANLF, tr\|F1PVP5\|F1PVP5\_CANLF, tr\|F1PWF5\|F1PWF5\_CANLF, tr\|F1PX75\|F1PX75\_CANLF, tr\|F1PYQ1\|F1PYQ1\_CANLF, tr\|F1Q1D5\|F1Q1D5\_CANLF, tr\|F1Q4I3\|F1Q4I3\_CANLF, tr\|F2Z4P5\|F2Z4P5\_CANLF, tr\|F6XY66\|F6XY66\_CANLF, tr\|J9NXT4\|J9NXT4\_CANLF, tr\|J9P4U8\|J9P4U8\_CANLF, tr\|J9P7Q4\|J9P7Q4\_CANLF |
| 48 | Small cell lung cancer | tr\|A0A5F4BVB0\|A0A5F4BVB0\_CANLF, tr\|A0A5F4C4G4\|A0A5F4C4G4\_CANLF, tr\|A0A5F4C9R4\|A0A5F4C9R4\_CANLF, tr\|A0A5F4CB93\|A0A5F4CB93\_CANLF, tr\|A0A5F4CJ74\|A0A5F4CJ74\_CANLF, tr\|A0A5F4CT67\|A0A5F4CT67\_CANLF, tr\|A0A5F4D5S9\|A0A5F4D5S9\_CANLF, tr\|A0A5F4D6R1\|A0A5F4D6R1\_CANLF, tr\|A0A5F4DCI2\|A0A5F4DCI2\_CANLF, tr\|E2R4J1\|E2R4J1\_CANLF, tr\|E2RJG8\|E2RJG8\_CANLF, tr\|E2RRA9\|E2RRA9\_CANLF, tr\|E2RT60\|E2RT60\_CANLF, tr\|F1PKR8\|F1PKR8\_CANLF, tr\|F1PQ60\|F1PQ60\_CANLF, tr\|F1PR66\|F1PR66\_CANLF, tr\|F1Q133\|F1Q133\_CANLF, tr\|J9NVU0\|J9NVU0\_CANLF, tr\|J9P1X5\|J9P1X5\_CANLF, tr\|J9P614\|J9P614\_CANLF, tr\|Q28272\|Q28272\_CANLF, tr\|Q597P9\|Q597P9\_CANLF, tr\|Q68V02\|Q68V02\_CANLF, tr\|Q867A1\|Q867A1\_CANLF, tr\|Q867A2\|Q867A2\_CANLF |
| 49 | Pancreatic secretion | sp\|P30803\|ADCY5\_CANLF, sp\|P63091\|GNAS\_CANLF, tr\|A0A5F4BVY4\|A0A5F4BVY4\_CANLF, tr\|A0A5F4C9C0\|A0A5F4C9C0\_CANLF, tr\|A0A5F4CNW6\|A0A5F4CNW6\_CANLF, tr\|A0A5F4CSQ5\|A0A5F4CSQ5\_CANLF, tr\|A0A5F4D507\|A0A5F4D507\_CANLF, tr\|E2R2K4\|E2R2K4\_CANLF, tr\|F1PBA1\|F1PBA1\_CANLF, tr\|F1PCE8\|F1PCE8\_CANLF, tr\|F1PDY8\|F1PDY8\_CANLF, tr\|F1PIB8\|F1PIB8\_CANLF, tr\|F1PID1\|F1PID1\_CANLF, tr\|F1PKU7\|F1PKU7\_CANLF, tr\|F1PW86\|F1PW86\_CANLF, tr\|F1PXM5\|F1PXM5\_CANLF, tr\|F1PYQ1\|F1PYQ1\_CANLF, tr\|F1Q1D5\|F1Q1D5\_CANLF, tr\|F6UQ33\|F6UQ33\_CANLF, tr\|F6XY66\|F6XY66\_CANLF, tr\|J9JHA5\|J9JHA5\_CANLF, tr\|J9P2B9\|J9P2B9\_CANLF, tr\|J9P2M3\|J9P2M3\_CANLF, tr\|J9P8P9\|J9P8P9\_CANLF, tr\|J9P988\|J9P988\_CANLF |
| 50 | Prostate cancer | sp\|P41148\|ENPL\_CANLF, sp\|Q1HG70\|MP2K2\_CANLF, tr\|A0A5F4BSL9\|A0A5F4BSL9\_CANLF, tr\|A0A5F4C4S3\|A0A5F4C4S3\_CANLF, tr\|A0A5F4C566\|A0A5F4C566\_CANLF, tr\|A0A5F4C5E5\|A0A5F4C5E5\_CANLF, tr\|A0A5F4CNR7\|A0A5F4CNR7\_CANLF, tr\|A0A5F4CT67\|A0A5F4CT67\_CANLF, tr\|A0A5F4D0Z3\|A0A5F4D0Z3\_CANLF, tr\|A0A5F4D6M3\|A0A5F4D6M3\_CANLF, tr\|A0A5F4D6R1\|A0A5F4D6R1\_CANLF, tr\|E2RLS3\|E2RLS3\_CANLF, tr\|E2RRA9\|E2RRA9\_CANLF, tr\|F1P874\|F1P874\_CANLF, tr\|F1PF03\|F1PF03\_CANLF, tr\|F1PGY1\|F1PGY1\_CANLF, tr\|F1PKR8\|F1PKR8\_CANLF, tr\|F1PYJ0\|F1PYJ0\_CANLF, tr\|F1Q0J0\|F1Q0J0\_CANLF, tr\|F1Q2Y3\|F1Q2Y3\_CANLF, tr\|J9P1X5\|J9P1X5\_CANLF, tr\|J9P4L9\|J9P4L9\_CANLF, tr\|Q45KI9\|Q45KI9\_CANLF, tr\|Q68V02\|Q68V02\_CANLF |
| 51 | Glycolysis / Gluconeogenesis | tr\|A0A346M2B2\|A0A346M2B2\_CANLF, tr\|A0A5F4C4W3\|A0A5F4C4W3\_CANLF, tr\|A0A5F4CVL0\|A0A5F4CVL0\_CANLF, tr\|A0A5F4CZB6\|A0A5F4CZB6\_CANLF, tr\|A0A5F4DCR1\|A0A5F4DCR1\_CANLF, tr\|A0A5F4DKH0\|A0A5F4DKH0\_CANLF, tr\|E2QV12\|E2QV12\_CANLF, tr\|E2R2C3\|E2R2C3\_CANLF, tr\|E2RQ99\|E2RQ99\_CANLF, tr\|E2RRC9\|E2RRC9\_CANLF, tr\|E2RT65\|E2RT65\_CANLF, tr\|F1PAB7\|F1PAB7\_CANLF, tr\|F1PBZ5\|F1PBZ5\_CANLF, tr\|F1PK43\|F1PK43\_CANLF, tr\|F1PPF7\|F1PPF7\_CANLF, tr\|F1PVW0\|F1PVW0\_CANLF, tr\|F1PXN6\|F1PXN6\_CANLF, tr\|F1Q3S9\|F1Q3S9\_CANLF, tr\|J9P208\|J9P208\_CANLF, tr\|J9P3N9\|J9P3N9\_CANLF, tr\|J9P7A6\|J9P7A6\_CANLF, tr\|J9P7N4\|J9P7N4\_CANLF, tr\|L7N0H7\|L7N0H7\_CANLF, tr\|Q28226\|Q28226\_CANLF |
| 52 | T cell receptor signaling pathway | sp\|Q1HG70\|MP2K2\_CANLF, tr\|A0A5F4C4S3\|A0A5F4C4S3\_CANLF, tr\|A0A5F4C566\|A0A5F4C566\_CANLF, tr\|A0A5F4C5E5\|A0A5F4C5E5\_CANLF, tr\|A0A5F4CYL9\|A0A5F4CYL9\_CANLF, tr\|A0A5F4D5V0\|A0A5F4D5V0\_CANLF, tr\|A0A5F4DF90\|A0A5F4DF90\_CANLF, tr\|E2R3R9\|E2R3R9\_CANLF, tr\|E2RA73\|E2RA73\_CANLF, tr\|E2RC09\|E2RC09\_CANLF, tr\|E2RCV5\|E2RCV5\_CANLF, tr\|E2REH3\|E2REH3\_CANLF, tr\|E2RGU7\|E2RGU7\_CANLF, tr\|E2RHV6\|E2RHV6\_CANLF, tr\|E2RNP2\|E2RNP2\_CANLF, tr\|E2RPY6\|E2RPY6\_CANLF, tr\|E2RRA9\|E2RRA9\_CANLF, tr\|F1PCU2\|F1PCU2\_CANLF, tr\|F1PDX2\|F1PDX2\_CANLF, tr\|F1Q1S4\|F1Q1S4\_CANLF, tr\|F2Z4P5\|F2Z4P5\_CANLF, tr\|F6XY66\|F6XY66\_CANLF, tr\|J9P1X5\|J9P1X5\_CANLF, tr\|J9P4L9\|J9P4L9\_CANLF |
| 53 | Cell cycle | tr\|A0A5F4BVY6\|A0A5F4BVY6\_CANLF, tr\|A0A5F4CT62\|A0A5F4CT62\_CANLF, tr\|A0A5F4CTR8\|A0A5F4CTR8\_CANLF, tr\|A0A5F4DJ19\|A0A5F4DJ19\_CANLF, tr\|E2QRU9\|E2QRU9\_CANLF, tr\|E2QV07\|E2QV07\_CANLF, tr\|E2QY29\|E2QY29\_CANLF, tr\|E2R7S5\|E2R7S5\_CANLF, tr\|E2R7T4\|E2R7T4\_CANLF, tr\|E2RIG3\|E2RIG3\_CANLF, tr\|E2RKS8\|E2RKS8\_CANLF, tr\|E2RRW0\|E2RRW0\_CANLF, tr\|F1P9J9\|F1P9J9\_CANLF, tr\|F1PBL1\|F1PBL1\_CANLF, tr\|F1PEX7\|F1PEX7\_CANLF, tr\|F1PKW7\|F1PKW7\_CANLF, tr\|F1PR63\|F1PR63\_CANLF, tr\|F1PTH2\|F1PTH2\_CANLF, tr\|J9NRH5\|J9NRH5\_CANLF, tr\|J9P6N4\|J9P6N4\_CANLF, tr\|J9P937\|J9P937\_CANLF, tr\|J9PAA1\|J9PAA1\_CANLF, tr\|Q68V02\|Q68V02\_CANLF |
| 54 | Lysosome | sp\|P17129\|PSPB\_CANLF, sp\|Q4LAL9\|CATD\_CANLF, sp\|Q9GL24\|CATL1\_CANLF, tr\|A0A5F4DI81\|A0A5F4DI81\_CANLF, tr\|A0A5F4DIW1\|A0A5F4DIW1\_CANLF, tr\|E2QXJ5\|E2QXJ5\_CANLF, tr\|E2R4C1\|E2R4C1\_CANLF, tr\|E2RLF1\|E2RLF1\_CANLF, tr\|E2RNP9\|E2RNP9\_CANLF, tr\|E2RPG3\|E2RPG3\_CANLF, tr\|E2RPI9\|E2RPI9\_CANLF, tr\|E2RRJ6\|E2RRJ6\_CANLF, tr\|F1P6L7\|F1P6L7\_CANLF, tr\|F1P6N4\|F1P6N4\_CANLF, tr\|F1PHQ0\|F1PHQ0\_CANLF, tr\|F1PRH5\|F1PRH5\_CANLF, tr\|F1PWA9\|F1PWA9\_CANLF, tr\|F1PWH9\|F1PWH9\_CANLF, tr\|F1Q260\|F1Q260\_CANLF, tr\|F6X5M3\|F6X5M3\_CANLF, tr\|F6X9C1\|F6X9C1\_CANLF, tr\|J9P1I0\|J9P1I0\_CANLF, tr\|K0J6C5\|K0J6C5\_CANLF |
| 55 | Natural killer cell mediated cytotoxicity | sp\|Q1HG70\|MP2K2\_CANLF, tr\|A0A5F4C4S3\|A0A5F4C4S3\_CANLF, tr\|A0A5F4C566\|A0A5F4C566\_CANLF, tr\|A0A5F4C5E5\|A0A5F4C5E5\_CANLF, tr\|A0A5F4CBH6\|A0A5F4CBH6\_CANLF, tr\|A0A5F4CE07\|A0A5F4CE07\_CANLF, tr\|E2RC09\|E2RC09\_CANLF, tr\|E2RCC8\|E2RCC8\_CANLF, tr\|E2RE31\|E2RE31\_CANLF, tr\|E2REH3\|E2REH3\_CANLF, tr\|E2RRA9\|E2RRA9\_CANLF, tr\|F1P874\|F1P874\_CANLF, tr\|F1PB95\|F1PB95\_CANLF, tr\|F1PKU7\|F1PKU7\_CANLF, tr\|F1PQC4\|F1PQC4\_CANLF, tr\|F1PYQ1\|F1PYQ1\_CANLF, tr\|F1Q1D5\|F1Q1D5\_CANLF, tr\|F2Z4P5\|F2Z4P5\_CANLF, tr\|J9P1X5\|J9P1X5\_CANLF, tr\|J9P4L9\|J9P4L9\_CANLF, tr\|O46880\|O46880\_CANLF, tr\|O46882\|O46882\_CANLF |
| 56 | Protein digestion and absorption | tr\|A0A5F4C4P9\|A0A5F4C4P9\_CANLF, tr\|A0A5F4CBC5\|A0A5F4CBC5\_CANLF, tr\|A0A5F4D4M8\|A0A5F4D4M8\_CANLF, tr\|A0A5F4DFP9\|A0A5F4DFP9\_CANLF, tr\|E2R0D7\|E2R0D7\_CANLF, tr\|F1PBA1\|F1PBA1\_CANLF, tr\|F1PCE8\|F1PCE8\_CANLF, tr\|F1PEK0\|F1PEK0\_CANLF, tr\|F1PFM6\|F1PFM6\_CANLF, tr\|F1PG69\|F1PG69\_CANLF, tr\|F1PHX8\|F1PHX8\_CANLF, tr\|F1PHY1\|F1PHY1\_CANLF, tr\|F1PR31\|F1PR31\_CANLF, tr\|F1Q133\|F1Q133\_CANLF, tr\|F1Q2F7\|F1Q2F7\_CANLF, tr\|F1Q3I5\|F1Q3I5\_CANLF, tr\|F6XWS5\|F6XWS5\_CANLF, tr\|J9P889\|J9P889\_CANLF, tr\|K7ZSN9\|K7ZSN9\_CANLF, tr\|Q28272\|Q28272\_CANLF, tr\|Q597P9\|Q597P9\_CANLF, tr\|Q9N1R4\|Q9N1R4\_CANLF |
| 57 | Cardiac muscle contraction | tr\|A0A5F4BXT7\|A0A5F4BXT7\_CANLF, tr\|A0A5F4BYS0\|A0A5F4BYS0\_CANLF, tr\|A0A5F4C4P9\|A0A5F4C4P9\_CANLF, tr\|A0A5F4C9C0\|A0A5F4C9C0\_CANLF, tr\|A0A5F4CBF2\|A0A5F4CBF2\_CANLF, tr\|A0A5F4CFM8\|A0A5F4CFM8\_CANLF, tr\|A0A5F4CUQ1\|A0A5F4CUQ1\_CANLF, tr\|A0A5F4CWM9\|A0A5F4CWM9\_CANLF, tr\|A0A5F4D0C4\|A0A5F4D0C4\_CANLF, tr\|A0A5F4D721\|A0A5F4D721\_CANLF, tr\|A0A5F4DCE0\|A0A5F4DCE0\_CANLF, tr\|E2QUA8\|E2QUA8\_CANLF, tr\|E2RHL0\|E2RHL0\_CANLF, tr\|F1PBA1\|F1PBA1\_CANLF, tr\|F1PID1\|F1PID1\_CANLF, tr\|F1PJ01\|F1PJ01\_CANLF, tr\|F1PLP2\|F1PLP2\_CANLF, tr\|F1PVV7\|F1PVV7\_CANLF, tr\|F1PYS6\|F1PYS6\_CANLF, tr\|G1K2D5\|G1K2D5\_CANLF, tr\|J9P2B9\|J9P2B9\_CANLF, tr\|V5LKV5\|V5LKV5\_CANLF |
| 58 | Salivary secretion | sp\|P30803\|ADCY5\_CANLF, sp\|P63091\|GNAS\_CANLF, tr\|A0A5F4BVY4\|A0A5F4BVY4\_CANLF, tr\|A0A5F4CNW6\|A0A5F4CNW6\_CANLF, tr\|A0A5F4CSQ5\|A0A5F4CSQ5\_CANLF, tr\|E2RS74\|E2RS74\_CANLF, tr\|F1PBA1\|F1PBA1\_CANLF, tr\|F1PI77\|F1PI77\_CANLF, tr\|F1PID1\|F1PID1\_CANLF, tr\|F1PKU7\|F1PKU7\_CANLF, tr\|F1PW86\|F1PW86\_CANLF, tr\|F1PXM5\|F1PXM5\_CANLF, tr\|F1Q1D5\|F1Q1D5\_CANLF, tr\|G1K265\|G1K265\_CANLF, tr\|G1K2D5\|G1K2D5\_CANLF, tr\|J9NWJ7\|J9NWJ7\_CANLF, tr\|J9NXT4\|J9NXT4\_CANLF, tr\|J9P2M3\|J9P2M3\_CANLF, tr\|J9P8P9\|J9P8P9\_CANLF, tr\|J9P8S3\|J9P8S3\_CANLF, tr\|J9P988\|J9P988\_CANLF |
| 59 | VEGF signaling pathway | sp\|Q1HG70\|MP2K2\_CANLF, tr\|A0A5F4C5E5\|A0A5F4C5E5\_CANLF, tr\|A0A5F4D5V0\|A0A5F4D5V0\_CANLF, tr\|A0A5F4DI14\|A0A5F4DI14\_CANLF, tr\|A0A5F4DIK8\|A0A5F4DIK8\_CANLF, tr\|E2QUL4\|E2QUL4\_CANLF, tr\|E2RRA9\|E2RRA9\_CANLF, tr\|E2RTE0\|E2RTE0\_CANLF, tr\|F1PKU7\|F1PKU7\_CANLF, tr\|F1PQC4\|F1PQC4\_CANLF, tr\|F1PUT6\|F1PUT6\_CANLF, tr\|F1PYE3\|F1PYE3\_CANLF, tr\|F1PYQ1\|F1PYQ1\_CANLF, tr\|F1Q1D5\|F1Q1D5\_CANLF, tr\|F2Z4P5\|F2Z4P5\_CANLF, tr\|F6UQ33\|F6UQ33\_CANLF, tr\|F6V544\|F6V544\_CANLF, tr\|J9NXT9\|J9NXT9\_CANLF, tr\|J9P1X5\|J9P1X5\_CANLF, tr\|J9P4L9\|J9P4L9\_CANLF, tr\|Q45KI9\|Q45KI9\_CANLF |
| 60 | Antigen processing and presentation | sp\|Q9GL24\|CATL1\_CANLF, tr\|A0A5F4BSL9\|A0A5F4BSL9\_CANLF, tr\|A0A5F4BUG1\|A0A5F4BUG1\_CANLF, tr\|A0A5F4DDP0\|A0A5F4DDP0\_CANLF, tr\|E2RD86\|E2RD86\_CANLF, tr\|E2RLS3\|E2RLS3\_CANLF, tr\|E2RN10\|E2RN10\_CANLF, tr\|E2RNP9\|E2RNP9\_CANLF, tr\|F1P8B4\|F1P8B4\_CANLF, tr\|F1PD77\|F1PD77\_CANLF, tr\|F1PGY1\|F1PGY1\_CANLF, tr\|F1PIC7\|F1PIC7\_CANLF, tr\|F1PYJ0\|F1PYJ0\_CANLF, tr\|F6UY11\|F6UY11\_CANLF, tr\|F6UYJ9\|F6UYJ9\_CANLF, tr\|G1K268\|G1K268\_CANLF, tr\|H9GW49\|H9GW49\_CANLF, tr\|O46880\|O46880\_CANLF, tr\|O46882\|O46882\_CANLF, tr\|Q30348\|Q30348\_CANLF, tr\|Q5W417\|Q5W417\_CANLF |
| 61 | Fc epsilon RI signaling pathway | sp\|Q1HG70\|MP2K2\_CANLF, tr\|A0A5F4C4S3\|A0A5F4C4S3\_CANLF, tr\|A0A5F4C566\|A0A5F4C566\_CANLF, tr\|A0A5F4C5E5\|A0A5F4C5E5\_CANLF, tr\|A0A5F4CBH6\|A0A5F4CBH6\_CANLF, tr\|A0A5F4D4H5\|A0A5F4D4H5\_CANLF, tr\|E2RC09\|E2RC09\_CANLF, tr\|E2RCC8\|E2RCC8\_CANLF, tr\|E2RE31\|E2RE31\_CANLF, tr\|E2RGU7\|E2RGU7\_CANLF, tr\|E2RNP2\|E2RNP2\_CANLF, tr\|E2RRA9\|E2RRA9\_CANLF, tr\|F1PDX2\|F1PDX2\_CANLF, tr\|F1PKU7\|F1PKU7\_CANLF, tr\|F1PYQ1\|F1PYQ1\_CANLF, tr\|F1Q1D5\|F1Q1D5\_CANLF, tr\|F6UQ33\|F6UQ33\_CANLF, tr\|J9NYG3\|J9NYG3\_CANLF, tr\|J9P1X5\|J9P1X5\_CANLF, tr\|J9P4L9\|J9P4L9\_CANLF |
| 62 | Long-term potentiation | sp\|Q1HG70\|MP2K2\_CANLF, tr\|A0A5F4BVY4\|A0A5F4BVY4\_CANLF, tr\|A0A5F4C5E5\|A0A5F4C5E5\_CANLF, tr\|A0A5F4CJ79\|A0A5F4CJ79\_CANLF, tr\|A0A5F4CNW6\|A0A5F4CNW6\_CANLF, tr\|A0A5F4CXJ9\|A0A5F4CXJ9\_CANLF, tr\|A0A5F4D429\|A0A5F4D429\_CANLF, tr\|E2R2K4\|E2R2K4\_CANLF, tr\|E2RG56\|E2RG56\_CANLF, tr\|F1P874\|F1P874\_CANLF, tr\|F1PKU7\|F1PKU7\_CANLF, tr\|F1PW86\|F1PW86\_CANLF, tr\|F1PXM5\|F1PXM5\_CANLF, tr\|F1Q1D5\|F1Q1D5\_CANLF, tr\|F2Z4P5\|F2Z4P5\_CANLF, tr\|G1K2D5\|G1K2D5\_CANLF, tr\|J9NWJ7\|J9NWJ7\_CANLF, tr\|J9NXT4\|J9NXT4\_CANLF, tr\|J9P4L9\|J9P4L9\_CANLF, tr\|J9P8S3\|J9P8S3\_CANLF |
| 63 | Glioma | sp\|Q1HG70\|MP2K2\_CANLF, tr\|A0A5F4C4S3\|A0A5F4C4S3\_CANLF, tr\|A0A5F4C566\|A0A5F4C566\_CANLF, tr\|A0A5F4C5E5\|A0A5F4C5E5\_CANLF, tr\|A0A5F4CJ79\|A0A5F4CJ79\_CANLF, tr\|A0A5F4CT67\|A0A5F4CT67\_CANLF, tr\|A0A5F4D6R1\|A0A5F4D6R1\_CANLF, tr\|E2RG56\|E2RG56\_CANLF, tr\|E2RRA9\|E2RRA9\_CANLF, tr\|F1P874\|F1P874\_CANLF, tr\|F1PF03\|F1PF03\_CANLF, tr\|F1PKR8\|F1PKR8\_CANLF, tr\|F1PKU7\|F1PKU7\_CANLF, tr\|F1PQC4\|F1PQC4\_CANLF, tr\|F1Q1D5\|F1Q1D5\_CANLF, tr\|G1K2D5\|G1K2D5\_CANLF, tr\|J9NWJ7\|J9NWJ7\_CANLF, tr\|J9P1X5\|J9P1X5\_CANLF, tr\|J9P4L9\|J9P4L9\_CANLF, tr\|J9P8S3\|J9P8S3\_CANLF |
| 64 | Chronic myeloid leukemia | sp\|Q1HG70\|MP2K2\_CANLF, tr\|A0A5F4C0L0\|A0A5F4C0L0\_CANLF, tr\|A0A5F4C4S3\|A0A5F4C4S3\_CANLF, tr\|A0A5F4C566\|A0A5F4C566\_CANLF, tr\|A0A5F4C5E5\|A0A5F4C5E5\_CANLF, tr\|A0A5F4DAI3\|A0A5F4DAI3\_CANLF, tr\|E2QWD3\|E2QWD3\_CANLF, tr\|E2RF98\|E2RF98\_CANLF, tr\|E2RRA9\|E2RRA9\_CANLF, tr\|F1P874\|F1P874\_CANLF, tr\|F1PEX7\|F1PEX7\_CANLF, tr\|F1PNW8\|F1PNW8\_CANLF, tr\|F1PQC4\|F1PQC4\_CANLF, tr\|F1PR63\|F1PR63\_CANLF, tr\|J9NYG3\|J9NYG3\_CANLF, tr\|J9P1X5\|J9P1X5\_CANLF, tr\|J9P2Z2\|J9P2Z2\_CANLF, tr\|J9P4L9\|J9P4L9\_CANLF, tr\|Q45KI9\|Q45KI9\_CANLF, tr\|Q68V02\|Q68V02\_CANLF |
| 65 | Melanogenesis | sp\|P30803\|ADCY5\_CANLF, sp\|P38400\|GNAI2\_CANLF, sp\|P63091\|GNAS\_CANLF, sp\|Q1HG70\|MP2K2\_CANLF, tr\|A0A5F4BVY4\|A0A5F4BVY4\_CANLF, tr\|A0A5F4C5E5\|A0A5F4C5E5\_CANLF, tr\|A0A5F4CJ79\|A0A5F4CJ79\_CANLF, tr\|A0A5F4D0Z3\|A0A5F4D0Z3\_CANLF, tr\|E2RG56\|E2RG56\_CANLF, tr\|F1PKU7\|F1PKU7\_CANLF, tr\|F1PXM5\|F1PXM5\_CANLF, tr\|F1PYJ0\|F1PYJ0\_CANLF, tr\|F1Q1D5\|F1Q1D5\_CANLF, tr\|G1K2D5\|G1K2D5\_CANLF, tr\|J9NWJ7\|J9NWJ7\_CANLF, tr\|J9NXT4\|J9NXT4\_CANLF, tr\|J9P2M3\|J9P2M3\_CANLF, tr\|J9P4L9\|J9P4L9\_CANLF, tr\|J9P8S3\|J9P8S3\_CANLF |
| 66 | B cell receptor signaling pathway | sp\|Q1HG70\|MP2K2\_CANLF, tr\|A0A5F4C4S3\|A0A5F4C4S3\_CANLF, tr\|A0A5F4C566\|A0A5F4C566\_CANLF, tr\|A0A5F4C5E5\|A0A5F4C5E5\_CANLF, tr\|A0A5F4CBH6\|A0A5F4CBH6\_CANLF, tr\|A0A5F4D4H5\|A0A5F4D4H5\_CANLF, tr\|E2RA73\|E2RA73\_CANLF, tr\|E2RCC8\|E2RCC8\_CANLF, tr\|E2RE31\|E2RE31\_CANLF, tr\|E2RRA9\|E2RRA9\_CANLF, tr\|F1PB66\|F1PB66\_CANLF, tr\|F1PCK2\|F1PCK2\_CANLF, tr\|F1PKU7\|F1PKU7\_CANLF, tr\|F1PYQ1\|F1PYQ1\_CANLF, tr\|F1Q1D5\|F1Q1D5\_CANLF, tr\|F2Z4P5\|F2Z4P5\_CANLF, tr\|J9NYG3\|J9NYG3\_CANLF, tr\|J9P1X5\|J9P1X5\_CANLF, tr\|J9P4L9\|J9P4L9\_CANLF |
| 67 | Endometrial cancer | sp\|F1PAA9\|CADH1\_CANLF, sp\|Q1HG70\|MP2K2\_CANLF, tr\|A0A5F4C4S3\|A0A5F4C4S3\_CANLF, tr\|A0A5F4C566\|A0A5F4C566\_CANLF, tr\|A0A5F4C5E5\|A0A5F4C5E5\_CANLF, tr\|A0A5F4CT67\|A0A5F4CT67\_CANLF, tr\|A0A5F4D0Z3\|A0A5F4D0Z3\_CANLF, tr\|A0A5F4D6R1\|A0A5F4D6R1\_CANLF, tr\|E2R5H4\|E2R5H4\_CANLF, tr\|E2R9S7\|E2R9S7\_CANLF, tr\|E2RRA9\|E2RRA9\_CANLF, tr\|F1P874\|F1P874\_CANLF, tr\|F1PF03\|F1PF03\_CANLF, tr\|F1PKR8\|F1PKR8\_CANLF, tr\|F1Q2Y3\|F1Q2Y3\_CANLF, tr\|J9P1X5\|J9P1X5\_CANLF, tr\|J9P4L9\|J9P4L9\_CANLF, tr\|Q45KI9\|Q45KI9\_CANLF |
| 68 | Oxidative phosphorylation | tr\|A0A5F4BYS0\|A0A5F4BYS0\_CANLF, tr\|A0A5F4C484\|A0A5F4C484\_CANLF, tr\|A0A5F4D0C4\|A0A5F4D0C4\_CANLF, tr\|A0A5F4D296\|A0A5F4D296\_CANLF, tr\|A0A5F4D6E6\|A0A5F4D6E6\_CANLF, tr\|A0A5F4DJV1\|A0A5F4DJV1\_CANLF, tr\|E2QUA8\|E2QUA8\_CANLF, tr\|E2QV28\|E2QV28\_CANLF, tr\|E2QXJ5\|E2QXJ5\_CANLF, tr\|E2RD75\|E2RD75\_CANLF, tr\|E2RHL0\|E2RHL0\_CANLF, tr\|E2RNG2\|E2RNG2\_CANLF, tr\|F1PG47\|F1PG47\_CANLF, tr\|F1PLL0\|F1PLL0\_CANLF, tr\|F1PLP2\|F1PLP2\_CANLF, tr\|F1PY24\|F1PY24\_CANLF, tr\|F1Q3W8\|F1Q3W8\_CANLF, tr\|V5LKV5\|V5LKV5\_CANLF |
| 69 | Inositol phosphate metabolism | tr\|A0A346M2B2\|A0A346M2B2\_CANLF, tr\|A0A5F4BQ52\|A0A5F4BQ52\_CANLF, tr\|A0A5F4BVY4\|A0A5F4BVY4\_CANLF, tr\|A0A5F4CT67\|A0A5F4CT67\_CANLF, tr\|A0A5F4D6R1\|A0A5F4D6R1\_CANLF, tr\|A0A5F4D8L2\|A0A5F4D8L2\_CANLF, tr\|E2QTD8\|E2QTD8\_CANLF, tr\|E2R9U0\|E2R9U0\_CANLF, tr\|E2RSF7\|E2RSF7\_CANLF, tr\|E2RSJ5\|E2RSJ5\_CANLF, tr\|F1PKR8\|F1PKR8\_CANLF, tr\|F1PMC5\|F1PMC5\_CANLF, tr\|F1PPB7\|F1PPB7\_CANLF, tr\|F1PWW8\|F1PWW8\_CANLF, tr\|J9JHK8\|J9JHK8\_CANLF, tr\|J9P2C8\|J9P2C8\_CANLF, tr\|J9P2I9\|J9P2I9\_CANLF, tr\|J9P6E1\|J9P6E1\_CANLF |
| 70 | Hematopoietic cell lineage | sp\|P02727\|GLPA\_CANLF, tr\|A0A5F4BUG1\|A0A5F4BUG1\_CANLF, tr\|A0A5F4BVB0\|A0A5F4BVB0\_CANLF, tr\|A0A5F4CBH6\|A0A5F4CBH6\_CANLF, tr\|A0A5F4CIA0\|A0A5F4CIA0\_CANLF, tr\|A0A5F4CUX4\|A0A5F4CUX4\_CANLF, tr\|E2QSC8\|E2QSC8\_CANLF, tr\|E2R6A0\|E2R6A0\_CANLF, tr\|E2RCC8\|E2RCC8\_CANLF, tr\|E2RE31\|E2RE31\_CANLF, tr\|F1PP33\|F1PP33\_CANLF, tr\|F1PPG5\|F1PPG5\_CANLF, tr\|F1PQ29\|F1PQ29\_CANLF, tr\|J9NVU0\|J9NVU0\_CANLF, tr\|J9P423\|J9P423\_CANLF, tr\|J9P988\|J9P988\_CANLF, tr\|Q30348\|Q30348\_CANLF, tr\|Q3HR31\|Q3HR31\_CANLF |
| 71 | Apoptosis | tr\|A0A5F4CDU8\|A0A5F4CDU8\_CANLF, tr\|A0A5F4CE07\|A0A5F4CE07\_CANLF, tr\|A0A5F4D0M2\|A0A5F4D0M2\_CANLF, tr\|A0A5F4DJ19\|A0A5F4DJ19\_CANLF, tr\|E2QZV5\|E2QZV5\_CANLF, tr\|E2R541\|E2R541\_CANLF, tr\|E2RJG8\|E2RJG8\_CANLF, tr\|E2RRA1\|E2RRA1\_CANLF, tr\|E2RRA9\|E2RRA9\_CANLF, tr\|E2RRD1\|E2RRD1\_CANLF, tr\|F1P975\|F1P975\_CANLF, tr\|F1PIK5\|F1PIK5\_CANLF, tr\|F2Z4P5\|F2Z4P5\_CANLF, tr\|F6UZV8\|F6UZV8\_CANLF, tr\|J9NXT4\|J9NXT4\_CANLF, tr\|J9P0U9\|J9P0U9\_CANLF, tr\|J9P1X5\|J9P1X5\_CANLF, tr\|Q45KI9\|Q45KI9\_CANLF |
| 72 | Complement and coagulation cascades | sp\|P02677\|FIBB\_CANLF, tr\|A1ILJ0\|A1ILJ0\_CANLF, tr\|E2R886\|E2R886\_CANLF, tr\|E2RES2\|E2RES2\_CANLF, tr\|E2RS80\|E2RS80\_CANLF, tr\|F1P8G0\|F1P8G0\_CANLF, tr\|F1PB37\|F1PB37\_CANLF, tr\|F1PGM1\|F1PGM1\_CANLF, tr\|F1PGS2\|F1PGS2\_CANLF, tr\|F1PNR5\|F1PNR5\_CANLF, tr\|F1PRW2\|F1PRW2\_CANLF, tr\|F1PWC7\|F1PWC7\_CANLF, tr\|F6UME0\|F6UME0\_CANLF, tr\|G1K2D9\|G1K2D9\_CANLF, tr\|J9NRV7\|J9NRV7\_CANLF, tr\|J9NTL7\|J9NTL7\_CANLF, tr\|J9PBN6\|J9PBN6\_CANLF, tr\|Q8MJD1\|Q8MJD1\_CANLF |
| 73 | Renal cell carcinoma | sp\|Q1HG70\|MP2K2\_CANLF, tr\|A0A5F4C4S3\|A0A5F4C4S3\_CANLF, tr\|A0A5F4C566\|A0A5F4C566\_CANLF, tr\|A0A5F4C5E5\|A0A5F4C5E5\_CANLF, tr\|A0A5F4D5V0\|A0A5F4D5V0\_CANLF, tr\|A0A5F4DF90\|A0A5F4DF90\_CANLF, tr\|E2QWD3\|E2QWD3\_CANLF, tr\|E2R2K4\|E2R2K4\_CANLF, tr\|E2REH3\|E2REH3\_CANLF, tr\|E2RF98\|E2RF98\_CANLF, tr\|E2RGR9\|E2RGR9\_CANLF, tr\|E2RRA9\|E2RRA9\_CANLF, tr\|F1P874\|F1P874\_CANLF, tr\|F1PYQ1\|F1PYQ1\_CANLF, tr\|F1Q2Y7\|F1Q2Y7\_CANLF, tr\|J9P1X5\|J9P1X5\_CANLF, tr\|J9P4L9\|J9P4L9\_CANLF |
| 74 | NOD-like receptor signaling pathway | sp\|P41148\|ENPL\_CANLF, tr\|A0A5F4BSL9\|A0A5F4BSL9\_CANLF, tr\|A0A5F4C5E5\|A0A5F4C5E5\_CANLF, tr\|A0A5F4CMN1\|A0A5F4CMN1\_CANLF, tr\|A0A5F4CWJ6\|A0A5F4CWJ6\_CANLF, tr\|E2R3R9\|E2R3R9\_CANLF, tr\|E2R8L1\|E2R8L1\_CANLF, tr\|E2RI72\|E2RI72\_CANLF, tr\|E2RLS3\|E2RLS3\_CANLF, tr\|E2RM43\|E2RM43\_CANLF, tr\|E2RQV5\|E2RQV5\_CANLF, tr\|E2RSJ3\|E2RSJ3\_CANLF, tr\|F1PGY1\|F1PGY1\_CANLF, tr\|F1PIV4\|F1PIV4\_CANLF, tr\|F1PUS0\|F1PUS0\_CANLF, tr\|F1Q433\|F1Q433\_CANLF, tr\|J9PB40\|J9PB40\_CANLF |
| 75 | Progesterone-mediated oocyte maturation | sp\|P30803\|ADCY5\_CANLF, sp\|P38400\|GNAI2\_CANLF, tr\|A0A5F4BSL9\|A0A5F4BSL9\_CANLF, tr\|A0A5F4C5E5\|A0A5F4C5E5\_CANLF, tr\|A0A5F4CXJ9\|A0A5F4CXJ9\_CANLF, tr\|E2QY29\|E2QY29\_CANLF, tr\|E2RKS8\|E2RKS8\_CANLF, tr\|E2RLS3\|E2RLS3\_CANLF, tr\|E2RRA9\|E2RRA9\_CANLF, tr\|F1P874\|F1P874\_CANLF, tr\|F1P9J9\|F1P9J9\_CANLF, tr\|F1PGY1\|F1PGY1\_CANLF, tr\|F1PTH2\|F1PTH2\_CANLF, tr\|J9NXT4\|J9NXT4\_CANLF, tr\|J9P1X5\|J9P1X5\_CANLF, tr\|J9P2M3\|J9P2M3\_CANLF, tr\|J9P4L9\|J9P4L9\_CANLF |
| 76 | Vasopressin-regulated water reabsorption | sp\|P51147\|RAB5C\_CANLF, sp\|P63091\|GNAS\_CANLF, tr\|A0A5F4CQ75\|A0A5F4CQ75\_CANLF, tr\|A0A5F4CZ62\|A0A5F4CZ62\_CANLF, tr\|A0A5F4CZW1\|A0A5F4CZW1\_CANLF, tr\|A0A5F4DJ85\|A0A5F4DJ85\_CANLF, tr\|E2QYN0\|E2QYN0\_CANLF, tr\|E2QZ05\|E2QZ05\_CANLF, tr\|E2RAL0\|E2RAL0\_CANLF, tr\|E2RFR5\|E2RFR5\_CANLF, tr\|E2RH71\|E2RH71\_CANLF, tr\|F1P6V8\|F1P6V8\_CANLF, tr\|F1PYJ0\|F1PYJ0\_CANLF, tr\|F6UMF1\|F6UMF1\_CANLF, tr\|J9JHN1\|J9JHN1\_CANLF, tr\|J9NXT4\|J9NXT4\_CANLF, tr\|J9P2M3\|J9P2M3\_CANLF |
| 77 | Pancreatic cancer | tr\|A0A5F4C5E5\|A0A5F4C5E5\_CANLF, tr\|A0A5F4CC58\|A0A5F4CC58\_CANLF, tr\|A0A5F4CYP2\|A0A5F4CYP2\_CANLF, tr\|A0A5F4D5V0\|A0A5F4D5V0\_CANLF, tr\|E2R0Z1\|E2R0Z1\_CANLF, tr\|E2R651\|E2R651\_CANLF, tr\|E2RI99\|E2RI99\_CANLF, tr\|E2RRA9\|E2RRA9\_CANLF, tr\|F1P874\|F1P874\_CANLF, tr\|F1PF03\|F1PF03\_CANLF, tr\|F1PS09\|F1PS09\_CANLF, tr\|F1PYQ1\|F1PYQ1\_CANLF, tr\|J9NX28\|J9NX28\_CANLF, tr\|J9P1X5\|J9P1X5\_CANLF, tr\|J9P4L9\|J9P4L9\_CANLF, tr\|J9P5X9\|J9P5X9\_CANLF, tr\|Q45KI9\|Q45KI9\_CANLF |
| 78 | Staphylococcus aureus infection | tr\|A0A5F4BUG1\|A0A5F4BUG1\_CANLF, tr\|A0A5F4CBH6\|A0A5F4CBH6\_CANLF, tr\|A0A5F4DJY1\|A0A5F4DJY1\_CANLF, tr\|E2R150\|E2R150\_CANLF, tr\|E2RCC8\|E2RCC8\_CANLF, tr\|E2RE31\|E2RE31\_CANLF, tr\|E2REU6\|E2REU6\_CANLF, tr\|E2RS80\|E2RS80\_CANLF, tr\|F1P8G0\|F1P8G0\_CANLF, tr\|F1PB95\|F1PB95\_CANLF, tr\|F1PGM1\|F1PGM1\_CANLF, tr\|F1PRW2\|F1PRW2\_CANLF, tr\|F1Q0N9\|F1Q0N9\_CANLF, tr\|G1K2D9\|G1K2D9\_CANLF, tr\|J9NTL7\|J9NTL7\_CANLF, tr\|Q30348\|Q30348\_CANLF, tr\|Q8MJD1\|Q8MJD1\_CANLF |
| 79 | RNA degradation | tr\|A0A5F4C4W3\|A0A5F4C4W3\_CANLF, tr\|A0A5F4CYJ8\|A0A5F4CYJ8\_CANLF, tr\|A0A5F4D1E4\|A0A5F4D1E4\_CANLF, tr\|A0A5F4D4C8\|A0A5F4D4C8\_CANLF, tr\|A0A5F4DCR1\|A0A5F4DCR1\_CANLF, tr\|A0A5F4DJ33\|A0A5F4DJ33\_CANLF, tr\|E2QSY2\|E2QSY2\_CANLF, tr\|E2QUU5\|E2QUU5\_CANLF, tr\|E2R3I2\|E2R3I2\_CANLF, tr\|E2RJG8\|E2RJG8\_CANLF, tr\|E2RPX4\|E2RPX4\_CANLF, tr\|E2RSD6\|E2RSD6\_CANLF, tr\|F1PB11\|F1PB11\_CANLF, tr\|F1PDS0\|F1PDS0\_CANLF, tr\|F1Q4H9\|F1Q4H9\_CANLF, tr\|F6XU41\|F6XU41\_CANLF, tr\|Q28226\|Q28226\_CANLF |
| 80 | Leishmaniasis | tr\|A0A5F4BRD0\|A0A5F4BRD0\_CANLF, tr\|A0A5F4BUG1\|A0A5F4BUG1\_CANLF, tr\|A0A5F4C5E5\|A0A5F4C5E5\_CANLF, tr\|A0A5F4CBH6\|A0A5F4CBH6\_CANLF, tr\|A0A5F4DCP4\|A0A5F4DCP4\_CANLF, tr\|E2R3R9\|E2R3R9\_CANLF, tr\|E2RCC8\|E2RCC8\_CANLF, tr\|E2RE31\|E2RE31\_CANLF, tr\|E2RI99\|E2RI99\_CANLF, tr\|E2RT60\|E2RT60\_CANLF, tr\|F1PGM1\|F1PGM1\_CANLF, tr\|F1PKU7\|F1PKU7\_CANLF, tr\|F1PQ29\|F1PQ29\_CANLF, tr\|F1Q1D5\|F1Q1D5\_CANLF, tr\|F6Y4A3\|F6Y4A3\_CANLF, tr\|Q30348\|Q30348\_CANLF |
| 81 | Valine, leucine and isoleucine degradation | tr\|A0A5F4CYX7\|A0A5F4CYX7\_CANLF, tr\|E2QVM3\|E2QVM3\_CANLF, tr\|E2REJ7\|E2REJ7\_CANLF, tr\|E2RQ99\|E2RQ99\_CANLF, tr\|E2RRP1\|E2RRP1\_CANLF, tr\|F1P6W6\|F1P6W6\_CANLF, tr\|F1PAB7\|F1PAB7\_CANLF, tr\|F1PAZ6\|F1PAZ6\_CANLF, tr\|F1PE09\|F1PE09\_CANLF, tr\|F1PIP0\|F1PIP0\_CANLF, tr\|F1PJ13\|F1PJ13\_CANLF, tr\|F1PYB6\|F1PYB6\_CANLF, tr\|F6XHT8\|F6XHT8\_CANLF, tr\|J9JHD4\|J9JHD4\_CANLF, tr\|J9NZC6\|J9NZC6\_CANLF, tr\|J9P2I9\|J9P2I9\_CANLF |
| 82 | mTOR signaling pathway | tr\|A0A5F4C5E5\|A0A5F4C5E5\_CANLF, tr\|A0A5F4CD52\|A0A5F4CD52\_CANLF, tr\|A0A5F4CFR9\|A0A5F4CFR9\_CANLF, tr\|A0A5F4CH02\|A0A5F4CH02\_CANLF, tr\|A0A5F4CNS5\|A0A5F4CNS5\_CANLF, tr\|A0A5F4CXJ9\|A0A5F4CXJ9\_CANLF, tr\|A0A5F4DEP2\|A0A5F4DEP2\_CANLF, tr\|E2QSP3\|E2QSP3\_CANLF, tr\|E2RP05\|E2RP05\_CANLF, tr\|E2RPA3\|E2RPA3\_CANLF, tr\|E2RRA9\|E2RRA9\_CANLF, tr\|F1P7Q1\|F1P7Q1\_CANLF, tr\|F1P874\|F1P874\_CANLF, tr\|F1PQ92\|F1PQ92\_CANLF, tr\|F1Q2Y3\|F1Q2Y3\_CANLF, tr\|J9P1X5\|J9P1X5\_CANLF |
| 83 | Long-term depression | sp\|P38400\|GNAI2\_CANLF, sp\|P63091\|GNAS\_CANLF, sp\|Q1HG70\|MP2K2\_CANLF, tr\|A0A5F4BVY4\|A0A5F4BVY4\_CANLF, tr\|A0A5F4C5E5\|A0A5F4C5E5\_CANLF, tr\|A0A5F4CNW6\|A0A5F4CNW6\_CANLF, tr\|A0A5F4D4H5\|A0A5F4D4H5\_CANLF, tr\|F1P874\|F1P874\_CANLF, tr\|F1PI77\|F1PI77\_CANLF, tr\|F1PKU7\|F1PKU7\_CANLF, tr\|F1PW86\|F1PW86\_CANLF, tr\|F1PX75\|F1PX75\_CANLF, tr\|F1PXM5\|F1PXM5\_CANLF, tr\|F1Q1D5\|F1Q1D5\_CANLF, tr\|F6UQ33\|F6UQ33\_CANLF, tr\|J9P4L9\|J9P4L9\_CANLF |
| 84 | Peroxisome | sp\|O97492\|CATA\_CANLF, sp\|Q8WNN6\|SODC\_CANLF, tr\|A0A5F4D8P8\|A0A5F4D8P8\_CANLF, tr\|E2RCH8\|E2RCH8\_CANLF, tr\|E2RCK9\|E2RCK9\_CANLF, tr\|E2RHG2\|E2RHG2\_CANLF, tr\|F1P6W6\|F1P6W6\_CANLF, tr\|F1P843\|F1P843\_CANLF, tr\|F1P9W3\|F1P9W3\_CANLF, tr\|F1PAL6\|F1PAL6\_CANLF, tr\|F1PTS5\|F1PTS5\_CANLF, tr\|F1PZA1\|F1PZA1\_CANLF, tr\|F1Q3R2\|F1Q3R2\_CANLF, tr\|J9NRV1\|J9NRV1\_CANLF, tr\|J9P9G0\|J9P9G0\_CANLF |
| 85 | Toll-like receptor signaling pathway | sp\|Q1HG70\|MP2K2\_CANLF, tr\|A0A5F4C5E5\|A0A5F4C5E5\_CANLF, tr\|A0A5F4CDU8\|A0A5F4CDU8\_CANLF, tr\|A0A5F4CXQ7\|A0A5F4CXQ7\_CANLF, tr\|E2R3R9\|E2R3R9\_CANLF, tr\|E2RCV5\|E2RCV5\_CANLF, tr\|E2RGZ5\|E2RGZ5\_CANLF, tr\|E2RI99\|E2RI99\_CANLF, tr\|E2RRA9\|E2RRA9\_CANLF, tr\|F1PGY6\|F1PGY6\_CANLF, tr\|F1PIK5\|F1PIK5\_CANLF, tr\|F1PYQ1\|F1PYQ1\_CANLF, tr\|F6XD36\|F6XD36\_CANLF, tr\|J9P0U9\|J9P0U9\_CANLF, tr\|J9P1X5\|J9P1X5\_CANLF |
| 86 | Glutathione metabolism | tr\|A0A5F4C1A4\|A0A5F4C1A4\_CANLF, tr\|A0A5F4CIA0\|A0A5F4CIA0\_CANLF, tr\|A0A5F4CNR7\|A0A5F4CNR7\_CANLF, tr\|A0A5F4D6M3\|A0A5F4D6M3\_CANLF, tr\|A0A5F4D8P8\|A0A5F4D8P8\_CANLF, tr\|E2QVM3\|E2QVM3\_CANLF, tr\|E2REJ7\|E2REJ7\_CANLF, tr\|F1PE09\|F1PE09\_CANLF, tr\|F1PF82\|F1PF82\_CANLF, tr\|F1PYB6\|F1PYB6\_CANLF, tr\|F1PZA1\|F1PZA1\_CANLF, tr\|F1Q0J0\|F1Q0J0\_CANLF, tr\|F6XH46\|F6XH46\_CANLF, tr\|J9NRV1\|J9NRV1\_CANLF, tr\|J9P028\|J9P028\_CANLF |
| 87 | Pyruvate metabolism | tr\|A0A5F4CZB6\|A0A5F4CZB6\_CANLF, tr\|A0A5F4DKH0\|A0A5F4DKH0\_CANLF, tr\|E2QV08\|E2QV08\_CANLF, tr\|E2R4S5\|E2R4S5\_CANLF, tr\|E2RFL0\|E2RFL0\_CANLF, tr\|E2RQ99\|E2RQ99\_CANLF, tr\|F1PAB7\|F1PAB7\_CANLF, tr\|F1PTT5\|F1PTT5\_CANLF, tr\|F1PVW0\|F1PVW0\_CANLF, tr\|F1PYG8\|F1PYG8\_CANLF, tr\|J9JHD4\|J9JHD4\_CANLF, tr\|J9P208\|J9P208\_CANLF, tr\|J9P3N9\|J9P3N9\_CANLF, tr\|J9P7N4\|J9P7N4\_CANLF, tr\|J9P7R2\|J9P7R2\_CANLF |
| 88 | PPAR signaling pathway | tr\|A0A5F4D6Z6\|A0A5F4D6Z6\_CANLF, tr\|A0A5F4DEY9\|A0A5F4DEY9\_CANLF, tr\|D9ILX1\|D9ILX1\_CANLF, tr\|E2R5H4\|E2R5H4\_CANLF, tr\|F1P6W6\|F1P6W6\_CANLF, tr\|F1PDJ5\|F1PDJ5\_CANLF, tr\|F1PND0\|F1PND0\_CANLF, tr\|F1Q0X8\|F1Q0X8\_CANLF, tr\|F1Q249\|F1Q249\_CANLF, tr\|F1Q2Y3\|F1Q2Y3\_CANLF, tr\|F1Q3R2\|F1Q3R2\_CANLF, tr\|F1Q3X6\|F1Q3X6\_CANLF, tr\|J9P5F9\|J9P5F9\_CANLF, tr\|J9P9G0\|J9P9G0\_CANLF |
| 89 | Non-small cell lung cancer | sp\|Q1HG70\|MP2K2\_CANLF, tr\|A0A5F4C4S3\|A0A5F4C4S3\_CANLF, tr\|A0A5F4C566\|A0A5F4C566\_CANLF, tr\|A0A5F4C5E5\|A0A5F4C5E5\_CANLF, tr\|E2RRA9\|E2RRA9\_CANLF, tr\|F1P874\|F1P874\_CANLF, tr\|F1PF03\|F1PF03\_CANLF, tr\|F1PGB1\|F1PGB1\_CANLF, tr\|F1PKU7\|F1PKU7\_CANLF, tr\|F1Q1D5\|F1Q1D5\_CANLF, tr\|F1Q2Y3\|F1Q2Y3\_CANLF, tr\|J9P1X5\|J9P1X5\_CANLF, tr\|J9P4L9\|J9P4L9\_CANLF, tr\|Q45KI9\|Q45KI9\_CANLF |
| 90 | Chagas disease | sp\|P38400\|GNAI2\_CANLF, sp\|P63091\|GNAS\_CANLF, tr\|A0A5F4BVY4\|A0A5F4BVY4\_CANLF, tr\|A0A5F4C5E5\|A0A5F4C5E5\_CANLF, tr\|E2RRA9\|E2RRA9\_CANLF, tr\|F1PCV3\|F1PCV3\_CANLF, tr\|F1PGM1\|F1PGM1\_CANLF, tr\|F1PIK5\|F1PIK5\_CANLF, tr\|F1PX75\|F1PX75\_CANLF, tr\|F1PXM5\|F1PXM5\_CANLF, tr\|F6UYJ9\|F6UYJ9\_CANLF, tr\|J9NSZ5\|J9NSZ5\_CANLF, tr\|J9P0L7\|J9P0L7\_CANLF, tr\|J9P1X5\|J9P1X5\_CANLF |
| 91 | Acute myeloid leukemia | sp\|Q1HG70\|MP2K2\_CANLF, tr\|A0A140T8E6\|A0A140T8E6\_CANLF, tr\|A0A5F4C4S3\|A0A5F4C4S3\_CANLF, tr\|A0A5F4C566\|A0A5F4C566\_CANLF, tr\|A0A5F4C5D6\|A0A5F4C5D6\_CANLF, tr\|A0A5F4C5E5\|A0A5F4C5E5\_CANLF, tr\|A0A5F4CC58\|A0A5F4CC58\_CANLF, tr\|E2RPA3\|E2RPA3\_CANLF, tr\|E2RRA9\|E2RRA9\_CANLF, tr\|F1P874\|F1P874\_CANLF, tr\|F1PNW8\|F1PNW8\_CANLF, tr\|J9P1X5\|J9P1X5\_CANLF, tr\|J9P4L9\|J9P4L9\_CANLF, tr\|Q45KI9\|Q45KI9\_CANLF |
| 92 | Prion diseases | sp\|Q1HG70\|MP2K2\_CANLF, sp\|Q8WNN6\|SODC\_CANLF, tr\|A0A5F4C5E5\|A0A5F4C5E5\_CANLF, tr\|A0A5F4CJ74\|A0A5F4CJ74\_CANLF, tr\|E2QZQ6\|E2QZQ6\_CANLF, tr\|E2R9N5\|E2R9N5\_CANLF, tr\|E2RC09\|E2RC09\_CANLF, tr\|F1P7V6\|F1P7V6\_CANLF, tr\|F1PIC7\|F1PIC7\_CANLF, tr\|F1PS94\|F1PS94\_CANLF, tr\|F6XMP7\|F6XMP7\_CANLF, tr\|G1K268\|G1K268\_CANLF, tr\|H9GW49\|H9GW49\_CANLF, tr\|J9NXT4\|J9NXT4\_CANLF |
| 93 | Vibrio cholerae infection | sp\|O18840\|ACTB\_CANLF, sp\|O97758\|ZO1\_CANLF, sp\|P63091\|GNAS\_CANLF, tr\|E2QXJ5\|E2QXJ5\_CANLF, tr\|E2QXY7\|E2QXY7\_CANLF, tr\|E2R7L1\|E2R7L1\_CANLF, tr\|E2RK67\|E2RK67\_CANLF, tr\|F1PKU7\|F1PKU7\_CANLF, tr\|F1Q1D5\|F1Q1D5\_CANLF, tr\|J9NXT4\|J9NXT4\_CANLF, tr\|J9P749\|J9P749\_CANLF, tr\|J9PAK3\|J9PAK3\_CANLF, tr\|Z4YHI2\|Z4YHI2\_CANLF |
| 94 | Phototransduction - fly | sp\|O18840\|ACTB\_CANLF, tr\|A0A5F4BVY4\|A0A5F4BVY4\_CANLF, tr\|A0A5F4CJ79\|A0A5F4CJ79\_CANLF, tr\|E2QXY7\|E2QXY7\_CANLF, tr\|E2RG56\|E2RG56\_CANLF, tr\|F1PKU7\|F1PKU7\_CANLF, tr\|F1PXM5\|F1PXM5\_CANLF, tr\|F1Q1D5\|F1Q1D5\_CANLF, tr\|G1K2D5\|G1K2D5\_CANLF, tr\|J9NWJ7\|J9NWJ7\_CANLF, tr\|J9P8S3\|J9P8S3\_CANLF, tr\|J9PAK3\|J9PAK3\_CANLF, tr\|Z4YHI2\|Z4YHI2\_CANLF |
| 95 | TGF-beta signaling pathway | sp\|Q29393\|PGS2\_CANLF, tr\|A0A5F4C5E5\|A0A5F4C5E5\_CANLF, tr\|A0A5F4D5V1\|A0A5F4D5V1\_CANLF, tr\|A0A5F4D9H1\|A0A5F4D9H1\_CANLF, tr\|E2R612\|E2R612\_CANLF, tr\|E2RIG3\|E2RIG3\_CANLF, tr\|E2RKY7\|E2RKY7\_CANLF, tr\|E2RRW0\|E2RRW0\_CANLF, tr\|F1PCC1\|F1PCC1\_CANLF, tr\|F1PX75\|F1PX75\_CANLF, tr\|F1Q4I3\|F1Q4I3\_CANLF, tr\|F6X9Y6\|F6X9Y6\_CANLF, tr\|F6XY66\|F6XY66\_CANLF |
| 96 | Melanoma | sp\|F1PAA9\|CADH1\_CANLF, sp\|Q1HG70\|MP2K2\_CANLF, tr\|A0A5F4C5E5\|A0A5F4C5E5\_CANLF, tr\|A0A5F4CT67\|A0A5F4CT67\_CANLF, tr\|A0A5F4D6R1\|A0A5F4D6R1\_CANLF, tr\|E2RRA9\|E2RRA9\_CANLF, tr\|F1P874\|F1P874\_CANLF, tr\|F1PF03\|F1PF03\_CANLF, tr\|F1PKR8\|F1PKR8\_CANLF, tr\|J9P1X5\|J9P1X5\_CANLF, tr\|J9P4L9\|J9P4L9\_CANLF, tr\|Q45KI9\|Q45KI9\_CANLF |
| 97 | Malaria | sp\|P02727\|GLPA\_CANLF, tr\|A0A5F4CZG6\|A0A5F4CZG6\_CANLF, tr\|A0A5F4DBL7\|A0A5F4DBL7\_CANLF, tr\|A0A5F4DDY0\|A0A5F4DDY0\_CANLF, tr\|A7VLI5\|A7VLI5\_CANLF, tr\|E2R629\|E2R629\_CANLF, tr\|F1PB66\|F1PB66\_CANLF, tr\|F1PB95\|F1PB95\_CANLF, tr\|F1PYL8\|F1PYL8\_CANLF, tr\|F1PYZ1\|F1PYZ1\_CANLF, tr\|F6X9Y6\|F6X9Y6\_CANLF, tr\|J9NXL3\|J9NXL3\_CANLF |
| 98 | p53 signaling pathway | tr\|A0A5F4C8G1\|A0A5F4C8G1\_CANLF, tr\|A0A5F4CE07\|A0A5F4CE07\_CANLF, tr\|A0A5F4CNS5\|A0A5F4CNS5\_CANLF, tr\|A0A5F4CT67\|A0A5F4CT67\_CANLF, tr\|A0A5F4D6R1\|A0A5F4D6R1\_CANLF, tr\|A0A5F4DJ19\|A0A5F4DJ19\_CANLF, tr\|E2QY29\|E2QY29\_CANLF, tr\|E2RJG8\|E2RJG8\_CANLF, tr\|F1P7Q1\|F1P7Q1\_CANLF, tr\|F1PKR8\|F1PKR8\_CANLF, tr\|F6X9Y6\|F6X9Y6\_CANLF, tr\|J9P9V8\|J9P9V8\_CANLF |
| 99 | Pentose phosphate pathway | tr\|A0A5F4BUZ8\|A0A5F4BUZ8\_CANLF, tr\|A0A5F4CVL0\|A0A5F4CVL0\_CANLF, tr\|E2QV12\|E2QV12\_CANLF, tr\|E2QVM3\|E2QVM3\_CANLF, tr\|E2R2C3\|E2R2C3\_CANLF, tr\|E2REJ7\|E2REJ7\_CANLF, tr\|F1PBZ5\|F1PBZ5\_CANLF, tr\|F1PE09\|F1PE09\_CANLF, tr\|F1PYB6\|F1PYB6\_CANLF, tr\|F1Q3S9\|F1Q3S9\_CANLF, tr\|H9GW87\|H9GW87\_CANLF, tr\|J9P7A6\|J9P7A6\_CANLF |
| 100 | Lysine degradation | tr\|A0A5F4C6G2\|A0A5F4C6G2\_CANLF, tr\|A0A5F4D491\|A0A5F4D491\_CANLF, tr\|A0A5F4DCU7\|A0A5F4DCU7\_CANLF, tr\|A0A5F4DJ33\|A0A5F4DJ33\_CANLF, tr\|A0A5F4DNA4\|A0A5F4DNA4\_CANLF, tr\|E2RQ99\|E2RQ99\_CANLF, tr\|F1PAB7\|F1PAB7\_CANLF, tr\|F1PAZ6\|F1PAZ6\_CANLF, tr\|F1PIP0\|F1PIP0\_CANLF, tr\|F6UMN8\|F6UMN8\_CANLF, tr\|F6XHT8\|F6XHT8\_CANLF, tr\|J9JHD4\|J9JHD4\_CANLF |
| 101 | Adipocytokine signaling pathway | tr\|A0A5F4BRD0\|A0A5F4BRD0\_CANLF, tr\|A0A5F4CC58\|A0A5F4CC58\_CANLF, tr\|A0A5F4CD52\|A0A5F4CD52\_CANLF, tr\|A0A5F4CFR9\|A0A5F4CFR9\_CANLF, tr\|E2R3M4\|E2R3M4\_CANLF, tr\|E2R3S0\|E2R3S0\_CANLF, tr\|E2RGU7\|E2RGU7\_CANLF, tr\|E2RNP2\|E2RNP2\_CANLF, tr\|F1PDX2\|F1PDX2\_CANLF, tr\|J9P1N8\|J9P1N8\_CANLF, tr\|J9P5F9\|J9P5F9\_CANLF, tr\|J9P9G0\|J9P9G0\_CANLF |
| 102 | Jak-STAT signaling pathway | tr\|A0A5F4BRD0\|A0A5F4BRD0\_CANLF, tr\|A0A5F4C4S3\|A0A5F4C4S3\_CANLF, tr\|A0A5F4C566\|A0A5F4C566\_CANLF, tr\|A0A5F4CC58\|A0A5F4CC58\_CANLF, tr\|E2QXW0\|E2QXW0\_CANLF, tr\|E2RI99\|E2RI99\_CANLF, tr\|E2RM84\|E2RM84\_CANLF, tr\|E2RRA9\|E2RRA9\_CANLF, tr\|F1PNW8\|F1PNW8\_CANLF, tr\|F1PQ60\|F1PQ60\_CANLF, tr\|F1PTB2\|F1PTB2\_CANLF, tr\|J9P1X5\|J9P1X5\_CANLF |
| 103 | Citrate cycle (TCA cycle) | tr\|A0A5F4C6G2\|A0A5F4C6G2\_CANLF, tr\|A0A5F4D491\|A0A5F4D491\_CANLF, tr\|A0A5F4D8P8\|A0A5F4D8P8\_CANLF, tr\|A0A5F4DIX0\|A0A5F4DIX0\_CANLF, tr\|E2QV08\|E2QV08\_CANLF, tr\|E2RGR9\|E2RGR9\_CANLF, tr\|F1PV92\|F1PV92\_CANLF, tr\|F1PYG8\|F1PYG8\_CANLF, tr\|F1PZA1\|F1PZA1\_CANLF, tr\|F1Q3V2\|F1Q3V2\_CANLF, tr\|F1Q3W8\|F1Q3W8\_CANLF, tr\|J9P208\|J9P208\_CANLF |
| 104 | Amyotrophic lateral sclerosis (ALS) | sp\|O97492\|CATA\_CANLF, sp\|Q8WNN6\|SODC\_CANLF, tr\|A0A5F4CE07\|A0A5F4CE07\_CANLF, tr\|A0A5F4DH40\|A0A5F4DH40\_CANLF, tr\|E2RJG8\|E2RJG8\_CANLF, tr\|E2RSG1\|E2RSG1\_CANLF, tr\|F1PRZ2\|F1PRZ2\_CANLF, tr\|F1PYQ1\|F1PYQ1\_CANLF, tr\|F2Z4P5\|F2Z4P5\_CANLF, tr\|F6XH46\|F6XH46\_CANLF, tr\|J9P028\|J9P028\_CANLF, tr\|Q45KI9\|Q45KI9\_CANLF |
| 105 | Amino sugar and nucleotide sugar metabolism | tr\|A0A5F4C483\|A0A5F4C483\_CANLF, tr\|A0A5F4CVL0\|A0A5F4CVL0\_CANLF, tr\|E2QV12\|E2QV12\_CANLF, tr\|E2R2C3\|E2R2C3\_CANLF, tr\|E2R601\|E2R601\_CANLF, tr\|F1PBZ5\|F1PBZ5\_CANLF, tr\|F1PPF7\|F1PPF7\_CANLF, tr\|F1Q0H0\|F1Q0H0\_CANLF, tr\|J9P2E3\|J9P2E3\_CANLF, tr\|J9P432\|J9P432\_CANLF, tr\|K0J6C5\|K0J6C5\_CANLF |
| 106 | Propanoate metabolism | tr\|A0A5F4CZB6\|A0A5F4CZB6\_CANLF, tr\|E2RQ99\|E2RQ99\_CANLF, tr\|F1P6W6\|F1P6W6\_CANLF, tr\|F1PAB7\|F1PAB7\_CANLF, tr\|F1PAZ6\|F1PAZ6\_CANLF, tr\|F1PIP0\|F1PIP0\_CANLF, tr\|F1PVW0\|F1PVW0\_CANLF, tr\|J9JHD4\|J9JHD4\_CANLF, tr\|J9P2I9\|J9P2I9\_CANLF, tr\|J9P3N9\|J9P3N9\_CANLF, tr\|J9P7N4\|J9P7N4\_CANLF |
| 107 | Colorectal cancer | tr\|A0A5F4C5E5\|A0A5F4C5E5\_CANLF, tr\|A0A5F4D0Z3\|A0A5F4D0Z3\_CANLF, tr\|E2R6J0\|E2R6J0\_CANLF, tr\|E2RRA9\|E2RRA9\_CANLF, tr\|F1P874\|F1P874\_CANLF, tr\|F1PS09\|F1PS09\_CANLF, tr\|F1PYQ1\|F1PYQ1\_CANLF, tr\|F6XY66\|F6XY66\_CANLF, tr\|J9P1X5\|J9P1X5\_CANLF, tr\|J9P4L9\|J9P4L9\_CANLF, tr\|Q45KI9\|Q45KI9\_CANLF |
| 108 | Epithelial cell signaling in Helicobacter pylori infection | sp\|O97758\|ZO1\_CANLF, tr\|A0A5F4D4H5\|A0A5F4D4H5\_CANLF, tr\|A0A5F4D5V0\|A0A5F4D5V0\_CANLF, tr\|E2QXJ5\|E2QXJ5\_CANLF, tr\|E2REH3\|E2REH3\_CANLF, tr\|E2RI72\|E2RI72\_CANLF, tr\|F1PF03\|F1PF03\_CANLF, tr\|F1PJP7\|F1PJP7\_CANLF, tr\|F1PP71\|F1PP71\_CANLF, tr\|F1PUS0\|F1PUS0\_CANLF, tr\|F1PYQ1\|F1PYQ1\_CANLF |
| 109 | Aminoacyl-tRNA biosynthesis | sp\|P49822\|ALBU\_CANLF, tr\|A0A5F4CTT0\|A0A5F4CTT0\_CANLF, tr\|A0A5F4DE71\|A0A5F4DE71\_CANLF, tr\|A0A5F4DIX8\|A0A5F4DIX8\_CANLF, tr\|E2RGG9\|E2RGG9\_CANLF, tr\|E2RNQ8\|E2RNQ8\_CANLF, tr\|E2RTJ7\|E2RTJ7\_CANLF, tr\|F1PYM5\|F1PYM5\_CANLF, tr\|F1Q332\|F1Q332\_CANLF, tr\|F6USV0\|F6USV0\_CANLF, tr\|J9P440\|J9P440\_CANLF |
| 110 | Proteasome | tr\|E2QUY7\|E2QUY7\_CANLF, tr\|E2R0B6\|E2R0B6\_CANLF, tr\|E2RCP9\|E2RCP9\_CANLF, tr\|E2RJ26\|E2RJ26\_CANLF, tr\|F1PD77\|F1PD77\_CANLF, tr\|F1PF02\|F1PF02\_CANLF, tr\|F1PPH7\|F1PPH7\_CANLF, tr\|F1PRW0\|F1PRW0\_CANLF, tr\|F6UY11\|F6UY11\_CANLF, tr\|J9JHW4\|J9JHW4\_CANLF, tr\|J9P849\|J9P849\_CANLF |
| 111 | Neuroactive ligand-receptor interaction | tr\|A0A5F4C280\|A0A5F4C280\_CANLF, tr\|A0A5F4D662\|A0A5F4D662\_CANLF, tr\|E2QVG6\|E2QVG6\_CANLF, tr\|F1P7Q6\|F1P7Q6\_CANLF, tr\|F1P9Y2\|F1P9Y2\_CANLF, tr\|F1PCE8\|F1PCE8\_CANLF, tr\|F1PI78\|F1PI78\_CANLF, tr\|F1PRW2\|F1PRW2\_CANLF, tr\|F1PZ92\|F1PZ92\_CANLF, tr\|F1Q298\|F1Q298\_CANLF |
| 112 | Tryptophan metabolism | sp\|O97492\|CATA\_CANLF, tr\|A0A5F4C6G2\|A0A5F4C6G2\_CANLF, tr\|E2R1E8\|E2R1E8\_CANLF, tr\|E2RGG9\|E2RGG9\_CANLF, tr\|E2RQ99\|E2RQ99\_CANLF, tr\|F1PAB7\|F1PAB7\_CANLF, tr\|F1PAZ6\|F1PAZ6\_CANLF, tr\|F1PIP0\|F1PIP0\_CANLF, tr\|F6XHT8\|F6XHT8\_CANLF, tr\|J9JHD4\|J9JHD4\_CANLF |
| 113 | Glycerophospholipid metabolism | tr\|A0A5F4BU16\|A0A5F4BU16\_CANLF, tr\|A0A5F4C8H3\|A0A5F4C8H3\_CANLF, tr\|A0A5F4D0K7\|A0A5F4D0K7\_CANLF, tr\|A0A5F4DFU1\|A0A5F4DFU1\_CANLF, tr\|E2R185\|E2R185\_CANLF, tr\|E2RRI3\|E2RRI3\_CANLF, tr\|F1Q2H0\|F1Q2H0\_CANLF, tr\|F6UQ33\|F6UQ33\_CANLF, tr\|J9P5F5\|J9P5F5\_CANLF, tr\|J9P6F5\|J9P6F5\_CANLF |
| 114 | Fatty acid metabolism | tr\|E2QXA3\|E2QXA3\_CANLF, tr\|E2RQ99\|E2RQ99\_CANLF, tr\|F1P6W6\|F1P6W6\_CANLF, tr\|F1PAB7\|F1PAB7\_CANLF, tr\|F1PAZ6\|F1PAZ6\_CANLF, tr\|F1PIP0\|F1PIP0\_CANLF, tr\|F6XHT8\|F6XHT8\_CANLF, tr\|J9JHD4\|J9JHD4\_CANLF, tr\|J9NZC6\|J9NZC6\_CANLF, tr\|J9P9G0\|J9P9G0\_CANLF |
| 115 | Arginine and proline metabolism | sp\|P05124\|KCRB\_CANLF, tr\|A0A5F4BUE3\|A0A5F4BUE3\_CANLF, tr\|A0A5F4C1A4\|A0A5F4C1A4\_CANLF, tr\|A0A5F4CHX9\|A0A5F4CHX9\_CANLF, tr\|A0A5F4CNU9\|A0A5F4CNU9\_CANLF, tr\|A0A5F4D8F3\|A0A5F4D8F3\_CANLF, tr\|E2RQ99\|E2RQ99\_CANLF, tr\|F1PAB7\|F1PAB7\_CANLF, tr\|F1PF82\|F1PF82\_CANLF, tr\|F1PPD7\|F1PPD7\_CANLF |
| 116 | Primary immunodeficiency | tr\|A0A5F4CBH6\|A0A5F4CBH6\_CANLF, tr\|E2RCC8\|E2RCC8\_CANLF, tr\|E2RE31\|E2RE31\_CANLF, tr\|F1PKY3\|F1PKY3\_CANLF, tr\|F1Q1S4\|F1Q1S4\_CANLF, tr\|F1Q2F5\|F1Q2F5\_CANLF, tr\|H9GWR8\|H9GWR8\_CANLF, tr\|L7N0K1\|L7N0K1\_CANLF, tr\|Q5W417\|Q5W417\_CANLF |
| 117 | Galactose metabolism | tr\|A0A5F4C483\|A0A5F4C483\_CANLF, tr\|A0A5F4CVL0\|A0A5F4CVL0\_CANLF, tr\|E2QV12\|E2QV12\_CANLF, tr\|F1PBZ5\|F1PBZ5\_CANLF, tr\|F1PGZ1\|F1PGZ1\_CANLF, tr\|F1PPF7\|F1PPF7\_CANLF, tr\|F1PWH9\|F1PWH9\_CANLF, tr\|F1Q3S9\|F1Q3S9\_CANLF, tr\|J9P7R2\|J9P7R2\_CANLF |
| 118 | Dorso-ventral axis formation | tr\|A0A5F4BYQ8\|A0A5F4BYQ8\_CANLF, tr\|A0A5F4C4S3\|A0A5F4C4S3\_CANLF, tr\|A0A5F4C566\|A0A5F4C566\_CANLF, tr\|A0A5F4C5E5\|A0A5F4C5E5\_CANLF, tr\|A0A5F4CNN3\|A0A5F4CNN3\_CANLF, tr\|E2RSQ3\|E2RSQ3\_CANLF, tr\|F1PF03\|F1PF03\_CANLF, tr\|F1PS94\|F1PS94\_CANLF, tr\|J9P3Q9\|J9P3Q9\_CANLF |
| 119 | Thyroid cancer | sp\|F1PAA9\|CADH1\_CANLF, sp\|Q1HG70\|MP2K2\_CANLF, tr\|A0A5F4C5E5\|A0A5F4C5E5\_CANLF, tr\|A0A5F4D0Z3\|A0A5F4D0Z3\_CANLF, tr\|A0A5F4D721\|A0A5F4D721\_CANLF, tr\|A0A5F4DCE0\|A0A5F4DCE0\_CANLF, tr\|E2REP2\|E2REP2\_CANLF, tr\|E2RQX1\|E2RQX1\_CANLF, tr\|F1P874\|F1P874\_CANLF |
| 120 | Aldosterone-regulated sodium reabsorption | tr\|A0A5F4C5E5\|A0A5F4C5E5\_CANLF, tr\|A0A5F4CUL0\|A0A5F4CUL0\_CANLF, tr\|E2RRA9\|E2RRA9\_CANLF, tr\|F1PBA1\|F1PBA1\_CANLF, tr\|F1PKU7\|F1PKU7\_CANLF, tr\|F1Q039\|F1Q039\_CANLF, tr\|F1Q1D5\|F1Q1D5\_CANLF, tr\|F1Q2Y3\|F1Q2Y3\_CANLF, tr\|J9P1X5\|J9P1X5\_CANLF |
| 121 | Bladder cancer | sp\|F1PAA9\|CADH1\_CANLF, sp\|Q1HG70\|MP2K2\_CANLF, tr\|A0A5F4C5E5\|A0A5F4C5E5\_CANLF, tr\|E2RA06\|E2RA06\_CANLF, tr\|F1P874\|F1P874\_CANLF, tr\|F1PF03\|F1PF03\_CANLF, tr\|F1PPM7\|F1PPM7\_CANLF, tr\|F6X9Y6\|F6X9Y6\_CANLF, tr\|J9P4L9\|J9P4L9\_CANLF |
| 122 | Notch signaling pathway | tr\|A0A5F4BVA2\|A0A5F4BVA2\_CANLF, tr\|A0A5K1UA11\|A0A5K1UA11\_CANLF, tr\|F1PDW7\|F1PDW7\_CANLF, tr\|F1PEV3\|F1PEV3\_CANLF, tr\|F1PKX6\|F1PKX6\_CANLF, tr\|F1PR63\|F1PR63\_CANLF, tr\|F1PS94\|F1PS94\_CANLF, tr\|F6UX32\|F6UX32\_CANLF, tr\|J9P334\|J9P334\_CANLF |
| 123 | Type II diabetes mellitus | tr\|A0A5F4C5E5\|A0A5F4C5E5\_CANLF, tr\|A0A5F4DKH0\|A0A5F4DKH0\_CANLF, tr\|E2RGU7\|E2RGU7\_CANLF, tr\|E2RNP2\|E2RNP2\_CANLF, tr\|E2RRA9\|E2RRA9\_CANLF, tr\|F1PDX2\|F1PDX2\_CANLF, tr\|F1PPF7\|F1PPF7\_CANLF, tr\|J9P1X5\|J9P1X5\_CANLF, tr\|J9P5F9\|J9P5F9\_CANLF |
| 124 | Alanine, aspartate and glutamate metabolism | tr\|A0A5F4BUE3\|A0A5F4BUE3\_CANLF, tr\|A0A5F4CHX9\|A0A5F4CHX9\_CANLF, tr\|A0A5F4CNU9\|A0A5F4CNU9\_CANLF, tr\|F1PAL6\|F1PAL6\_CANLF, tr\|F1PP35\|F1PP35\_CANLF, tr\|F1PPD7\|F1PPD7\_CANLF, tr\|F1PRF8\|F1PRF8\_CANLF, tr\|J9JHC8\|J9JHC8\_CANLF, tr\|J9P432\|J9P432\_CANLF |
| 125 | Pyrimidine metabolism | tr\|A0A5F4CWL0\|A0A5F4CWL0\_CANLF, tr\|A0A5F4D0K7\|A0A5F4D0K7\_CANLF, tr\|A0A5F4DIU7\|A0A5F4DIU7\_CANLF, tr\|E2R3I2\|E2R3I2\_CANLF, tr\|E2R8N8\|E2R8N8\_CANLF, tr\|E2RC20\|E2RC20\_CANLF, tr\|E2RD29\|E2RD29\_CANLF, tr\|F1PQM1\|F1PQM1\_CANLF, tr\|F1PY94\|F1PY94\_CANLF |
| 126 | RIG-I-like receptor signaling pathway | tr\|A0A5F4CDU8\|A0A5F4CDU8\_CANLF, tr\|A0A5F4CFL1\|A0A5F4CFL1\_CANLF, tr\|E2R3R9\|E2R3R9\_CANLF, tr\|E2R3V7\|E2R3V7\_CANLF, tr\|E2RA47\|E2RA47\_CANLF, tr\|E2RMV6\|E2RMV6\_CANLF, tr\|F1PIK5\|F1PIK5\_CANLF, tr\|J9P0U9\|J9P0U9\_CANLF |
| 127 | beta-Alanine metabolism | tr\|A0A5F4DCR7\|A0A5F4DCR7\_CANLF, tr\|E2RQ99\|E2RQ99\_CANLF, tr\|F1P6W6\|F1P6W6\_CANLF, tr\|F1PAB7\|F1PAB7\_CANLF, tr\|F1PAZ6\|F1PAZ6\_CANLF, tr\|F1PF82\|F1PF82\_CANLF, tr\|F1PIP0\|F1PIP0\_CANLF, tr\|F1PRF8\|F1PRF8\_CANLF |
| 128 | Tyrosine metabolism | tr\|A0A5F4BUE3\|A0A5F4BUE3\_CANLF, tr\|A0A5F4CNR7\|A0A5F4CNR7\_CANLF, tr\|A0A5F4CNU9\|A0A5F4CNU9\_CANLF, tr\|A0A5F4D6M3\|A0A5F4D6M3\_CANLF, tr\|A0A5F4DCR7\|A0A5F4DCR7\_CANLF, tr\|F1PXN6\|F1PXN6\_CANLF, tr\|F1Q0J0\|F1Q0J0\_CANLF, tr\|Q60F99\|Q60F99\_CANLF |
| 129 | Butanoate metabolism | tr\|F1PAZ6\|F1PAZ6\_CANLF, tr\|F1PIP0\|F1PIP0\_CANLF, tr\|F1PJ13\|F1PJ13\_CANLF, tr\|F1PP35\|F1PP35\_CANLF, tr\|F1PRF8\|F1PRF8\_CANLF, tr\|F6XHT8\|F6XHT8\_CANLF, tr\|J9JHD4\|J9JHD4\_CANLF, tr\|J9P208\|J9P208\_CANLF |
| 130 | Glycerolipid metabolism | tr\|A0A5F4BU16\|A0A5F4BU16\_CANLF, tr\|A0A5F4C8H3\|A0A5F4C8H3\_CANLF, tr\|E2RQ99\|E2RQ99\_CANLF, tr\|E2RRI3\|E2RRI3\_CANLF, tr\|F1PAB7\|F1PAB7\_CANLF, tr\|F1PK43\|F1PK43\_CANLF, tr\|J9P6F5\|J9P6F5\_CANLF, tr\|J9P7R2\|J9P7R2\_CANLF |
| 131 | Proximal tubule bicarbonate reclamation | sp\|Q9N2J4\|AQP1\_CANLF, tr\|A0A5F4CHX9\|A0A5F4CHX9\_CANLF, tr\|A0A5F4D4M8\|A0A5F4D4M8\_CANLF, tr\|A0A5F4D507\|A0A5F4D507\_CANLF, tr\|E2QV08\|E2QV08\_CANLF, tr\|F1PBA1\|F1PBA1\_CANLF, tr\|F1PDY8\|F1PDY8\_CANLF, tr\|F1PPD7\|F1PPD7\_CANLF |
| 132 | Olfactory transduction | tr\|A0A5F4CJ79\|A0A5F4CJ79\_CANLF, tr\|A0A5F4CKN4\|A0A5F4CKN4\_CANLF, tr\|E2RG56\|E2RG56\_CANLF, tr\|G1K2D5\|G1K2D5\_CANLF, tr\|J9NWJ7\|J9NWJ7\_CANLF, tr\|J9NXT4\|J9NXT4\_CANLF, tr\|J9NY42\|J9NY42\_CANLF, tr\|J9P8S3\|J9P8S3\_CANLF |
| 133 | Cysteine and methionine metabolism | tr\|A0A5F4BUE3\|A0A5F4BUE3\_CANLF, tr\|A0A5F4CNU9\|A0A5F4CNU9\_CANLF, tr\|A0A5F4DF70\|A0A5F4DF70\_CANLF, tr\|F1PF82\|F1PF82\_CANLF, tr\|F1PMQ1\|F1PMQ1\_CANLF, tr\|F1PVW0\|F1PVW0\_CANLF, tr\|J9P3N9\|J9P3N9\_CANLF |
| 134 | Autoimmune thyroid disease | tr\|A0A5F4BUG1\|A0A5F4BUG1\_CANLF, tr\|A0A5F4CBH6\|A0A5F4CBH6\_CANLF, tr\|E2RCC8\|E2RCC8\_CANLF, tr\|E2RE31\|E2RE31\_CANLF, tr\|O46880\|O46880\_CANLF, tr\|O46882\|O46882\_CANLF, tr\|Q30348\|Q30348\_CANLF |
| 135 | Allograft rejection | tr\|A0A5F4BUG1\|A0A5F4BUG1\_CANLF, tr\|A0A5F4CBH6\|A0A5F4CBH6\_CANLF, tr\|E2RCC8\|E2RCC8\_CANLF, tr\|E2RE31\|E2RE31\_CANLF, tr\|O46880\|O46880\_CANLF, tr\|O46882\|O46882\_CANLF, tr\|Q30348\|Q30348\_CANLF |
| 136 | Drug metabolism - cytochrome P450 | sp\|Q95LA2\|FMO1\_CANLF, tr\|A0A5F4CNR7\|A0A5F4CNR7\_CANLF, tr\|A0A5F4D6M3\|A0A5F4D6M3\_CANLF, tr\|E2RHC8\|E2RHC8\_CANLF, tr\|F1PXN6\|F1PXN6\_CANLF, tr\|F1Q0J0\|F1Q0J0\_CANLF, tr\|J9NRV1\|J9NRV1\_CANLF |
| 137 | Starch and sucrose metabolism | tr\|A0A5F4CVL0\|A0A5F4CVL0\_CANLF, tr\|E2QV12\|E2QV12\_CANLF, tr\|E2R2C3\|E2R2C3\_CANLF, tr\|E2RLM7\|E2RLM7\_CANLF, tr\|F1PBZ5\|F1PBZ5\_CANLF, tr\|F1PPF7\|F1PPF7\_CANLF, tr\|F1PSM2\|F1PSM2\_CANLF |
| 138 | Carbohydrate digestion and absorption | tr\|A0A5F4BVY4\|A0A5F4BVY4\_CANLF, tr\|E2RRA9\|E2RRA9\_CANLF, tr\|F1PBA1\|F1PBA1\_CANLF, tr\|F1PKU7\|F1PKU7\_CANLF, tr\|F1PPF7\|F1PPF7\_CANLF, tr\|F1Q1D5\|F1Q1D5\_CANLF, tr\|J9P1X5\|J9P1X5\_CANLF |
| 139 | Intestinal immune network for IgA production | tr\|A0A5F4BUG1\|A0A5F4BUG1\_CANLF, tr\|A0A5F4CBH6\|A0A5F4CBH6\_CANLF, tr\|E2RCC8\|E2RCC8\_CANLF, tr\|E2RE31\|E2RE31\_CANLF, tr\|F1PQ29\|F1PQ29\_CANLF, tr\|F1Q2F5\|F1Q2F5\_CANLF, tr\|Q30348\|Q30348\_CANLF |
| 140 | SNARE interactions in vesicular transport | tr\|A0A5F4C0Q0\|A0A5F4C0Q0\_CANLF, tr\|A0A5F4D5G0\|A0A5F4D5G0\_CANLF, tr\|A0A5F4DBI9\|A0A5F4DBI9\_CANLF, tr\|E2R4Q2\|E2R4Q2\_CANLF, tr\|E2R516\|E2R516\_CANLF, tr\|F1P8F6\|F1P8F6\_CANLF, tr\|J9NRK3\|J9NRK3\_CANLF |
| 141 | Taste transduction | sp\|P62872\|GBB1\_CANLF, sp\|P63091\|GNAS\_CANLF, tr\|A0A5F4BVY4\|A0A5F4BVY4\_CANLF, tr\|A0A5F4CKN4\|A0A5F4CKN4\_CANLF, tr\|A0A5F4CNW6\|A0A5F4CNW6\_CANLF, tr\|J9NXT4\|J9NXT4\_CANLF, tr\|J9P2M3\|J9P2M3\_CANLF |
| 142 | N-Glycan biosynthesis | sp\|E2RG47\|STT3B\_CANLF, sp\|E2RQ08\|RPN1\_CANLF, tr\|A0A5F4CVB8\|A0A5F4CVB8\_CANLF, tr\|A0A5F4D0J5\|A0A5F4D0J5\_CANLF, tr\|E2R8C9\|E2R8C9\_CANLF, tr\|F1PGZ1\|F1PGZ1\_CANLF |
| 143 | Cytosolic DNA-sensing pathway | tr\|A0A5F4CDU8\|A0A5F4CDU8\_CANLF, tr\|E2R3V7\|E2R3V7\_CANLF, tr\|E2R8N8\|E2R8N8\_CANLF, tr\|E2RMV6\|E2RMV6\_CANLF, tr\|J9P0U9\|J9P0U9\_CANLF, tr\|J9P4M2\|J9P4M2\_CANLF |
| 144 | Metabolism of xenobiotics by cytochrome P450 | tr\|A0A5F4CNR7\|A0A5F4CNR7\_CANLF, tr\|A0A5F4D6M3\|A0A5F4D6M3\_CANLF, tr\|F1PXN6\|F1PXN6\_CANLF, tr\|F1Q0J0\|F1Q0J0\_CANLF, tr\|F1Q102\|F1Q102\_CANLF, tr\|J9NRV1\|J9NRV1\_CANLF |
| 145 | Phototransduction | sp\|P62872\|GBB1\_CANLF, tr\|G1K2D5\|G1K2D5\_CANLF, tr\|J9NWJ7\|J9NWJ7\_CANLF, tr\|J9NY42\|J9NY42\_CANLF, tr\|J9P211\|J9P211\_CANLF, tr\|J9P8S3\|J9P8S3\_CANLF |
| 146 | Riboflavin metabolism (no map in kegg database) | tr\|A0A5F4DAB6\|A0A5F4DAB6\_CANLF, tr\|E2R9U0\|E2R9U0\_CANLF, tr\|E2RLF5\|E2RLF5\_CANLF, tr\|F1PJD1\|F1PJD1\_CANLF, tr\|F1PKG8\|F1PKG8\_CANLF, tr\|F1PU81\|F1PU81\_CANLF |
| 147 | Type I diabetes mellitus | tr\|A0A5F4BUG1\|A0A5F4BUG1\_CANLF, tr\|E2QUU5\|E2QUU5\_CANLF, tr\|F1PRF8\|F1PRF8\_CANLF, tr\|O46880\|O46880\_CANLF, tr\|O46882\|O46882\_CANLF, tr\|Q30348\|Q30348\_CANLF |
| 148 | Nicotinate and nicotinamide metabolism | tr\|F1PLG2\|F1PLG2\_CANLF, tr\|F1PQM1\|F1PQM1\_CANLF, tr\|F1PY94\|F1PY94\_CANLF, tr\|F1Q3X6\|F1Q3X6\_CANLF, tr\|J9P111\|J9P111\_CANLF, tr\|J9P988\|J9P988\_CANLF |
| 149 | Fructose and mannose metabolism | tr\|A0A346M2B2\|A0A346M2B2\_CANLF, tr\|F1PKG8\|F1PKG8\_CANLF, tr\|F1PPF7\|F1PPF7\_CANLF, tr\|F1Q3S9\|F1Q3S9\_CANLF, tr\|J9P7A6\|J9P7A6\_CANLF, tr\|J9P7R2\|J9P7R2\_CANLF |
| 150 | MAPK signaling pathway - fly | tr\|A0A5F4C4S3\|A0A5F4C4S3\_CANLF, tr\|A0A5F4C566\|A0A5F4C566\_CANLF, tr\|A0A5F4C5E5\|A0A5F4C5E5\_CANLF, tr\|E2QUY1\|E2QUY1\_CANLF, tr\|F1PF03\|F1PF03\_CANLF, tr\|F6UT62\|F6UT62\_CANLF |
| 151 | Cytokine-cytokine receptor interaction | sp\|Q5ECR9\|CCR5\_CANLF, tr\|A0A5F4CUX4\|A0A5F4CUX4\_CANLF, tr\|E2RMN7\|E2RMN7\_CANLF, tr\|F1PCC1\|F1PCC1\_CANLF, tr\|F1PF03\|F1PF03\_CANLF, tr\|F1PTB2\|F1PTB2\_CANLF |
| 152 | Basal transcription factors | tr\|A0A5F4D2S8\|A0A5F4D2S8\_CANLF, tr\|E2R0P0\|E2R0P0\_CANLF, tr\|E2R680\|E2R680\_CANLF, tr\|F6Y258\|F6Y258\_CANLF, tr\|J9NS44\|J9NS44\_CANLF |
| 153 | Nucleotide excision repair | tr\|A0A5F4CGL9\|A0A5F4CGL9\_CANLF, tr\|A0A5F4CP79\|A0A5F4CP79\_CANLF, tr\|E2R4A5\|E2R4A5\_CANLF, tr\|F6URE1\|F6URE1\_CANLF, tr\|F6UYJ0\|F6UYJ0\_CANLF |
| 154 | Collecting duct acid secretion | tr\|A0A5F4D507\|A0A5F4D507\_CANLF, tr\|E2QXJ5\|E2QXJ5\_CANLF, tr\|F1PA63\|F1PA63\_CANLF, tr\|F1PDY8\|F1PDY8\_CANLF, tr\|Q2Z1P8\|Q2Z1P8\_CANLF |
| 155 | ABC transporters | sp\|Q6UR05\|MRP1\_CANLF, tr\|E2R872\|E2R872\_CANLF, tr\|F1P843\|F1P843\_CANLF, tr\|J9NU18\|J9NU18\_CANLF, tr\|Q5W417\|Q5W417\_CANLF |
| 156 | Glyoxylate and dicarboxylate metabolism | tr\|A0A5F4DIX0\|A0A5F4DIX0\_CANLF, tr\|E2QV08\|E2QV08\_CANLF, tr\|F1PV92\|F1PV92\_CANLF, tr\|F1PYG8\|F1PYG8\_CANLF, tr\|F6XNH4\|F6XNH4\_CANLF |
| 157 | Asthma | tr\|A0A5F4BUG1\|A0A5F4BUG1\_CANLF, tr\|A0A5F4CBH6\|A0A5F4CBH6\_CANLF, tr\|E2RCC8\|E2RCC8\_CANLF, tr\|E2RE31\|E2RE31\_CANLF, tr\|Q30348\|Q30348\_CANLF |
| 158 | Arachidonic acid metabolism | tr\|A0A5F4DG67\|A0A5F4DG67\_CANLF, tr\|F6UQ33\|F6UQ33\_CANLF, tr\|F6XH46\|F6XH46\_CANLF, tr\|F6Y290\|F6Y290\_CANLF, tr\|J9P028\|J9P028\_CANLF |
| 159 | Ether lipid metabolism | tr\|A0A5F4BTY2\|A0A5F4BTY2\_CANLF, tr\|A0A5F4BU16\|A0A5F4BU16\_CANLF, tr\|E2RLA8\|E2RLA8\_CANLF, tr\|F1PLR0\|F1PLR0\_CANLF, tr\|F6UQ33\|F6UQ33\_CANLF |
| 160 | Fatty acid elongation in mitochondria | tr\|A0A5F4DI81\|A0A5F4DI81\_CANLF, tr\|F1PAZ6\|F1PAZ6\_CANLF, tr\|F1PIP0\|F1PIP0\_CANLF, tr\|F6XHT8\|F6XHT8\_CANLF, tr\|J9NZC6\|J9NZC6\_CANLF |
| 161 | Phenylalanine metabolism | tr\|A0A5F4BUE3\|A0A5F4BUE3\_CANLF, tr\|A0A5F4CF81\|A0A5F4CF81\_CANLF, tr\|A0A5F4CNU9\|A0A5F4CNU9\_CANLF, tr\|A0A5F4DCR7\|A0A5F4DCR7\_CANLF, tr\|F1PXN6\|F1PXN6\_CANLF |
| 162 | Drug metabolism - other enzymes | tr\|A0A5F4DIU7\|A0A5F4DIU7\_CANLF, tr\|F1PTS5\|F1PTS5\_CANLF, tr\|J9NVT2\|J9NVT2\_CANLF, tr\|Q95N05\|Q95N05\_CANLF |
| 163 | Protein export | sp\|P33731\|SRP72\_CANLF, tr\|A0A5F4CVW1\|A0A5F4CVW1\_CANLF, tr\|F1PIC7\|F1PIC7\_CANLF, tr\|J9NUW8\|J9NUW8\_CANLF |
| 164 | Glycosaminoglycan degradation | tr\|F1P6L7\|F1P6L7\_CANLF, tr\|F1PRH5\|F1PRH5\_CANLF, tr\|F1PWH9\|F1PWH9\_CANLF, tr\|K0J6C5\|K0J6C5\_CANLF |
| 165 | Histidine metabolism | tr\|E2RQ99\|E2RQ99\_CANLF, tr\|F1PAB7\|F1PAB7\_CANLF, tr\|F1PRF8\|F1PRF8\_CANLF, tr\|F1PXN6\|F1PXN6\_CANLF |
| 166 | Biosynthesis of unsaturated fatty acids (no map in kegg database) | tr\|D9ILX1\|D9ILX1\_CANLF, tr\|E2RSN5\|E2RSN5\_CANLF, tr\|F1P6W6\|F1P6W6\_CANLF, tr\|F1PIP0\|F1PIP0\_CANLF |
| 167 | Sphingolipid metabolism | tr\|A0A5F4BU16\|A0A5F4BU16\_CANLF, tr\|E2RSX5\|E2RSX5\_CANLF, tr\|F1PWA9\|F1PWA9\_CANLF, tr\|F1PWH9\|F1PWH9\_CANLF |
| 168 | Nitrogen metabolism | tr\|A0A5F4CHX9\|A0A5F4CHX9\_CANLF, tr\|A0A5F4D507\|A0A5F4D507\_CANLF, tr\|F1PDY8\|F1PDY8\_CANLF, tr\|F1PPD7\|F1PPD7\_CANLF |
| 169 | Graft-versus-host disease | tr\|A0A5F4BUG1\|A0A5F4BUG1\_CANLF, tr\|O46880\|O46880\_CANLF, tr\|O46882\|O46882\_CANLF, tr\|Q30348\|Q30348\_CANLF |
| 170 | Valine, leucine and isoleucine biosynthesis | tr\|A0A5F4CTT0\|A0A5F4CTT0\_CANLF, tr\|E2RTJ7\|E2RTJ7\_CANLF, tr\|F6USV0\|F6USV0\_CANLF, tr\|J9P208\|J9P208\_CANLF |
| 171 | Fatty acid biosynthesis | tr\|A0A5F4CT67\|A0A5F4CT67\_CANLF, tr\|A0A5F4D6R1\|A0A5F4D6R1\_CANLF, tr\|F1PKR8\|F1PKR8\_CANLF |
| 172 | Pentose and glucuronate interconversions | tr\|A0A5F4DG67\|A0A5F4DG67\_CANLF, tr\|E2QY46\|E2QY46\_CANLF, tr\|J9P7R2\|J9P7R2\_CANLF |
| 173 | Base excision repair | sp\|Q6YKA4\|HMGB1\_CANLF, tr\|F6URE1\|F6URE1\_CANLF, tr\|J9PA46\|J9PA46\_CANLF |
| 174 | Glycine, serine and threonine metabolism | tr\|A0A5F4DCR7\|A0A5F4DCR7\_CANLF, tr\|E2RDI0\|E2RDI0\_CANLF, tr\|E2RQ99\|E2RQ99\_CANLF |
| 175 | alpha-Linolenic acid metabolism | tr\|D9ILX1\|D9ILX1\_CANLF, tr\|F1P6W6\|F1P6W6\_CANLF, tr\|F6UQ33\|F6UQ33\_CANLF |
| 176 | Ascorbate and aldarate metabolism | tr\|E2QTD8\|E2QTD8\_CANLF, tr\|E2RQ99\|E2RQ99\_CANLF, tr\|F1PAB7\|F1PAB7\_CANLF |
| 177 | Glycosphingolipid biosynthesis - ganglio series | tr\|F1PWH9\|F1PWH9\_CANLF, tr\|K0J6C5\|K0J6C5\_CANLF |
| 178 | Porphyrin and chlorophyll metabolism | tr\|E2QVU9\|E2QVU9\_CANLF, tr\|F1PZA0\|F1PZA0\_CANLF |
| 179 | Selenoamino acid metabolism | tr\|A0A5F4DF70\|A0A5F4DF70\_CANLF, tr\|E2R1E3\|E2R1E3\_CANLF |
| 180 | Hedgehog signaling pathway | tr\|A0A5F4CV51\|A0A5F4CV51\_CANLF, tr\|J9NXT4\|J9NXT4\_CANLF |
| 181 | D-Glutamine and D-glutamate metabolism | tr\|A0A5F4CHX9\|A0A5F4CHX9\_CANLF, tr\|F1PPD7\|F1PPD7\_CANLF |
| 182 | Retinol metabolism | tr\|A0A5F4BZU6\|A0A5F4BZU6\_CANLF, tr\|J9P9J4\|J9P9J4\_CANLF |
| 183 | Sulfur metabolism | sp\|Q29476\|ST1A1\_CANLF, tr\|E2R1E3\|E2R1E3\_CANLF |
| 184 | Regulation of autophagy | tr\|A0A5F4CD52\|A0A5F4CD52\_CANLF, tr\|F1PJ26\|F1PJ26\_CANLF |
| 185 | Terpenoid backbone biosynthesis | tr\|A0A5F4C1U9\|A0A5F4C1U9\_CANLF, tr\|J9JHD4\|J9JHD4\_CANLF |
| 186 | Steroid hormone biosynthesis | tr\|A0A5F4DKQ5\|A0A5F4DKQ5\_CANLF, tr\|Q60F99\|Q60F99\_CANLF |
| 187 | Other glycan degradation (no map in kegg database) | tr\|F1PWH9\|F1PWH9\_CANLF, tr\|K0J6C5\|K0J6C5\_CANLF |
| 188 | Phenylalanine, tyrosine and tryptophan biosynthesis | tr\|A0A5F4BUE3\|A0A5F4BUE3\_CANLF, tr\|A0A5F4CNU9\|A0A5F4CNU9\_CANLF |
| 189 | DNA replication | tr\|F6URE1\|F6URE1\_CANLF, tr\|J9P937\|J9P937\_CANLF |
| 190 | Synthesis and degradation of ketone bodies | tr\|F1PJ13\|F1PJ13\_CANLF, tr\|J9JHD4\|J9JHD4\_CANLF |
| 191 | RNA polymerase | tr\|A0A5F4D0K7\|A0A5F4D0K7\_CANLF, tr\|E2R8N8\|E2R8N8\_CANLF |
| 192 | One carbon pool by folate | tr\|F1P797\|F1P797\_CANLF, tr\|F6XNH4\|F6XNH4\_CANLF |
| 193 | Renin-angiotensin system | tr\|A0A5F4CIA0\|A0A5F4CIA0\_CANLF, tr\|F1PCV3\|F1PCV3\_CANLF |
| 194 | Glycosaminoglycan biosynthesis - keratan sulfate (no map in kegg database) | tr\|F1PGZ1\|F1PGZ1\_CANLF |
| 195 | Linoleic acid metabolism | tr\|F6UQ33\|F6UQ33\_CANLF |
| 196 | Circadian rhythm - mammal | tr\|E2RRW0\|E2RRW0\_CANLF |
| 197 | Taurine and hypotaurine metabolism | tr\|F1PRF8\|F1PRF8\_CANLF |
| 198 | Primary bile acid biosynthesis | tr\|F1Q3R2\|F1Q3R2\_CANLF |
| 199 | Lysine biosynthesis | tr\|E2RQ99\|E2RQ99\_CANLF |
| 200 | Steroid biosynthesis | tr\|E2QV27\|E2QV27\_CANLF |
| 201 | Sulfur relay system | tr\|E2R3I2\|E2R3I2\_CANLF |
| 202 | Glycosphingolipid biosynthesis - globo series | tr\|K0J6C5\|K0J6C5\_CANLF |
| 203 | Caffeine metabolism | tr\|F1PTS5\|F1PTS5\_CANLF |
| 204 | Glycosphingolipid biosynthesis - lacto and neolacto series | tr\|F1PGZ1\|F1PGZ1\_CANLF |
| 205 | Mismatch repair | tr\|F6URE1\|F6URE1\_CANLF |
| 206 | Pantothenate and CoA biosynthesis | tr\|F1PAX0\|F1PAX0\_CANLF |
| 207 | Folate biosynthesis | tr\|J9P7N1\|J9P7N1\_CANLF |
| 208 | Thiamine metabolism | tr\|A0A5F4D5L1\|A0A5F4D5L1\_CANLF |
| 209 | Non-homologous end-joining | tr\|J9P1R2\|J9P1R2\_CANLF |
| 210 | Basal cell carcinoma | tr\|A0A5F4D0Z3\|A0A5F4D0Z3\_CANLF |
| 211 | Butirosin and neomycin biosynthesis | tr\|F1PPF7\|F1PPF7\_CANLF |
