# Supplementary material for: Phosphoproteomics to Characterize Host Response During H3N2 Canine Influenza Virus Infection of Dog Lung
Source: Front Vet Sci. 2020 Dec 3;7:585071. doi: 10.3389/fvets.2020.585071 (PMC7744373; doi:10.3389/fvets.2020.585071)
Supplement: Supplementary file 1 [file Data_Sheet_1.ZIP › Project_data/Motif/MoMo results.html]

MoMo results


MoMo outputs a tab-separated values (TSV) file ('momo.tsv') that
contains one line for each motif found and that is suitable
for parsing by scripts and viewing with Excel.
The first line in the file contains the (tab-separated) names of the fields.
Your command line is given at the end of the TSV file in a comment line
starting with the character '#'.
The names and meanings of each of the fields are described in the table below.
Not all fields are present for all algorithms, and the field numbers are
indicated for each algorithm in the first three columns of the table.

| Field Number | | | Field Name | Field Contents |
| --- | --- | --- | --- | --- |
| motif-x | MoDL | simple |  |  |
| 1 | 1 | 1 | mod | The post-translationally modified residue located at the center of the motif. |
| 2 | 2 | 2 | motif | (A string similar to) a regular expression describing the motif. Lower case 'x' represents a match to any residue the non-central positions. The central peptide and its modification weight (if any) is surrounded by '\_' characters. If option `--single-motif-per-mass` was specified, the central peptide will be represented by an uppercase 'X', which also represents a match to any residue. With the simple algorithm, all non-central positions will contain an 'x', regardless of which residues appeared in the input sequences. (See the Regular Expression column of the output for further information.) Motifs are reported in the order in which they are found by MoMo, and are not sorted in any way. |
| 3 | 3 | 3 | regexp | A PERL regular expression suitable for searching for the motif in protein sequences using fasta-grep or PERL scripts. The PERL wildcard character '.' matches any resudue. Groups of residues in square brackets '[ ]' match any of those residues. For the motif-x and MoDL algorithms, this is the regular expression for the motif as output by that algorithm. For the simple algorithm, this regular expression includes all of the residues in the motif occurrences that match the motif. |
| 4 | 4 |  | score | The algorithm-dependent score of the motif. For algorithm motif-x, this is the sum over the significant position/residue pairs of -log(p_binomial_); for algorithm MoDL, this is the increase in description length in bits if the motif were to be removed from the final set of motifs. |
| 5 | 5 | 4 | fg\_match | fg\_match is the number of foreground peptides that match the motif. **Note:** For motif-x, the foreground counts are based on **all** the peptides unless the `--harvard` option is specified, in which case the counts are based on the **remaining** peptides. |
| 6 | 6 |  | fg\_size | fg\_size is the total number of foreground peptides with the given central modification. |
| 7 | 7 |  | bg\_match | bg\_match is the number of background peptides that match the motif. **Note:** For motif-x, the background counts are based on **all** the peptides unless the `--harvard` option is specified, in which case the counts are based on the **remaining** peptides. |
| 8 | 8 |  | bg\_size | bg\_size is the total number of background peptides with the given central modification. |
| 9 | 9 |  | fg/bg | The fold enrichment of the foreground matches vs. the background matches. This is equal to:     (fg\_match / fg\_size) / (bg\_match / bg\_size). |
| 10 | 10 |  | unadjusted\_p-value | The *p*-value of the Fisher Exact test on the enrichment of the motif in the foreground vs. the background peptides. This value does **not** accurately represent the statistical significance of the motif, and should be interpreted as a **score** only. |
| 11 |  |  | tests | The number of independent tests performed in the search for the motif, which is the number of position/residue pairs algorithm motif-x tested for statistical significance. **Note:** This field only appears for motif-x, and only when the `--db-background` option is **not** used. |
| 12 |  |  | adjusted\_p-value | The *p*-value of the Fisher Exact test on the enrichment of the motif in the foreground vs. the background peptides, adjusted for the number of independent tests (1 - (1-pvalue)^tests^). **Note 1:** This value **does** accurately represent the statistical significance of the motif. **Note 2:** This field only appears for motif-x, and only when the `--db-background` option is **not** used. |

[
close ]

MoMo outputs a file ('momo.txt') that contains each discovered motif
in MEME motif format
that is suitable for use with other MEME Suite programs.
MoMo creates these position-frequency matrix motifs by aligning the foreground
peptides matching the motif and computing the position-frequency matrix.
No pseudo-counts are added.

For motif-x motifs where the adjusted
*p*-value can be accurately calculated (e.g., you did not specify the
`--db-background` option), MoMo writes the value of
`tests` times the `unadjusted_p-value`
in the *E*-value field. In all other cases, MoMo writes
'1' in the *E*-value field of the motif.

[
close ]

MoMo creates a sequence logo for each motif it discovers using
the ceqlogo utility.

[
close ]

The post-translationally modified residue located
at the center of the motif.

[
close ]

(A string similar to) a regular expression describing the motif.
Lower case 'x' represents a match to any residue the non-central
positions.
The central peptide and its modification weight (if any) is surrounded
by '\_' characters. If option `--single-motif-per-mass`
was specified, the central peptide will be represented by an uppercase 'X',
which also represents a match to any residue. With the simple algorithm,
all non-central positions will contain an 'x', regardless of which residues appeared in
the input sequences. (See the Regular Expression column of the output
for further information.) Motifs are reported in the order
in which they are found by MoMo, and are not sorted in any way.

[
close ]

A PERL regular expression suitable for searching for the motif in
protein sequences using
fasta-grep
or PERL scripts. The PERL wildcard character '.' matches any
resudue. Groups of residues in square brackets '[ ]' match
any of those residues. For the motif-x and MoDL algorithms,
this is the regular expression for the motif as output by that
algorithm. For the simple algorithm,
this regular expression includes all of the residues
in the motif occurrences that match the motif.

[
close ]

The algorithm-dependent score of the motif.
For algorithm motif-x, this is the sum over the significant
position/residue pairs of -log(p_binomial_);
for algorithm MoDL, this is the increase in description length in
bits if the motif were to be removed from the final set of motifs.

[
close ]

For the simple algorithm: fg\_match   
For the other algorithms: fg\_match / fg\_size   

fg\_size is the total number of foreground peptides with the given
central modification.

fg\_match is the number of foreground peptides that match the motif.
**Note:** For motif-x, the foreground counts are based on
**all** the peptides unless the
`--harvard` option is specified, in which
case the counts are based on the **remaining** peptides.

[
close ]

For the simple algorithm: bg\_match   
For the other algorithms: bg\_match / bg\_size   

bg\_size is the total number of background peptides with the given
central modification.

bg\_match is the number of background peptides that match the motif.
**Note:** For motif-x, the background counts are based on
**all** the peptides unless the
`--harvard` option is specified, in which
case the counts are based on the **remaining** peptides.

[
close ]

The fold enrichment of the foreground matches vs. the background
matches. This is equal to:   
  (fg\_match / fg\_size) / (bg\_match / bg\_size).

[
close ]

The *p*-value of the Fisher Exact test on the
enrichment of the motif in the foreground vs. the background peptides.
This value does **not** accurately represent the statistical
significance of the motif, and should be interpreted
as a **score** only.

[
close ]

The number of independent tests performed in the search for the motif,
which is the number of position/residue pairs algorithm motif-x
tested for statistical significance. **Note:** This field only appears for
motif-x, and only when the `--db-background` option is **not** used.

[
close ]

The *p*-value of the Fisher Exact test on the
enrichment of the motif in the foreground vs. the background
peptides, adjusted for the number of independent tests
(1 - (1-pvalue)^tests^). **Note 1:** This value **does**
accurately represent the statistical significance of the motif.
**Note 2:** This field only appears for motif-x, and only when the
`--db-background`
option is **not** used.

[
close ]

The modified peptides matching the motif.
**Note:** For motif-x, the only the the
occurrences that are not covered by a previous
motif are shown, so there may be fewer occurrences
than indicated by `FG` unless you
specified the `--harvard` option.

[
close ]

The log of steps taken by the MoDL algorithm in discovering the
group of motifs. The log is only shown on the line for the
first motif in a group output by MoDL.
The description length (DL) given the motifs in the group is
reported for each step, followed by the motifs in the group.
The step (e.g., group of motifs)
achieving the lowest description length is preceded by an aserisk ('\*').

The steps are followed by three lines stating the
final step number, the description length of the motifs in
the final group, and the decrease in description length
relative to the empty group (Step 0).

[
close ]

# MoMo

## Modification Motifs

For further information on how to interpret these results please access
http://meme-suite.org//doc/momo-output-format.html.  
To get a copy of the MEME software please access
http://meme-suite.org.

If you use MoMo in your research, please cite the following paper:  

Alice Cheng, Charles Grant, Timothy L. Bailey and William Noble,
"MoMo: Discovery of statistically significant post-translational modification motifs", *Bioinformatics*, 2018.
[full text]

Discovered Motifs
  |  
Inputs & Settings
  |  
Program information
  |  
Results in TSV Format 


  |  
Results in MEME Format


# Javascript is required to view these results!


## Discovered Motifs

| Motif Logo | Mod | Motif | Regular Expression | Score | FG | BG | Fold Enrichment | Unadjusted *p*-value | Tests | Adjusted *p*-value | Motif Occurrences | MoDL Log |
| --- | --- | --- | --- | --- | --- | --- | --- | --- | --- | --- | --- | --- |
|  | S | xxxRxx\_S\_Pxxxxx | ...R..SP..... | 362.52 | 133/2644 | 4745/929339 | 9.9 | 6.5e-83 | 370 | 0.0e0 |  |  |
|  | S | xxxxxx\_S\_PxxxRx | ......SP...R. | 292.73 | 116/2644 | 4714/929339 | 8.6 | 1.3e-66 | 358 | 0.0e0 |  |  |
|  | S | xxxxPx\_S\_Pxxxxx | ....P.SP..... | 238.31 | 170/2644 | 8528/929339 | 7.0 | 7.4e-84 | 355 | 0.0e0 |  |  |
|  | S | xxxxxx\_S\_PxxxxK | ......SP....K | 173.15 | 77/2644 | 3143/929339 | 8.6 | 1.6e-44 | 338 | 0.0e0 |  |  |
|  | S | xxxxSx\_S\_Pxxxxx | ....S.SP..... | 148.83 | 171/2644 | 8212/929339 | 7.3 | 3.9e-87 | 332 | 0.0e0 |  |  |
|  | S | xLxRSx\_S\_xxxxxx | .L.RS.S...... | 159.64 | 32/2644 | 648/929339 | 17.4 | 3.5e-28 | 323 | 0.0e0 |  |  |
|  | S | xxxRSx\_S\_xxxxxx | ...RS.S...... | 128.20 | 171/2644 | 6881/929339 | 8.7 | 1.4e-98 | 313 | 0.0e0 |  |  |
|  | S | xxxxxx\_S\_Pxxxxx | ......SP..... | 112.57 | 920/2644 | 71072/929339 | 4.5 | 1.7e-345 | 310 | 0.0e0 |  |  |
|  | S | xxxxxx\_S\_DxExEx | ......SD.E.E. | 166.50 | 45/2644 | 562/929339 | 28.1 | 9.7e-48 | 296 | 0.0e0 |  |  |
|  | S | xxxRxx\_S\_xExxxx | ...R..S.E.... | 108.35 | 98/2644 | 4064/929339 | 8.5 | 1.1e-55 | 270 | 0.0e0 |  |  |
|  | S | xxxxxx\_S\_DxExDx | ......SD.E.D. | 115.78 | 26/2644 | 349/929339 | 26.2 | 1.8e-27 | 276 | 0.0e0 |  |  |
|  | S | xxxRTx\_S\_xxxxxx | ...RT.S...... | 75.78 | 67/2644 | 3156/929339 | 7.5 | 2.7e-35 | 245 | 0.0e0 |  |  |
|  | S | xxxxxx\_S\_DxExxx | ......SD.E... | 85.23 | 136/2644 | 4205/929339 | 11.4 | 2.4e-92 | 264 | 0.0e0 |  |  |
|  | S | xxxRxx\_S\_xDxxxx | ...R..S.D.... | 59.63 | 71/2644 | 2545/929339 | 9.8 | 1.1e-44 | 222 | 0.0e0 |  |  |
|  | S | xxxxxx\_S\_EEExxx | ......SEEE... | 66.07 | 34/2644 | 1024/929339 | 11.7 | 1.7e-24 | 230 | 0.0e0 |  |  |
|  | S | xxxRxx\_S\_xxDxxx | ...R..S..D... | 41.31 | 54/2644 | 2596/929339 | 7.3 | 2.5e-28 | 208 | 0.0e0 |  |  |
|  | S | xxxxxx\_S\_xEDxxx | ......S.ED... | 46.49 | 54/2644 | 4283/929339 | 4.4 | 1.1e-18 | 220 | 0.0e0 |  |  |
|  | S | xxxxxx\_S\_xDExxx | ......S.DE... | 39.70 | 72/2644 | 3406/929339 | 7.4 | 1.0e-37 | 217 | 0.0e0 |  |  |
|  | S | xxxRxx\_S\_xxxxxx | ...R..S...... | 29.10 | 590/2644 | 56997/929339 | 3.6 | 9.3e-164 | 197 | 0.0e0 |  |  |
|  | S | xRxxSx\_S\_xxxxxx | .R..S.S...... | 31.58 | 86/2644 | 6884/929339 | 4.4 | 1.3e-28 | 212 | 0.0e0 |  |  |
|  | S | xxxxxx\_S\_xExxxx | ......S.E.... | 24.75 | 461/2644 | 59934/929339 | 2.7 | 1.3e-82 | 180 | 0.0e0 |  |  |
|  | S | xxxxxx\_S\_xDxxDx | ......S.D..D. | 22.20 | 37/2644 | 2516/929339 | 5.2 | 3.0e-15 | 165 | 0.0e0 |  |  |
|  | S | xxxxRx\_S\_xxxxxx | ....R.S...... | 15.54 | 243/2644 | 51906/929339 | 1.6 | 7.6e-14 | 161 | 0.0e0 |  |  |
|  | S | xxxxSx\_S\_xxxxxx | ....S.S...... | 18.07 | 561/2644 | 99649/929339 | 2.0 | 3.8e-55 | 164 | 0.0e0 |  |  |
|  | S | xxxxxx\_S\_Exxxxx | ......SE..... | 12.15 | 249/2644 | 55817/929339 | 1.6 | 4.8e-12 | 140 | 0.0e0 |  |  |
|  | S | xxxxxx\_S\_xxDxxx | ......S..D... | 8.64 | 237/2644 | 44662/929339 | 1.9 | 2.1e-19 | 128 | 0.0e0 |  |  |
|  | S | xxxSxx\_S\_xxxxxx | ...S..S...... | 9.90 | 297/2644 | 98626/929339 | 1.1 | 1.6e-1 | 118 | 0.0e0 |  |  |
|  | S | xxxxxx\_S\_xPxxxx | ......S.P.... | 8.56 | 267/2644 | 67212/929339 | 1.4 | 4.2e-8 | 86 | 0.0e0 |  |  |
|  | S | xxxxxx\_S\_xxExxx | ......S..E... | 6.01 | 458/2644 | 64309/929339 | 2.5 | 5.9e-72 | 62 | 0.0e0 |  |  |
|  | T | xxxxxx\_T\_PPxxxx | ......TPP.... | 62.98 | 29/252 | 3473/582801 | 19.3 | 8.6e-28 | 52 | 0.0e0 |  |  |
|  | T | xxxxxx\_T\_Pxxxxx | ......TP..... | 35.96 | 107/252 | 40334/582801 | 6.1 | 6.1e-56 | 38 | 0.0e0 |  |  |
|  | T | xxxxxx\_T\_Sxxxxx | ......TS..... | 8.48 | 38/252 | 50496/582801 | 1.7 | 6.0e-4 | 10 | 0.0e0 |  |  |

## Inputs & Settings

#### Algorithm

- **Name:** motif-x
- **Motif Width:** 13
- **Minimum Occurrences for Residue/Position pair:** 20
- **Binomial probability threshold for Residue/Position pair:** 0.000001
- **Binomial *p*-value calculations:** accurate

#### Post-translationally modified peptides

| **File Name** | File Type |
| --- | --- |
| pep4motif.txt | Raw |

- **Number of Mods:** 2995
- **Number of Mod Types:** 3
- **Number of Mods Passing Filters:** 2896
- **Number of Mod Types Passing Filters:** 2

#### Protein Database and Control Peptides

- **Protein Database:** uniprot-taxonomy\_Canis\_lupus\_familiaris.fasta
- **Protein Database Format:** FASTA
- **Control peptides extracted from protein database:** 1512140

#### Other Settings

|  |  |
| --- | --- |
| filter foreground peptides? | no |
| remove peptides if they contain 'X'? | yes |
| eliminate duplicate peptides? | yes, if their 13 central residues match |
| generate single motif per mass? | no |
| random number seed | 0 |

##### MoMo version

5.1.1
(Release date: Wed Jan 29 15:00:42 2020 -0800)

##### Command line summary
