# Supplementary material for: Phosphoproteomics to Characterize Host Response During H3N2 Canine Influenza Virus Infection of Dog Lung
Source: Front Vet Sci. 2020 Dec 3;7:585071. doi: 10.3389/fvets.2020.585071 (PMC7744373; doi:10.3389/fvets.2020.585071)
Supplement: Supplementary file 1 [file Data_Sheet_1.ZIP › Project_data/Pictures/Figure3.3_uniqP.pdf]

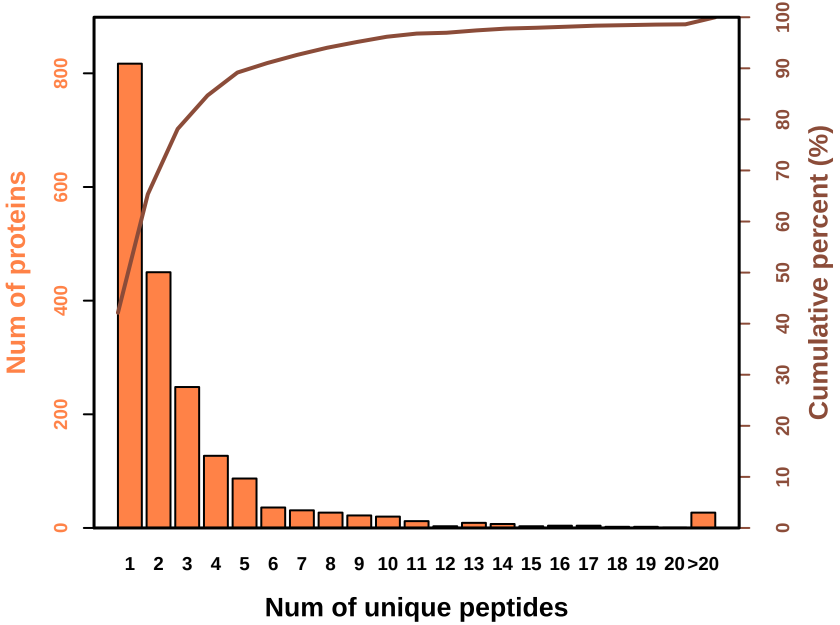
Num of proteins
0
200
400
600
800
Cumulative percent (%)
0
10
20
30
40
50
60
70
80
90
100
1 2 3 4 5 6 7 8 9 10 11 12 13 14 15 16 17 18 19 20 >20
Num of unique peptides
