# Supplementary material for: Phosphoproteomics to Characterize Host Response During H3N2 Canine Influenza Virus Infection of Dog Lung
Source: Front Vet Sci. 2020 Dec 3;7:585071. doi: 10.3389/fvets.2020.585071 (PMC7744373; doi:10.3389/fvets.2020.585071)
Supplement: Supplementary file 1 [file Data_Sheet_1.ZIP › Project_data/Pictures/Figure3.4_Phos_site.pdf]

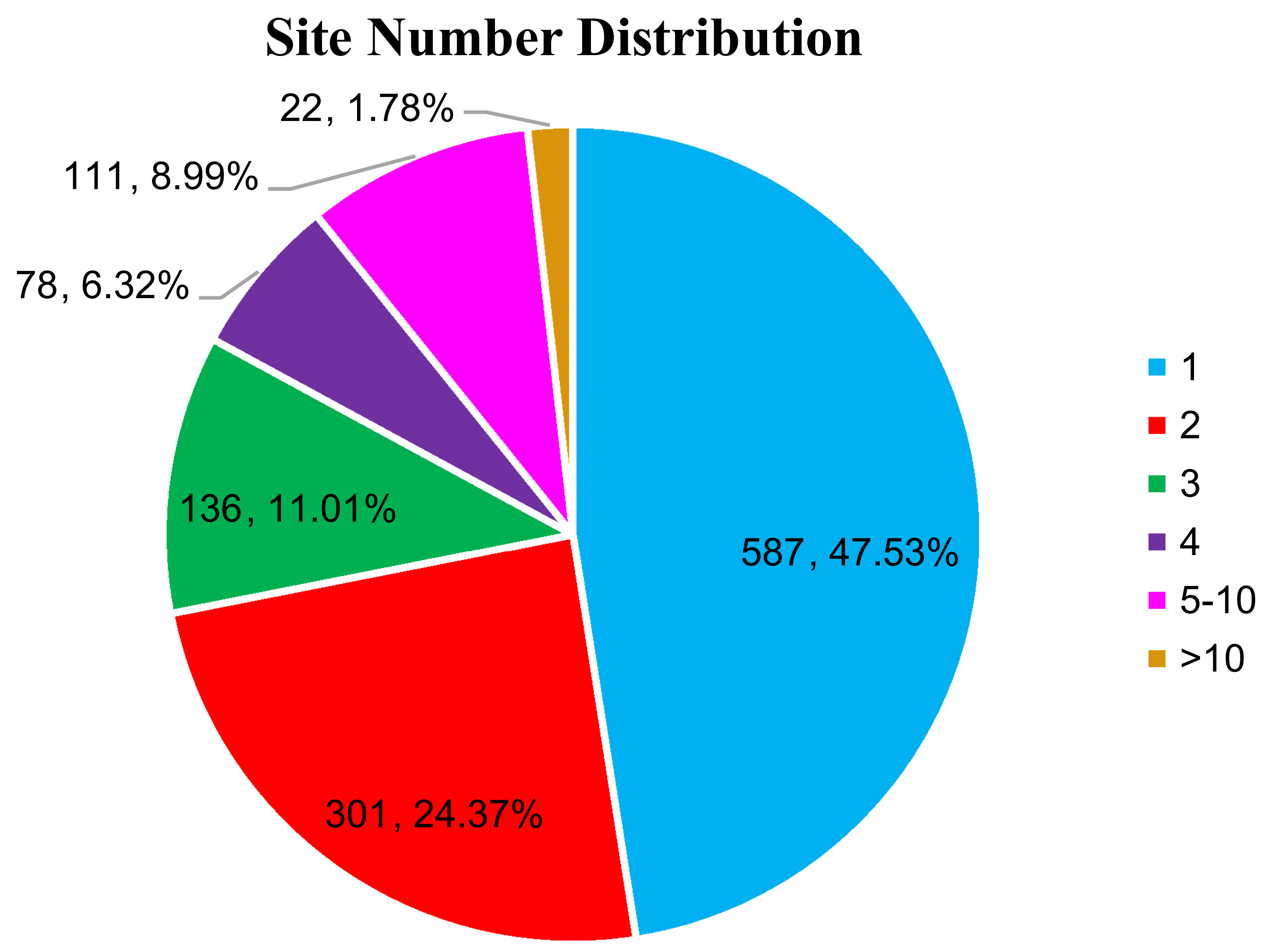
Site Number Distribution
22, 1.78%
111, 8.99%
78, 6.32%
136, 11.01%
587, 47.53%
301, 24.37%
1
2
3
4
5-10
>10
