# Supplementary material for: Phosphoproteomics to Characterize Host Response During H3N2 Canine Influenza Virus Infection of Dog Lung
Source: Front Vet Sci. 2020 Dec 3;7:585071. doi: 10.3389/fvets.2020.585071 (PMC7744373; doi:10.3389/fvets.2020.585071)
Supplement: Supplementary file 1 [file Data_Sheet_1.ZIP › Project_data/Pictures/Figure3.6_pepSeq.pepLength.pdf]

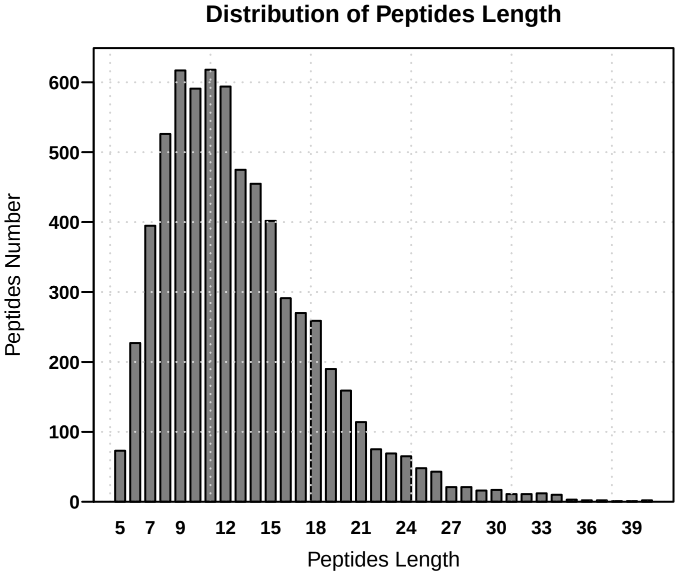

Distribution of Peptides Length
Peptides Number
Peptides Length
0
100
200
300
400
500
600
5
7
9
12
15
18
21
24
27
30
33
36
39
