# Supplementary material for: Phosphoproteomics to Characterize Host Response During H3N2 Canine Influenza Virus Infection of Dog Lung
Source: Front Vet Sci. 2020 Dec 3;7:585071. doi: 10.3389/fvets.2020.585071 (PMC7744373; doi:10.3389/fvets.2020.585071)
Supplement: Supplementary file 1 [file Data_Sheet_1.ZIP › Project_data/Pictures/Figure4.1_phospepRatiodistribution.pdf]

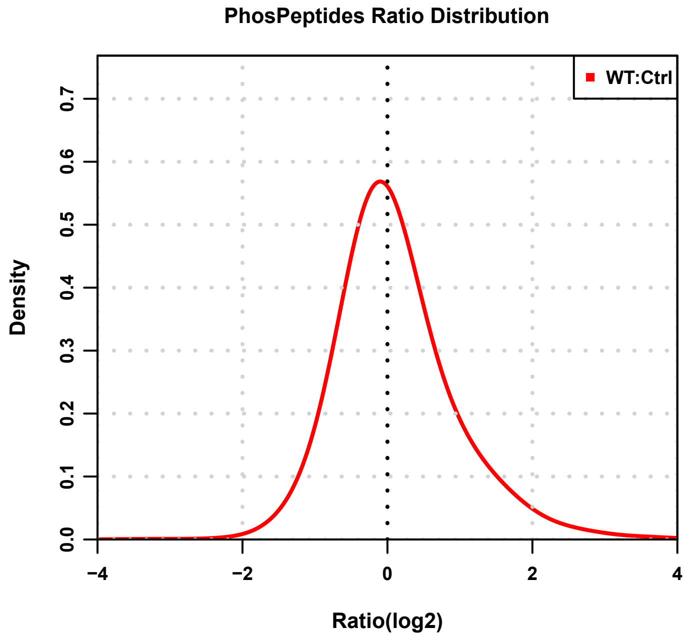
PhosPeptides Ratio Distribution
WT:Ctrl
Density
0.0
0.1
0.2
0.3
0.4
0.5
0.6
0.7
−4
−2
0
2
4
Ratio(log2)
