# Supplementary material for: Phosphoproteomics to Characterize Host Response During H3N2 Canine Influenza Virus Infection of Dog Lung
Source: Front Vet Sci. 2020 Dec 3;7:585071. doi: 10.3389/fvets.2020.585071 (PMC7744373; doi:10.3389/fvets.2020.585071)

biological_process

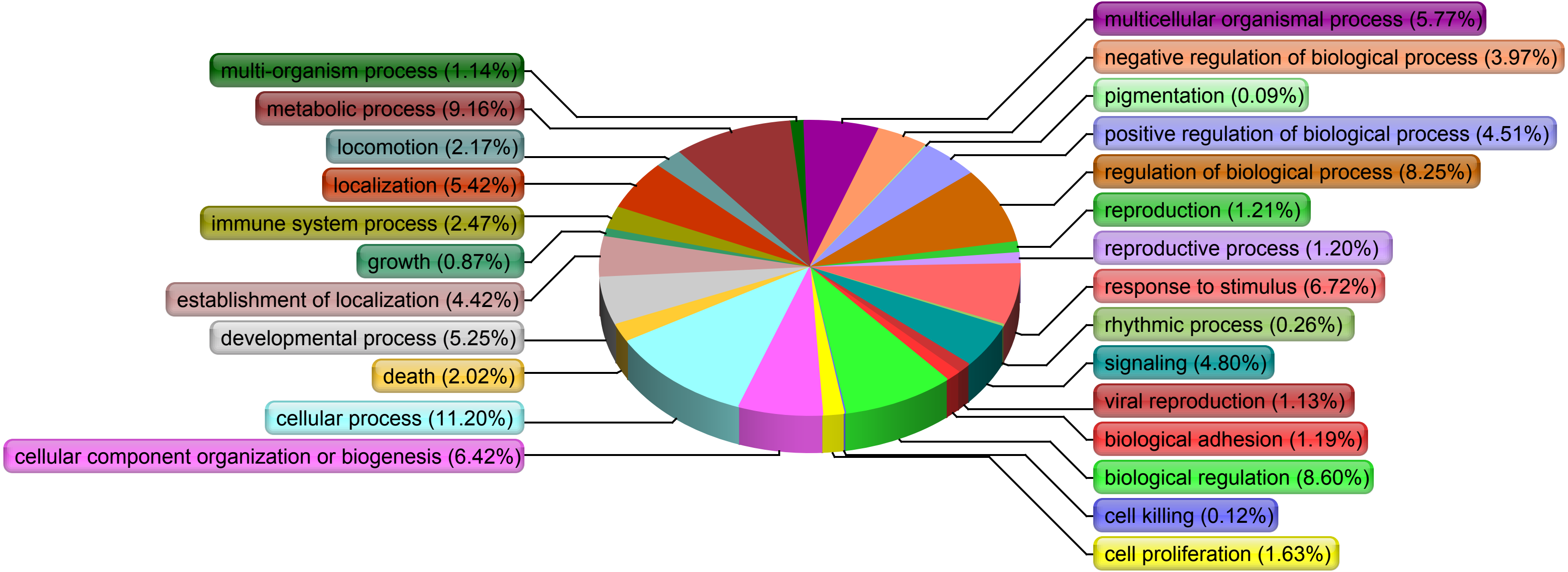

Supplement: Supplementary file 1 [file Data_Sheet_1.ZIP › Project_data/Pictures/Figure6.2_biological_process.pdf]
