# Supplementary material for: Phosphoproteomics to Characterize Host Response During H3N2 Canine Influenza Virus Infection of Dog Lung
Source: Front Vet Sci. 2020 Dec 3;7:585071. doi: 10.3389/fvets.2020.585071 (PMC7744373; doi:10.3389/fvets.2020.585071)
Supplement: Supplementary file 1 [file Data_Sheet_1.ZIP › Project_data/Pictures/Figure6.3_All_ID.fa.cog.pdf]

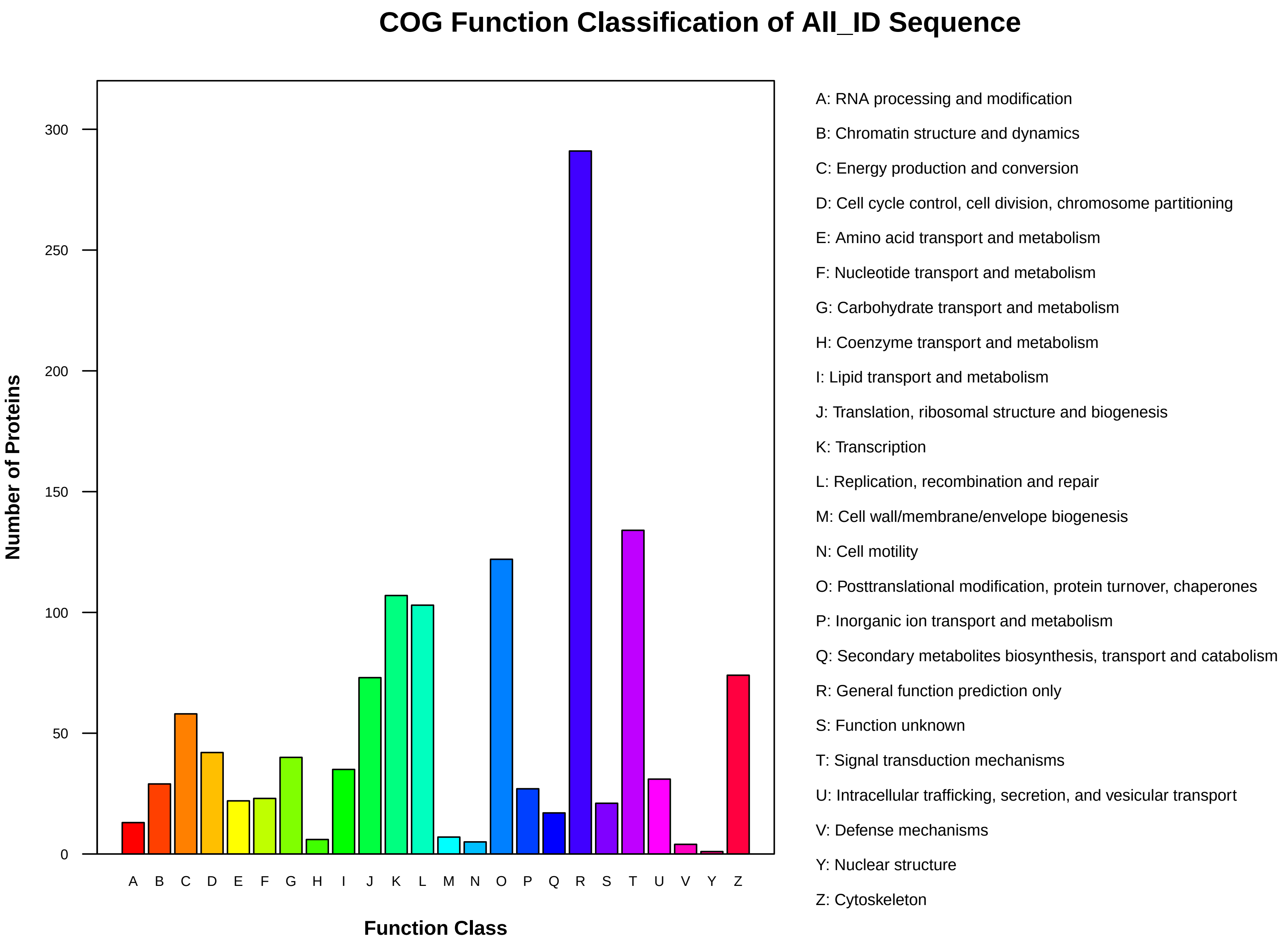
COG Function Classification of All_ID Sequence
Number of Proteins
0
50
100
150
200
250
300
A B C D E F G H I J K L M N O P Q R S T U V Y Z
Function Class
A: RNA processing and modification
B: Chromatin structure and dynamics
C: Energy production and conversion
D: Cell cycle control, cell division, chromosome partitioning
E: Amino acid transport and metabolism
F: Nucleotide transport and metabolism
G: Carbohydrate transport and metabolism
H: Coenzyme transport and metabolism
I: Lipid transport and metabolism
J: Translation, ribosomal structure and biogenesis
K: Transcription
L: Replication, recombination and repair
M: Cell wall/membrane/envelope biogenesis
N: Cell motility
O: Posttranslational modification, protein turnover, chaperones
P: Inorganic ion transport and metabolism
Q: Secondary metabolites biosynthesis, transport and catabolism
R: General function prediction only
S: Function unknown
T: Signal transduction mechanisms
U: Intracellular trafficking, secretion, and vesicular transport
V: Defense mechanisms
Y: Nuclear structure
Z: Cytoskeleton
